# Discovery of triazole-tethered glycinate and propanoate derivatives bearing a thiolactone moiety as quorum sensing inhibitors of *Pseudomonas aeruginosa*: design, synthesis, biological evaluation, and biofilm inhibition

**DOI:** 10.1039/d6ra00717a

**Published:** 2026-05-22

**Authors:** Kosana Sai Chaitanya, Tsz Tin Yu, Hrushikesh Chaudhari, Nidhi Orenkonday, Pranali Vijaykumar Kuthe, Naresh Kumar, Ruchi Jain Dey, Sankaranarayanan Murugesan, Kondapalli Venkata Gowri Chandra Sekhar

**Affiliations:** a Department of Chemistry, Birla Institute of Technology and Science Pilani, Hyderabad Campus, Jawahar Nagar Hyderabad – 500 078 Telangana India kvgc@hyderabad.bits-pilani.ac.in kvgcs.bits@gmail.com +91 40 66303527; b School of Chemistry, The University of New South Wales Sydney NSW 2052 Australia; c Department of Biological Sciences, Birla Institute of Technology and Science Pilani, Hyderabad Campus, Jawahar Nagar Hyderabad – 500 078 Telangana India; d Department of Pharmacy, Birla Institute of Technology and Science Pilani Campus, Vidya Vihar Pilani-333031 Rajasthan India

## Abstract

Quorum sensing is the bacterial communication that regulates biofilm formation, virulence, and drug resistance development. The misuse of antibiotics accelerates the emergence of resistant pathogens, highlighting the urgent need for alternative anti-virulence strategies. In this context, LasR, a key transcriptional regulator in the QS network of *Pseudomonas aeruginosa*, was targeted to disrupt bacterial communication and biofilm development. In the present study, we designed a library of glycinate and propanoate derivatives (*n* = 30), and carried out molecular docking, MM-GBSA studies, synthesized and characterized. Their QS inhibitory activity was evaluated against the *P. aeruginosa* MH602 reporter strain at concentrations ranging from 250 to 8 µM. The compounds exhibited 79–35% inhibition at 250 µM, retaining moderate to low activity (28–7%) at 8 µM. SAR studies indicated that the electron-withdrawing phenyl substituents on the triazole ring enhanced activity, with 11b and 10o (3-nitrophenyl) showing the highest inhibition. *In silico* ADME, molecular dynamics studies supported favorable LasR binding. The most active compounds were evaluated for cytotoxicity, biofilm inhibition, and suppression of pyocyanin and protease production. 10o emerged as the most promising, demonstrating strong anti-biofilm activity and significant reduction of pyocyanin, suggesting thiolactone-based triazoles as potential QS inhibitors to combat bacterial resistance.

## Abstract 2

Quorum sensing regulates biofilm, virulence and resistance in bacteria. Thiolactone-based 1,2,3-triazole hybrids were synthesized as LasR-targeting inhibitors against *Pseudomonas aeruginosa*. Compounds 10o and 11b showed strong biofilm inhibition.

## Graphical abstract

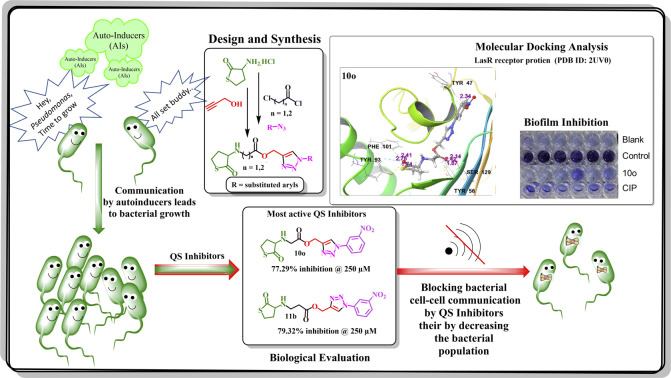

## 1 Introduction

The discovery of antibiotics transformed modern medicine by allowing the effective treatment of bacterial infections, but their overuse has contributed to the emergence of antibiotic-resistant bacteria, which have diminished therapeutic effectiveness and become a significant global public health threat.^1,2^ Antimicrobial resistance (AMR) is caused by selective survival pressure that generates multidrug-resistant organisms. The Institute for Health Metrics and Evaluation (IHME) estimates that AMR is responsible for around 1.14 million deaths worldwide, mainly caused by lower respiratory and blood stream infections.^3^ The current treatment guidelines recommended by the Infectious Disease Society of America (IDSA) are based on last-resort antibiotics, such as carbapenems or combination therapy, but these are accompanied by high cost, long hospitalization, and severe side effects.^4^

**Fig. 1:**
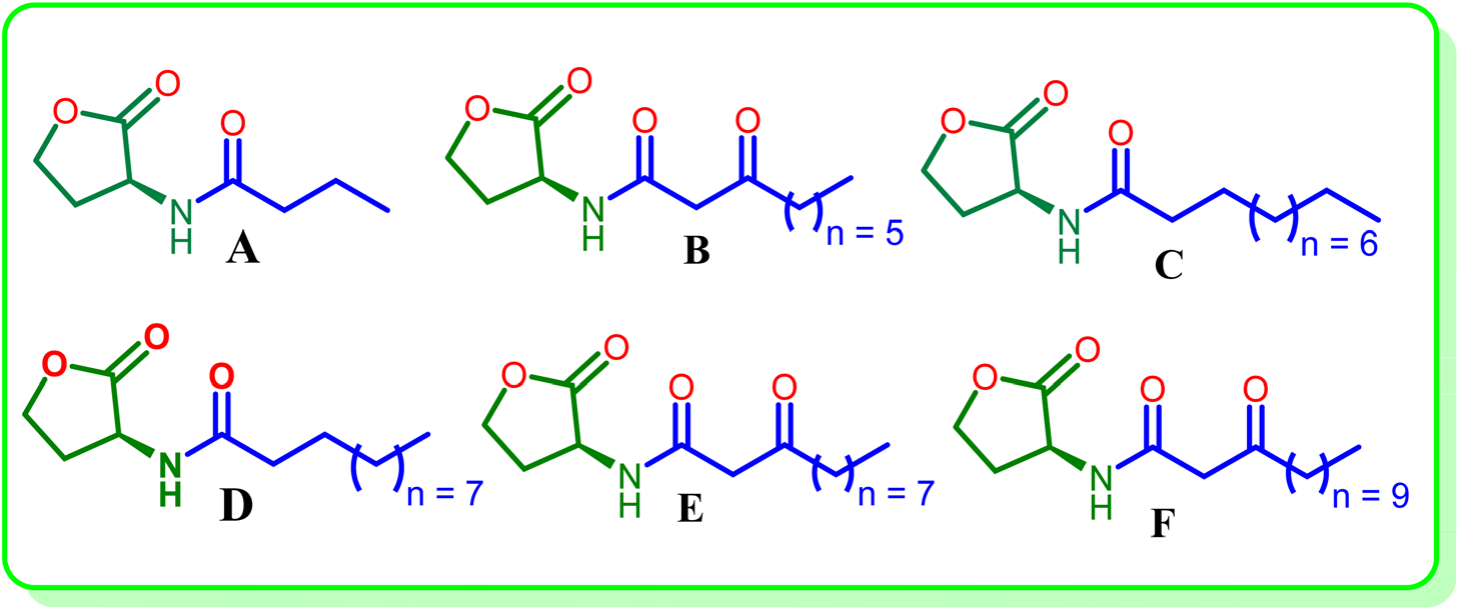
The common AHLs in *P. aeruginosa*.

Quorum sensing (QS) inhibition is a potential alternative approach.^5,6^ It is an inter-bacterial communication process mediated by autoinducers that controls gene regulation, virulence and biofilm formation, decreases pathogenicity and increases antibiotic sensitivity.^7–9^ In Gram-negative bacteria, QS mainly consists of *N*-acyl homoserine lactones (AHLs) and their receptors.^10–13^ As a model organism, *Pseudomonas aeruginosa* has two QS regulons (LasI/R and RhlI/R) that control virulence *via* AHLs, including 3-oxo-C12-HSL and C4-HSL. The common AHLs released by *P. aeruginosa* include l-homoserine lactones of various acyl side chains (A–F) as shown in Fig. 1.^14–19^

LasR is a key transcriptional regulator in quorum sensing and an attractive target for inhibition, whereas AHL signal generation is carried out by the synthase LasI. Autoinducer structural homology has led to the notion that either the lactone ring or the acyl side chain can be modified to provide potent AHL synthase inhibitors.^20,21^ Most AHLs are composed of an HSL core *N*-acylated with a fatty acyl chain at the α-position.^22^ As shown earlier, the amide linkage can be replaced by reverse amides or nitrogen-heterocycles (*e.g.*, 1,2,3-triazoles) to prepare biomimetic QS inhibitors.^23,24^ The designed thiolactone-triazole hybrids address key limitations of existing QSIs. Unlike conventional AHL analogs prone to hydrolysis, thiolactone improve the chemical stability and 1,2,3-triazoles provides conformational rigidity and tunable interactions with LasR receptor. Additionally, these scaffolds allow systematic modifications of substituents, enabling better SAR control and targeting the QS more effectively.

**Fig. 2:**
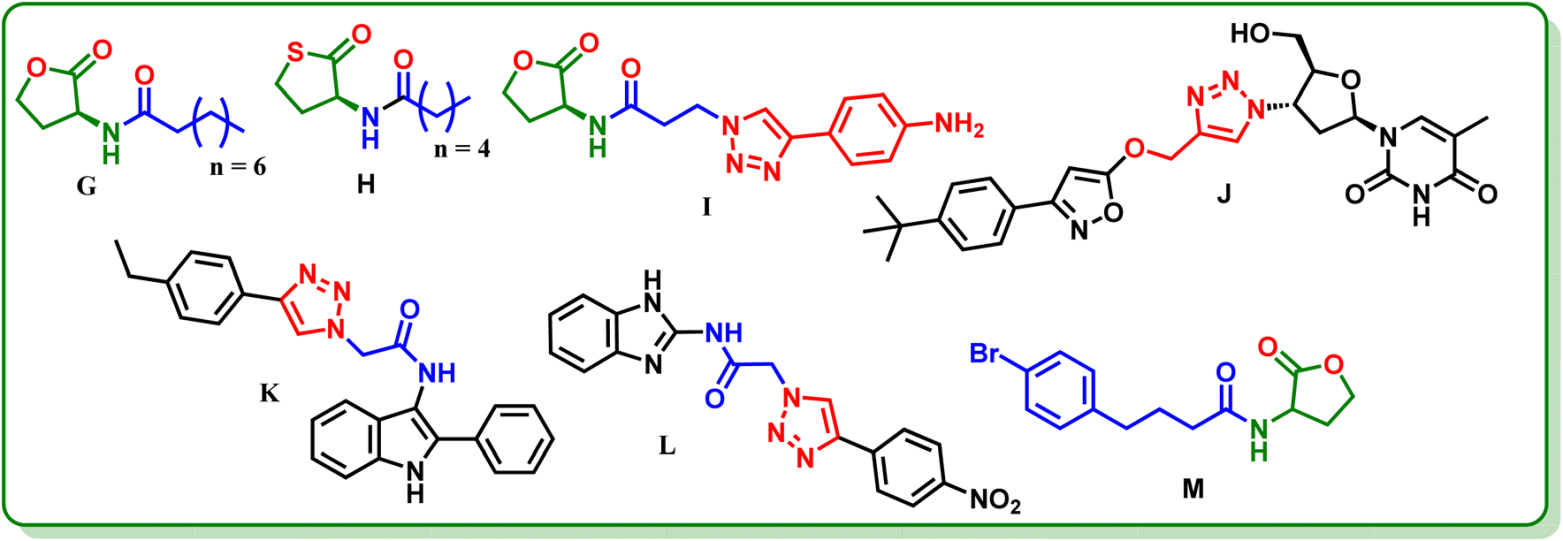
Reported synthetic QSIs.

Several research studies have pointed out the significance of such modifications. Geske *et al.* (2007) observed certain AHL analogues with potent QS inhibition (IC_50_: 0.25–0.61 µM), amongst which compound G exhibited the most potent activity with IC_50_ = 0.25 µM.^13^ McInnis *et al.* (2011) reported thiolactone analogues with potential LasR antagonistic activity, showing IC_50_ values as low as 0.14 µM for compound H.^25^ Stacy *et al.* (2013) showed that a methylene spacer between the *N*-acyl chain and triazole would favour QS inhibition exhibiting up to 77% inhibition for compound I.^26^ Zhang *et al.* (2017) reported 1,4-disubstituted triazoles and found compounds J, exhibited IC_50_ of 42.8 ± 4.5 µM.^27^ From our group, Srinivasarao *et al.*, synthesized 2-phenylindole and 2-amino benzimidazoles based triazole analogs and evaluated their QSI activity against the *P. aeruginosa* and found two promising molecules, compounds K and L (Fig. 2), with their percentage inhibition of 60.82% and 68.23% respectively.^28,29^ Wei and colleagues (2022) showed phenyl-substituted AHL analogues with moderate inhibition (up to 37%) for compound M.^30^

Collectively, these studies show that *N*-acetamide-containing thiolactones exhibit inhibitory activity. Molecular docking results of the current work suggest that the *N*-acetamide group is not a necessary component but rather a secondary amine linked to a lactone/thiolactone ring and hydrophobic interactions are required for activity (Section 2.1). These observations guided the design of novel glycinate and propanoate derivatives through molecular hybridization.

### 1.1 Design strategy

**Fig. 3:**
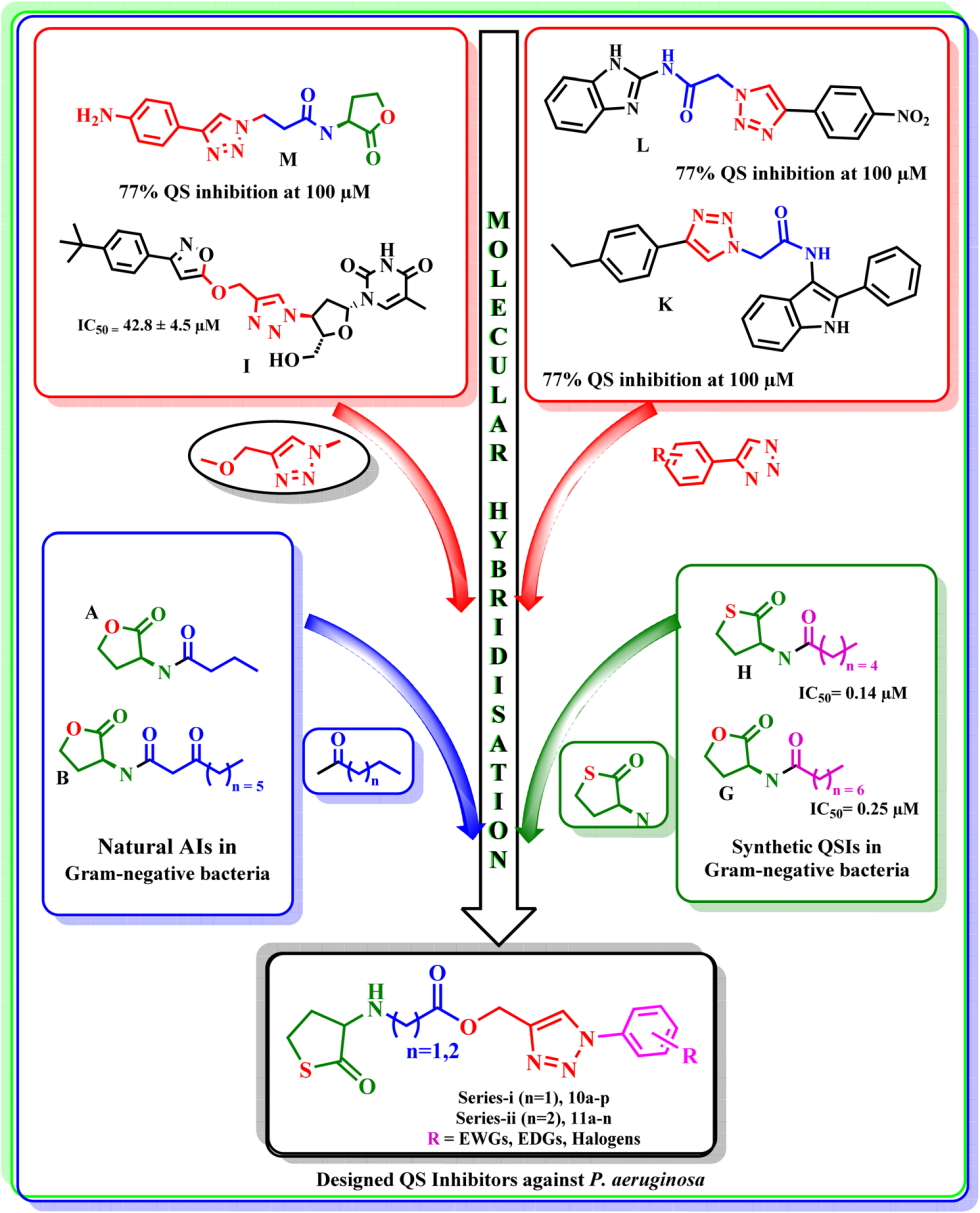
Design strategy for the synthesis of targeted molecules.

Our group has described 1,2,3-triazole-based, 2-phenylindole and 2-aminobenzimidazole derivatives as potent LasR inhibitors,^28,29^ but these scaffolds are non – AHL – mimetic and distinct from thiolactone – based AHL analogues. Hereby, we, with guided literature, report the design of novel AHL derivatives with thiolactone core linked to a 1,2,3-triazole by a flexible ester linker of varying lengths, and substituted aryl groups on triazole (Fig. 3). This is the first report of a thiolactone–triazole hybrid chemotype. The flexible linker allows optimal spatial arrangement within the binding site and may help with pharmacokinetics, while the triazole moiety, being an amide bioisostere, imparts rigidity, metabolic stability and facilitates H–bonding and π–π interactions. The aryl substitution imparts hydrophobic interactions and may improve pharmacokinetic properties. The designed compounds were subjected to molecular docking and binding free energy calculations, QS inhibition activity, molecular dynamics, and ADME profiling.

## 2 Results and discussion

### 2.1 Molecular docking and MM-GBSA study analysis

For carrying out molecular docking studies, the protein corresponding to *P. aeruginosa* LasR ligand-binding domain (PDB ID: 2UV0) was imported from protein databank and prepared using default parameters of the protein preparation wizard in the Schrödinger software. The forcefield applied was optimized potential for liquid simulations 2005 (OPLS_2005).^31^ The same force field was used to minimize all the designed ligands, and then they were subjected to a docking study using the Glide module of Schrödinger software, followed by MM-GBSA calculations to estimate binding free energies (Δ*G*_bind_).^32^ The complete methodology for carrying out both studies is described in the SI file (Section 5). The docking protocol was validated by re-docking the native ligand from the co-crystal structure. The final compounds were further analyzed for three parameters: (i) docking scores comparable to or better than the cocrystal/reference ligand(s); (ii) favorable MM-GBSA binding energies; and (iii) interaction pattern like the co-crystal/reference molecule-to assess their binding potential.

**Fig. 4:**
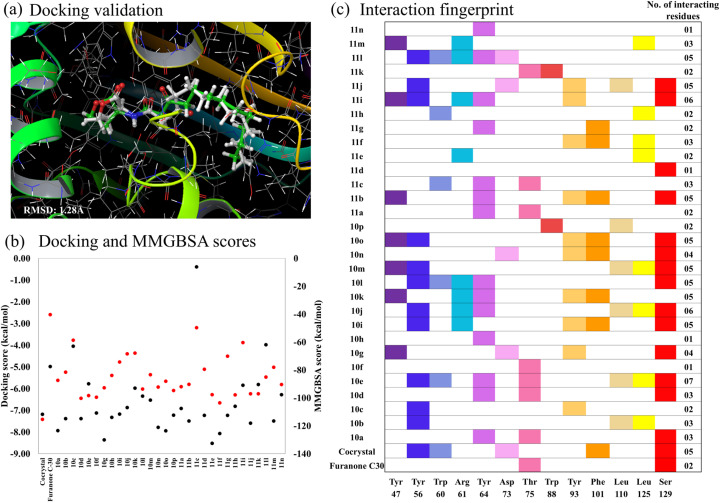
Molecular docking results representing (a) superimposition image of redocked pose on the native pose showing docking validation (b) docking scores (glide score, represented as black dots) and MM-GBSA score (represented as red dots) of the designed molecules; and (c) interaction fingerprint showing the active site residues interacting with the designed molecules.

Re-docking of the co-crystallized ligand reproduced the experimental binding mode (RMSD: 1.2809 Å) and yielded a Glide *g*score of −7.179 kcal mol^−1^ with an MM-GBSA Δ*G*_bind_ of −115.34 kcal mol^−1^, supporting the reliability of the docking protocol. The experimental reference compound (Furanone C-30) exhibited a docking score of −4.98 kcal mol^−1^ and an MM-GBSA score of −40.25 kcal mol^−1^ (Fig. 4a and b). The designed compounds exhibited a broad range of docking scores (−8.521 to −3.975) and MM-GBSA scores (−103 kcal mol^−1^ −58 kcal mol^−1^), with several candidates showing equal or improved docking performance relative to the native ligand. Across the series, most compounds demonstrated MM-GBSA binding energies comparable to those of the reference ligand, indicating consistent energetic stabilization within the binding pocket despite modest variations in docking rank. However, these values should be interpreted qualitatively rather than absolute binding free energy. Compounds 11e, 10g, 11f, and 10o achieved the most favorable docking scores (≤−7.9 kcal mol^−1^), while maintaining favorable MM-GBSA binding free energies (approximately −88 to −103 kcal mol^−1^), supporting their predicted binding stability. In contrast, compound 11c showed a substantially weaker docking score (−0.389 kcal mol^−1^) and unfavorable MM-GBSA binding energy (−49.59 kcal mol^−1^), consistent with poor predicted binding potential. We further compared the binding interactions between the ligands and the active site residues. The co-crystal ligand showed a total of five interactions, and similar number of interactions were observed with 10e, 10i–m, 10o, 11b, 11i, 11j and 11l, while the other molecules: 10a–d, 10f–h, 10n–p, 11a, 11c–h, 11k, 11m–n have shown relatively less interactions (Fig. 4c). Although variation in the number of interactions was observed, molecular docking and MM-GBSA results provide useful insights into binding modes and relative affinities, and do not capture fully the dynamic binding effects and entropic contributions arising from the chemical modifications. Therefore, these observations were used to support and rationalize for subsequent experimental validation rather than predict the activity. Accordingly, we synthesized all the molecules and evaluated them for their biological activity. From our designed molecules, due to poor binding affinity and fewer interactions, 11c was included as a negative control for subsequent experimental evaluation, while Furanone C-30 was considered as a positive control.^36^

### 2.2 Chemistry

**Scheme 1:**
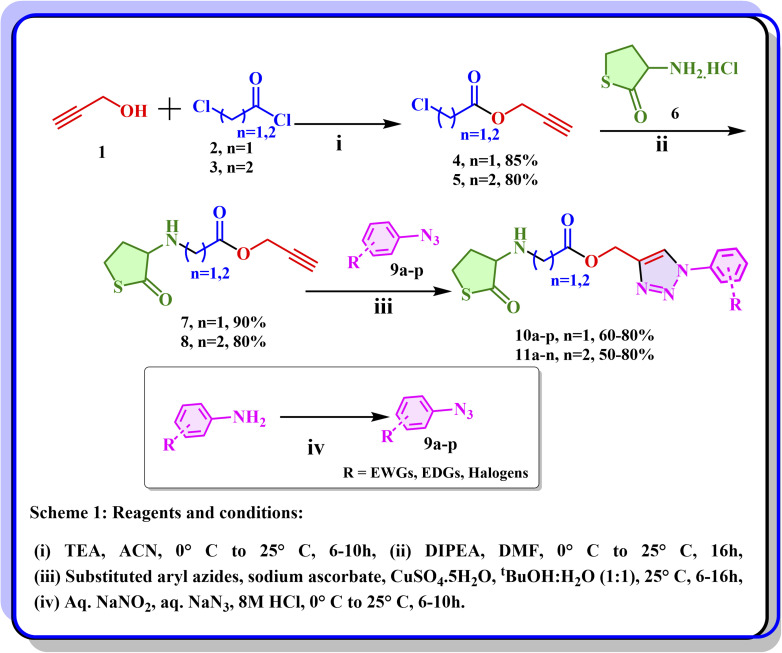
Synthetic scheme for the synthesis of titled compounds.

To a stirred solution of propargyl alcohol 1 in acetonitrile, TEA was added at 0 °C, followed by dropwise addition of compound 2 to obtain the corresponding ester 4. The product formation was confirmed by LC-MS and ^1^H NMR analysis. The chloro pattern was observed with the desired mass, and the appearance of a doublet around *δ* 4.72 ppm with 2H's corresponding to CH_2_ and a triplet at *δ* 2.46 ppm with ^1^H corresponding to the terminal alkyne indicated the formation of the desired product (compound 4). A similar method was used to obtain compound 5, where the reaction progress was monitored by TLC, while product formation was confirmed by LC-MS and ^1^H NMR analysis. The chloro pattern was observed with the desired mass, and the appearance of a doublet around *δ* 4.79 ppm with 2H's corresponding to CH_2_ of ester and a triplet at *δ* 2.52 ppm with ^1^H corresponding to the terminal alkyne indicated the formation of the desired product. Compounds 4 and 5 were reacted with compound 6*via* chloro displacement, yielding the corresponding compounds 7 and 8, as confirmed by mass and ^1^H NMR. The disappearance of the chloro pattern with the desired mass and the shift of *δ* 4.72 ppm and 4.79 ppm peaks to *δ* 4.67 ppm and 4.65 ppm, indicated the formation of desired compounds 7 and 8. They were reacted with various substituted aryl azides *via* Cu-catalyzed click reaction to obtain corresponding thiolactone-based 1,2,3-triazoles of series I (10a–p) and series II (11a–n), respectively. The final derivatives were monitored by LC-MS, and ^1^H NMR by the disappearance of peaks at *δ* 2.46 and 2.52 ppm, confirming the formation of desired products. We synthesized thirty final compounds in two series. The reaction's progress was monitored by TLC (thin-layer chromatography) and mass spectroscopy, and purification of compounds was done wherever necessary. All the synthesized compounds are subjected to ^1^H and ^13^C NMR, IR, and HRMS for their structural confirmation (Scheme 1).

### 2.3 LasR QS inhibition assay

Following our initial docking and MM-GBSA analyses, all designed molecules were synthesized and their LasR quorum sensing (QS) inhibition assay was performed to all the compounds of both the series: series I (10a–p) and series II (11a–n) against MH602 LasB reporter strain of *P. aeruginosa* MH602 [P_las_B: gfp (ASV)] at six different concentrations (8, 16, 32, 62.5, 125, and 250 µM) following the Hentzer *et al.* protocol.^33^ In an active QS system, this reporter strain generates AHL signals that causes an increase in unstable green fluorescent protein (GFPASV). The QS inhibition results of all the compounds are listed in Table 1. At 250 µM, most of the compounds showed significant activity, and at 125–8 µM, they showed moderate to low potency.

**Table 1:** Percentage QS inhibition on Las system of *P. aeruginosa* P_las_B: gfp (ASV) reporter strain using percentage of green fluorescent protein (GFP) fluorescence at 485 nm

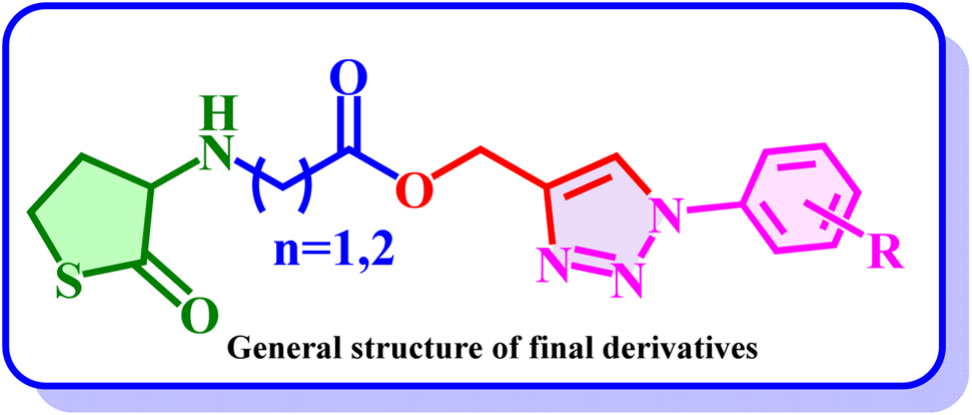								
Concentration (µM)^d^								
Code	R	*n*	250	125	62.5	32	16	8
10a	4-Ethyl	1	51.58±6.57^c^	26.16 ± 5.33^c^	18.23 ± 5.9^a^	20.29 ± 6.51^a^	6.57 ± 4.82^a^	NA
10b	2-Fluoro	1	62.69 ± 5.21^c^	50.37 ± 8.73^a^	26.4 ± 4.63^a^	10.02 ± 7.86^NA^	10.47 ± 7.1^NA^	NA
10c	3,4,5-Trimethoxy	1	70.23 ± 2.64^b^	42.19 ± 6.97^a^	39.71 ± 3.93^a^	36.72 ± 6.7^NA^	5.92 ± 6.51^NA^	NA
10d	4-Chloro	1	64.85 ± 4.46^c^	51.02 ± 3.64^a^	38.88 ± 7.13^a^	43.41 ± 5.16^NA^	17.00 ± 6.4^NA^	NA
10e	**3-(Trifluoromethyl)**	**1**	**74.55 ± 4.51^c^**	**45.13 ± 2.89^a^**	**31.3 ± 5.02^a^**	**38.3 ± 3.72** ^**NA**^	**13.19 ± 2.5** ^**NA**^	**8.74 ± 5.79** ^**NA**^
10f	4-Bromo	1	62.17 ± 1.16^b^	45.058 ± 7.7^a^	32.46 ± 3.38^a^	36.17 ± 2.94^NA^	15.59 ± 1.1^NA^	6.46 ± 3.59^NA^
10g	3,5-Dimethyl	1	56.05 ± 2.39^b^	38.99 ± 3.61^a^	28.26 ± 7.21^NA^	8.66 ± 2.7^NA^	12.7 ± 8.7^NA^	4.25 ± 4.53^NA^
10h	**2-Chloro**	**1**	**73.05 ± 3.36^c^**	**45.44 ± 7.96^a^**	**37.91 ± 7.03^a^**	**44.91 ± 5.93^a^**	**29.39 ± 5.7** ^**NA**^	**28.69 ± 6.85^a^**
10i	4-Methoxy-2-nitro	1	68.84 ± 0.79^c^	52.82 ± 7.72^c^	43.21 ± 5.76^c^	47.05 ± 8.35^b^	23.79 ± 8.7^b^	4.83 ± 5.89^b^
10j	4-Bromo-2-nitro	1	54.84 ± 1.72^b^	36.2 ± 4.07^a^	27.8 ± 6.84^a^	33.79 ± 4.12^NA^	12.07 ± 4.7^NA^	NA
10k	4-Nitro	1	63.37 ± 4.77^b^	43.1 ± 5.13^a^	35.27 ± 3.58^a^	34.26 ± 3.3^NA^	15.09 ± 1.5^NA^	5.81 ± 7.26^NA^
10l	2,4-Dichloro	1	53.11 ± 1.89^b^	30.04 ± 2.41^a^	33.61 ± 4.14^a^	33.94 ± 5.3^NA^	12.77 ± 2.3^NA^	NA
10m	2-Nitro	1	68.24 ± 6.09^b^	44.66 ± 8.14^a^	43.64 ± 2^a^	38.03 ± 8.32^NA^	14.07 ± 2.7^NA^	3.48 ± 6.12^NA^
10n	H	1	71.11 ± 7.9^c^	43.02 ± 4.9^a^	44.25 ± 6.38^a^	40.42 ± 6.6^NA^	15.93 ± 5.1^NA^	4.29 ± 0.79^NA^
10o	**3-Nitro**	**1**	**77.29 ± 2.77^b^**	**66.09 ± 6.66^a^**	**47.36 ± 7.24^a^**	**44.89 ± 2.03^a^**	**23.26 ± 5.2** ^**NA**^	**6.75 ± 2.71^b^**
10p	4-Hydroxy	1	74.8 ± 3.05^c^	45.92 ± 8.58^a^	38.02 ± 8.65^a^	42.29 ± 7.3^b^	28.75 ± 6.3^a^	25.72 ± 6.21^b^
11a	3,4-Dimethyl	2	67.66 ± 3.8^c^	5.17 ± 5.34^c^	14.6 ± 8.72^b^	15.42 ± 4.94^NA^	7.62 ± 7.2^b^	13.05 ± 6.65^NA^
11b	**3-Nitro**	**2**	**79.32 ± 5.48^c^**	**60.55 ± 5.04^b^**	**34.33 ± 4.46^b^**	**34.01 ± 5.47** ^**NA**^	**21.51 ± 5.5** ^**NA**^	**NA**
11c	3,4,5-Trimethoxy	2	34.75 ± 11.2^c^	6.12 ± 6.32^b^	NA	NA	1.69 ± 6.55^NA^	NA
11d	4-Chloro	2	65.02 ± 6.02^c^	44.83 ± 9.13^b^	22.04 ± 6.38^a^	30.31 ± 7.81^NA^	14.77 ± 3.8^NA^	NA
11e	3-(Trifluoromethyl)	2	71.11 ± 7.41^b^	56.89 ± 8.36^a^	28.52 ± 7.05^b^	31.88 ± 7.3^NA^	17.66 ± 4.7^NA^	14.83 ± 7.35^NA^
11f	3-Chloro	2	66.16 ± 6.33^c^	49.78 ± 5.79^c^	39.23 ± 5.82^a^	29.03 ± 6.48^NA^	16.82 ± 3.1^NA^	2.26 ± 3.86^NA^
11g	3,5-Dimethyl	2	63.24 ± 7.07^c^	46.06 ± 7.74^b^	35.63 ± 5.92^a^	25.52 ± 8.89^NA^	13.89 ± 6.5^NA^	9.59 ± 7.21^NA^
11h	2-Chloro	2	69.29 ± 16.5^c^	48.97 ± 6.79^b^	13.8 ± 8.98^b^	17.66 ± 5.23^a^	25.21 ± 4.7^a^	22.24 ± 7.65^b^
11i	4-Methoxy-2-nitro	2	65.78 ± 5.84^c^	42.84 ± 13.3^a^	25.34 ± 3.99^b^	29.13 ± 4.82^b^	39.59 ± 3.2^a^	19.78 ± 6.48^a^
11j	H	2	56.28 ± 5.01^c^	43.26 ± 6.71^b^	26.68 ± 1.88^b^	17.22 ± 7.17^a^	16.48 ± 7.4^b^	5.83 ± 3.23^a^
11k	**4-Nitro**	**2**	**75.58 ± 2.79^c^**	**63.41 ± 7.32^b^**	**50.3 ± 5.61^b^**	**43.16 ± 6.18^a^**	**26.68 ± 4.9^b^**	**7.32 ± 4.46** ^**NA**^
11l	**4-Bromo-2-nitro**	**2**	**72.07 ± 3.60^c^**	**59.55 ± 6.67^b^**	**43.02 ± 8.37^b^**	**42.07 ± 7.22** ^**NA**^	**17.66 ± 8.9^b^**	**10.92 ± 6.49** ^**NA**^
11m	2-Nitro	2	70.34 ± 6.35^c^	50.49 ± 5.54^b^	30.13 ± 8.26^b^	27.89 ± 5.26^NA^	17.31 ± 0.9^b^	4.49 ± 3.53^NA^
11n	2,4-Dichloro	2	71.23 ± 4.0^b^	62.07 ± 2.87^b^	45.59 ± 7.99^b^	44.14 ± 5.28^NA^	24.54 ± 3.0^b^	3.6 ± 9.88^NA^
Furanone C-30			—	86.86 ± 1.18	83.02 ± 3.15	77.33 ± 2.00	—	—

a Growth inhibition between 0–15%.

b Growth inhibition between 15–30%.

c Growth inhibition >30%, NA: no inhibition activity.

d All measurements were performed in triplicate with ± standard deviation from the mean.

### 2.4 Structure–activity relationship (SAR) trends

**Fig. 5:**
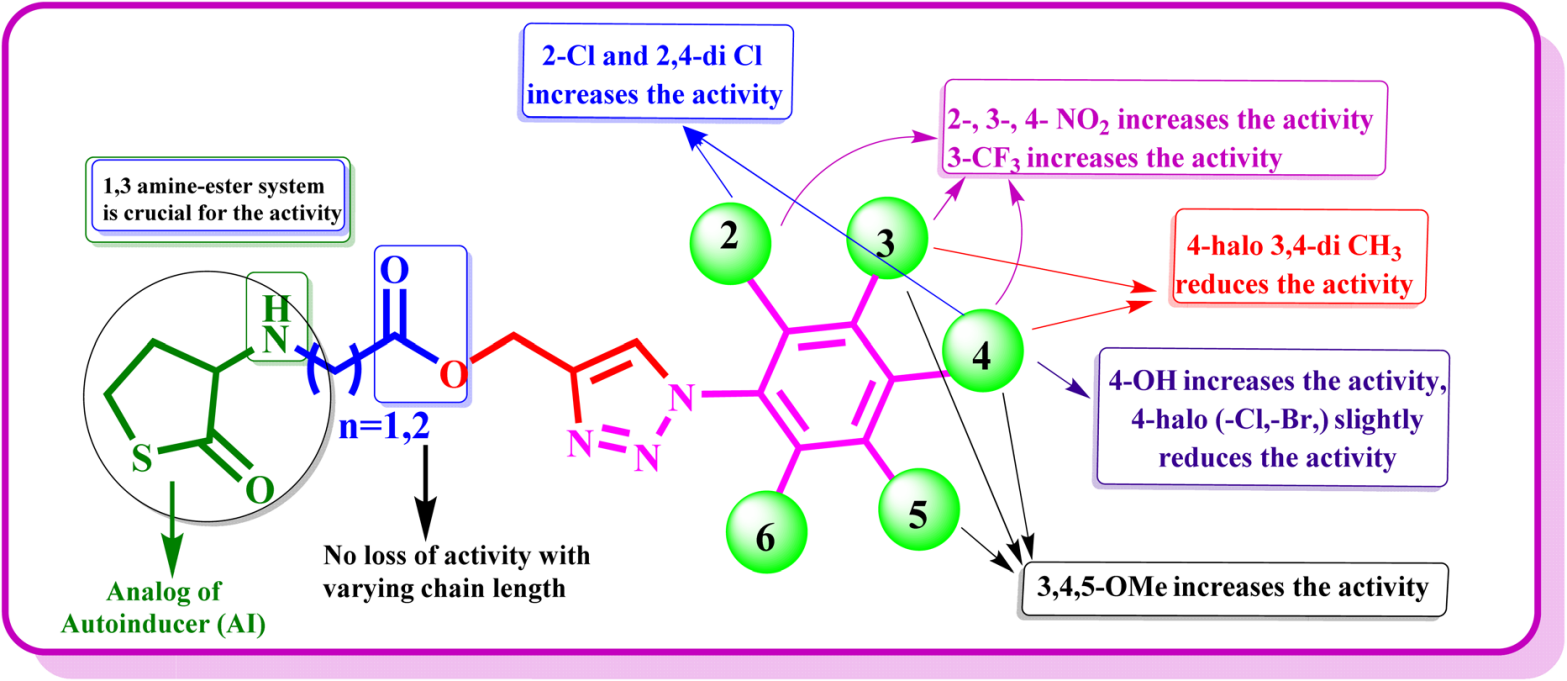
General SAR trends in inhibition of *P. aeruginosa* LasR.

We have designed and synthesized thiolactone-based AHL biomimics of natural AHLs and synthesized two series (10a–p and 11a–n) consisting of 30 novel 1,2,3-triazoles carrying core of glycinate (series I) or propanoate (series II). QSI activity of all novel 1,2,3-triazoles (10a–p and 11a–n) against *P. aeruginosa* MH602 strain was investigated and different modifications on phenyl ring were examined for structure–activity relationships (SAR) (Fig. 5). Several of the 1,2,3-triazole analogs displayed potent inhibition and may find consideration for further optimization. In general, the electron-withdrawing substituents help in increasing the activity and the propanoate-based analogs 11a–n exhibited moderately stronger inhibition than glycinate analogs 10a–p. For instance, CF_3_-substituted analogs 10e, 11e and strong electron-withdrawing substituents such as 4-bromo-2-nitro (10j, 11l), 4-nitro (10k, 11k), 4-bromo/3-chloro (10f, 11f), 2-chloro (10h, 11h), 2,4-dichloro (10l, 11n) and 2-nitro (10m, 11m) displayed this trend. Exceptions were 3-nitro (10o, 11b) and 4-chloro (10d, 11d). Electron donating substituents were more active in series I (trimethoxy: 10c, 11c). When the phenyl ring has increase in alkyl substitution/bulkiness was more active in series I (10a (4-ethyl) → 10g (3,5-dimethyl) → 10c (3,4,5-trimethoxy)) but had mixed results in series II (11g (3,5-dimethyl) is active, but 11a and 11c have low inhibition). While both electron-withdrawing and electron-donating substituents (10i, 11i) were moderately active and the unsubstituted analogs (10n, 11j) were equipotent. Fluoro and hydroxy derivatives (10b, 10p) were also moderately active. At 250 µM, most compounds were 65–80% inhibitory in both series. In series I, only 10a, 10g, 10j and 10l were <60%. In series II, all except 11c and 11j were in the 65–80% range. Compound 11c, which was considered as negative control from our docking studies have shown the least inhibition of 34.75% at 250 µM. Based on their QS inhibition profiles, potent compound 10o, 11b and 11k also showed favorable interaction pattern. These were selected along the reference compounds for molecular dynamics simulation to gain mechanistic insights indicating consistency between computational and experimental analyses.

### 2.5 Influence of predicted ADME properties on QS inhibition

To rationalize the observed biological activity and to access the drug-like properties of the designed compounds, absorption, distribution, metabolism and excretion (ADME) profiles were evaluated using SwissADME web-server (https://www.swissadme.ch/).^35^ Since QS targets are membrane associated (or) intracellular bacterial receptors, good membrane penetration is necessary for biological activity. *In silico* results indicated the designed molecules are small with molecular weights (MW) ranging between 350 and 470 Da demonstrating favorable size to show diffusion. Their lipophilicity (log *P*) values ranged from low to moderate with varying log *P* values (0.4 to 2.8), and total polar surface area (TPSA), which represents the sum of surface contributions from polar atoms, was between 110 and 166 Å^2^. All these findings obey Lipinski Rule of five (RO5) indicating acceptable drug-like properties (MW ≤ 500, hydrogen bond donor (HBD) ≤ 5, hydrogen bond acceptor (HBA) ≤ 10 and log *P* ≤ 5)^39^ (Table 2). In the case of QS inhibition, higher log *P* values indicate insolubility while the lower values indicate poor membrane penetration. An optimal log *P* values are desired for the compounds to penetrate the membrane. For instance, Furanone C-30, a known QS inhibitor, has log *P* value of 1.81, and its TPSA was found to be 26.3, as a result, it showed 77% inhibition even at 32 µM concentration which could be due to efficient membrane diffusion. On the other hand, the designed molecules exhibited comparatively higher TPSA values which may limit their ability to penetrate the bacterial membrane effectively. This trend is also observed in the biological activity (Table 1) in which most of the compounds showed moderate to good inhibition at higher concentration but reduced activity at lower concentrations. This lower activity at lower concentration can be due to limited intracellular accessibility, likely due to increased polarity and larger surface area. Furthermore, the designed molecules have no blood–brain barrier permeability which is advantageous for QS inhibitors as they are intended for peripheral bacterial infections and minimize potential CNS exposure. The molecules also showed a uniform bioavailability score of 0.55 and low to high GI absorption indicating acceptable oral drug-like properties and do not account for observed differences in biological activity. These observations also suggest that membrane permeability, rather than binding alone, plays an essential role in determining the QS inhibition of the designed molecules.

**Table 2:** *In Silico* predicted ADME and drug-likeness properties

Code	MW	Log *P*	HBD	HBA	TPSA	RB	G.I absorption	Synthetic accessibility	BBB	Bioavailability score
10a	360.43	2	1	6	111.41	8	High	3.51	No	0.55
10b	350.37	1.58	1	7	111.41	7	High	3.34	No	0.55
10c	422.46	1.29	1	9	139.1	10	High	3.87	No	0.55
10d	366.82	1.7	1	6	111.41	7	High	3.36	No	0.55
10e	400.38	2.23	1	9	111.41	8	High	3.49	No	0.55
10f	411.27	1.8	1	6	111.41	7	High	3.37	No	0.55
10g	360.43	1.87	1	6	111.41	7	High	3.53	No	0.55
10h	366.82	1.79	1	6	111.41	7	High	3.37	No	0.55
10i	407.4	0.58	1	9	166.46	9	Low	3.82	No	0.55
10j	456.27	1.08	1	8	157.23	8	Low	3.67	No	0.55
10k	377.38	0.44	1	8	157.23	8	Low	3.47	No	0.55
10l	401.27	2.22	1	6	111.41	7	High	3.42	No	0.55
10m	377.38	0.5	1	8	157.23	8	Low	3.62	No	0.55
10n	332.38	1.16	1	6	111.41	7	High	3.34	No	0.55
10o	377.38	0.53	1	8	157.23	8	Low	3.66	No	0.55
10p	348.38	0.75	2	7	131.64	7	High	3.36	No	0.55
11a	374.46	2.31	1	6	111.41	8	High	3.64	No	0.55
11b	391.4	0.9	1	8	157.23	9	Low	3.77	No	0.55
11c	436.48	1.66	1	9	139.1	11	High	3.98	No	0.55
11d	380.85	2.17	1	6	111.41	8	High	3.46	No	0.55
11e	414.4	2.76	1	9	111.41	9	High	3.61	No	0.55
11f	380.85	2.19	1	6	111.41	8	High	3.5	No	0.55
11g	374.46	2.23	1	6	111.41	8	High	3.64	No	0.55
11h	380.85	2.16	1	6	111.41	8	High	3.47	No	0.55
11i	421.43	0.91	1	9	166.46	10	Low	3.93	No	0.55
11j	346.4	1.55	1	6	111.41	8	High	3.44	No	0.55
11k	391.4	0.92	1	8	157.23	9	Low	3.57	No	0.55
11l	470.3	1.47	1	8	157.23	9	Low	3.78	No	0.55
11m	391.4	0.89	1	8	157.23	9	Low	3.72	No	0.55
11n	415.29	2.68	1	6	111.41	8	High	3.52	No	0.55
Furanone C30	253.88	1.81	0	2	26.3	0	High	3.03	Yes	0.55

### 2.6 Molecular dynamics studies

**Fig. 6:**
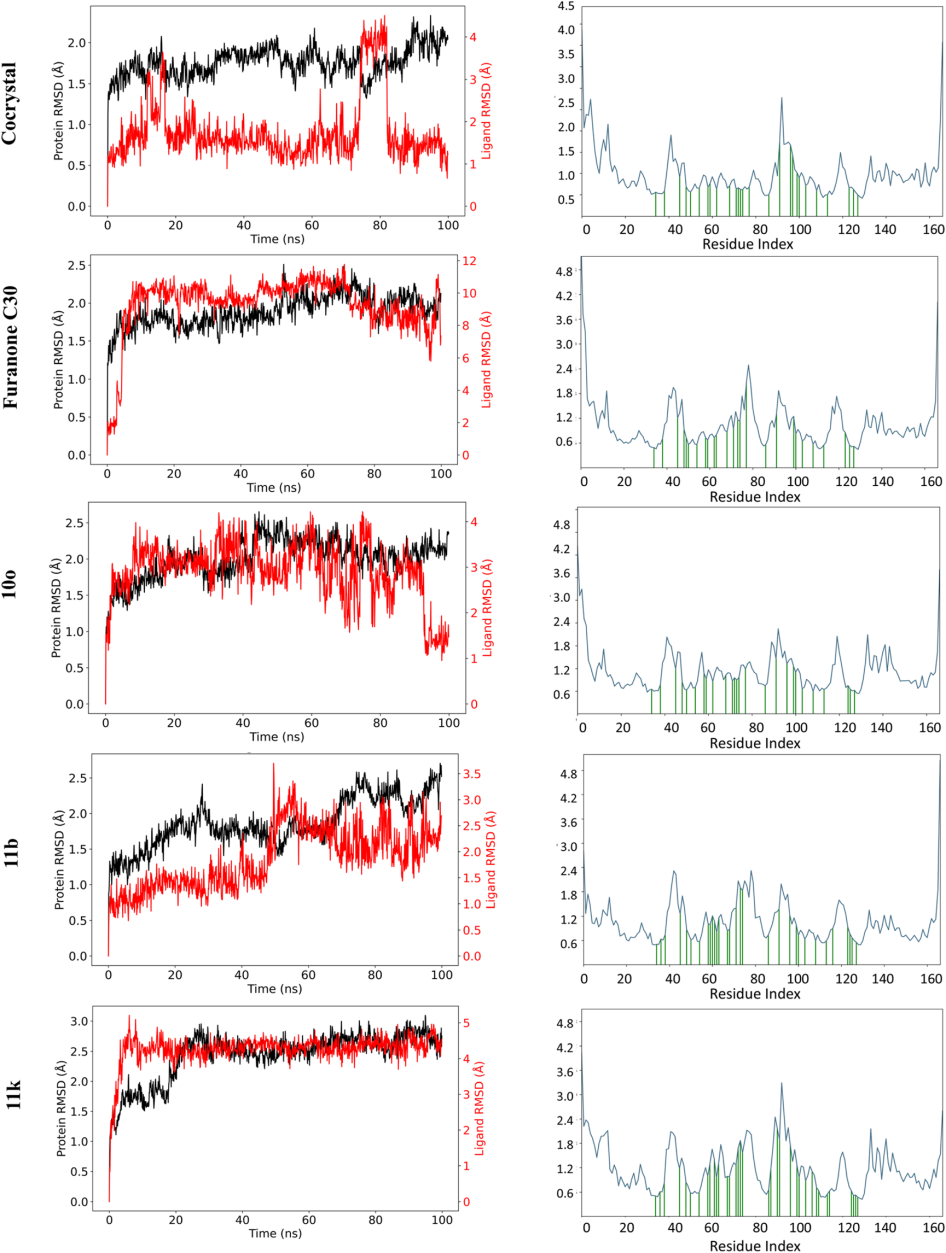
Molecular dynamics results representing the root mean square deviation (RMSD) plots and root mean square fluctuation (RMSF) plots of 10o, 11b, and 11k.

To gain mechanistic insights into the activity of the potent compounds identified from the QS inhibition as shown in Table 2, the top performing compounds (10o, 11b, 11k), reference controls (co-crystal and furanone) were subjected to molecular dynamics simulation to determine the stability of the complexes over 100 ns.^34^ The RMSD profiles of the protein in case of all the complexes show stable deviation ranging between 1.8 Å and 2.6 Å indicating no global structural destabilization (Fig. 6). The cocrystal shows the most stable ligand behavior, with ligand RMSD largely remaining below ∼1.5 Å, consistent with a well-anchored binding mode. In contrast, Furanone C30 displays an increasing RMSD for the initial 5 ns and remain stable until the end with deviations between 8–12 Å, indicative of significant ligand rearrangement initially. Compounds 10o, 11b, and 11k exhibit similar ligand behavior with RMSD remaining less than 4.0 Å. Upon analyzing the interaction pattern of the protein–ligand complexes (PLCs), moderate interactions were observed in the case of co-crystal with Trp60 (H-bond, 80%), Asp73 (H-bond, 60%) and Ser129 (H-bond, 38%) while the furanone showed two water-mediated H-bond interactions each with Tyr47 (45%) and Asp65 (44%). In the case of compound 10o, the polar atoms, such as the amino group, and the carbonyl group of the glycinate linker showed interactions with Asp73 (H-bond, 69%) and Try60 (H-bond, 78%) respectively, while the triazole moiety interacted with Arg61 (H-bond, 82%) and Tyr64 (π–π, 77%). Similarly, in 11b the amino, and the carbonyl group of the propanoate linker interacted with Asp73 (H-bond, 46%) and Try60 (H-bond, 60%). The triazole moiety interacted with Arg61 (H-bond, 34%) and Tyr64 (π–π, 33%). In the case of 11k, the carbonyl group of the propanoate linker interacted with Arg61 (water-mediated H-bond, 36%), and no interactions were observed with the amino group. However, the triazole showed two interactions with Trp60 (H-bond, 31%; π–π, 63%), and 4-nitro phenyl interacted with Trp88 (π–cation, 65%; π–π, 96%), Phe101 (π–π, 37%) and Phe102 (π–cation, 41%) (Fig. 6). This analysis revealed that the presence of polar functional groups (*e.g.* –NO_2_, –C=O) appears to facilitate H-bonding within the active site, which may contribute to enhanced QS inhibition, however, this is not observed in all molecules and depends on the overall structure of the molecule. Additionally, the selected most active compounds show consistent interactions with the residues Trp60, Asp73 and Arg61, which are also present in the co-crystal ligand. This suggests that the enhanced QS inhibition may be due to the ability of the compound to interact with these key residues under dynamic conditions.

### 2.7 Assessing antimicrobial and biofilm inhibition activity

**Fig. 7:**
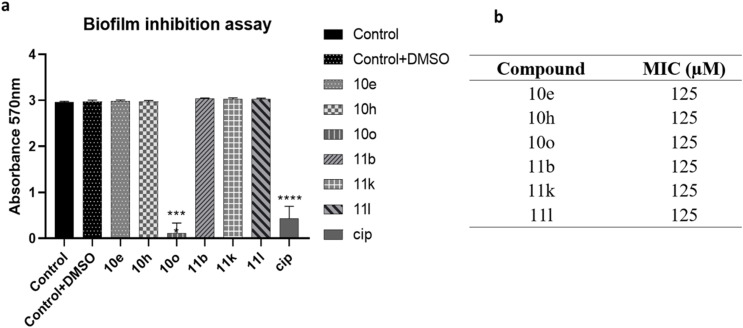
Biofilm inhibition assay for *P. aeruginosa*. (a) A graphical representation of the mean absorbance of crystal violet dye measured at 570 nm. The biofilm inhibition assay was performed with six technical replicates for each condition, and the error bar represents the standard deviation (SD). Two-way ANOVA was performed to compare the mean differences of each condition compared to the control, **** – *p* value < 0.0001. (b) The table corresponds to the concentration (sub-MIC) of each compound used for the biofilm inhibition assay.

**Fig. 8:**
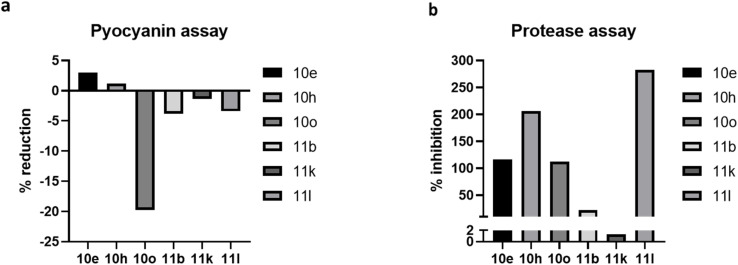
Pyocyanin and protease assay for *P. aeruginosa*. (a) A graphical representation of the percentage change of pyocyanin levels in samples treated with sub-MIC concentration of shortlisted compounds. The pyocyanin assay was performed with six technical replicates, and the graph presents the average percentage change with respect to the untreated control. (b) A graphical representation of the percentage of relative protease activity in samples treated at sub-MIC concentration of shortlisted compounds. The protease assay was performed with six technical replicates and represented graphically as the average percentage protease activity inhibition relative to the untreated control.

As outlined previously in this article, an optimal inhibitor should interfere with QS pathways without negatively impacting bacterial growth. The six lead compounds (10e, 10h, 10o, 11b, 11k, 11l), which demonstrated the greatest QSI, were advanced for further assessment of their antibacterial properties against *P. aeruginosa*. These candidates were evaluated at concentrations of 250, 125, 62.5, 32, 16, 8, 4, and 2 µM using the Microplate Alamar Blue Assay (MABA). The minimum inhibitory concentration (MIC) for each compound was determined visually by observing the change in color of the indicator dye, as described in the methods. Results indicate that none of the selected compounds exhibit antibacterial activity at 125 µM or lower. They exhibited mild growth inhibition only at the highest concentration tested (250 µM), while concentrations below this threshold had no significant impact on bacterial viability (Fig. S1, Section 3.3, in SI). Accordingly, a sub-MIC concentration of 125 µM was chosen for subsequent assays to evaluate QS inhibition, biofilm formation (Fig. 7), pyocyanin production, and protease activity (Fig. 8). While most analogues demonstrated moderate LasR inhibition (50–80%), this level of antagonism may be insufficient to disrupt downstream signaling cascades required for biofilm formation. In contrast, compound 10o likely achieves a higher functional level of pathway inhibition, crossing the threshold necessary to impact the interconnected QS circuitry and downstream virulence phenotypes. The apparent inconsistency arises from the fact that inhibition of LasR reporter activity does not necessarily translate directly into biofilm suppression, as biofilm formation in *P. aeruginosa* is regulated by a complex and hierarchical quorum sensing network involving Las, Rhl, and PQS systems.

From a structure–activity perspective, subtle differences in substituents may influence target engagement, cellular uptake, or membrane permeability, thereby affecting the effective intracellular concentration and functional activity of the compounds. Such factors could enable compound 10o to exert a more pronounced phenotypic effect, while other analogues remain limited to partial QS interference.

QS exerts significant control over various stages of biofilm development. Among the four major QS systems, the Las system specifically contributes to the repression of genes encoding extracellular polymeric substance (EPS) components, thereby modulating both biofilm formation and dispersion. We evaluated the impact of six selected compounds (10e, 10h, 10o, 11b, 11k, and 11l) at sub-minimal inhibitory concentration (sub-MIC) levels on biofilm formation. Notably, only compound 10o, which exhibited 66% QS inhibition at 125 µM, resulted in a significant reduction in biofilm formation, while the remaining compounds did not elicit comparable effects.

While most analogues demonstrated moderate LasR inhibition (50–80%), this level of antagonism may be insufficient to disrupt downstream signaling cascades required for biofilm formation. The apparent inconsistency arises from the fact that inhibition of LasR reporter activity does not necessarily translate directly into biofilm suppression, as biofilm formation in *P. aeruginosa* is regulated by a complex and hierarchical QS network involving Las, Rhl, and PQS systems. Thus, it is not surprising that certain compounds selectively or indirectly affect biofilm formation. In contrast, compound 10o likely achieves a higher functional level of pathway inhibition, crossing the threshold necessary to impact the interconnected QS circuitry and downstream virulence phenotypes. This demonstrates LasR-specific inhibition without translation to biofilm disruption, consistent with reports that LasR antagonists often fail to inhibit biofilm formation due to RhlR/PqsR redundancy. Our compounds exhibit circuit-specific inhibition rather than broad QS or biofilm suppression.^37^

From a structure–activity perspective, the distinct substituent pattern of compound 10o may enhance membrane partitioning or interaction with hydrophobic components of the biofilm matrix, contributing to its phenotypic selectivity. Such structural variations can influence effective cellular uptake and target engagement, enabling 10o to produce a more pronounced phenotypic response, whereas other analogues remain limited to partial QS interference.^37^

Since biofilm formation is governed by a broader network of QS-regulated and indirect pathways beyond LasR, this limitation accounts for the inconsistent anti-biofilm effects. These findings emphasize the complex interplay between QS and biofilm regulation, explaining the observed selectivity in biofilm inhibition.

Previous research has demonstrated that certain inhibitors targeting the Las QS system in *P. aeruginosa* also suppress the production of key virulence factors, including pyocyanin and proteases.^38^ These virulence determinants not only contribute to bacterial pathogenicity but are also essential for biofilm remodelling and dispersion processes Therefore, assessing these additional factors was important to determine whether the shortlisted compounds affect said virulence factors/pathways and QS mechanisms in addition to inhibiting biofilm *via* the Las system. Compound 10o showed reduction in pyocyanin levels along with biofilm inhibition, whereas in protease assays, it was observed that compounds 11b and 11k showed pronounced inhibition in protease activity (with no significant anti-biofilm activity).

Notably, 10o failed to inhibit protease activity despite exhibiting pronounced anti-biofilm activity and reduced pyocyanin levels (Fig. 8 and Table 3, respectively). The observed reduction in biofilm and pyocyanin, alongside unchanged protease activity, suggests that compound 10o may selectively target specific virulence pathways in *P. aeruginosa*, while leaving others unaffected. This pattern of selective inhibition aligns with previous findings, which reported that distinct interventions can differentially modulate virulence factors governed by separate genetic or regulatory systems. These compounds were rationally designed and synthesized to target LasR-mediated quorum sensing-driven biofilm formation. The study aims to evaluate their potential as QSIs to be used as adjuncts to conventional antibiotic therapies, thereby enhancing the efficacy of existing antibiotics and mitigating the emergence and spread of antimicrobial resistance. Moreover, combining these agents with established antibiotic classes has the potential to lower both the effective concentrations of QSI compounds below the tested 125 µM threshold and the required dosages of antibiotics themselves.^13^ Numerous studies have established that QSIs are active at micromolar concentrations, even those of modest potency, and can serve as valuable adjuncts to antibiotics. In such combinations, synergistic anti-biofilm and anti-virulence effects substantially improve therapeutic outcomes.

**Table 3:** The tabular column corresponds to the comparison of biofilm, pyocyanin, and protease assay results for shortlisted compounds^a^

Code	Biofilm inhibition	%Pyocyanin reduction	%Protease inhibition
10e	No (ns)	2.99	116.12
10h	No (ns)	1.17	206.45
10o	Yes (****)	−19.76	112.25
11b	No (ns)	−3.78	21.93
11k	No (ns)	−1.36	1.29
11l	No (ns)	−3.4	282.58

a In the biofilm inhibition column, ****, *p* value < 0.0001, ns-not significant, obtained from two-way ANOVA.

### 2.8 Cytotoxicity studies

The top four active compounds with percentage inhibition of 75–79% were evaluated for toxicity on normal human embryonic kidney cell line (HEK293T) at various doses ranging 5, 10, 20, 40, 80, 160, 320 µM. The resultant IC_50_ values ranged from 165 to 193 µM, indicating that the compounds are non-toxic to the normal cells. The cell proliferation was measured at an OD of 450 nm (Table 4).

**Table 4:** Results of cytotoxicity studies on the HEK293T cell line

Code	Entry	IC_50_^a^ (HEK293T) (µM)
1	10e	165 ± 2.4
2	10o	193.8 ± 2.2
3	11b	186.05 ± 1.55
4	11k	179.9 ± 2.05

a IC_50_ value is shown as mean ± SD. The assay was carried out in triplicate.

## 3 Conclusion

In conclusion, our study demonstrates the design and synthesis of two series of novel thiolactone-based glycinate and propanoate derivatives. The designed molecules were primarily subjected to pharmacokinetic parameter analysis, followed by molecular docking and free energy (MM-GBSA) calculations. The pharmacokinetic profile suggests that all the molecules possess favorable physicochemical characteristics, as they comply with the Lipinski rule of five. Furthermore, docking and MM-GBSA calculations revealed the desired interactions with the active site residues. To validate the activity of our designed molecules, we synthesized and characterized them using ^1^H and ^13^C-NMR, IR, and HRMS, and evaluated biologically against *P. aeruginosa* MH602 at six concentrations (250, 125, 62.5, 32, 16, and 8 µM). These compounds significantly inhibited QS (at 250 µM). Amongst the two series, compound 10o in Series I, and 11b in Series II, with a 3-nitrophenyl substituent on the triazole ring, exhibited the highest QSI activity of 77.2% and 79.3% at 250 µM, respectively. Most active compounds from each series were also evaluated for cytotoxicity studies against the HEK 293T cell line. The overall structure–activity relationship docking and dynamics analyses provides a mechanistic rationale for QS inhibition. Compound with electron-withdrawing and polar groups such as nitro and/or carbonyl groups showed enhanced activity which can be due to the ability of those molecules to show interactions with the residues Trp60, Asp73 and Arg61 which are also present with the co-crystal ligand. These interactions were also present consistently during the MD simulations resulting in stable binding conformations throughout the simulation period. Although our designed molecules do not exhibit high potency, our SAR analysis based on experimental data provides the structural features essential for designing newer glycinate and propanoate analogues. Overall, this study presents novel chemotypes for the future development of QSIs as innovative SAR-driven approach in combating antibiotic resistance.

## 4 Analytical data for the final compounds

### 4.1 (1-(4-Ethylphenyl)-1*H*-1,2,3-triazol-4-yl) methyl (2-oxotetrahydrothiophen-3-yl) glycinate (10a)

Brown gum; yield (60%). IR (*ν̄*, cm^−1^): 1741.26(C=O, ester), 1683.33(C=O, amide), 3369.3(–NH–, amine), 3095.14(C–H, aromatic), 1519.18(C=C, aromatic), 2934.42(C–H, alkane). ^1^H NMR (400 MHz, CDCl_3_) *δ* 7.97 (s, 1H), 7.55 (d, *J* = 8.3 Hz, 2H), 7.27 (d, *J* = 8.2 Hz, 2H), 5.28 (s, 2H), 3.53 (s, 2H), 3.42–3.40 (m, 1H), 3.17–3.20 (m, 2H), 2.68–2.61 (m, 2H), 2.54–2.44 (m, 1H), 1.98–1.91 (m, 1H), 1.20 (t, *J* = 7.6 Hz, 3H). ^13^C NMR (101 MHz, CDCl_3_) *δ* 207.43, 171.76, 145.46, 142.94, 134.69, 129.14, 122.26, 120.64, 66.26, 58.01, 48.57, 32.07, 28.46, 27.75, 15.43. HRMS: (*m*/*z*) of C_17_H_20_N_4_O_3_S calculated for [M + H]^+^ is 361.1334 and observed 361.1278.

### 4.2 (1-(2-Fluorophenyl)-1*H*-1,2,3-triazol-4-yl) methyl (2-oxotetrahydrothiophen-3-yl) glycinate (10b)

Brown gum; yield (71%). IR (*ν̄*, cm^−1^): 1741.52(C=O, ester), 1685.52(C=O, amide), 3402.71(–NH–, amine), 3068.02(C–H, aromatic), 1597.96(C=C, aromatic), 2947.4(C–H, alkane). ^1^H NMR (400 MHz, CDCl_3_) *δ* 8.09 (s, 1H), 7.89–7.84 (m, 1H), 7.41–7.36 (m, 1H), 7.26 (m, 2H), 5.31 (s, 2H), 3.54 (s, 2H), 3.44–3.42 (m, 1H), 3.23–3.13 (m, 2H), 2.51–2.49 (m, 1H), 2.01–1.89 (m, 1H). ^13^C NMR (101 MHz, CDCl_3_) *δ* 207.32, 171.64, 152.14, 142.78, 130.53, 130.46, 125.29, 124.94, 117.18, 116.98, 66.21, 57.85, 48.50, 32.01, 27.74. HRMS: (*m*/*z*) of C_15_H_15_FN_4_O_3_S calculated for [M + H]^+^ is 351.0927 and observed 351.0858.

### 4.3 (1-(3,4,5-Trimethoxyphenyl)-1*H*-1,2,3-triazol-4-yl) methyl (2-oxotetrahydrothiophen-3-yl) glycinate (10c)

Brown gum; yield (65%). IR (*ν̄*, cm^−1^): 1741.99(C=O, ester), 1685.85(C=O, amide), 3448.11(–NH–, amine), 3099.87(C–H, aromatic), 1598.69(C=C, aromatic), 2927.12(C–H, alkane). ^1^H NMR (400 MHz, CDCl_3_) *δ* 8.00 (s, 1H), 6.88 (s, 2H), 5.28 (s, 2H), 3.86 (s, 6H), 3.81 (s, 3H), 3.53 (s, 2H), 3.46–3.35 (m, 1H), 3.27–3.09 (m, 2H), 2.50–2.48 (m, 1H), 2.03–1.84 (m, 1H). ^13^C NMR (101 MHz, CDCl_3_) *δ* 207.49, 171.71, 143.37, 135.30, 134.81, 130.01, 121.76, 66.25, 57.91, 53.58, 48.52, 32.03, 27.77. HRMS: (*m*/*z*) of C_18_H_22_N_4_O_6_S calculated for [M + H]^+^ is 423.1338 and observed 423.1243.

### 4.4 (1-(4-Chlorophenyl)-1*H*-1,2,3-triazol-4-yl) methyl (2-oxotetrahydrothiophen-3-yl) glycinate (10d)

Brown gum; yield (68%). IR (*ν̄*, cm^−1^): 1741.56(C=O, ester), 1686.03(C=O, amide), 3399.3(–NH–, amine), 3075.31(C–H, aromatic), 1560.31(C=C, aromatic), 2926.04(C–H, alkane). ^1^H NMR (400 MHz, CDCl_3_) *δ* 8.01 (s, 1H), 7.62 (d, *J* = 8.9 Hz, 2H), 7.44 (d, *J* = 8.9 Hz, 2H), 5.28 (s, 2H), 3.53 (s, 2H), 3.43–3.40 (m, 1H), 3.23–3.14 (m, 2H), 2.50–2.45 (m, 1H), 2.02–1.89 (m, 1H). ^13^C NMR (101 MHz, CDCl_3_) *δ* 207.49, 171.71, 143.37, 135.30, 134.81, 130.01, 122.21, 121.76, 121.72, 66.25, 57.91, 48.52, 32.03, 27.77. HRMS: (*m*/*z*) of C_15_H_15_ClN_4_O_3_S calculated for [M + H]^+^ is 367.0631 and observed 367.0564.

### 4.5 (1-(3-(Trifluoromethyl) phenyl)-1*H*-1,2,3-triazol-4-yl) methyl (2-oxotetrahydrothiophen-3-yl) glycinate (10e)

Brown gum; yield (80%). IR (*ν̄*, cm^−1^): 1743.73(C=O, ester), 1687.65(C=O, amide), 3412.04(–NH–, amine), 3086.16(C–H, aromatic), 1560.04(C=C, aromatic), 2930.57(C–H, alkane). ^1^H NMR (400 MHz, CDCl_3_) *δ* 8.12 (s, 1H), 7.97 (s, 1H), 7.92–7.87 (m, 1H), 7.65–7.60 (m, 2H), 5.30 (s, 2H), 3.54 (s, 2H), 3.44–3.41 (m, 1H), 3.25–3.10 (m, 2H), 2.52–2.44 (m, 1H), 2.04–1.89 (m, 1H). ^13^C NMR (101 MHz, CDCl_3_) *δ* 207.54, 171.70, 143.63, 137.14, 130.65, 125.62, 125.58, 122.30, 117.55, 117.51, 117.46, 66.24, 57.87, 56.43, 48.49, 31.99, 27.77. HRMS: (*m*/*z*) of C_16_H_15_F_3_N_4_O_3_Scalculated for [M + H]^+^ is 401.0895 and observed 401.0887.

### 4.6 (1-(4-Bromophenyl)-1*H*-1,2,3-triazol-4-yl) methyl (2-oxotetrahydrothiophen-3-yl) glycinate (10f)

Brown gum; yield (75%). IR (*ν̄*, cm^−1^): 1734.31(C=O, ester), 1684.35(C=O, amide), 3408.78(–NH–, amine), 3084.45(C–H, aromatic), 1537.01(C=C, aromatic), 2936.33(C–H, alkane). ^1^H NMR (400 MHz, CDCl_3_) *δ* 8.03 (s, 1H), 7.57 (d, *J* = 2.9 Hz, 4H), 5.27 (s, 2H), 3.52 (s, 2H), 3.45–3.40 (m, 1H), 3.21–3.16 (m, 2H), 2.50–2.47 (m, 1H), 1.97–1.89 (m, 1H). ^13^C NMR (101 MHz, CDCl_3_) *δ* 207.54, 171.71, 143.39, 135.76, 132.96, 122.65, 122.16, 121.96, 66.25, 57.88, 48.52, 32.03, 27.79. HRMS: (*m*/*z*) of C_15_H_15_BrN_4_O_3_S calculated for [M + H]^+^ is 411.0126 and observed 411.0068.

### 4.7 (1-(3,5-Dimethylphenyl)-1*H*-1,2,3-triazol-4-yl) methyl (2-oxotetrahydrothiophen-3-yl) glycinate (10g)

Brown gum; yield (73%). IR (*ν̄*, cm^−1^): 1744.28(C=O, ester), 1682.57(C=O, amide), 3391.08(–NH–, amine), 3082.81(C–H, aromatic), 1597.61(C=C, aromatic), 2947.53(C–H, alkane). ^1^H NMR (400 MHz, CDCl_3_) *δ* 7.97 (s, 1H), 7.27 (s, 2H), 7.01 (s, 1H), 5.28 (s, 2H), 3.53 (s, 2H), 3.46–3.38 (m, 1H), 3.21–3.18 (m, 2H), 2.49 (m, 1H), 2.33 (s, 6H), 1.97–1.90 (m, 1H). ^13^C NMR (101 MHz, CDCl_3_) *δ* 207.43, 171.76, 145.46, 142.94, 134.69, 129.14, 122.27, 120.64, 66.26, 58.01, 48.57, 32.07, 27.75, 15.43. HRMS: (*m*/*z*) of C_17_H_20_N_4_O_3_S calculated for [M + H]^+^ is 361.1334 and observed 361.1241.

### 4.8 (1-(2-Chlorophenyl)-1*H*-1,2,3-triazol-4-yl) methyl (2-oxotetrahydrothiophen-3-yl) glycinate (10h)

Brown gum; yield (69%). IR (*ν̄*, cm^−1^): 1739.98(C=O, ester), 1685.31(C=O, amide), 3375.12(–NH–, amine), 3069.77(C–H, aromatic), 1590.14(C=C, aromatic), 2936.17(C–H, alkane) ^1^H NMR (400 MHz, CDCl_3_) *δ* 8.01 (s, 1H), 7.54–7.50 (m, 2H), 7.40–7.38 (m, 2H), 5.31 (s, 2H), 3.60 (s, 2H), 3.47–3.31 (m, 1H), 3.29–3.07 (m, 2H), 2.53–2.46 (m, 1H), 2.05–1.85 (m, 1H). ^13^C NMR (101 MHz, CDCl_3_) *δ* 207.37, 171.67, 142.19, 134.87, 130.99, 128.59, 128.01, 126.08, 123.88, 66.21, 57.88, 48.51, 32.01, 27.75. HRMS: (*m*/*z*) of C_15_H_15_ClN_4_O_3_S calculated for [M + H]^+^ is 367.0631 and observed 367.0540.

### 4.9 (1-(4-Methoxy-2-nitrophenyl)-1*H*-1,2,3-triazol-4-yl) methyl (2-oxotetrahydrothiophen-3-yl) glycinate (10i)

Brown gum; yield (66%). IR (*ν̄*, cm^−1^): 1741.71(C=O, ester), 1681.87(C=O, amide), 3377.35(–NH–, amine), 3083.65(C–H, aromatic), 1580.05(C=C, aromatic), 2941.72(C–H, alkane). ^1^H NMR (400 MHz, CDCl_3_) *δ* 7.85 (s, 1H), 7.52 (d, *J* = 2.8 Hz, 1H), 7.44 (d, *J* = 8.8 Hz, 1H), 7.21 (d, *J* = 1.5 Hz, 1H), 5.29 (s, 2H), 3.89 (d, *J* = 2.5 Hz, 3H), 3.54 (s, 2H), 3.47–3.34 (m, 2H), 3.19–3.14 (m, 2H), 2.52–2.46 (m, 1H), 1.97–1.90 (m, 1H). ^13^C NMR (101 MHz, CDCl_3_) *δ* 207.51, 171.77, 161.01, 145.19, 142.71, 129.46, 126.27, 122.81, 119.33, 110.70, 65.84, 57.78, 48.57, 32.06, 27.74. HRMS: (*m*/*z*) of C_16_H_17_N_5_O_6_S calculated for [M + H]^+^ is 408.0978 and observed 408.0949.

### 4.10 (1-(4-Bromo-2-nitrophenyl)-1*H*-1,2,3-triazol-4-yl) methyl (2-oxotetrahydrothiophen-3-yl) glycinate (10j)

Brown gum; yield (70%). IR (*ν̄*, cm^−1^): 1742.45(C=O, ester), 1685.77(C=O, amide), 3374.79(–NH–, amine), 3098.03(C–H, aromatic), 1599.06(C=C, aromatic), 2936.6 (C–H, alkane). ^1^H NMR (400 MHz, CDCl_3_) *δ* 8.16 (d, *J* = 2.1 Hz, 1H), 7.91 (s, 1H), 7.86 (m, 1H), 7.48–7.44 (m, 1H), 5.29 (s, 2H), 3.53 (s, 2H), 3.41–3.39 (m, 1H), 3.22–3.18 (m, 2H), 2.51–2.48 (m, 1H), 1.96–1.89 (m, 1H). ^13^C NMR (101 MHz, CDCl_3_) *δ* 207.51, 171.77, 161.01, 145.19, 142.71, 129.46, 126.27, 122.81, 119.33, 110.70, 65.84, 57.78, 48.57, 32.06, 27.74. HRMS: (*m*/*z*) of C_15_H_14_BrN_5_O_5_S calculated for [M + H]^+^ is 455.9977 and observed 455.9900.

### 4.11 (1-(4-Nitrophenyl)-1*H*-1,2,3-triazol-4-yl) methyl (2-oxotetrahydrothiophen-3-yl) glycinate (10k)

Brown gum; yield (75%). IR (*ν̄*, cm^−1^): 1741.66(C=O, ester), 1685.49(C=O, amide), 3316.73(–NH–, amine), 3076.25(C–H, aromatic), 1596.9(C=C, aromatic), 2936.44(C–H, alkane). ^1^H NMR (400 MHz, CDCl_3_) *δ* 8.35 (d, *J* = 9.2 Hz, 2H), 8.19 (s, 1H), 7.93 (d, *J* = 9.2 Hz, 2H), 5.31 (s, 2H), 3.54 (s, 2H), 3.47–3.36 (m, 1H), 3.31–3.13 (m, 2H), 2.50–2.47 (m, 1H), 2.00–1.91 (m, 1H). ^13^C NMR (101 MHz, CDCl_3_) *δ* 207.73, 171.75, 147.37, 144.09, 140.96, 125.59, 122.25, 120.63, 66.29, 57.77, 48.53, 32.07, 29.30. HRMS: (*m*/*z*) of C_15_H_15_N_5_O_5_S calculated for [M + H]^+^ is 378.0872 and observed 378.0814.

### 4.12 (1-(2,4-Dichlorophenyl)-1*H*-1,2,3-triazol-4-yl) methyl (2-oxotetrahydrothiophen-3-yl) glycinate (10l)

Brown gum; yield (75%). IR (*ν̄*, cm^−1^): 1744.03(C=O, ester), 1691.17(C=O, amide), 3391.51(–NH–, amine), 3085.7(C–H, aromatic), 1561.6(C=C, aromatic), 2946.02(C–H, alkane). ^1^H NMR (400 MHz, CDCl_3_) *δ* 8.00 (s, 1H), 7.54 (d, *J* = 2.2 Hz, 1H), 7.49 (s, 1H), 7.37 (dd, *J* = 8.6, 2.3 Hz, 1H), 5.31 (s, 2H), 3.54 (s, 2H), 3.42–3.35 (m, 1H), 3.20–3.17 (m, 2H), 2.53–2.43 (m, 1H), 2.04–1.88 (m, 1H). ^13^C NMR (101 MHz, CDCl_3_) *δ* 207.57, 171.75, 144.48, 143.24, 137.03, 129.14, 128.72, 125.72, 124.44, 119.39, 66.24, 57.64, 48.56, 32.06, 27.75. HRMS: (*m*/*z*) of C_15_H_14_C_12_N_4_O_3_S calculated for [M + H]^+^ is 401.0242 and observed 401.0172.

### 4.13 (1-(2-Nitrophenyl)-1*H*-1,2,3-triazol-4-yl) methyl (2-oxotetrahydrothiophen-3-yl) glycinate (10m)

Brown gum; yield (68%). IR (*ν̄*, cm^−1^): 1742.23(C=O, ester), 1686.69(C=O, amide), 3392.51(–NH–, amine), 3083.35(C–H, aromatic), 1587.96(C=C, aromatic), 2947.11(C–H, alkane). ^1^H NMR (400 MHz, CDCl_3_) *δ* 8.03 (dd, *J* = 8.1, 1.4 Hz, 1H), 7.91 (s, 1H), 7.75 (td, *J* = 7.7, 1.5 Hz, 1H), 7.68–7.64 (m, 1H), 7.56 (dd, *J* = 7.9, 1.4 Hz, 1H), 5.30 (s, 2H), 3.53 (s, 2H), 3.45–3.36 (m, 1H), 3.21–3.19 (m, 2H), 2.49–2.46 (m, 1H), 2.00–1.87 (m, 1H). ^13^C NMR (101 MHz, CDCl_3_) *δ* 207.53, 171.75, 144.40, 143.00, 134.01, 131.07, 130.03, 128.05, 125.81, 125.69, 120.81, 66.22, 57.72, 48.55, 32.05, 27.75. HRMS: (*m*/*z*) of C_15_H_15_N_5_O_5_S calculated for [M + H]^+^ is 378.0872 and observed 378.0867.

### 4.14 (1-Phenyl-1*H*-1,2,3-triazol-4-yl) methyl (2-oxotetrahydrothiophen-3-yl) glycinate (10n)

Brown gum; yield (62%). IR (*ν̄*, cm^−1^): 1739.46(C=O, ester), 1684.13(C=O, amide), 3352.19(–NH–, amine), 3084.36(C–H, aromatic), 1596.69(C=C, aromatic), 2926.87(C–H, alkane). ^1^H NMR (400 MHz, CDCl_3_) *δ* 8.02 (s, 1H), 7.68–7.65 (m, 2H), 7.48–7.43 (m, 2H), 7.38–7.35 (2, 1H), 5.29 (s, 2H), 3.54 (s, 2H), 3.45–3.38 (m, 1H), 3.24–3.14 (m, 2H), 2.53–2.46 (m, 1H), 2.00–1.89 (m, 1H). ^13^C NMR (101 MHz, CDCl_3_) *δ* 207.36, 171.65, 136.82, 129.83, 128.97, 122.30, 120.63, 66.22, 58.00, 48.50, 31.97, 27.75. HRMS: (*m*/*z*) of C_15_H_16_N_4_O_3_S calculated for [M + H]^+^ is 333.1021 and observed 333.0938.

### 4.15 (1-(3-Nitrophenyl)-1*H*-1,2,3-triazol-4-yl) methyl (2-oxotetrahydrothiophen-3-yl) glycinate (10o)

Brown gum; yield (75%). IR (*ν̄*, cm^−1^): 1739.99(C=O, ester), 1687.57(C=O, amide), 3389.07(–NH–, amine), 3099.85(C–H, aromatic), 1531.26(C=C, aromatic), 2969.36(C–H, alkane). ^1^H NMR (400 MHz, CDCl_3_) *δ* 8.56–8.54 (m, 1H), 8.26–8.23 (m, 1H), 8.19 (s, 1H), 8.14–8.10 (m, 1H), 7.71–7.68 (m, 1H), 5.32 (s, 2H), 3.55 (s, 2H), 3.45–3.35 (m, 1H), 3.25–3.13 (m, 2H), 2.53–2.46 (m, 1H), 1.97–1.90 (m, 1H). ^13^C NMR (101 MHz, CDCl_3_) *δ* 207.61, 171.73, 148.96, 143.96, 137.54, 131.07, 125.98, 123.43, 122.19, 66.28, 57.83, 48.52, 32.04, 27.10. HRMS: (*m*/*z*) of C_15_H_15_N_5_O_5_S calculated for [M + H]^+^ is 378.0872 and observed 378.0808.

### 4.16 (1-(4-Hydroxyphenyl)-1*H*-1,2,3-triazol-4-yl) methyl (2-oxotetrahydrothiophen-3-yl) glycinate (10p)

Brown gum; yield (72%). IR (*ν̄*, cm^−1^): 1731.3(C=O, ester), 3294.98(–NH–, amine), 1553.18(C=C, aromatic), 2955.14(C–H, alkane). ^1^H NMR (400 MHz, CDCl_3_) *δ* 7.89 (s, 1H), 7.45 (d, *J* = 8.9 Hz, 2H), 6.90 (d, *J* = 8.9 Hz, 2H), 5.28 (s, 2H), 3.54 (s, 2H), 3.44–3.40 (m, 1H), 3.23–3.17 (m, 2H), 2.54–2.45 (m, 1H), 1.98–1.89 (m, 1H). ^13^C NMR (101 MHz, CDCl_3_) *δ* 207.54, 171.68, 157.20, 142.73, 129.75, 122.48, 120.12, 116.47, 66.26, 57.96, 48.50, 31.94, 27.76. HRMS: (*m*/*z*) of C_15_H_16_N_4_O_4_S calculated for [M + H]^+^ is 349.097 and observed 349.0906.

### 4.17 (1-(3,4-Dimethylphenyl)-1*H*-1,2,3-triazol-4-yl) methyl 3-((2-oxotetrahydrothiophen-3-yl) amino) propanoate (11a)

Brown gum; yield (75%). IR (*ν̄*, cm^−1^): 1726.37(C=O, ester), 1672.92(C=O, amide), 3376.58(–NH–, amine), 3052.83(C–H, aromatic), 1591.18(C=C, aromatic), 2922.87(C–H, alkane). ^1^H NMR (400 MHz, CDCl_3_) *δ* 8.02 (s, 1H), 7.52–7.43 (m, 1H), 7.37 (dd, *J* = 8.3, 2.1 Hz, 1H), 7.17 (dd, *J* = 8.4, 1.1 Hz, 1H), 5.25 (s, 2H), 3.46–3.44 (m, 1H), 3.02–2.92 (m, 2H), 2.94–2.82 (m, 2H), 2.41 (s, 3H), 2.39 (s, 3H), 2.12–2.10 (m, 1H), 2.10–1.99 (m, 2H), 1.99–1.94 (m, 1H). ^13^C NMR (101 MHz, CDCl_3_) *δ* 207.93, 172.26, 145.96, 143.44, 135.19, 129.64, 122.77, 121.14, 66.76, 58.51, 49.07, 32.57, 28.97, 28.25, 15.93. HRMS: (*m*/*z*) of C_18_H_22_N_4_O_3_S calculated for [M + H]^+^ is 375.1491 and observed 375.1472.

### 4.18 (1-(3-Nitrophenyl)-1*H*-1,2,3-triazol-4-yl) methyl 3-((2-oxotetrahydrothiophen-3-yl) amino) propanoate (11b)

Brown gum; yield (78%). IR (*ν̄*, cm^−1^): 1729.53(C=O, ester), 1672.97(C=O, amide), 3405.22(–NH–, amine), 3054.25(C–H, aromatic), 1529.27(C=C, aromatic), 2931.54(C–H, alkane). ^1^H NMR (400 MHz, CDCl_3_) *δ* 8.12 (s, 1H), 8.08–8.05 (m, 1H), 8.02 (s, 1H), 8.00–7.98 (m, 1H), 7.45 (t, *J* = 8.2 Hz, 1H), 5.29 (s, 2H), 3.52–3.48 (m, 1H), 3.15–3.04 (m, 2H), 3.04–2.90 (m, 2H), 2.16–1.89 (m, 4H). ^13^C NMR (101 MHz, CDCl_3_) *δ* 208.11, 172.23, 149.46, 144.46, 138.04, 131.57, 126.48, 123.93, 122.69, 66.78, 58.33, 49.02, 32.54, 28.29, 27.60. HRMS: (*m*/*z*) of C_16_H_17_N_5_O_5_S calculated for [M + H]^+^ is 392.1028 and observed 392.1055.

### 4.19 (1-(3,4,5-Trimethoxyphenyl)-1*H*-1,2,3-triazol-4-yl) methyl 3-((2-oxotetrahydrothiophen-3-yl) amino) propanoate (11c)

Brown gum; yield (50%). IR (*ν̄*, cm^−1^): 1733.51(C=O, ester), 1666.81(C=O, amide), 3427.52(–NH–, amine), 1602.94(C=C, aromatic), 2939.92(C–H, alkane). ^1^H NMR (400 MHz, CDCl_3_) *δ* 8.05 (s, 1H), 6.86 (s, 2H), 5.29 (s, 2H), 3.85 (s, 6H), 3.84 (s, 3H), 3.45 (dt, *J* = 6.5, 3.4 Hz, 1H), 3.04–3.02 (m, 2H), 2.99–2.97 (m, 2H), 2.19–1.96 (m, 4H). ^13^C NMR (101 MHz, CDCl_3_) *δ* 207.99, 172.21, 143.87, 135.80, 135.31, 130.51, 122.26, 66.75, 58.41, 49.02, 32.53, 30.19, 28.27. HRMS: (*m*/*z*) of C_19_H_24_N_4_O_6_S calculated for [M + H]^+^ is 437.1495 and observed 437.1502.

### 4.20 (1-(4-Chlorophenyl)-1*H*-1,2,3-triazol-4-yl) methyl 3-((2-oxotetrahydrothiophen-3-yl) amino) propanoate (11d)

Brown gum; yield (52%). IR (*ν̄*, cm^−1^): 1725.28(C=O, ester), 1672.33(C=O, amide), 3413.89(–NH–, amine), 3052.12(C–H, aromatic), 1501.94(C=C, aromatic), 2926.28(C–H, alkane). ^1^H NMR (400 MHz, CDCl_3_) *δ* 8.05 (s, 1H), 7.57 (d, *J* = 8.4 Hz, 2H), 7.35 (d, *J* = 8.4 Hz, 2H), 5.25 (s, 2H), 3.49–3.47 (m, 1H), 3.0–3.02 (m, 2H), 2.99–2.95 (m, 2H), 2.15–1.92 (m, 4H). ^13^C NMR (101 MHz, CDCl_3_) *δ* 207.99, 172.21, 143.87, 135.80, 135.31, 130.51, 122.71, 122.26, 122.22, 66.75, 58.41, 58.33, 54.08, 49.02, 32.53, 28.27. HRMS: (*m*/*z*) of C_16_H_17_ClN_4_O_3_S calculated for [M + H]^+^ is 381.0788 and observed 381.0810.

### 4.21 (1-(3-(Trifluoromethyl) phenyl)-1*H*-1,2,3-triazol-4-yl) methyl 3((2oxotetrahydrothiophen-3-yl) amino) propanoate (11e)

Brown gum; yield (65%). IR (*ν̄*, cm^−1^): 1726.53(C=O, ester), 1674.25(C=O, amide), 3391.68(–NH–, amine), 3052.91(C–H, aromatic), 1600.26(C=C, aromatic), 2929.08(C–H, alkane). ^1^H NMR (400 MHz, CDCl_3_) *δ* 8.03 (s, 1H), 7.76 (t, *J* = 2.1 Hz, 1H), 7.74–7.71 (m, 1H), 7.67–7.65 (m, 1H), 7.41–7.37 (m, 1H), 5.29 (s, 2H), 3.52–3.49 (m, 1H), 3.10–2.97 (m, 2H), 2.98–2.87 (m, 2H), 2.11–1.80 (m, 4H). ^13^C NMR (101 MHz, CDCl_3_) *δ* 208.04, 172.20, 144.13, 137.64, 131.15, 126.12, 126.08, 122.80, 118.05, 118.01, 117.96, 66.74, 58.37, 48.99, 32.49, 30.17, 28.27. HRMS: (*m*/*z*) of C_17_H_17_F_3_N_4_O_3_S calculated for [M + H]^+^ is 415.1051 and observed 415.1083.

### 4.22 (1-(3-Chlorophenyl)-1*H*-1,2,3-triazol-4-yl) methyl 3-((2-oxotetrahydrothiophen-3-yl) amino) propanoate (11f)

Brown gum; yield (57%). IR (*ν̄*, cm^−1^): 1730.77(C=O, ester), 1674.2(C=O, amide), 3428.26(–NH–, amine), 3092.03(C–H, aromatic), 1597.05(C=C, aromatic), 2959.69(C–H, alkane). ^1^H NMR (400 MHz, CDCl_3_) *δ* 8.04 (s, 1H), 7.78 (s, 1H), 7.66–7.64 (m, 1H), 7.44–7.33 (m, 1H), 7.29 (t, *J* = 8.0 Hz, 2H), 5.29 (s, 2H), 3.95–3.39 (m, 2H), 3.05–3.02 (m, 2H), 3.00–2.94 (m, 2H), 2.08–2.03 (m, 4H). ^13^C NMR (101 MHz, CDCl_3_) *δ* 207.87, 172.17, 142.69, 135.37, 131.49, 129.09, 128.51, 126.58, 124.38, 66.71, 58.38, 49.01, 32.51, 29.86, 28.25. HRMS: (*m*/*z*) of C_16_H_17_ClN_4_O_3_S calculated for [M + H]^+^ is 381.0788 and observed 381.3027.

### 4.23 (1-(3,5-Dimethylphenyl)-1*H*-1,2,3-triazol-4-yl) methyl 3-((2-oxotetrahydrothiophen-3-yl) amino) propanoate (11g)

Brown gum; yield (68%). IR (*ν̄*, cm^−1^): 1730.89(C=O, ester), 1674.03(C=O, amide), 3434.88(–NH–, amine), 3096.34(C–H, aromatic), 1597.83(C=C, aromatic), 2921.93(C–H, alkane). ^1^H NMR (400 MHz, CDCl_3_) *δ* 8.04 (s, 1H), 7.27 (s, 2H), 6.99 (s, 1H), 5.25 (s, 2H), 3.50–3.46 (m, 1H), 3.12–3.00 (m, 2H), 2.98–2.96 (m, 2H), 2.32 (s, 6H), 2.21–1.83 (m, 4H). ^13^C NMR (101 MHz, CDCl_3_) *δ* 207.93, 172.26, 145.96, 143.44, 135.19, 129.64, 122.77, 121.14, 66.76, 58.51, 49.07, 32.57, 28.97, 28.25, 15.93. HRMS: (*m*/*z*) of C_18_H_22_N_4_O_3_S calculated for [M + H]^+^ is 375.1491 and observed 375.1497.

### 4.24 (1-(2-Chlorophenyl)-1*H*-1,2,3-triazol-4-yl) methyl 3-((2-oxotetrahydrothiophen-3-yl) amino) propanoate (11h)

Brown gum; yield (78%). IR (*ν̄*, cm^−1^): 1733.39(C=O, ester), 1668.9(C=O, amide), 3402.32(–NH–, amine), 3086.7(C–H, aromatic), 1603.79(C=C, aromatic), 2931.71(C–H, alkane). ^1^H NMR (400 MHz, CDCl_3_) *δ* 8.15 (s, 1H), 7.63–7.48 (m, 3H), 7.30–7.28 (m, 1H), 5.28 (s, 2H), 3.49–3.46 (m, 1H), 3.05–3.03 (m, 2H), 2.96–2.93 (m, 2H), 2.08–1.92 (m, 4H). ^13^C NMR (101 MHz, CDCl_3_) *δ* 207.87, 172.17, 142.69, 135.37, 131.49, 129.09, 128.51, 126.58, 124.38, 66.71, 58.38, 49.01, 32.51, 29.86, 28.25. HRMS: (*m*/*z*) of C_16_H_17_ClN_4_O_3_S calculated for [M + H]^+^ is 381.0788 and observed 381.0839.

### 4.25 (1-(4-Methoxy-2-nitrophenyl)-1*H*-1,2,3-triazol-4-yl) methyl 3-((2-oxotetrahydrothiophen-3-yl) amino) propanoate (11i)

Brown gum; yield (75%). IR (*ν̄*, cm^−1^): 1732.75(C=O, ester), 1667.43(C=O, amide), 3415.76(–NH–, amine), 3082.14(C–H, aromatic), 1606.71(C=C, aromatic), 2928.56(C–H, alkane). ^1^H NMR (400 MHz, CDCl_3_) *δ* 7.96 (s, 1H), 7.94 (d, *J* = 8.6 Hz, 1H), 7.69 (d, *J* = 2.1 Hz, 1H), 7.57 (dd, *J* = 8.6, 2.2 Hz, 1H), 5.28 (s, 2H), 3.85 (s, 3H), 3.59–3.56 (m, 1H), 3.09–2.92 (m, 4H), 2.08–1.94 (m, 4H). ^13^C NMR (101 MHz, CDCl_3_) *δ* 208.01, 172.27, 161.51, 145.69, 143.21, 129.96, 126.77, 123.31, 119.83, 111.20, 66.34, 58.28, 56.94, 49.07, 32.56, 30.18, 28.24. HRMS: (*m*/*z*) of C_17_H_19_N_5_O_6_S calculated for [M + H]^+^ is 422.1134 and observed 422.1186.

### 4.26 (1-Phenyl-1*H*-1,2,3-triazol-4-yl) methyl 3-((2-oxotetrahydrothiophen-3-yl) amino) propanoate (11j)

Brown gum; yield (55%). IR (*ν̄*, cm^−1^): 1732.99(C=O, ester), 1672.57(C=O, amide), 3484.65(–NH–, amine), 3064.36(C–H, aromatic), 1596.08(C=C, aromatic), 2962.15(C–H, alkane). ^1^H NMR (400 MHz, CDCl_3_) *δ* 8.05 (s, 1H), 7.69–7.56 (m, 2H), 7.56–7.43 (m, 2H), 7.42–7.28 (m, 1H), 5.25 (s, 2H), 3.58–3.56 (m, 1H), 3.23–2.82 (m, 4H), 2.11–1.81 (m, 4H). ^13^C NMR (101 MHz, CDCl_3_) *δ* 207.86, 172.15, 137.32, 130.33, 129.47, 122.80, 121.13, 66.72, 58.50, 49.00, 32.47, 30.19, 28.25. HRMS: (*m*/*z*) of C_16_H_18_N_4_O_3_S calculated for [M + H]^+^ is 347.1178 and observed 347.1241.

### 4.27 (1-(4-Nitrophenyl)-1*H*-1,2,3-triazol-4-yl) methyl 3-((2-oxotetrahydrothiophen-3-yl) amino) propanoate (11k)

Brown gum; yield (65%). IR (*ν̄*, cm^−1^): 1731.95(C=O, ester), 1675.3(C=O, amide), 3432.77(–NH–, amine), 3081.89 (C–H, aromatic), 1593.29(C=C, aromatic), 2956.84(C–H, alkane). ^1^H NMR (400 MHz, CDCl_3_) *δ* 8.43 (d, *J* = 9.7 Hz, 2H), 8.03 (s, 1H), 7.99 (d, *J* = 9.7 Hz, 2H), 5.28 (s, 2H), 3.50–3.47 (m, 1H), 3.17–3.00 (m, 2H), 2.98–2.94 (m, 2H), 2.17–1.92 (m, 4H). ^13^C NMR (101 MHz, CDCl_3_) *δ* 208.23, 172.25, 147.87, 144.59, 141.46, 126.09, 122.75, 121.13, 66.79, 58.27, 49.03, 32.57, 29.80, 28.32. HRMS: (*m*/*z*) of C_16_H_17_N_5_O_5_S calculated for [M + H]^+^ is 392.1028 and observed 392.0692.

### 4.28 (1-(4-Bromo-2-nitrophenyl)-1*H*-1,2,3-triazol-4-yl) methyl 3-((2-oxotetrahydrothiophen-3-yl) amino) propanoate (11l)

Brown gum; yield (60%). IR (*ν̄*, cm^−1^): 1733.3(C=O, ester), 1667.63(C=O, amide), 3401.09 (–NH–, amine), 3090.05 (C–H, aromatic), 1604.25(C=C, aromatic), 2961.43(C–H, alkane). ^1^H NMR (400 MHz, CDCl_3_) *δ* 8.32 (d, *J* = 2.2 Hz, 1H), 8.06 (s, 1H), 7.94 (dd, *J* = 8.6, 2.2 Hz, 1H), 7.65 (d, *J* = 8.6 Hz, 1H), 5.28 (s, 2H), 3.68–3.65 (m, 1H), 3.15–3.06 (m, 2H), 3.09–2.93 (m, 2H), 2.26–1.80 (m, 4H). ^13^C NMR (101 MHz, CDCl_3_) *δ* 207.88, 172.25, 145.68, 144.98, 137.53, 129.96, 129.21, 126.22, 124.94, 119.89, 66.74, 58.28, 49.06, 32.56, 29.82, 28.25. HRMS: (*m*/*z*) of C_16_H_16_BrN_5_O_5_S calculated for [M + H]^+^ is 470.0134 and observed 470.0178.

### 4.29 (1-(2-Nitrophenyl)-1*H*-1,2,3-triazol-4-yl) methyl 3-((2-oxotetrahydrothiophen-3-yl) amino) propanoate (11m)

Brown gum; yield (68%). IR (*ν̄*, cm^−1^): 1732.87(C=O, ester), 1667.36(C=O, amide), 3386.95(–NH–, amine), 3085.1(C–H, aromatic), 1605.76(C=C, aromatic), 2965.01(C–H, alkane). ^1^H NMR (400 MHz, CDCl_3_) *δ* 8.13–8.10 (m, 2H), 8.05 (s, 1H), 7.75–7.54 (m, 2H), 5.29 (s, 2H), 3.48–3.45 (m, 1H), 3.24–2.95 (m, 4H), 2.15–1.81 (m, 4H). ^13^C NMR (101 MHz, CDCl_3_) *δ* 208.03, 172.25, 144.90, 143.50, 134.51, 131.57, 130.53, 128.55, 126.31, 126.19, 121.31, 66.72, 58.22, 49.05, 32.55, 29.84, 28.25. HRMS: (*m*/*z*) of C_16_H_17_N_5_O_5_S calculated for [M + H]^+^ is 392.1028 and observed 392.1076.

### 4.30 (1-(2,4-Dichlorophenyl)-1*H*-1,2,3-triazol-4-yl) methyl 3-((2-oxotetrahydrothiophen-3-yl) amino) propanoate (11n)

Brown gum; yield (75%). IR (*ν̄*, cm^−1^): 1731.85(C=O, ester), 1671.48(C=O, amide), 3373.43(–NH–, amine), 3083.78(C–H, aromatic), 1565.13(C=C, aromatic), 2927.39(C–H, alkane). ^1^H NMR (400 MHz, CDCl_3_) *δ* 8.20 (s, 1H), 7.58 (d, *J* = 2.2 Hz, 1H), 7.38 (d, *J* = 8.6 Hz, 1H), 7.18 (dd, *J* = 8.7, 2.1 Hz, 1H), 5.28 (s, 2H), 3.51–3.48 (m, 1H), 3.15–3.05 (m, 2H), 3.06–2.93 (m, 2H), 2.26–1.84 (m, 4H). ^13^C NMR (101 MHz, CDCl_3_) *δ* 208.07, 172.25, 144.98, 143.74, 137.53, 129.64, 129.22, 126.22, 124.94, 119.89, 66.74, 58.14, 49.06, 32.56, 29.82, 28.25. HRMS: (*m*/*z*) of C_16_H_16_C_l2_N_4_O_3_S calculated for [M + H]^+^ is 415.0398 and observed 415.044.

## Author contributions

Kosana Sai Chaitanya: chemistry conceptualization, data curation, methodology, writing original draft – reviewing and editing, formal analysis, validation. Tsz Tin Yu: QS inhibition methodology, data curation. Hrushikesh Chaudhari and Pranali Vijaykumar Kuthe: docking analysis and validation. Nidhi Orekonday: microbial assays methodology, data curation. Naresh Kumar: QS inhibition assays conceptualization, writing – reviewing and editing. Ruchi Jain Dey: microbial assays conceptualization, writing – reviewing and editing. Sankaranarayanan Murugesan: project administration, reviewing and editing. Kondapalli Venkata Gowri Chandra Sekhar: supervision, resources, project administration, chemistry conceptualization, writing – reviewing and editing, funding acquisition.

## Conflicts of interest

The authors declare that they have no known competing financial interests or personal relationships that could have appeared to influence the work reported in this paper.

## Supplementary Material

RA-016-D6RA00717A-s001

## Data Availability

Experimental protocols for the synthesis of intermediates and final derivatives, along with the spectroscopic/analytical data such as ^1^H NMR, ^13^C NMR, IR, and HRMS spectra, are provided in the supplementary information (SI). Detailed experimental procedures carried out for biological studies and molecular docking studies are also provided. Supplementary information is available. See DOI: https://doi.org/10.1039/d6ra00717a.
