# Supplementary material for: Discovery of triazole-tethered glycinate and propanoate derivatives bearing a thiolactone moiety as quorum sensing inhibitors of Pseudomonas aeruginosa: design, synthesis, biological evaluation, and biofilm inhibition
Source: RSC Adv. 2026 May 22;16(30):27977–93. doi: 10.1039/d6ra00717a (PMC13202525; doi:10.1039/d6ra00717a)
Supplement: RA-016-D6RA00717A-s001 [file RA-016-D6RA00717A-s001.pdf]

Supplementary Information (SI) for RSC Advances.
This journal is © The Royal Society of Chemistry 2026

Discovery of Triazole-Tethered Glycinate and Propanoate Derivatives Bearing a Thiolactone Moiety as Quorum Sensing Inhibitors of *Pseudomonas aeruginosa*: Design, Synthesis, Biological Evaluation, and Biofilm Inhibition

Kosana Sai Chaitanya[a], Tsz Tin Yu[b], Hrushikesh Chaudhari[a], Nidhi Orenkonday[c], Pranali Vijaykumar Kuthe[d], Naresh Kumar[b], Ruchi Jain Dey[c], Sankaranarayanan Murugesan[d], Kondapalli Venkata Gowri Chandra Sekhar[a*]

[a] *Department of Chemistry, Birla Institute of Technology and Science, Pilani, Hyderabad Campus, Jawahar Nagar, Hyderabad – 500 078, Telangana, India.*

[b] *School of Chemistry, The University of New South Wales, Sydney, NSW 2052, Australia.*

[c] *Department of Biological Sciences, Birla Institute of Technology and Science, Pilani, Hyderabad Campus, Jawahar Nagar, Hyderabad – 500 078, Telangana, India.*

[d] *Department of Pharmacy, Birla Institute of Technology and Science, Pilani Campus, Vidya Vihar, Pilani– 333031, Rajasthan, India.*

[*] Corresponding author
Tel: +91 40 66303527; E-mail: kvgc@hyderabad.bits-pilani.ac.in; kvgcs.bits@gmail.com

Contents: **Page no**

1.	Materials and methods	S3
2.	General procedure	S3-S5
3.	Biological Procedures	S6-S9
4.	In Silico Predicted ADME	S9
5.	Molecular docking studies	S10-S12
6.	Molecular Dynamics simulation (MDS) Studies	S12
7.	^{1}HNMR Spectra	S13-S29
8.	^{13}C NMR Spectra	S30-S44
9.	HRMS-spectra	S45-S54
10.	IR spectrum of	S55-S69
11.	References	S70

1. Materials and methods

Experimental

1.1 *Chemistry*

All chemical reagents and solvents are purchased from Aldrich, Alfa Aesar, Finar, TCI, BLD pharma. The solvents and reagents were of LR grade for the reactions and commercial grade for the column purification. Thin-layer chromatography (TLC) was carried out on aluminum-supportedsilica gel plates (Merck 60 F254) with visualization of components by UV light (254 nm). Column chromatography was carried out on silica gel (Merck 100-200 mesh). ^{1}H NMR and ^{13}C NMR spectra were recorded at 400 MHz and 101 MHz, respectively, using a Bruker AV 400 spectrometer (Bruker CO.) in $CDCl_3$ solution with tetramethyl silane as the internal standard, and chemical shift values (δ) were given in ppm. ^{1}H NMR spectra were recorded in $CDCl_3$. Melting points were determined on an electro thermal melting point apparatus (Stuart-SMP30) in open capillary tubes. HRMS and Mass spectra (ESI-MS) were recorded on Schimadzu HRMS and MS/ESI mass spectrometer respectively.

2. General Procedure and Spectral Data

2.1 Synthesis of *Prop-2-yn-1-yl 2-chloroacetate* (4): A stirred solution of propargyl alcohol (1.0 mmol) **1** in Acetonitrile was cooled to 0°C and added TEA (1.5 mmol) and stirred the solution at 0° C for 10-15 min. 2-chloroacetyl chloride **2** (1.5 mmol) was added slowly dropwise to the reaction mixture, maintaining the internal temperature less than 5° C. After the completion of addition, the resultant reaction mixture was allowed to come to room temperature and stirred for 6 hrs, monitored by TLC. After the completion of reaction as indicated in TLC, formation of new spot, and confirmed the product mass was in LCMS. The reaction mixture was evaporated on rotavapor. The crude compound was cooled to 0°C and slowly neutralized by adding Sat.$NaHCO_3$ solution and extracted thrice with EtOAc and the combined organic layer was given water washes three times followed by brine wash. The organic layer was dried over sodium sulphate, filtered and concentrated. The crude residue obtained was purified by column chromatography using hexane and ethyl acetate (0-15%,) as the eluents and concentrated and dried to obtain **4** as brown gum. The appearance of a doublet around δ 4.72 ppm with 2H's corresponding to CH_2 and a triplet at δ 2.46 ppm with 1H corresponding to the terminal alkyne indicated the formation of the desired product. ESI-MS: (*m/z*) calcd for $[M+H]^+$ for $C_5H_5ClO_2$: 131.9978 and found 131.9981. ^{1}H NMR (400 MHz, $CDCl_3$) δ 4.72 (d, J = 2.4 Hz, 2H), 4.08 (s, 2H), 2.46 (t, J = 2.5 Hz, 1H).

2.2 Synthesis of *Prop-2-yn-1-yl 3-chloropropanoate* (5): A stirred solution of propargyl alcohol **1** (1.0 mmol) in Acetonitrile was cooled to 0°C, and TEA (1.5 mmol) was added. The solution was stirred at 0 °C for 10-15 min. 3-chloropropanoyl chloride **3** (1.5 mmol) was added slowly dropwise to the reaction mixture, maintaining the internal temperature less than 5° C. After the completion of addition, the resultant reaction mixture was allowed to come to room temperature and stirred for 6 hrs, monitored by TLC. After the completion of reaction as indicated in TLC, formation of new spot, and confirmed the product mass was in LCMS. The reaction mixture was evaporated on rotavapor. The crude compound was cooled to 0°C and slowly neutralized by adding sat. $NaHCO_3$ solution and extracted thrice with EtOAc and the combined organic layer was given water washes three times followed by brine wash. The organic layer was dried over sodium sulphate, filtered and concentrated. The crude residue obtained was purified by column chromatography using hexane and ethyl acetate (0-15%,) as the eluents and concentrated and dried to obtain **5** as brown gum. The appearance of a doublet around δ 4.79 ppm with 2H's corresponding to CH_2 of ester and a triplet at δ 2.52 ppm with 1H corresponding to the terminal alkyne indicated the formation of the desired product. ESI-MS: (*m/z*) calcd for $[M+H]^+$ for $C_6H_7ClO_2$: 146.0135 and found 146.0128. ^{1}H NMR (400 MHz, $CDCl_3$) δ 4.79 (d, *J* = 2.5 Hz, 2H), 3.67 – 3.60 (m, 2H), 2.52 (t, *J* = 2.5 Hz, 1H), 2.51 – 2.46 (m, 2H).

2.3 Synthesis of *Prop-2-yn-1-yl (2-oxotetrahydrothiophen-3-yl) glycinate* (7): A stirred solution of 3-aminodihydrothiophen-2(3H)-one hydrochloride **6** (1.0 mmol) in DMF was stirred at room temperature until a clear solution was obtained, then cooled to 0° C. DIPEA (3.0 mmol) was added slowly at 0°C and stirred for 15-20 min. To this reaction mixture, compound **4** (1.5 mmol) was added, and the resultant mixture was allowed to stir at room temperature overnight. The progress of the reaction is monitored by TLC. After the completion of reaction as indicated in TLC, formation of new spot, and confirmed the product mass was in LCMS, sat. $NaHCO_3$ solution was added to the reaction mixture and extracted with 10% MeOH in $CHCl_3$ three times. The combined organic layer was given water washes three times followed by brine wash. The organic layer was dried over sodium sulphate, filtered and concentrated. The crude residue obtained was purified by column chromatography using hexane-ethyl acetate (0-100%) followed by MeOH-$CHCl_3$ (0-6%) as the eluents and concentrated under the vacuum and dried to obtain **7** as a brown gum, the shift of δ 5.66 ppm peak to δ 4.67 ppm indicated the formation of the desired product **7**. ESI-MS: (*m/z*) calcd for $[M+H]^+$ for $C_9H_{11}NO_3S$: 214.0460 and found 214.0490. ^{1}H NMR (400 MHz, $CDCl_3$) δ 4.67 (d, *J* = 2.5 Hz, 2H), 3.55 (s, 2H), 3.43 (m, 1H), 3.25 – 3.17 (m, 2H), 2.56 – 2.47 (m, 1H), 2.45 – 2.44 (m, 1H), 2.46 – 2.42 (m, 1H), 2.12 (m, 1H), 2.02 – 1.89 (m, 1H).

2.4 Synthesis of *Prop-2-yn-1-yl 3-((2-oxotetrahydrothiophen-3-yl) amino) propanoate* (8): A stirred solution of 3-aminodihydrothiophen-2(3H)-one hydrochloride **6** (1.0 mmol) in DMF was stirred at room temperature until the clear solution is obtained then cooled to 0° C. DIPEA (3.0 mmol) was added slowly at 0°C and stirred for 15-20 min. To this reaction mixture compound **5** (1.5 mmol) was added and the resultant mixture was allowed to stir at room temperature overnight. The progress of the reaction is monitored by TLC. After the completion of reaction as indicated in TLC, formation of new spot, and confirmed the product mass was in LCMS, sat. $NaHCO_3$ solution was added to the reaction mixture and extracted with 10% MeOH in $CHCl_3$ three times. The combined organic layer was given water washes three times, followed by a brine wash. The organic layer was dried over sodium sulphate, filtered and concentrated. The crude residue obtained was purified by column chromatography using hexane-ethyl acetate (0-100%) followed by MeOH-$CHCl_3$ (0-6%) as the eluents and concentrated under vacuum and dried to obtain **8** as yellow gum. The shift of δ 5.63 ppm peak to δ δ 4.65 ppm indicated the formation of desired product **8**. ESI-MS: (*m/z*) calcd for $[M+H]^+$ for $C_{10}H_{13}NO_3S$: 228.0616and found 228.0646. ^1H NMR (400 MHz, $CDCl_3$) δ 4.65 (d, J = 2.5 Hz, 2H), 4.12 (m, 1H), 3.25 – 3.13 (m, 1H), 2.90 – 2.83 (m, 1H), 2.80 – 2.71 (m, 2H), 2.68 – 2.58 (m, 2H), 2.57 – 2.44 (m, 2H), 2.28 – 2.12 (m, 1H), 2.10 – 1.91 (m, 1H).

2.5 Synthesis of s*ubstituted aryl azides* (9a-p): A solution of substituted aryl amines (1.0 mmol) in 8N HCl was cooled to 0°C and stirred for 10-15 min, and aq. $NaNO_2$ (2.0 mmol) was added dropwise and stirred for 30 min at 0°C. To the resultant reaction mixture, aq. NaN_3 (2.0 mmol) was added dropwise and the reaction mixture was allowed to stir at room temperature for 6-16h. The progress of the reaction is monitored by TLC. After the completion, the reaction mixture was cooled to 0° C, and ice-cold water was added, and extracted with hexane and concentrated under reduced pressure without heat on a rotavapor to obtain the desired azides **9a-p.**

2.6 *General synthetic procedure for 1,2,3-triazoles* (10a-p and 11a-n): To a stirred solution of alkyne **7** (1.0 mmol) in ^{t}BuOH: H_2O (1:1) was added substituted aryl azide derivatives **9a-p,** followed by $CuSO_2.5H_2O$ (0.05 mmol), Sodium ascorbate (0.1 mmol). The resultant reaction mixture was then stirred at room temperature overnight (50°C heating for few compounds for 6-16 h). The progress of the reaction was monitored by TLC and MS. After the completion of reaction as indicated in TLC, formation of new spot, and confirmed the product mass was in LCMS, water was added to the reaction mixture and extracted with 10% MeOH in $CHCl_3$ three times. The combined organic layer was given water washes three times, followed by a brine wash. The organic layer was dried over sodium sulphate, filtered, and concentrated. The crude residue

obtained was purified by column chromatography using hexane-ethyl acetate (0-100%) followed by MeOH-$CHCl_3$ (0-12%) as the eluents and concentrated under vacuum and dried to obtain **10a-p** and **11a-n** as the desired products. All compounds were triturated with MTBE and dried to get title compounds. The disappearance of peaks at δ 2.46 and 2.52 ppm in ^{1}H NMR confirmed the formation of the desired products **10a-p** and **11a-n**.

3. Biological Procedures

3.1 Quorum sensing inhibition assay

P. aeruginosa MH602 P_{las}B::*gfp*(ASV) reporter strain, which harbors a chromosomal fusion of the *lasB* promoter to an unstable *gfp* gene and responds to the AHL 3-oxo-dodecanoyl homoserine lactone (3oxo-C12-HSL) [1], was used to evaluate the QSI activities of the synthesized compounds on QS signalling. An overnight culture was prepared in Luria-Bertani (LB10) media supplemented with gentamycin (40 µM). This culture was then diluted (1:100) in TSB/LB10 (4:1) medium supplemented with gentamycin (40 µM) and 200 µL aliquots were dispensed to flat bottom 96-well plate wells (Costar). The culture was supplemented with varying concentrations of test compounds dissolved in DMSO, with the final concentration of test compounds to be 250, 125, 62.5, 32, 16 and 8 µM. Wells with bacterial culture but no compound was used as negative control. Plates were incubated for 15 h at 37 °C with shaking at 120 rpm. After incubation, the reading of fluorescence (excitation, 485 nm; emission, 520 nm) and cell growth (OD at 600 nm) was taken in a plate reader (FLUOstar Omega, BMG Labtech). Each experiment was performed in triplicate and was repeated in at least three independent experiments.

3.2 Bacterial strains, growth conditions

Pseudomonas aeruginosa (MTCC 424) was procured from the Microbial Type Culture Collection and Gene Bank (MTCC), Institute of Microbial Technology (IMTECH), Chandigarh, India. Glycerol stock cultures were streaked onto Luria-Bertani (LB) agar plates and incubated overnight at 37°C. A single colony was used to inoculate in LB broth, which was incubated overnight at 37°C with shaking to ensure aeration.

3.3 Assessment of antimicrobial activity

The Microplate Alamar Blue Assay (MABA) was utilized, with slight modifications to established protocols, to assess the antimicrobial activity of selected test compounds [2]. A 96-well microtiter plate was set up by adding 200 µl of LB broth to designated blank wells. For experimental wells, 195 µl of LB broth was mixed with 5 µl of each test compound stock solution (2mg/mL), followed by serial dilution to obtain final concentrations ranging from 250 µM to 2 µM. Established antimicrobials—ciprofloxacin, amikacin, and cephalosporin—served as positive controls.

Bacterial cultures harvested during the logarithmic phase and adjusted to an OD_{600nm} of 0.001 were added (100 µl per well) to all wells except blanks. Plates were sealed and incubated at 37 °C for 3 hrs under static conditions. Following incubation, 20 µl of resazurin (Sigma) solution was dispensed into each well, and plates were returned to 37 °C for an additional 3–4 hrs (without shaking). A color change from purple to pink reflected bacterial metabolic activity and viability. The minimum concentration of a compound at which the purple color was retained indicated the minimum inhibitory concentration (MIC).

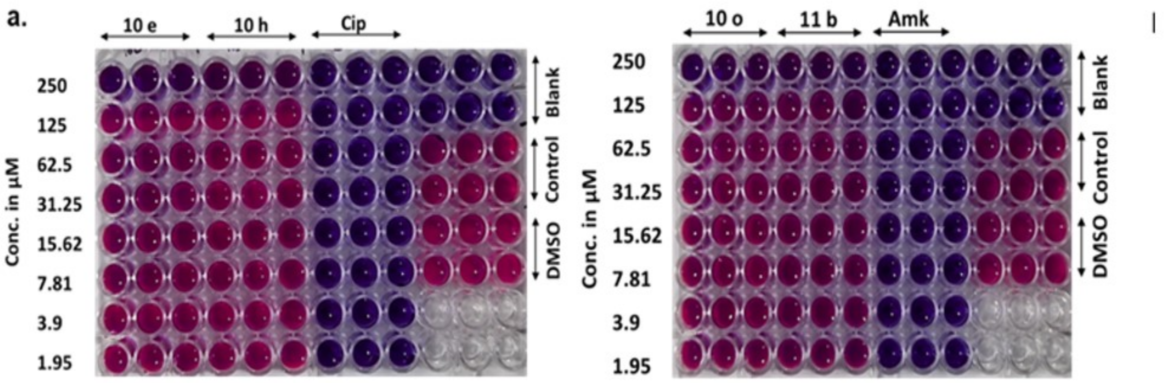

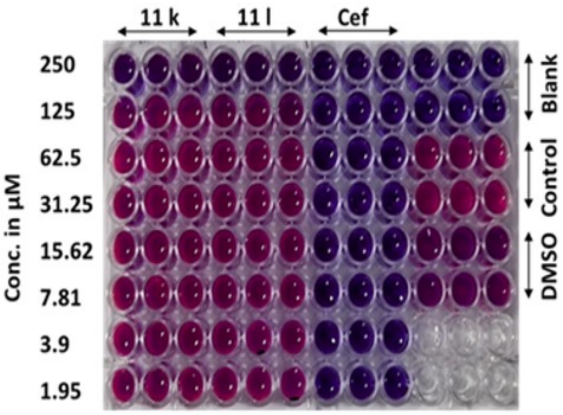

b.

Compound	MIC (µM)
10e	250
10h	250
10o	250
11b	250
11k	250
11l	250

Figure S1. Microplate Alamar blue assay (MABA) for *P. aeruginosa*. (a) The figure depicts the representative pictograms for the top 6 shortlisted compounds. The assay was performed with technical triplicates for each condition. The shortlisted compounds were tested at various concentrations (250-2 µM). (b) Tabulation of MIC values obtained for the top 6 shortlisted compounds

3.4 Biofilm inhibition assay

Biofilm inhibition assays were conducted in 24-well polystyrene plates with minor adaptations to previously published protocols [3]. An overnight bacterial culture was transferred into fresh LB broth and grown to log phase (OD_{600nm} 0.5–0.6). From this, 100 µl of culture was added to 1.9 ml of media—either containing the test compounds at sub-MIC concentrations or lacking them—in each well of the 24-well plate. Uninoculated media were used as a negative control. Plates were

incubated statically at 37 °C for 72 hrs. Following incubation, planktonic cells were removed, and wells were rinsed with sterile water to eliminate any remaining non-adherent bacteria or loosely attached biofilm. Biomass of the biofilm was fixed with 1 ml of methanol for 15 min and then air-dried. The dried biofilm was stained with 200 µl of 0.2 % crystal violet (Himedia) for 10 min, after which excess stain was washed off with sterile water. Bound stain was subsequently solubilized with 500 µl of 0.5 M glacial acetic acid. Absorbance was measured at 570 nm using a microplate reader (SpectraMax iD3 Multi-Mode Microplate Readers, Molecular Devices) to quantify biofilm biomass.

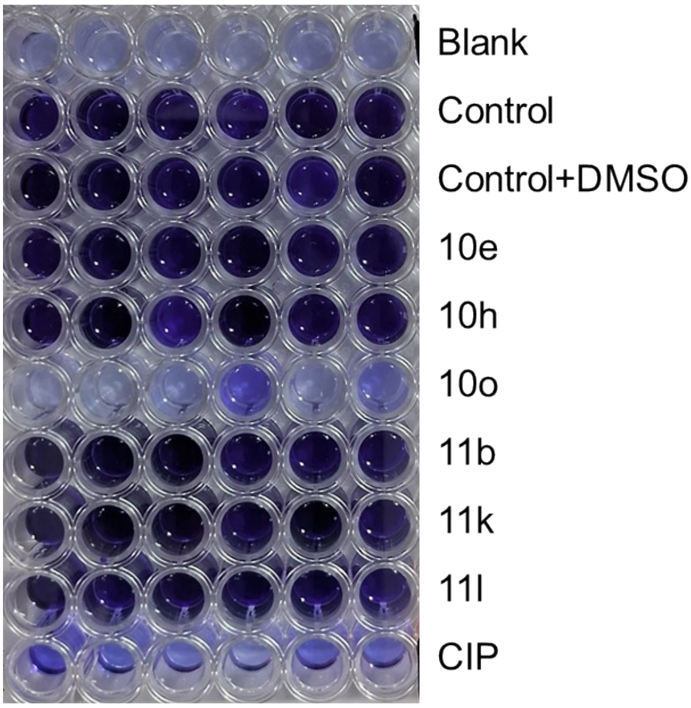

Figure S2. Biofilm inhibition assay for *P. aeruginosa*. Microplate pictogram of glacial acetic acid extracted crystal violet fraction obtained from stained biofilm biomass. The experiment were conducted in six technical replicates to ensure reproducibility.

3.5 Pyocyanin estimation

Pyocyanin inhibition was evaluated following a previously described procedure with minor adjustments [4-5]. Bacterial cultures were incubated for 72 hrs at 37 °C in the presence of test compounds at sub-MIC concentrations. After incubation, samples were centrifuged at 6000 rpm for 2 min, and 400 µL of the resulting supernatant was combined with 210 µL of chloroform in a microcentrifuge tube and vortexed for 1 min. The mixture was subsequently centrifuged at 13,000 rpm for 5 min, after which 150 µL of the organic (chloroform) phase was separated and neutralized by adding 400 µL of 0.1 N hydrochloric acid. This biphasic solution was vortexed again for 1 min and centrifuged similarly. Finally, 200 µL of the aq.ueous phase was transferred to a 96-well plate,

and pyocyanin concentration was determined by measuring absorbance at 290 nm using a microplate reader (SpectraMax iD3 Multi-Mode Microplate Readers, Molecular Devices).

Percentage reduction in pyocyanin was calculated using the formula,

% reduction in pyocyanin = [(control - sample) / control] X 100

3.6 Protease assay

To evaluate the inhibition of protease activity in *P. aeruginosa*, a colorimetric assay using azocasein as the substrate was employed, with minor adjustments to standard protocols [4-5]. After 72 hrs of incubation at 37 °C with sub-MIC levels of test compounds, bacterial cultures were centrifuged at 6000 rpm for 2 min. Next, 10 µL of the resulting supernatant was combined with 100 µL of a substrate solution containing 1.25 % azocasein in protease buffer, and the mixture was incubated at 37 °C with shaking at 200 rpm for 30 min. Upon completion, 50 µL of the reaction was transferred to tubes containing 200 µL of 1 % nitric acid to halt the enzymatic activity. The samples were then centrifuged at 13,000 rpm for 2 min to pellet down the precipitated proteins and peptides. Subsequently, 50 µL of the clear supernatant was mixed with 150 µL of 0.5 % sodium hydroxide in a 96-well plate, and absorbance was assessed at 440 nm using a microplate reader (SpectraMax iD3 Multi-Mode Microplate Readers, Molecular Devices). Protease inhibition was quantified by comparing absorbance values with those of the untreated control.

Percentage reduction/inhibition in protease activity was calculated using the formula,

% inhibition in protease = [(control - sample) / control] X 100

3.7 Cytotoxicity studies: Cell proliferation assay – IC_{50} experiments

Cell proliferation assay was performed using WST-1 reagent (Cat#. 05015944001, Roche) according to the manufacturer's instructions for the indicated time points [2-4]. Around 5,000–7,000 cells were seeded per well in 96-well plates and then treated with the compounds at different concentrations (5,10,20,40,80,160,320 µM) or with Neratinib Maleate (5,10,20,40,80,160,320 nM). After 24 hrs of incubation at 37°C, WST-1 reagent was added to the cells, and readings were taken at a wavelength of 450 nM. The half-maximal inhibitory concentration (IC_{50}) values were calculated by GraphPad Prism version 5 at different concentrations for each compound.

3.8 Statistical analysis

Graphs and statistical analyses were generated using GraphPad Prism 8. Group means were statistically evaluated using Two-Way ANOVA, with significance established at *p* values less than 0.05. Data are reported as mean ± standard deviation, except where alternative formats are specified in the figure legends

4. *In Silico* **Predicted ADME and Drug-Likeness Properties**

The *in silico* ADME predictions revealed the new compounds' critical physical and chemical properties. These include LogP, number of rotatable bonds (R.B.), molecular weight (M.W.), count of aromatic heavy atoms (nAH), hydrogen bond donors (HBD), hydrogen bond acceptors (HBA), octanol/water partition coefficient (LogP), molecular refractivity (M.R.), and topological polar surface area (TPSA). All compounds adhere to Lipinski's rule of five with no more than two violations, indicating they possess drug-like properties and are suitable for further research.

5. **Molecular docking and MMGBSA studies:**

Molecular docking was carried out to the designed molecules using the glide module of the Schrödinger software. Initially, all the designed ligands were sketched in the maestro interface (Schrödinger, LLC, New York, NY, 2025) and prepared using Ligprep wizard applying OPLS_2005 forcefield[6]. The chirality was determined from the 3D structures. Then the LasR protein (PDB: 2UV0, resolution: 1.8Å) was imported from protein databank and prepared by adding hydrogens and filling the missing residues using the prime module via protein preparation wizard of the maestro interface in the Schrödinger software. A receptor grid was generated using the co-ordinates of the co-crystal (x = -15.72, y = -9.18, z = 88.57), and the same was used to perform molecular docking via ligand docking wizard in the extra precision (XP) mode[7,8]. The 3D pose and the interaction of each molecule is depicted in the figure S3-S5. Post-docking, the docked conformations were used to evaluate the binding free energy by performing MMGBSA calculations[9]. These calculations were carried out in VSGB solvent medium, and OPLS_2005 forcefield was used. The calculations are carried out based on the following formula:

$$\Delta G_{bind} = (\Delta G_{ligand} + \Delta G_{receptor}) - \Delta G_{complex}$$

Where, ΔG_{bind} = binding free energy; ΔG_{ligand} = change in free energy of the ligand; $\Delta G_{receptor}$ = change in free energy of the receptor; $\Delta G_{complex}$ = change in free energy of the protein-ligand complex.

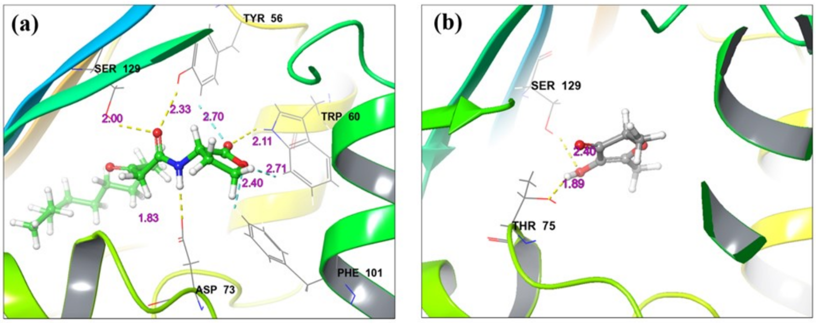

Figure S3. 3D interactions image of the (a) cocrystal, (b) furanone C30 at the active site of LasR target protein (**PDB: 2UV0**)

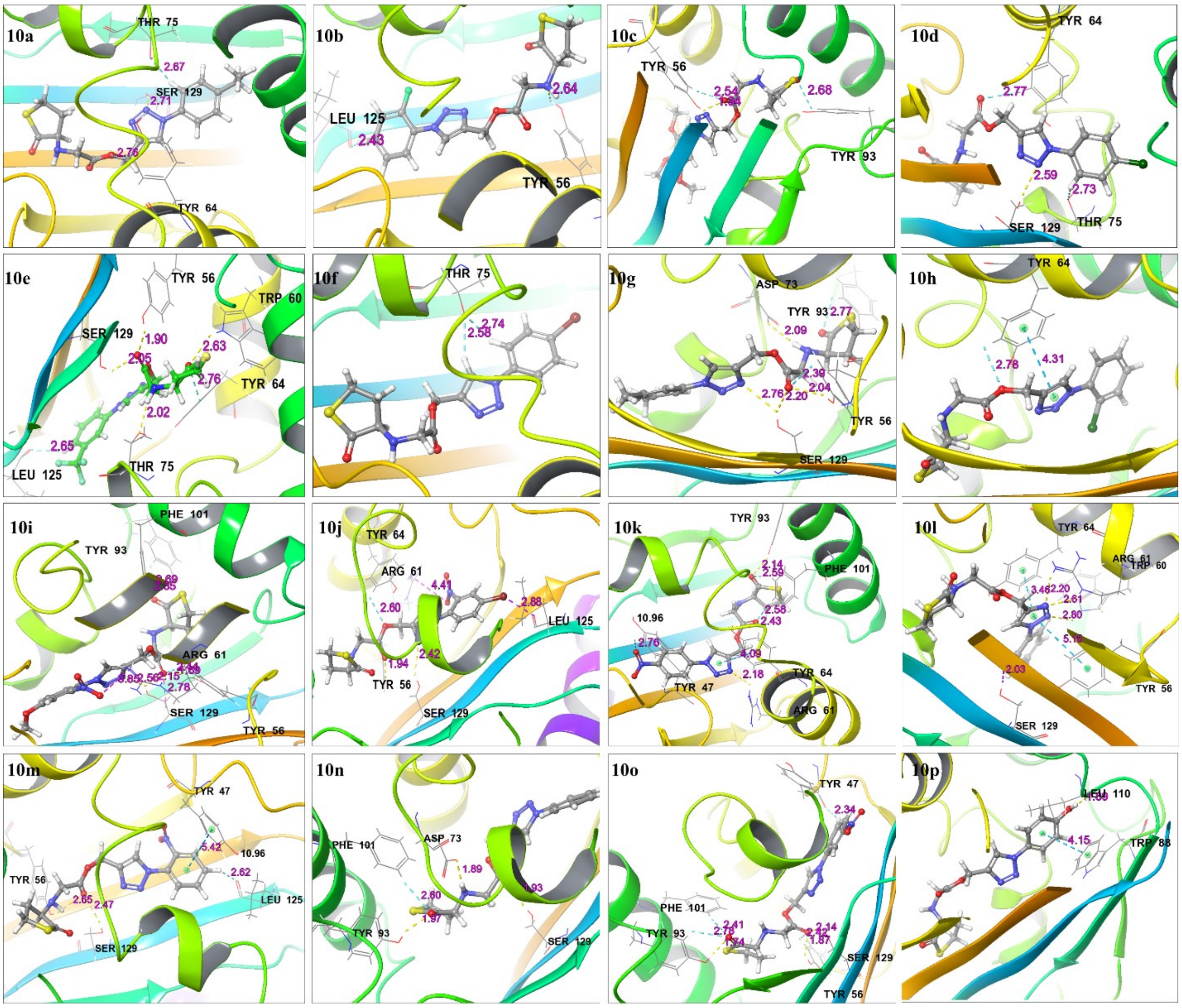

Figure S4. 3D interactions image of the glycinate derivatives (compounds **10a-p)** at the active site of LasR target protein (**PDB: 2UV0**)

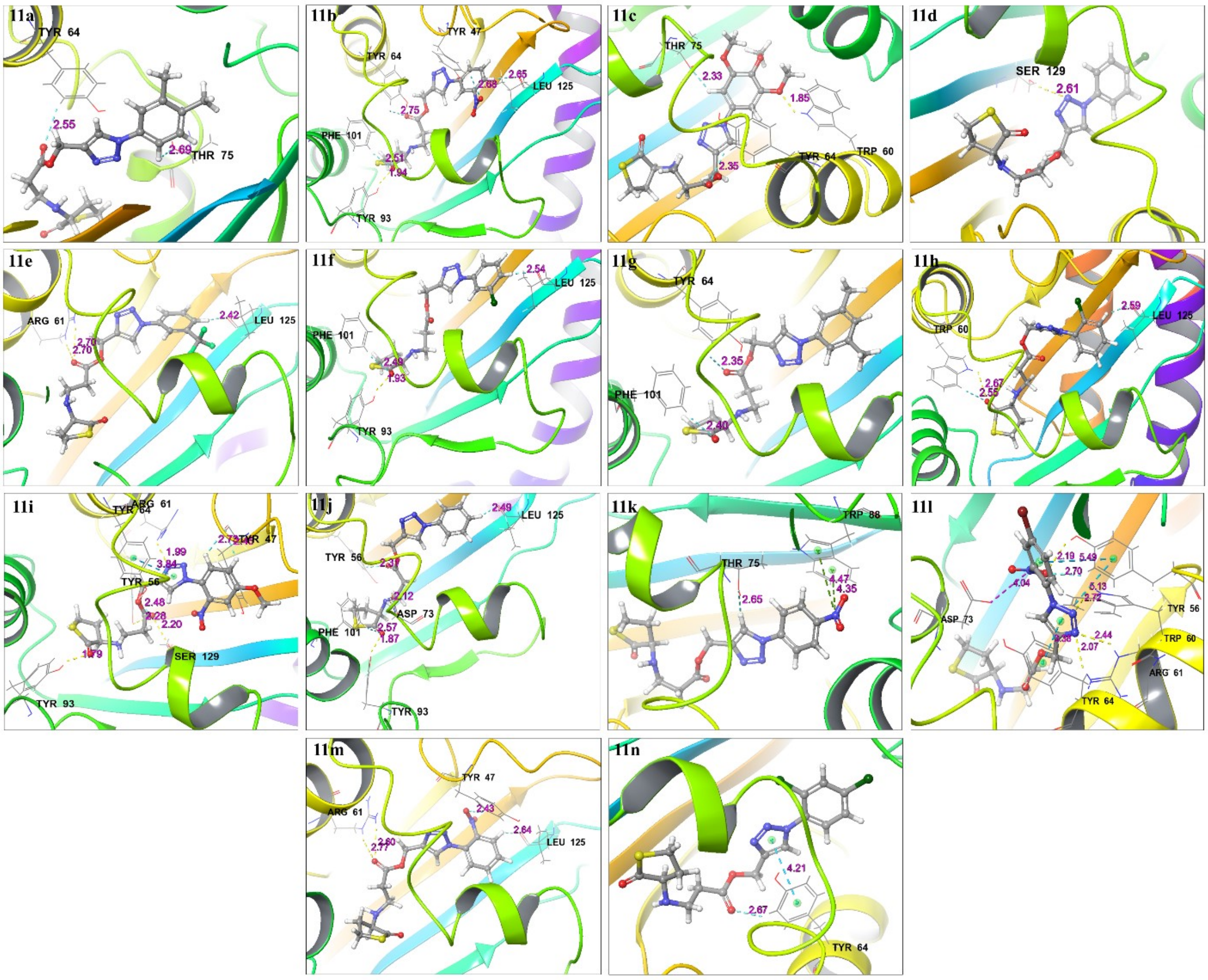

Figure S5: 3D interactions image of the propanoate derivatives (compounds **11a-n)** at the active site of LasR target protein (**PDB: 2UV0**)

6. Molecular Dynamics simulation (MDS) Studies

To assess the impact of the solvent system on the protein-ligand complex, the most active compounds were subjected to molecular dynamics simulations[10]. A Transferable Intermolecular Potential 3 Points (TIP3P) solvent system was initially added to the protein-ligand complex within a cubic system boundary of 10Å^3, followed by ions and salt concentration to neutralize the system. A minimization step was carried out to attain equilibrium, followed by molecular dynamics (MD) simulations for 100 ns at constant number of particles (N), constant temperature (T), and constant pressure (P) (NPT) ensemble at 310 K, with a thermostat relaxation time of 200 ps and a pressure of 1 atm. The Nose-Hoover thermostat and Martyna-Tobias-Klein barostat maintained the temperature and pressure[11,12]. Simulation data were recorded every 10 ps.

7. **^{1}H NMR spectra intermediates (4-8) and final compounds (10a-p, 11a-n)**:

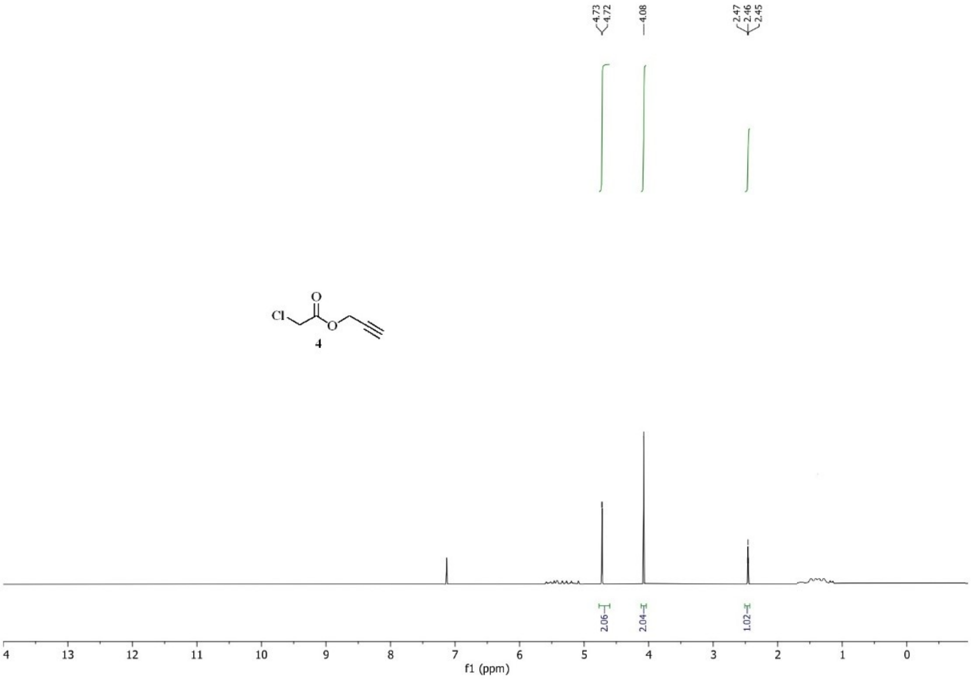

^{1}H-NMR spectrum of 4 (400 MHz, $CDCl_3$)

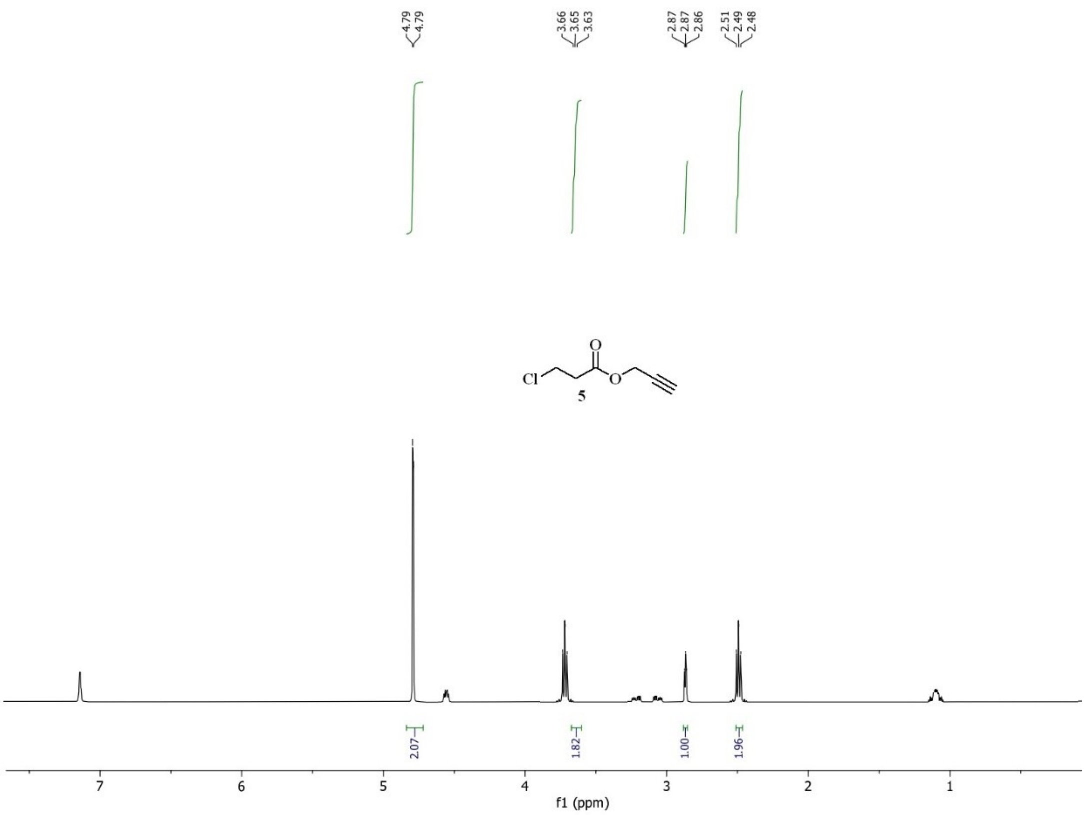

^{1}H-NMR spectrum of 5 (400 MHz, $CDCl_3$)

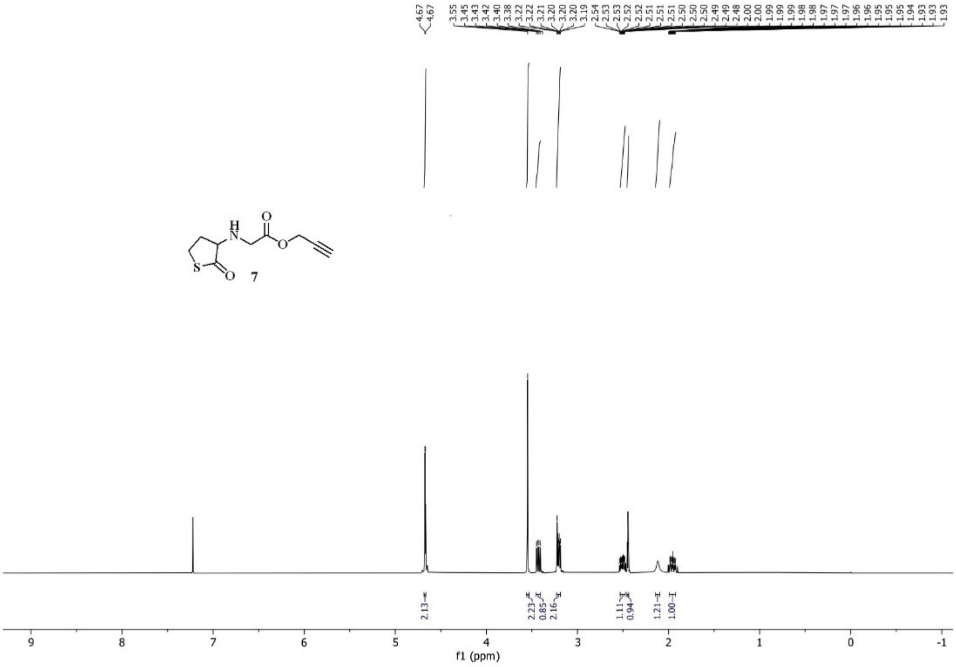

^{1}H-NMR spectrum of 7 (400 MHz, $CDCl_3$)

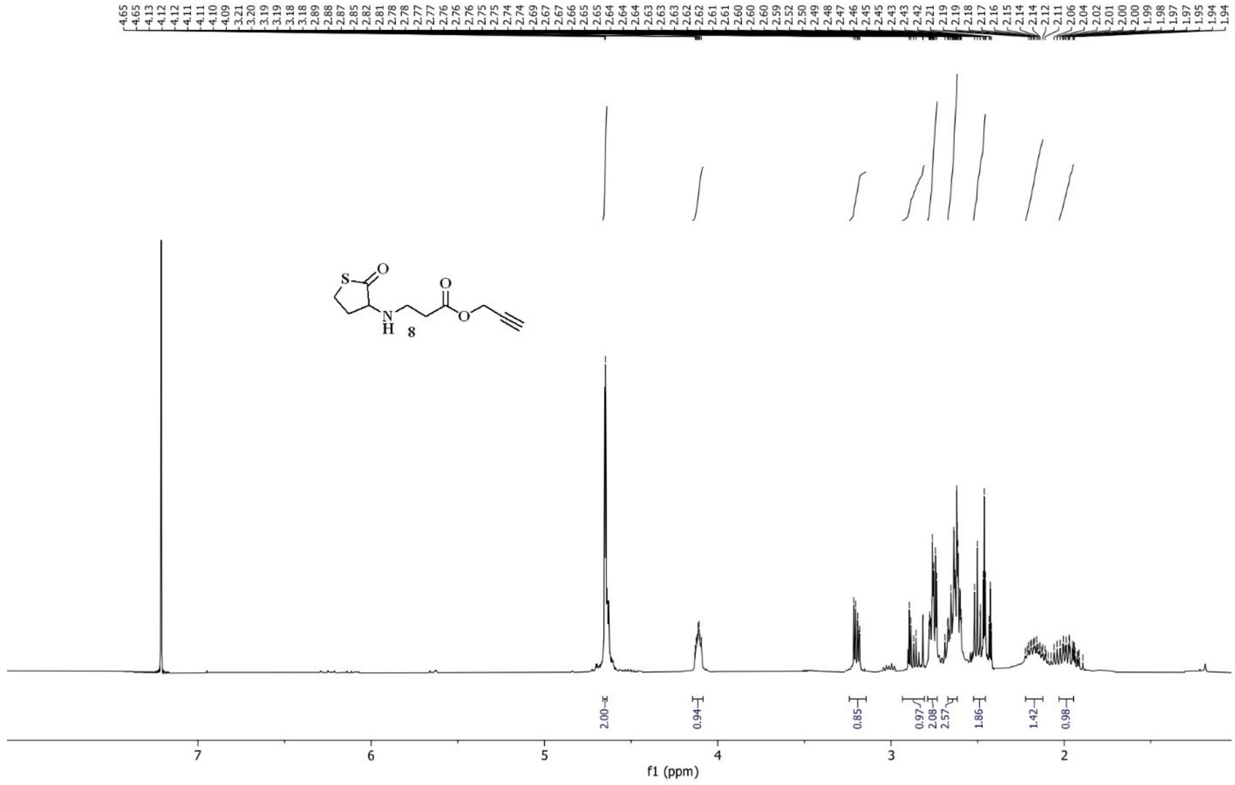

^{1}H-NMR spectrum of 8 (400 MHz, $CDCl_3$)

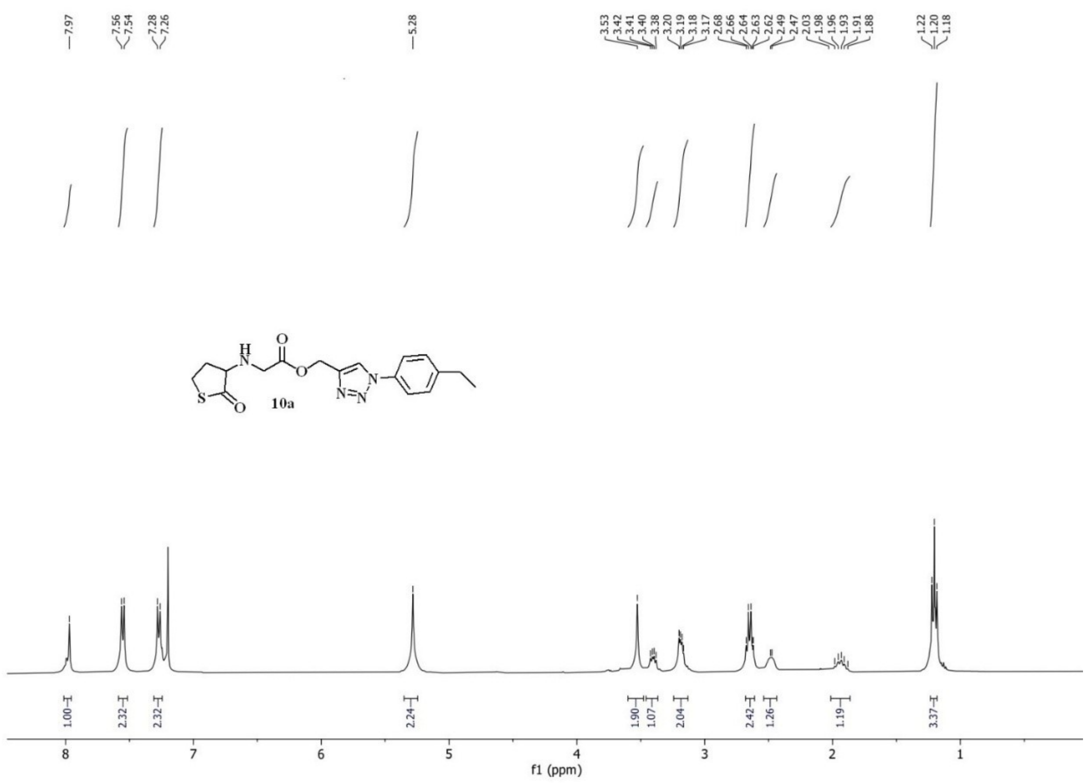

^{1}H-NMR spectrum of 10a (400 MHz, CDCl$_3$)

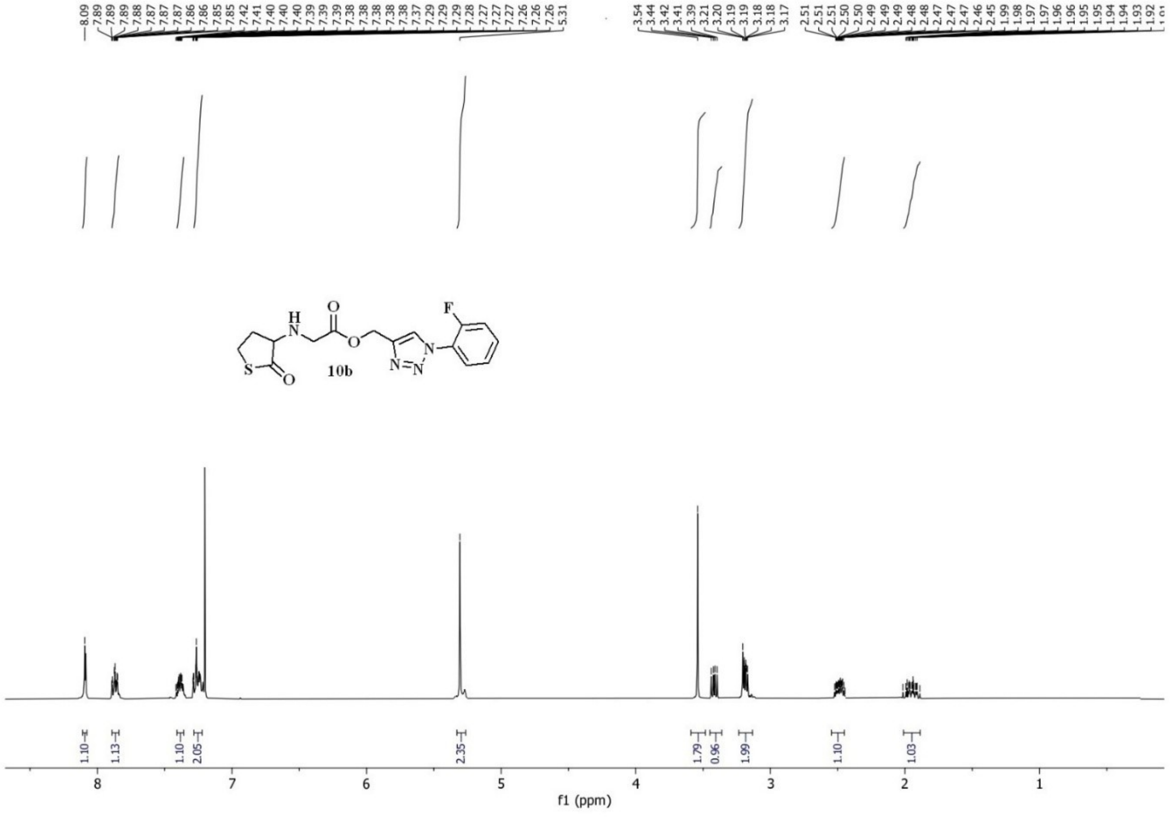

^{1}H-NMR spectrum of 10b (400 MHz, CDCl$_3$)

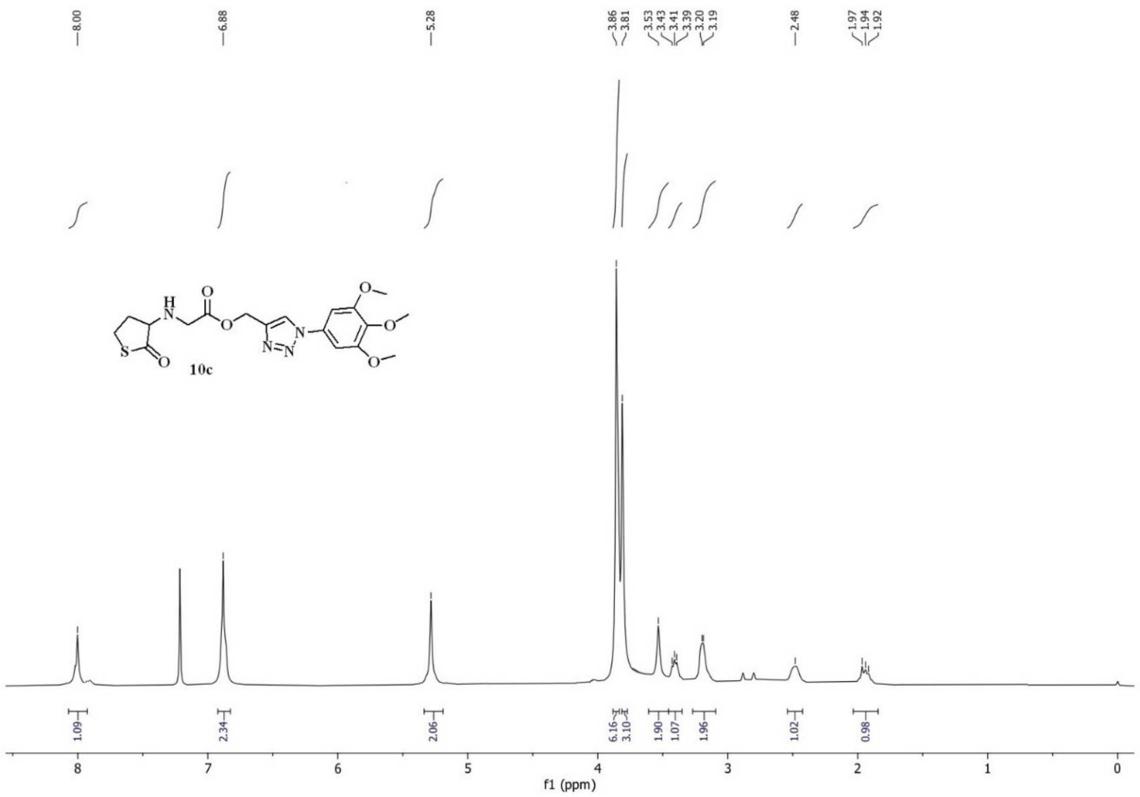

¹H-NMR spectrum of 10c (400 MHz, CDCl$_3$)

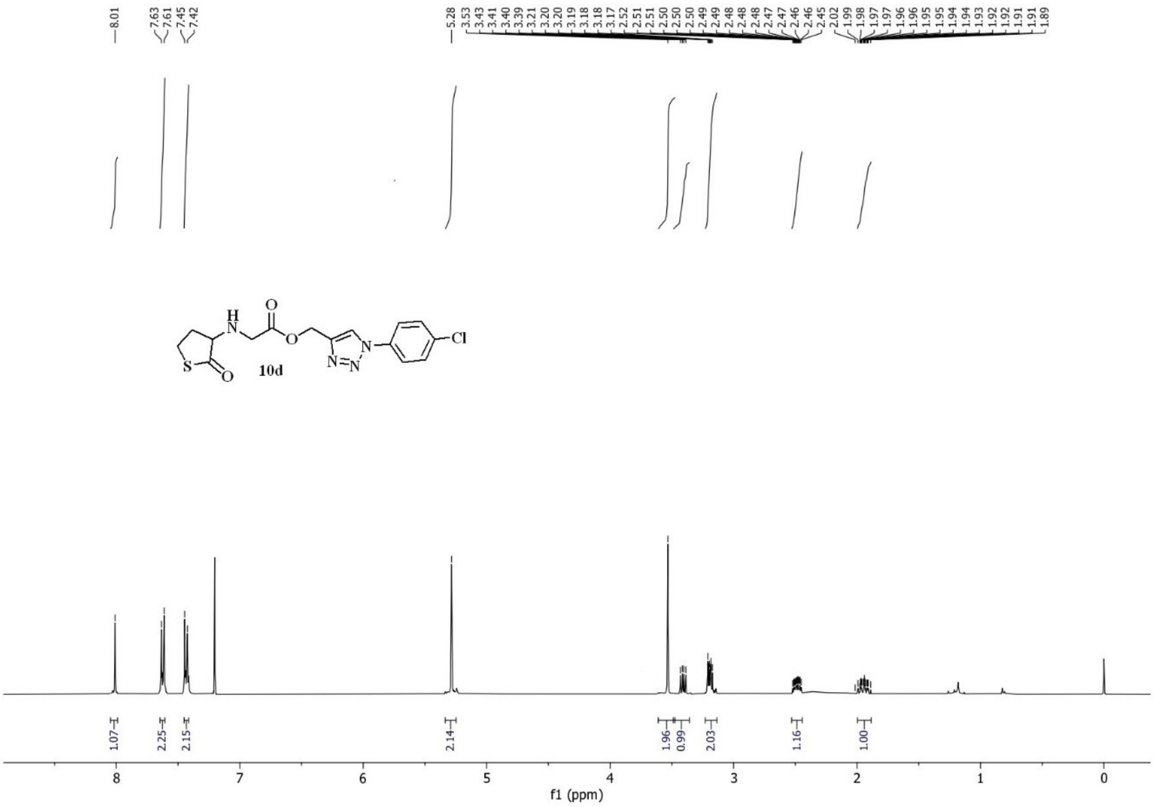

¹H-NMR spectrum of 10d (400 MHz, CDCl$_3$)

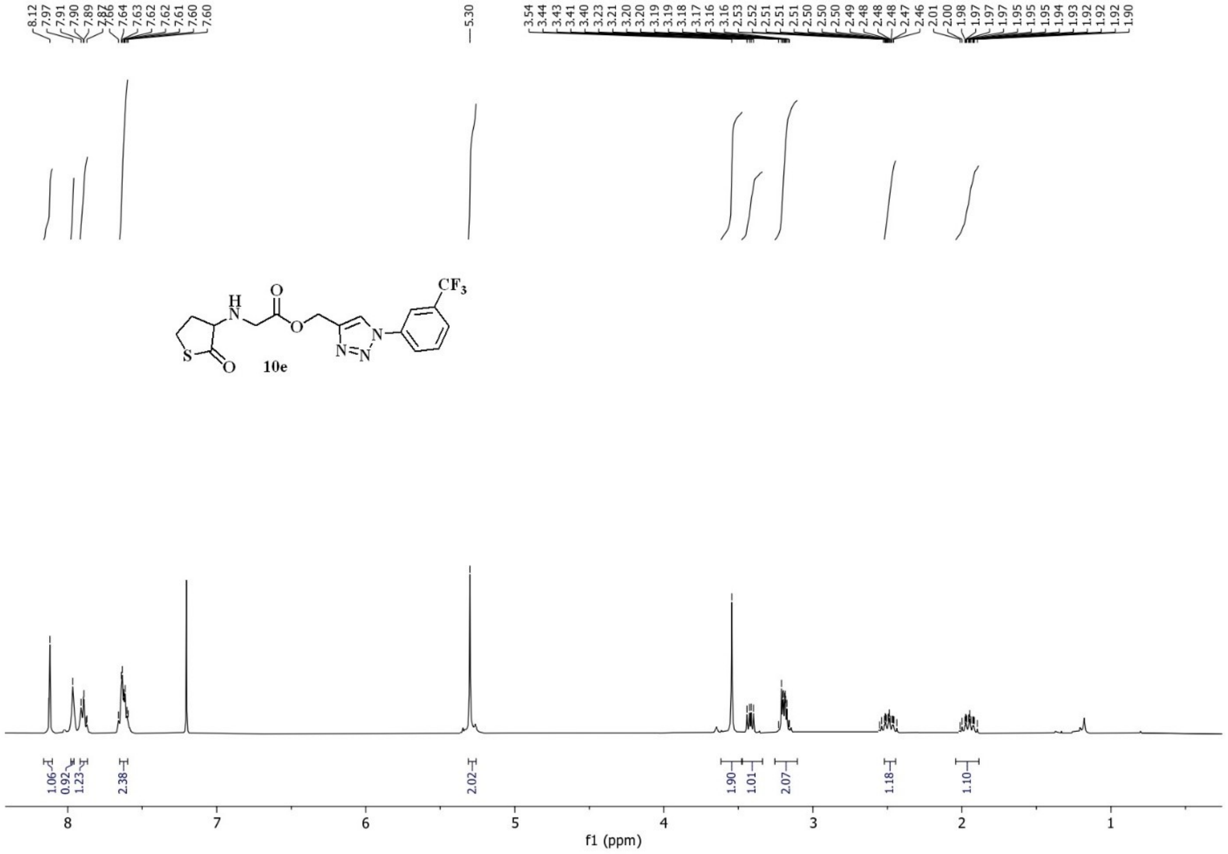

^{1}H-NMR spectrum of 10e (400 MHz, $CDCl_3$)

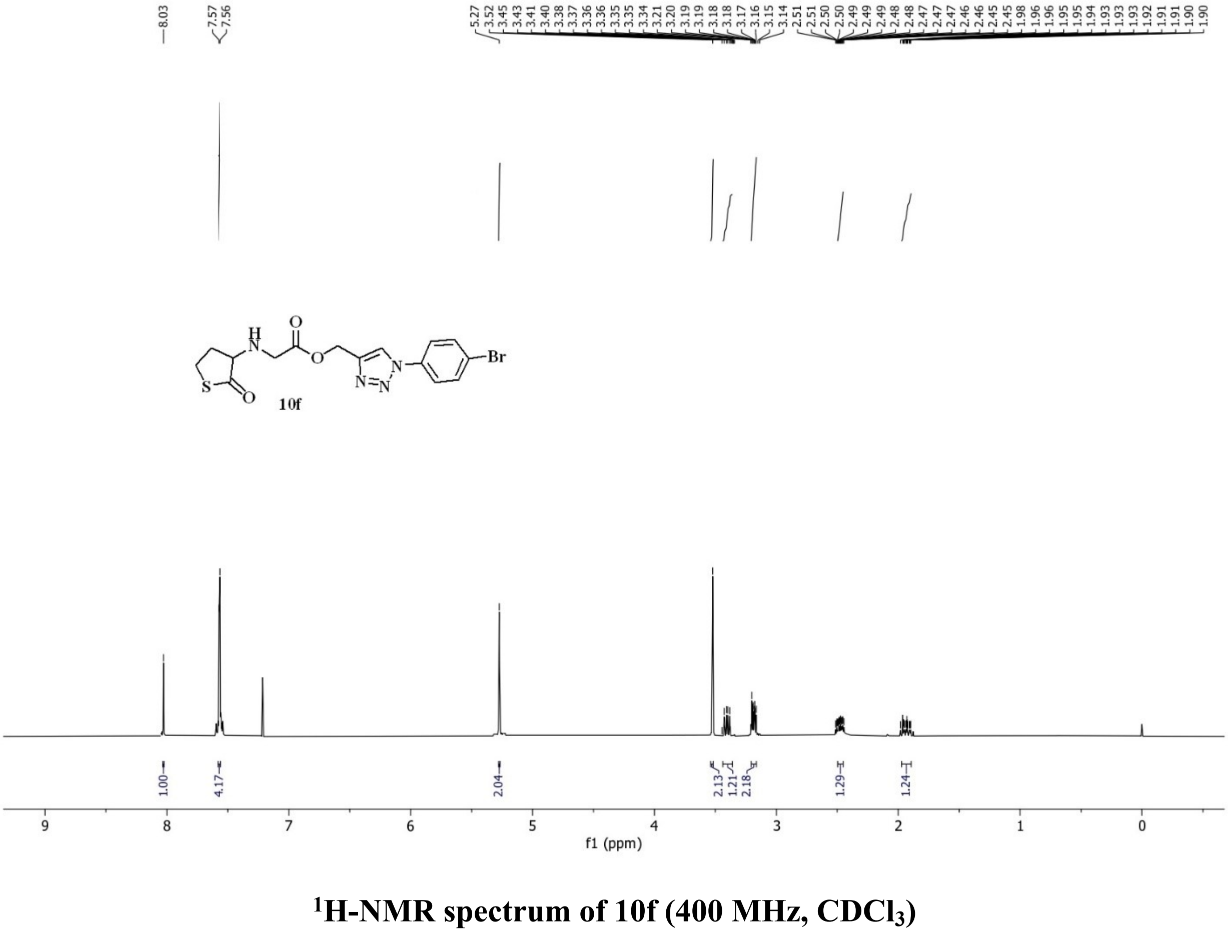

^{1}H-NMR spectrum of 10f (400 MHz, $CDCl_3$)

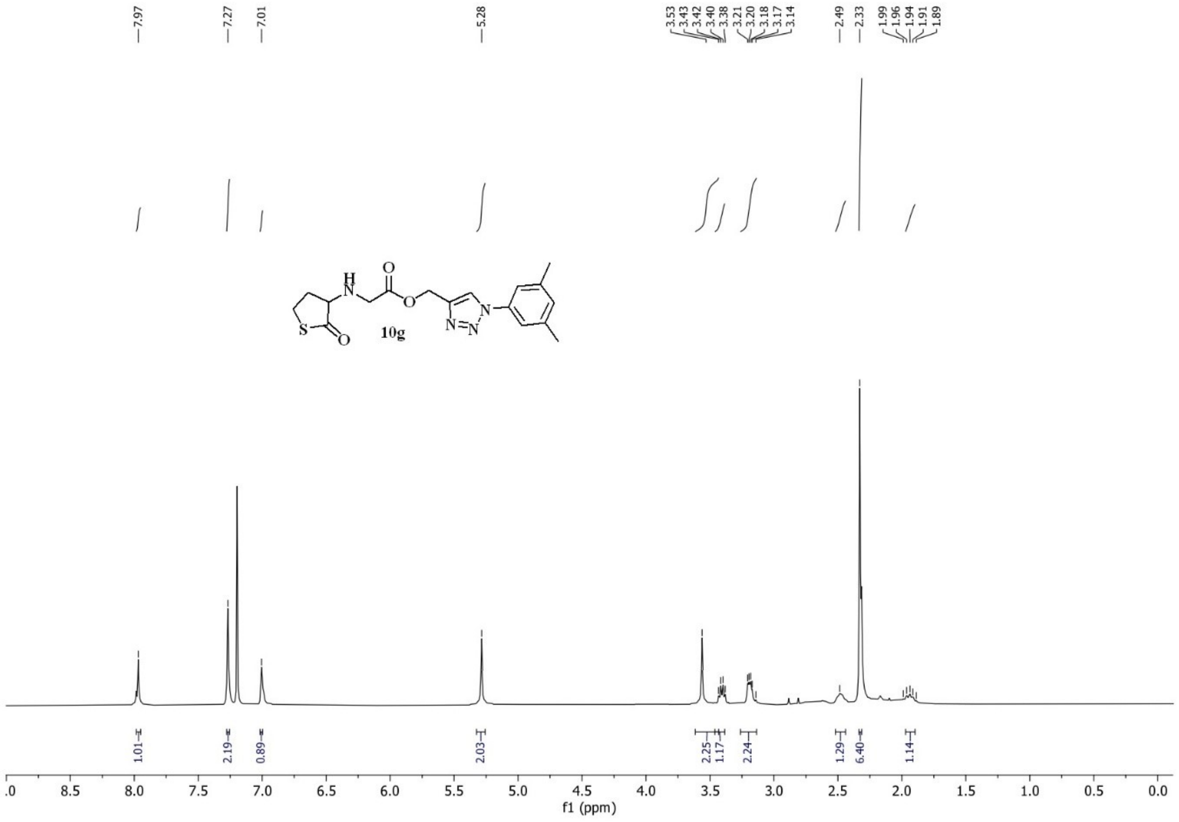

^{1}H-NMR spectrum of 10g (400 MHz, $CDCl_3$)

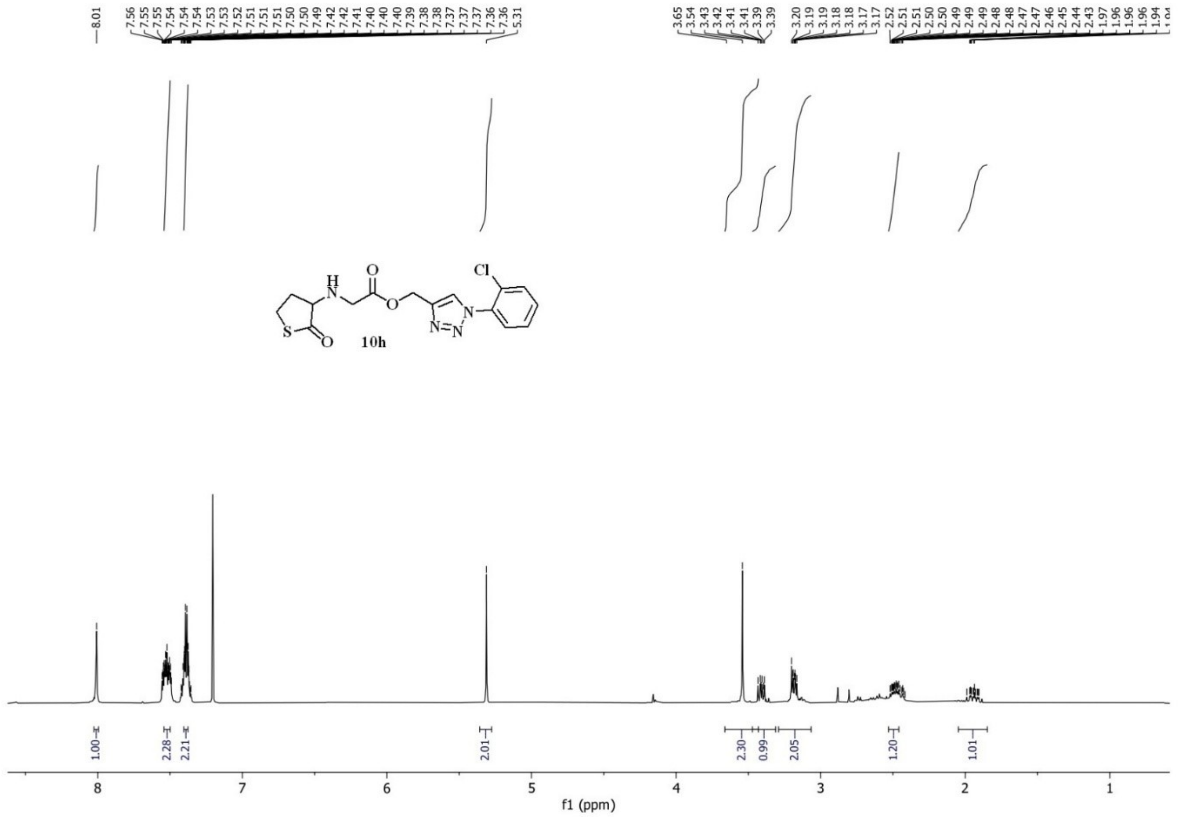

^{1}H-NMR spectrum of 10h (400 MHz, $CDCl_3$)

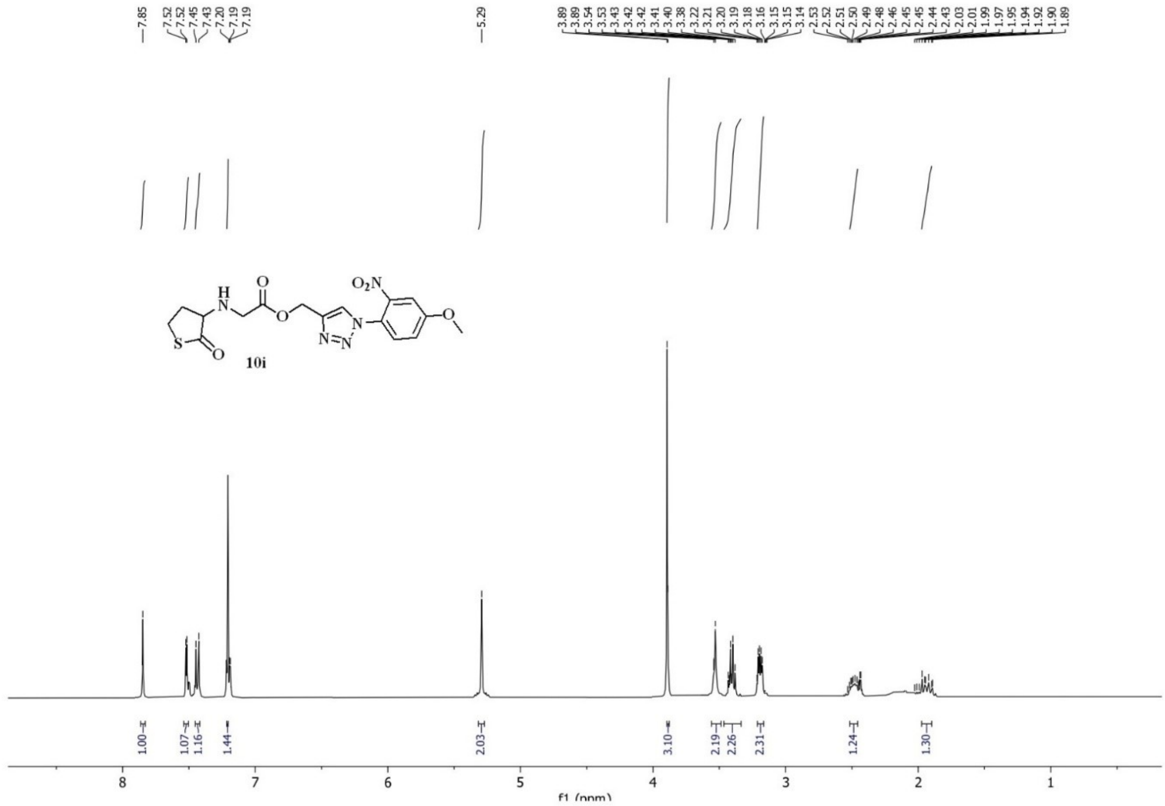

^{1}H-NMR spectrum of 10i (400 MHz, CDCl$_3$)

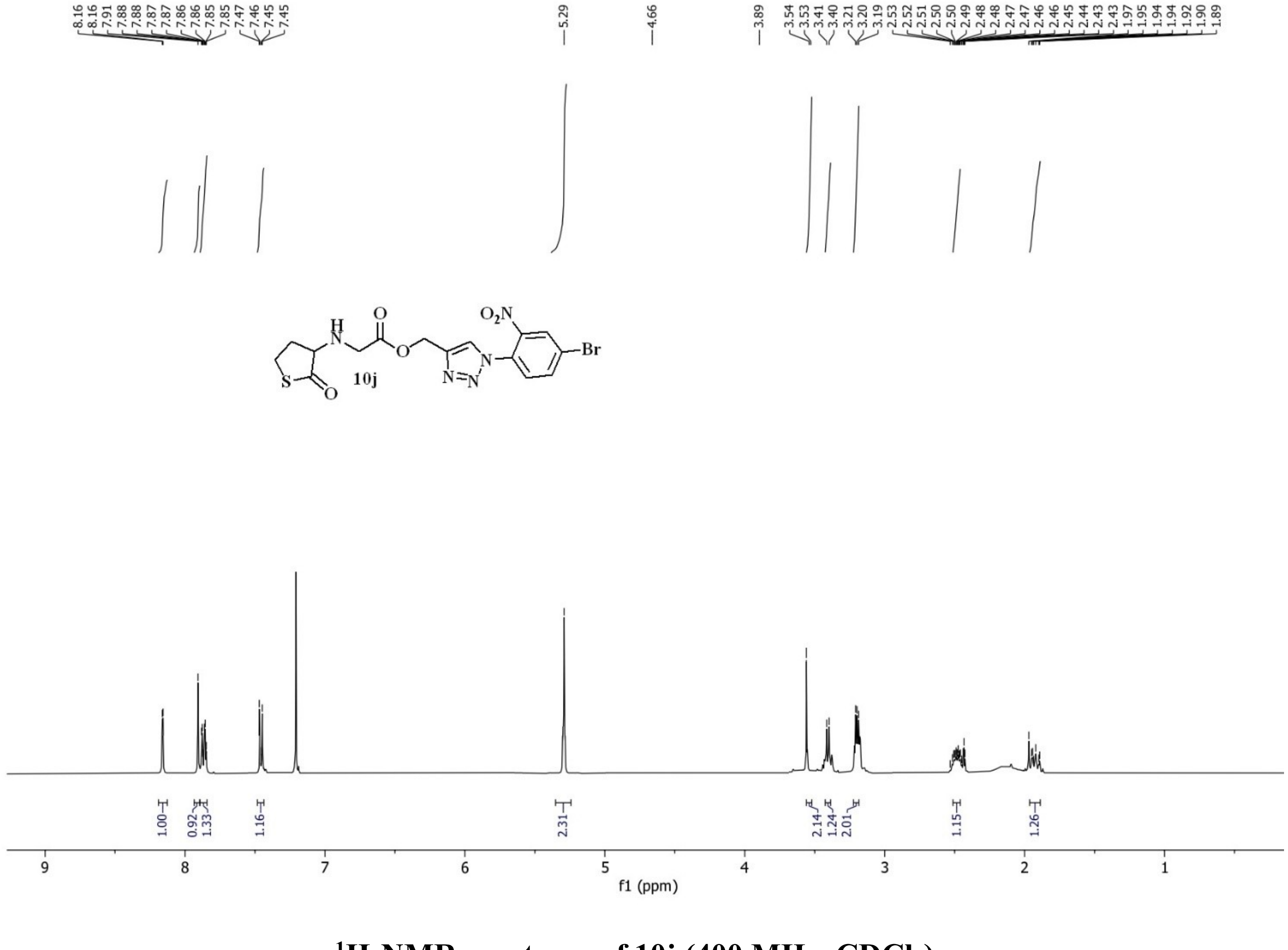

^{1}H-NMR spectrum of 10j (400 MHz, CDCl$_3$)

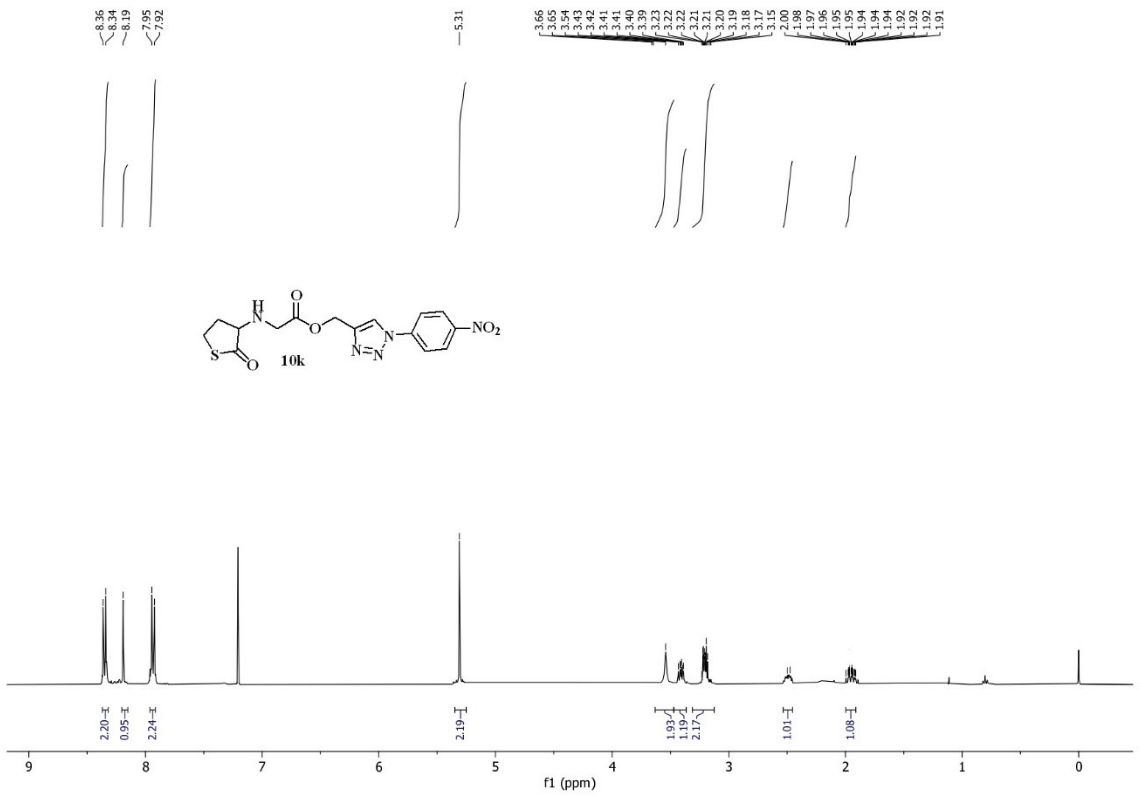

10k

^{1}H-NMR spectrum of 10k (400 MHz, CDCl$_3$)

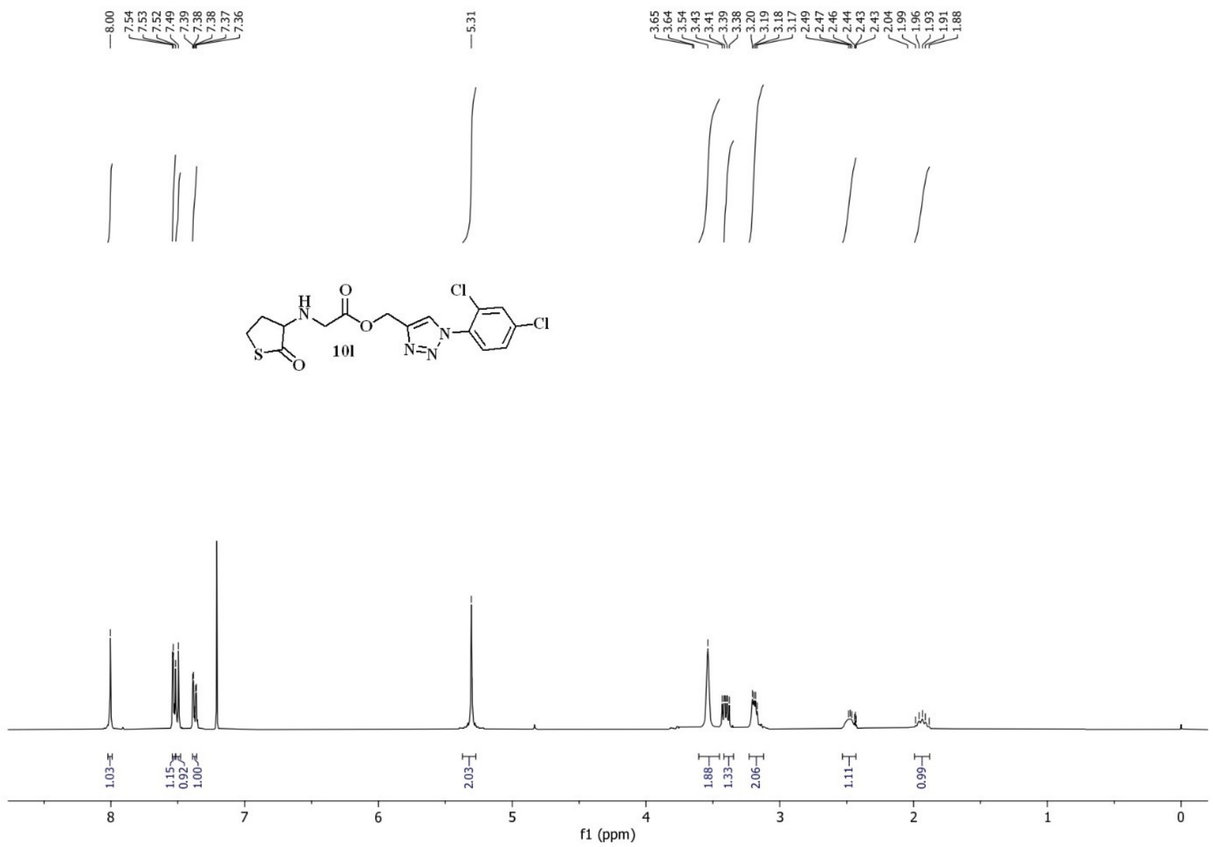

10l

^{1}H-NMR spectrum of 10l (400 MHz, CDCl$_3$)

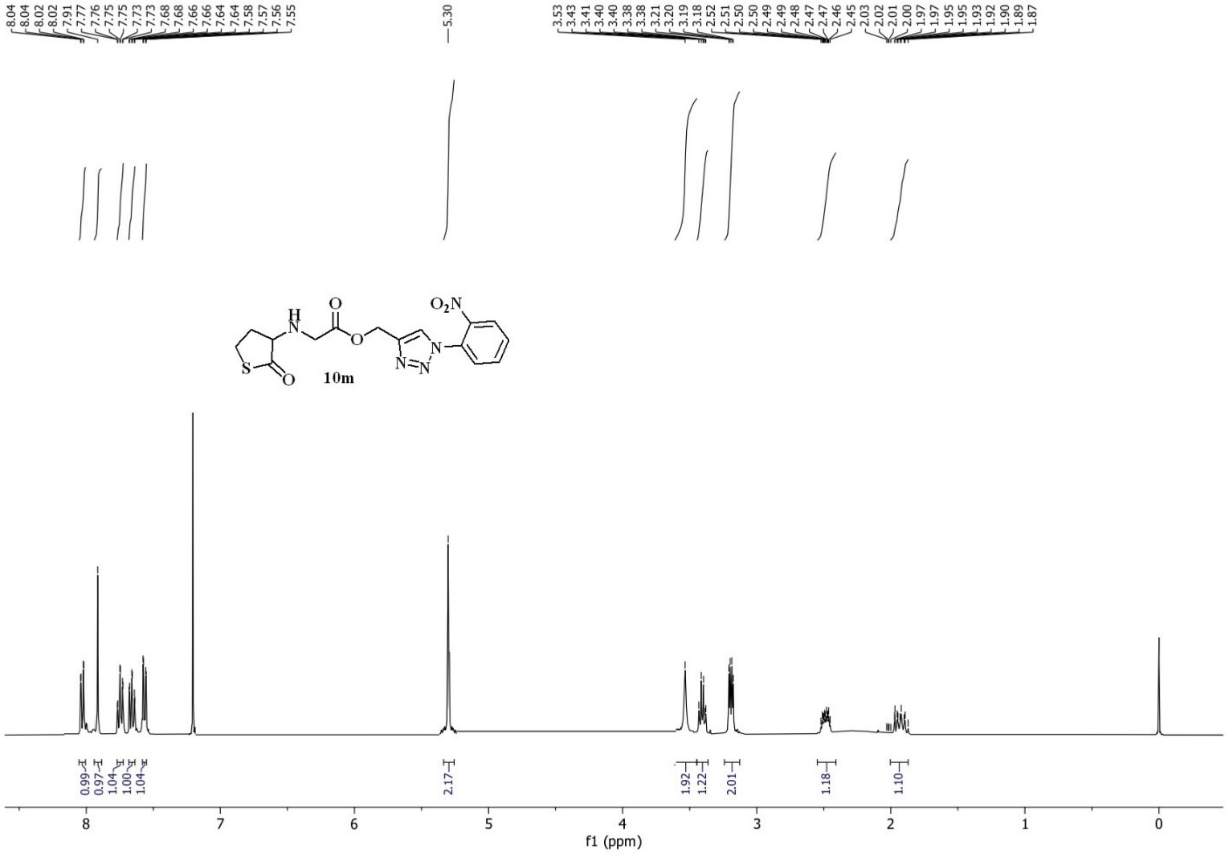

^{1}H-NMR spectrum of 10m (400 MHz, $CDCl_3$)

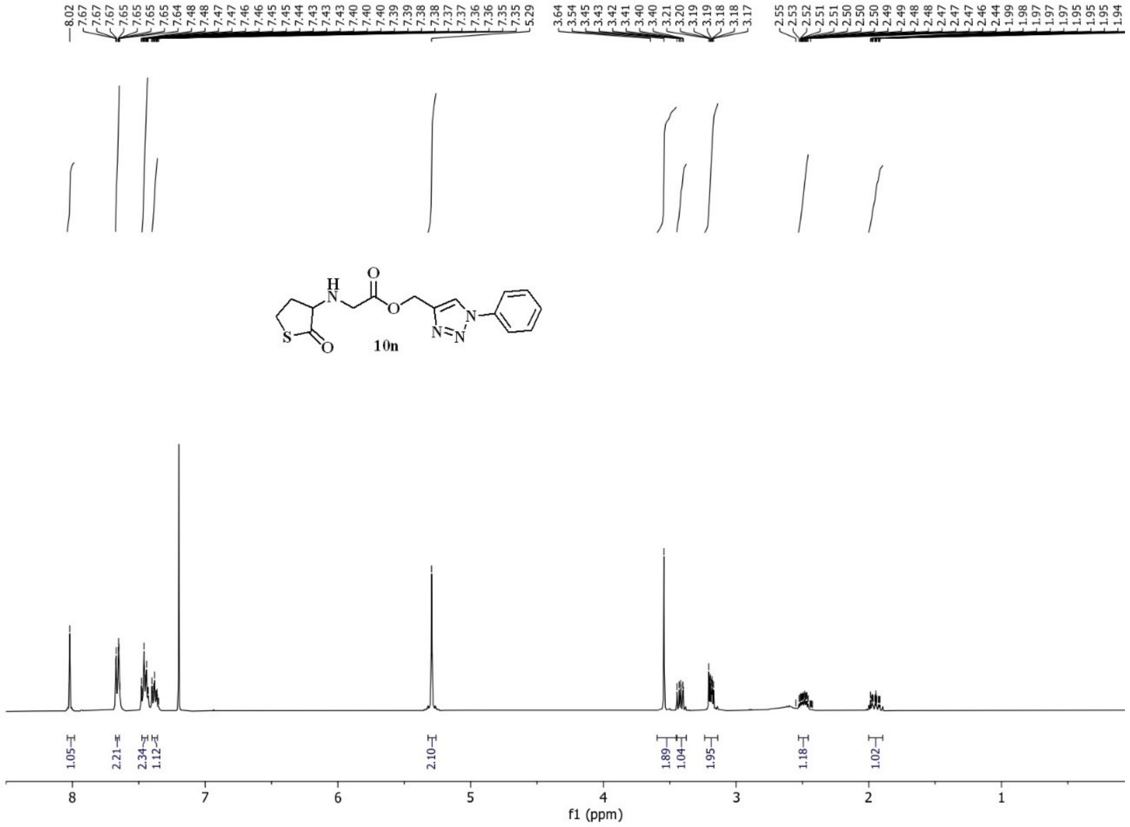

^{1}H-NMR spectrum of 10n (400 MHz, $CDCl_3$)

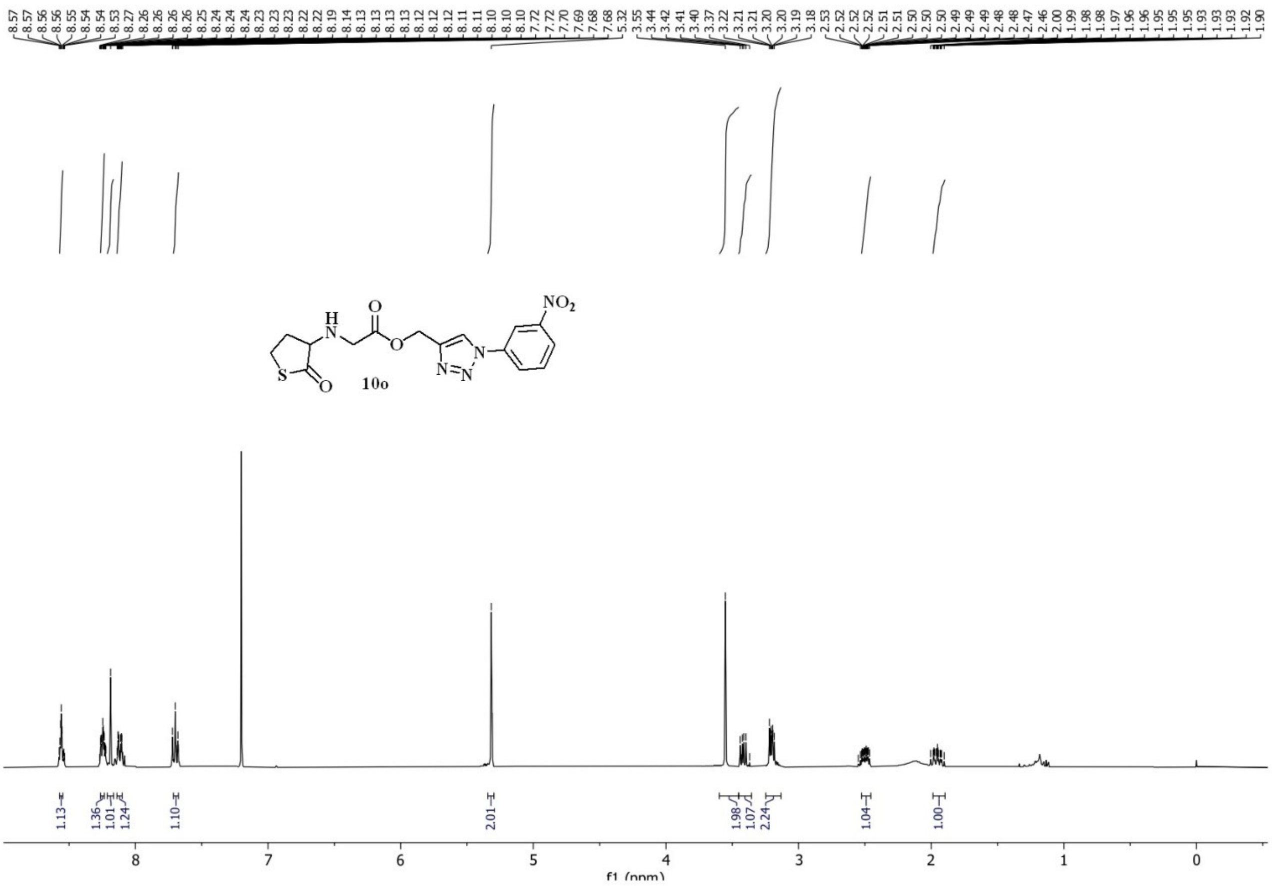

^{1}H-NMR spectrum of 10o (400 MHz, $CDCl_3$)

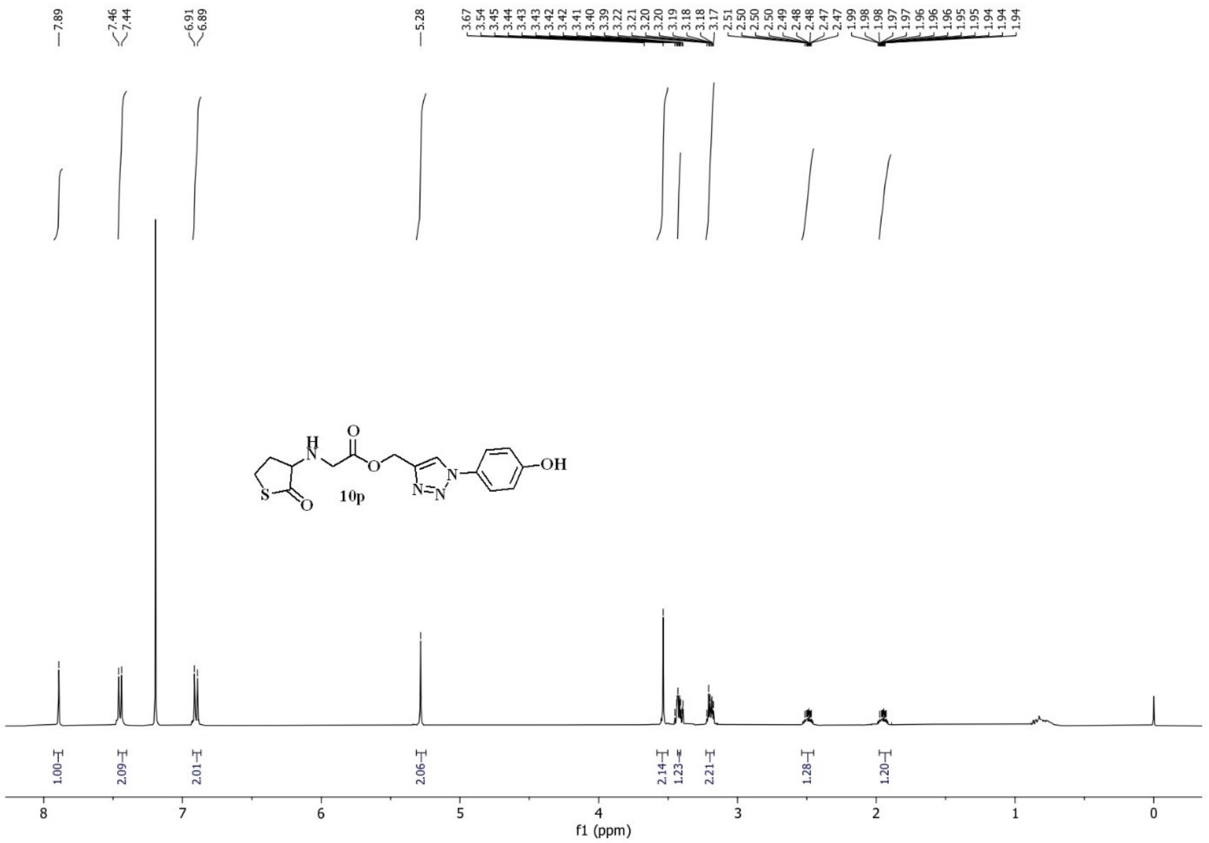

^{1}H-NMR spectrum of 10p (400 MHz, $CDCl_3$)

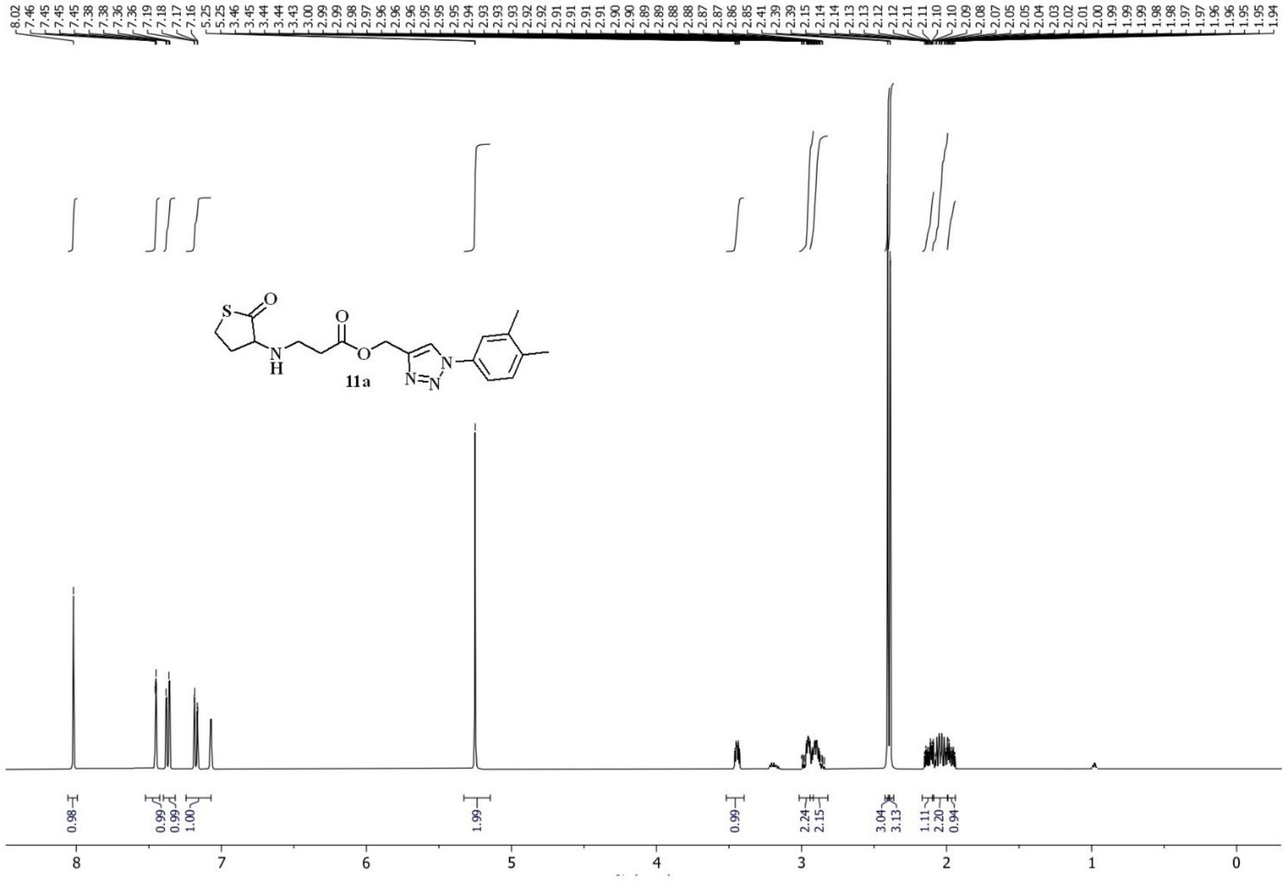

^{1}H-NMR spectrum of 11a (400 MHz, CDCl$_3$)

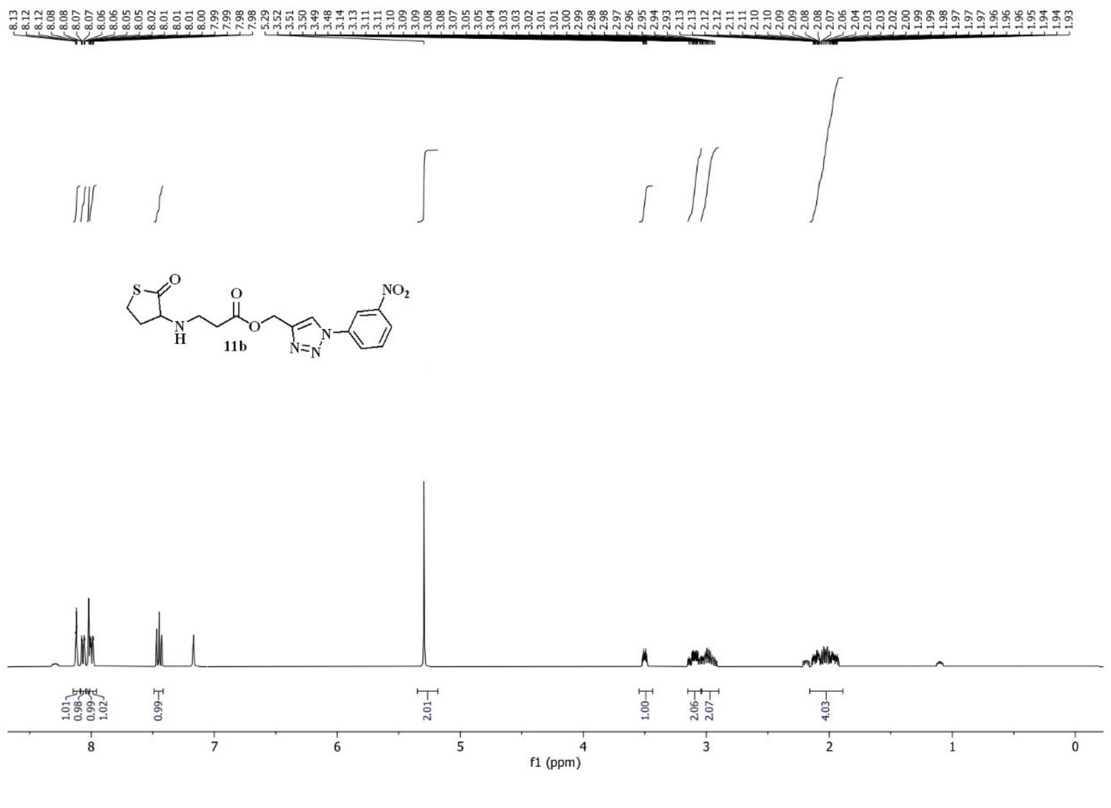

^{1}H-NMR spectrum of 11b (400 MHz, CDCl$_3$)

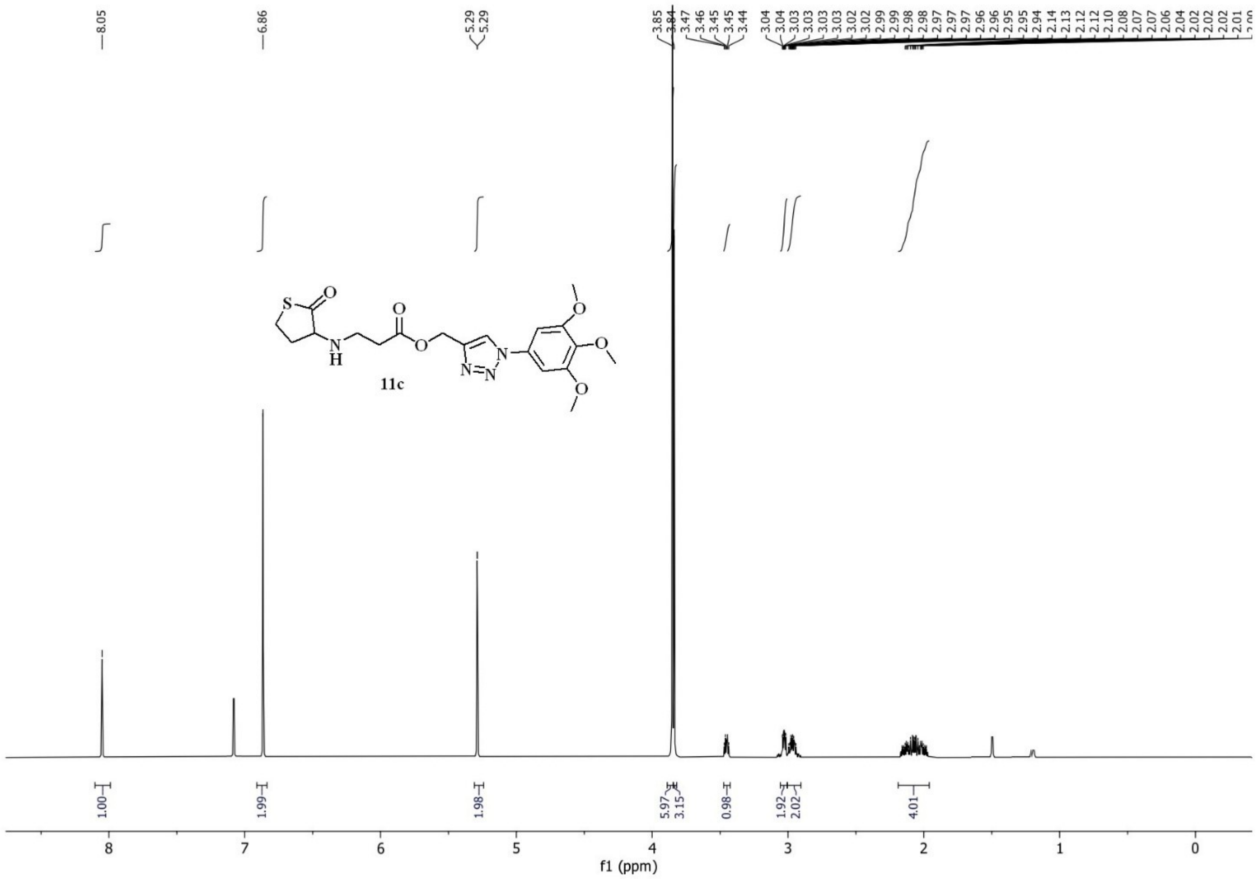

^{1}H-NMR spectrum of 11c (400 MHz, $CDCl_3$)

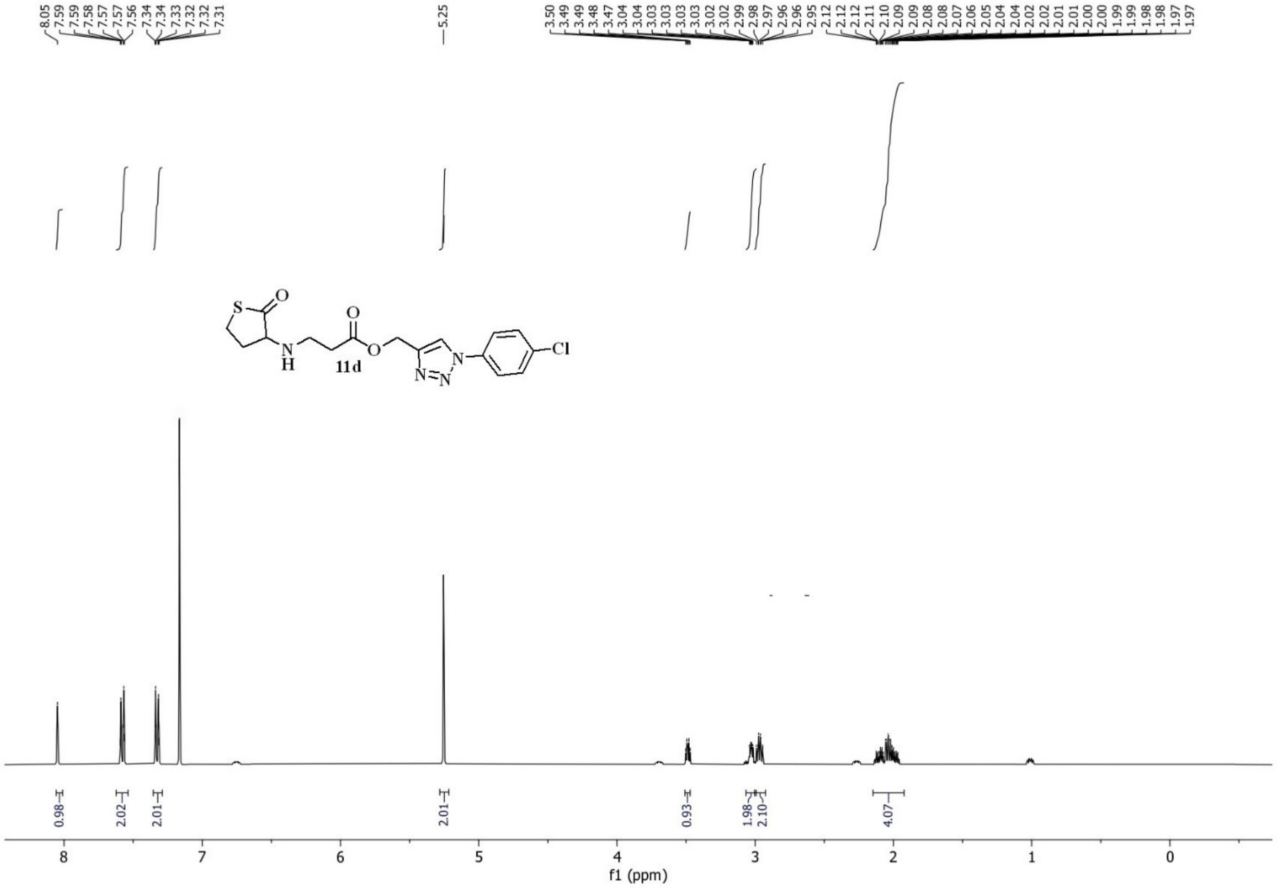

^{1}H-NMR spectrum of 11d (400 MHz, $CDCl_3$)

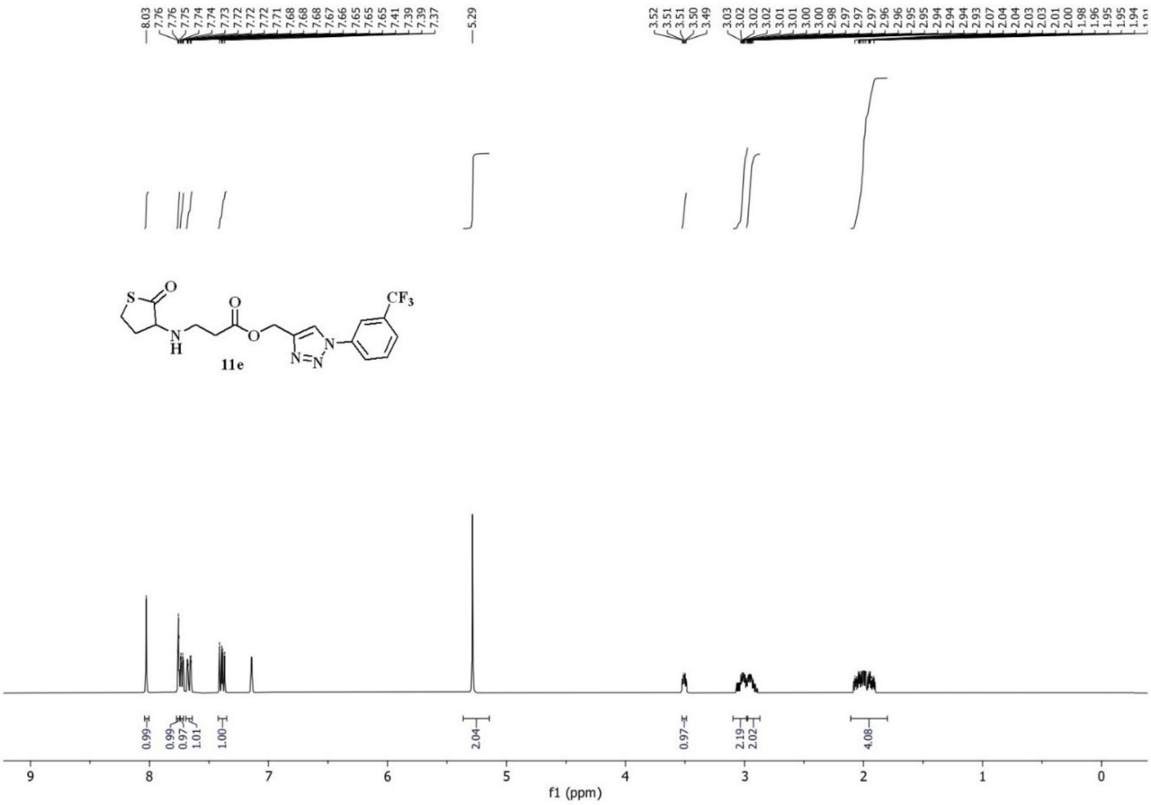

^{1}H-NMR spectrum of 11e (400 MHz, $CDCl_3$)

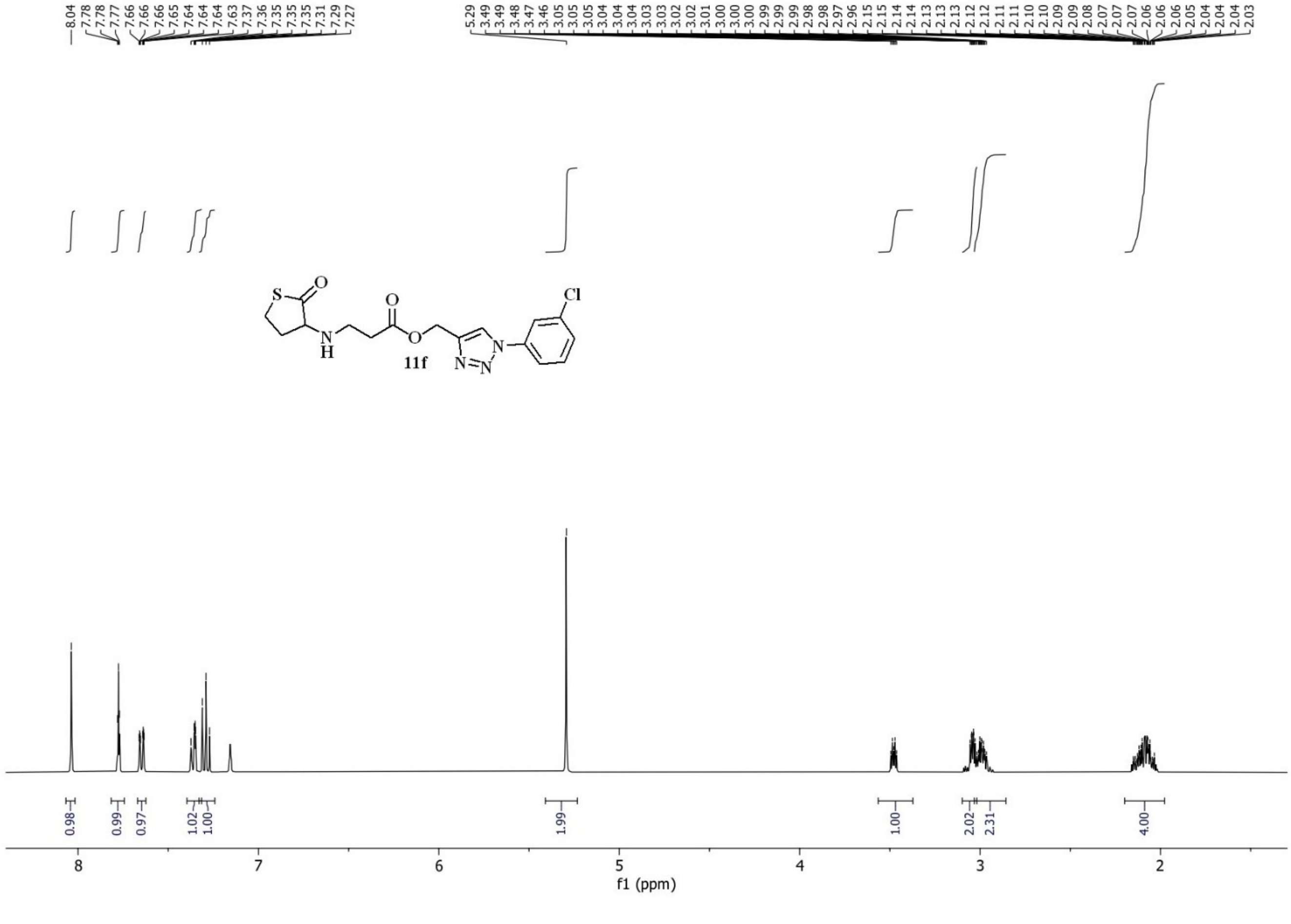

^{1}H-NMR spectrum of 11f (400 MHz, $CDCl_3$)

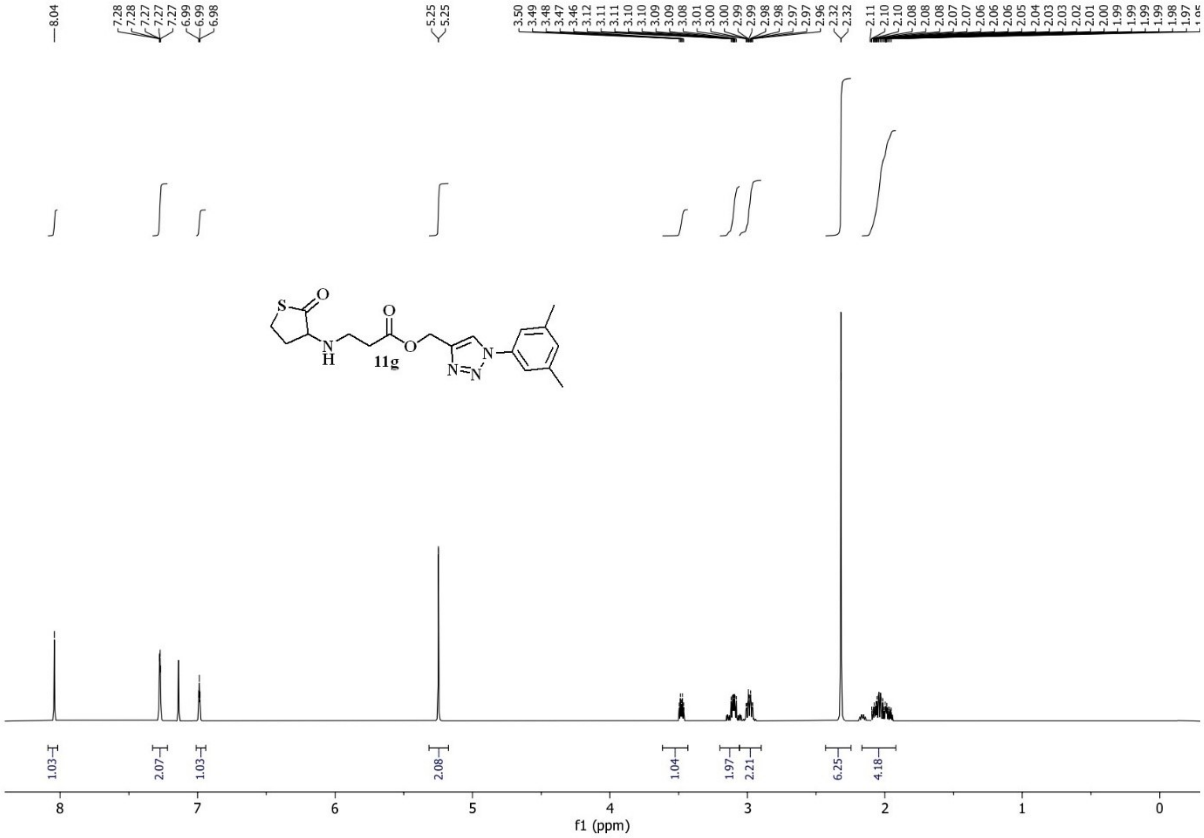

^{1}H-NMR spectrum of 11g (400 MHz, $CDCl_3$)

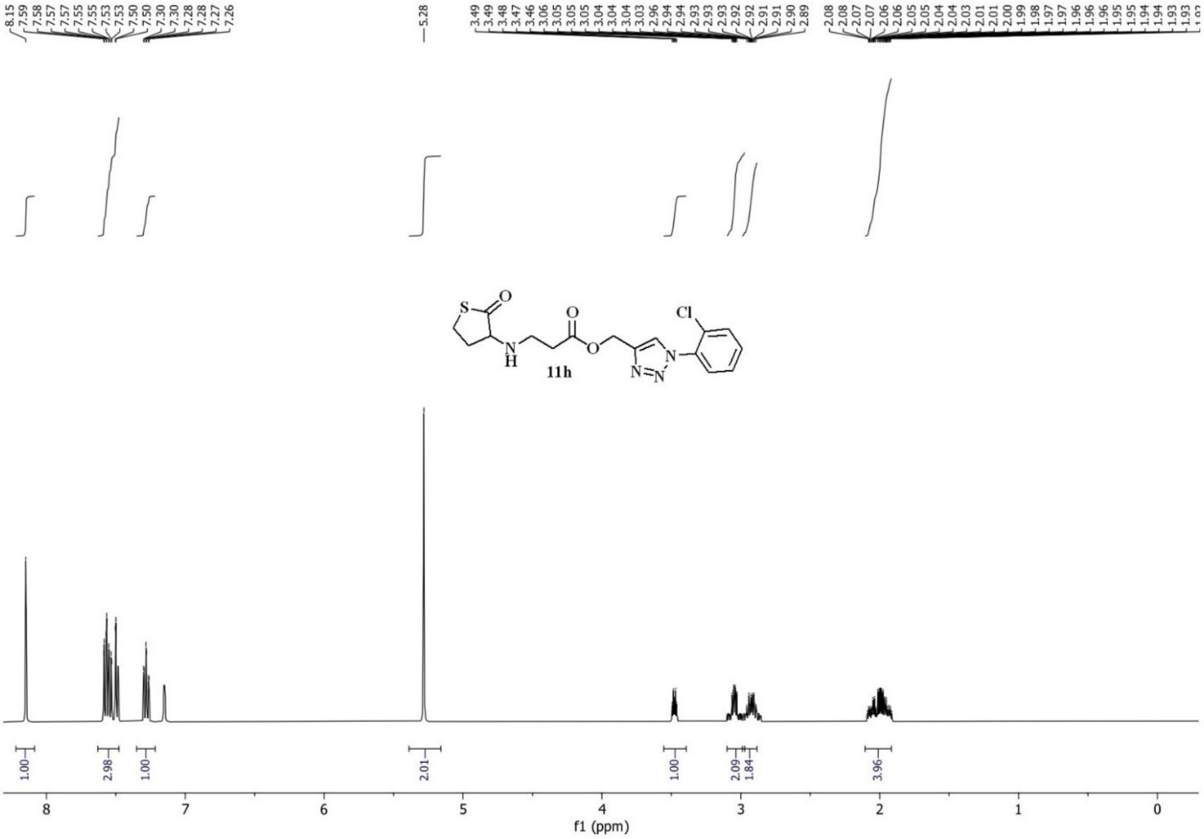

^{1}H-NMR spectrum of 11h (400 MHz, $CDCl_3$)

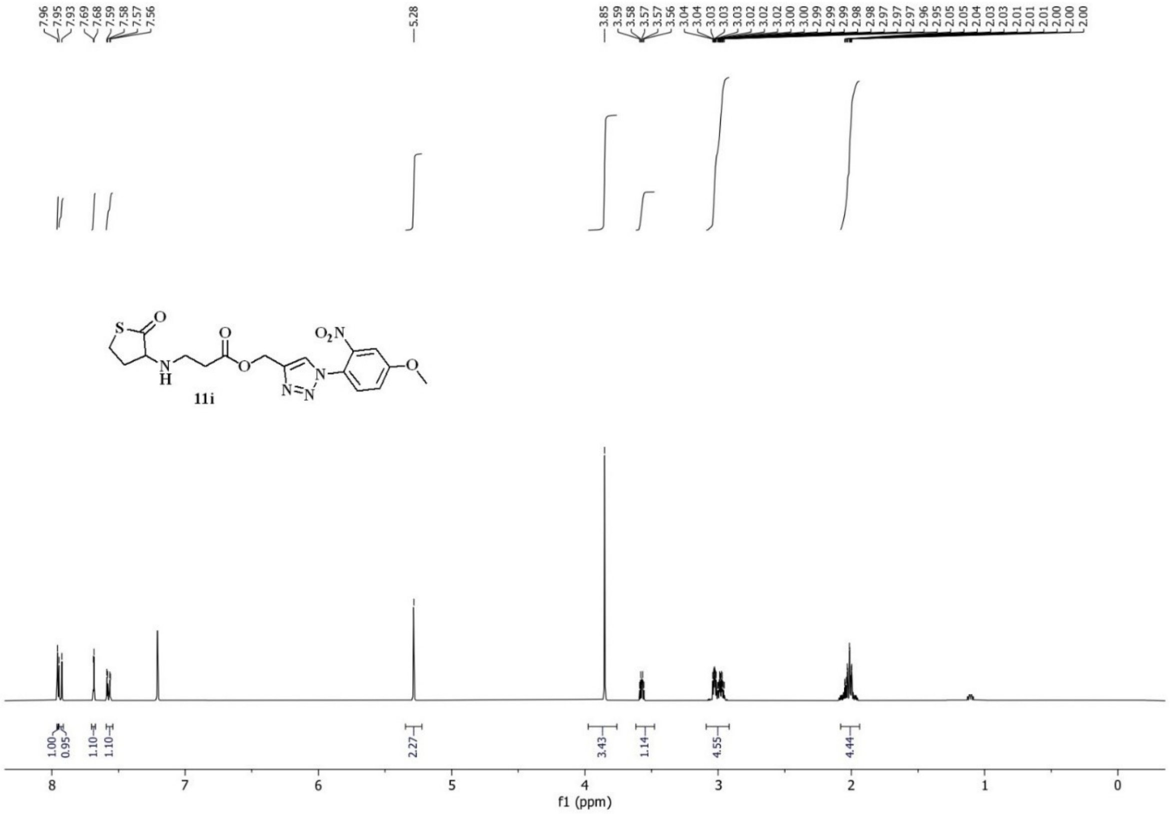

^{1}H-NMR spectrum of 11i (400 MHz, $CDCl_3$)

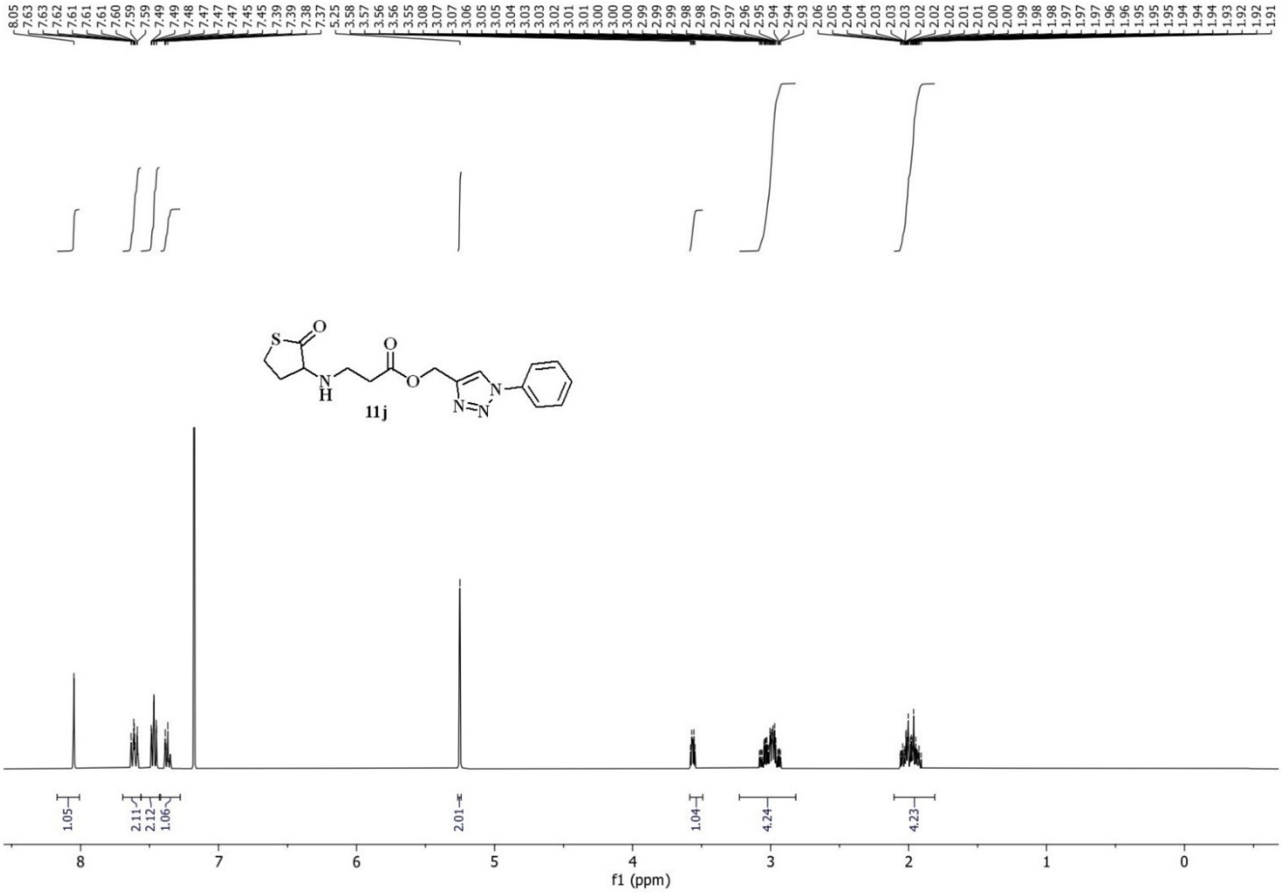

^{1}H-NMR spectrum of 11j (400 MHz, $CDCl_3$)

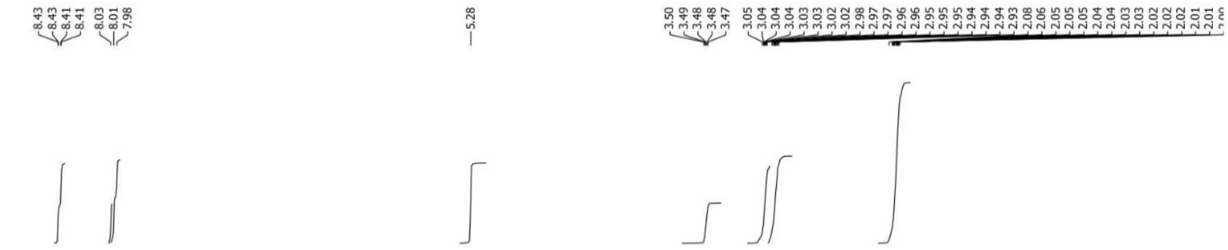

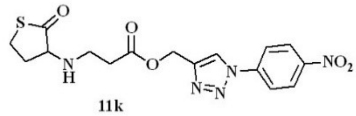

11k

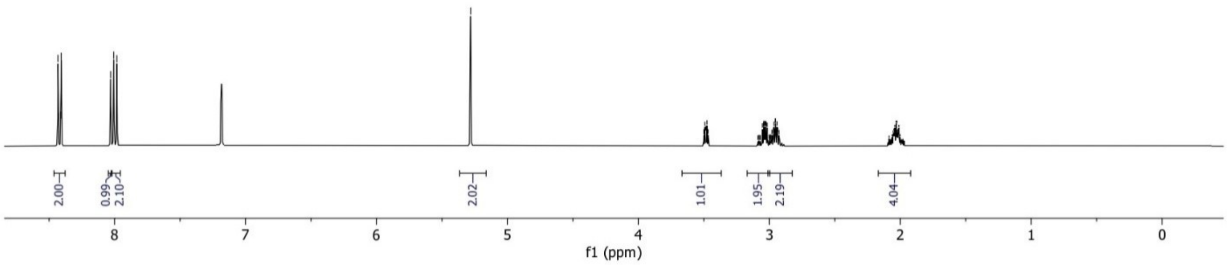

^{1}H-NMR spectrum of 11k (400 MHz, $CDCl_3$)

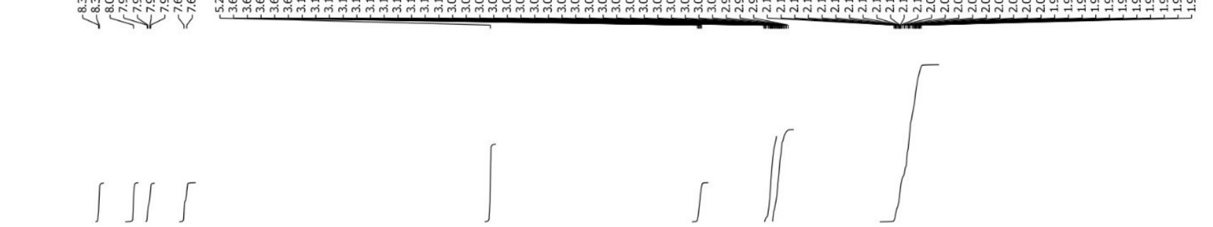

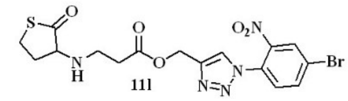

11l

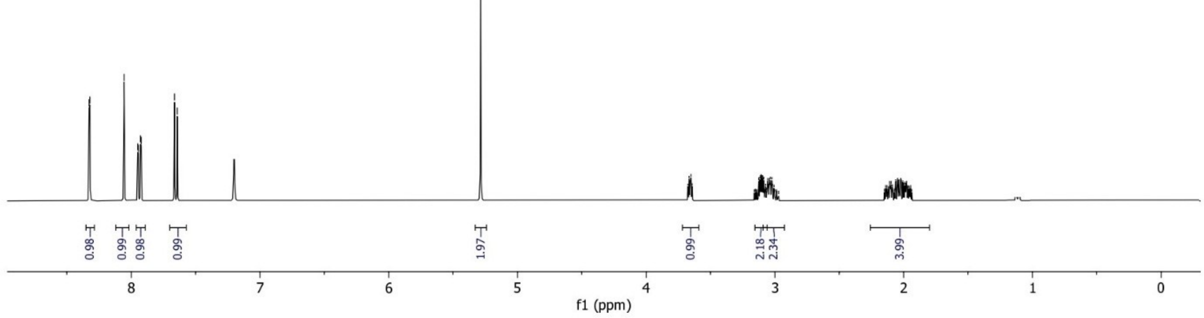

^{1}H-NMR spectrum of 11l (400 MHz, $CDCl_3$)

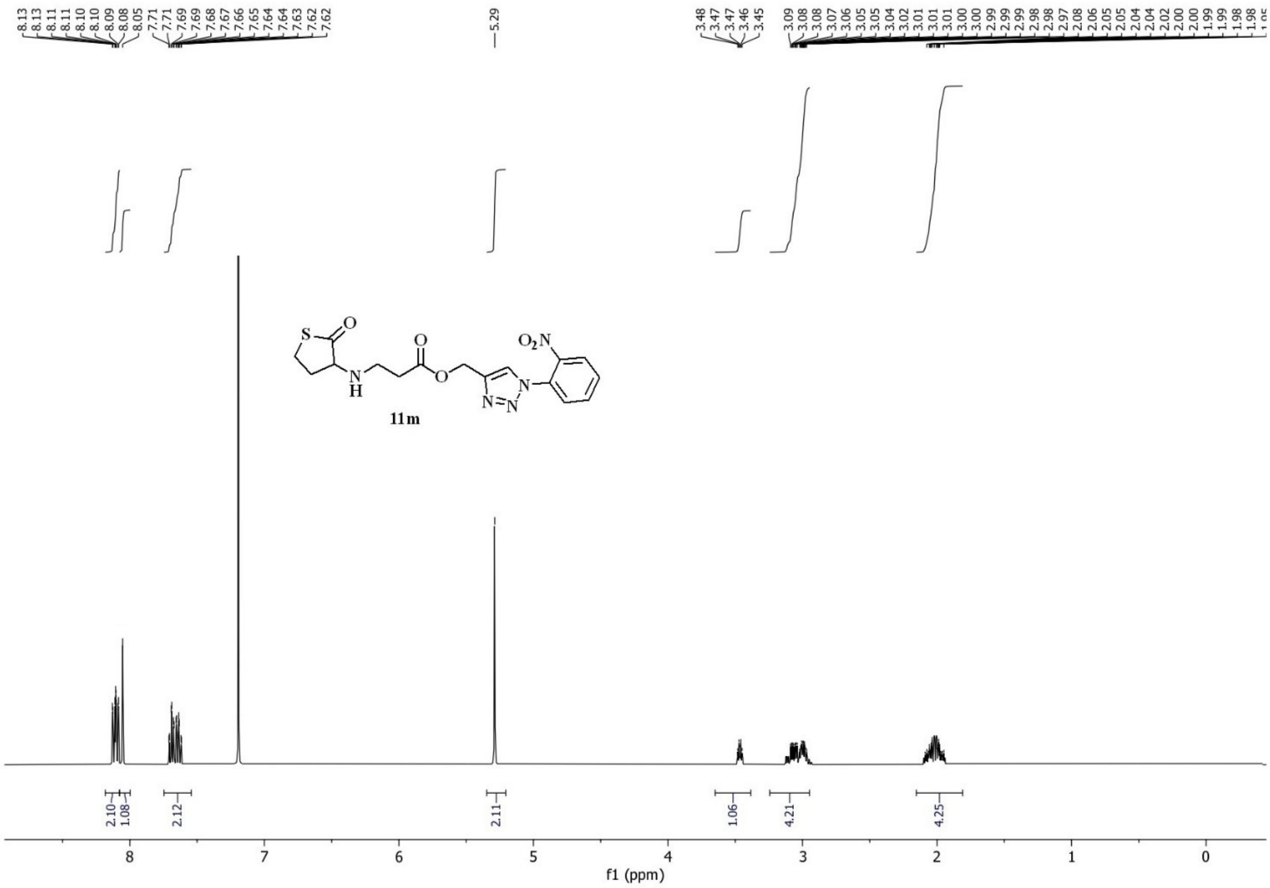

^{1}H-NMR spectrum of 11m (400 MHz, $CDCl_3$)

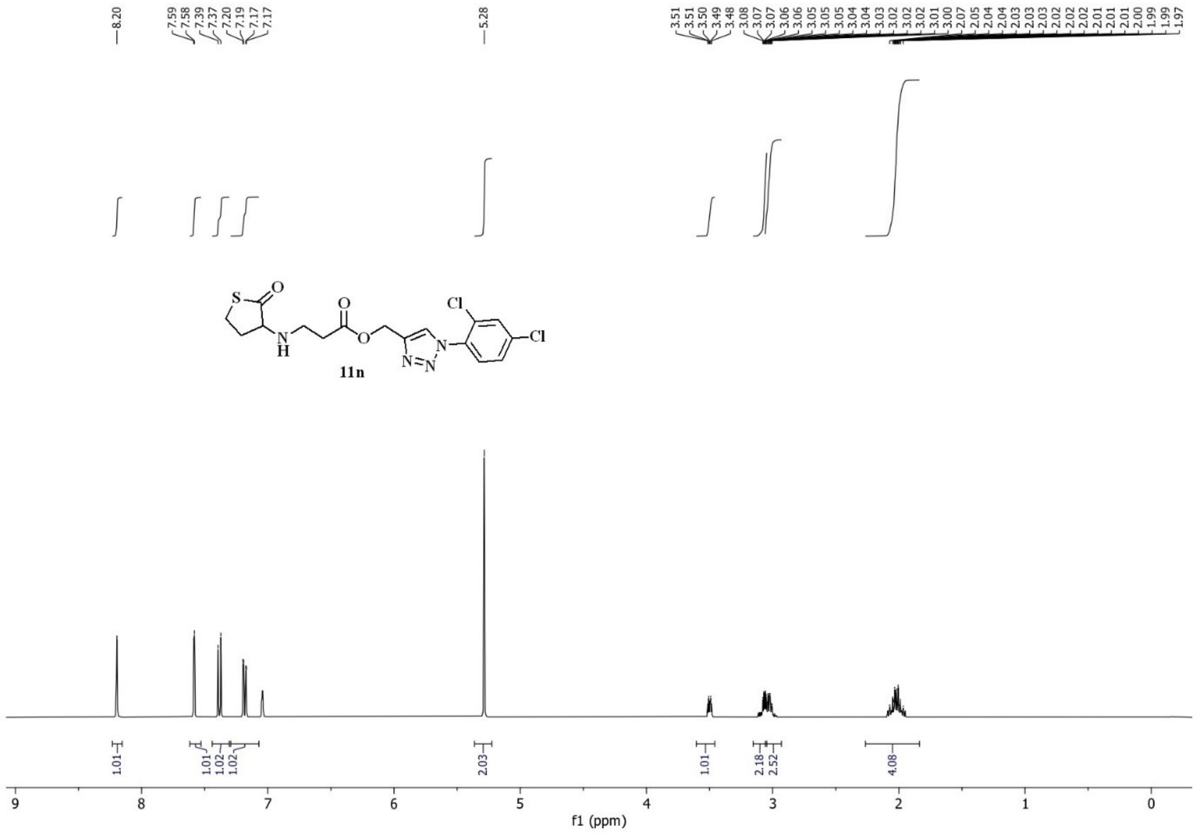

^{1}H-NMR spectrum of 11n (400 MHz, $CDCl_3$)

^{13}C-NMR spectrum of final compounds (10a-p, 11a-n):

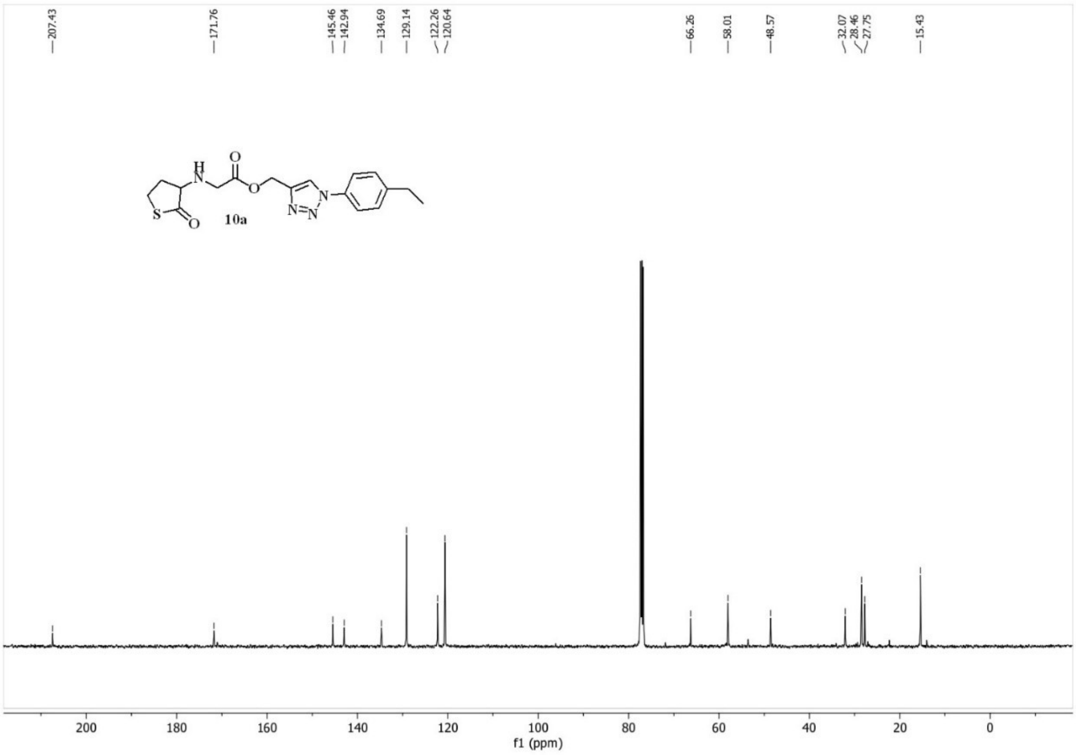

^{13}C-NMR spectrum of 10a (101 MHz, $CDCl_3$)

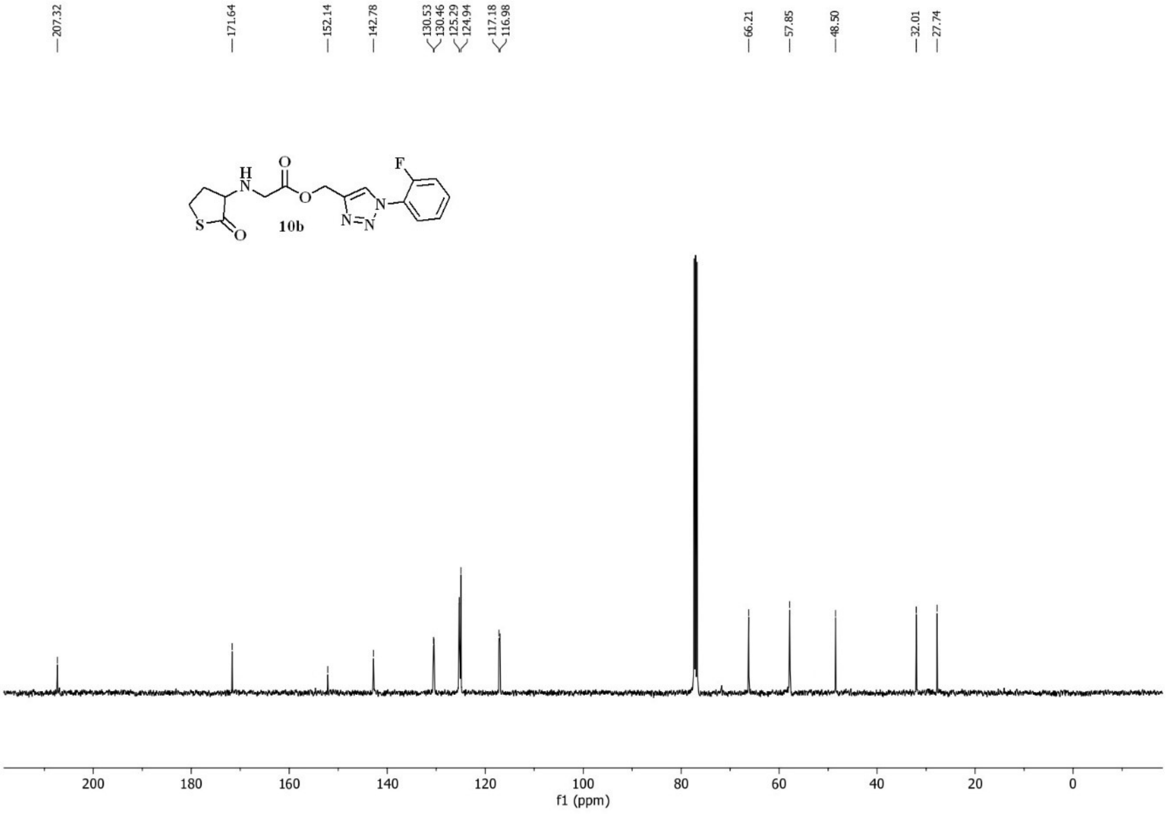

^{13}C-NMR spectrum of 10b (101 MHz, $CDCl_3$)

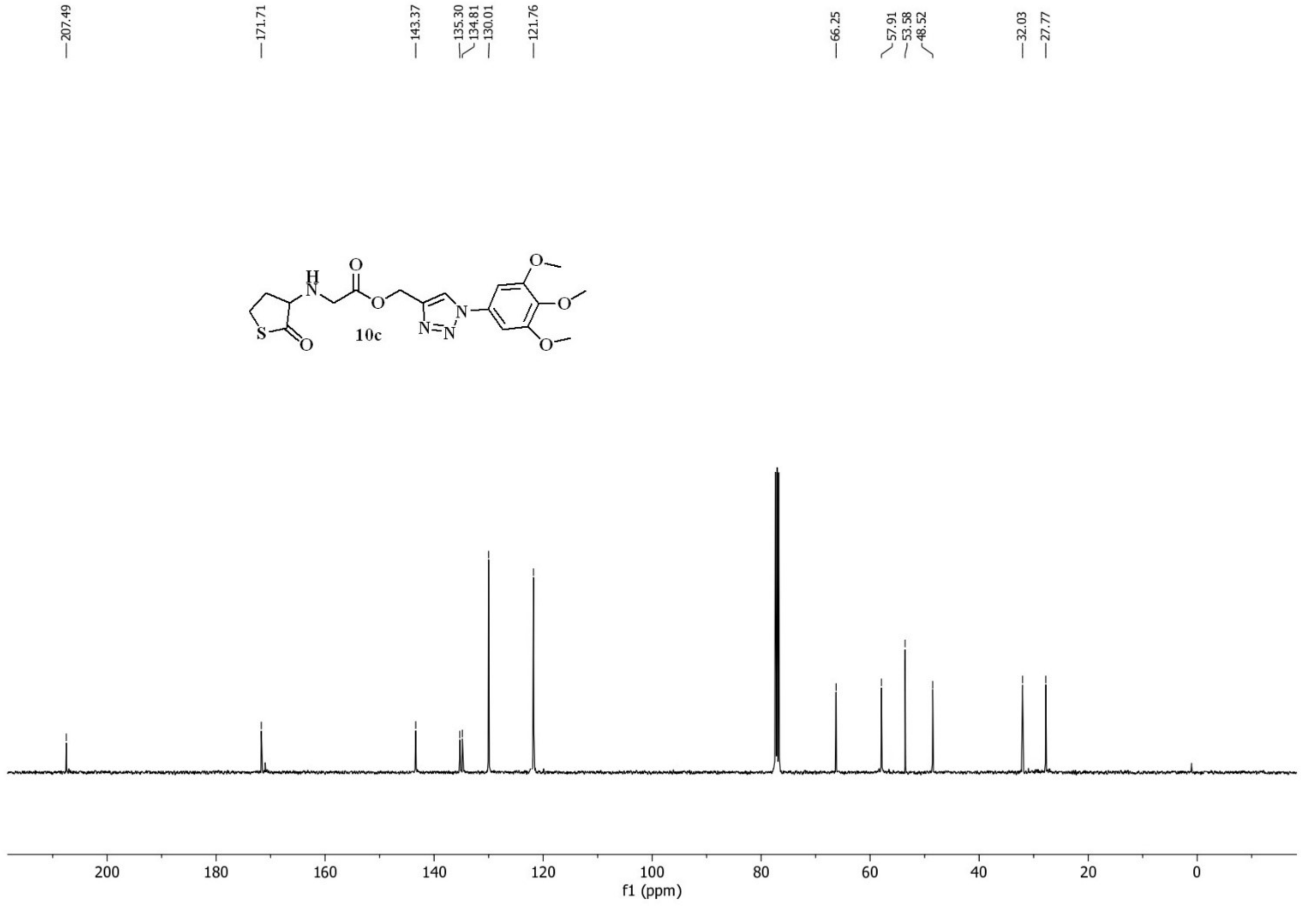

^{13}C-NMR spectrum of 10c (101 MHz, $CDCl_3$)

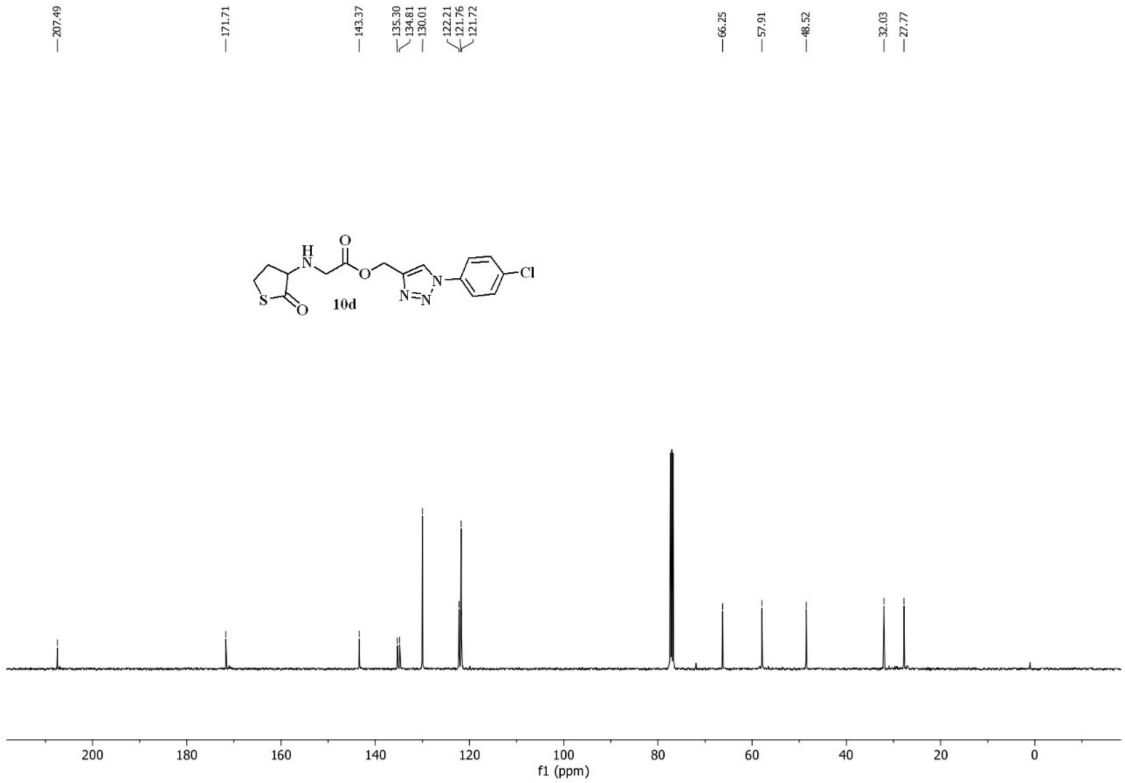

^{13}C-NMR spectrum of 10d (101 MHz, $CDCl_3$)

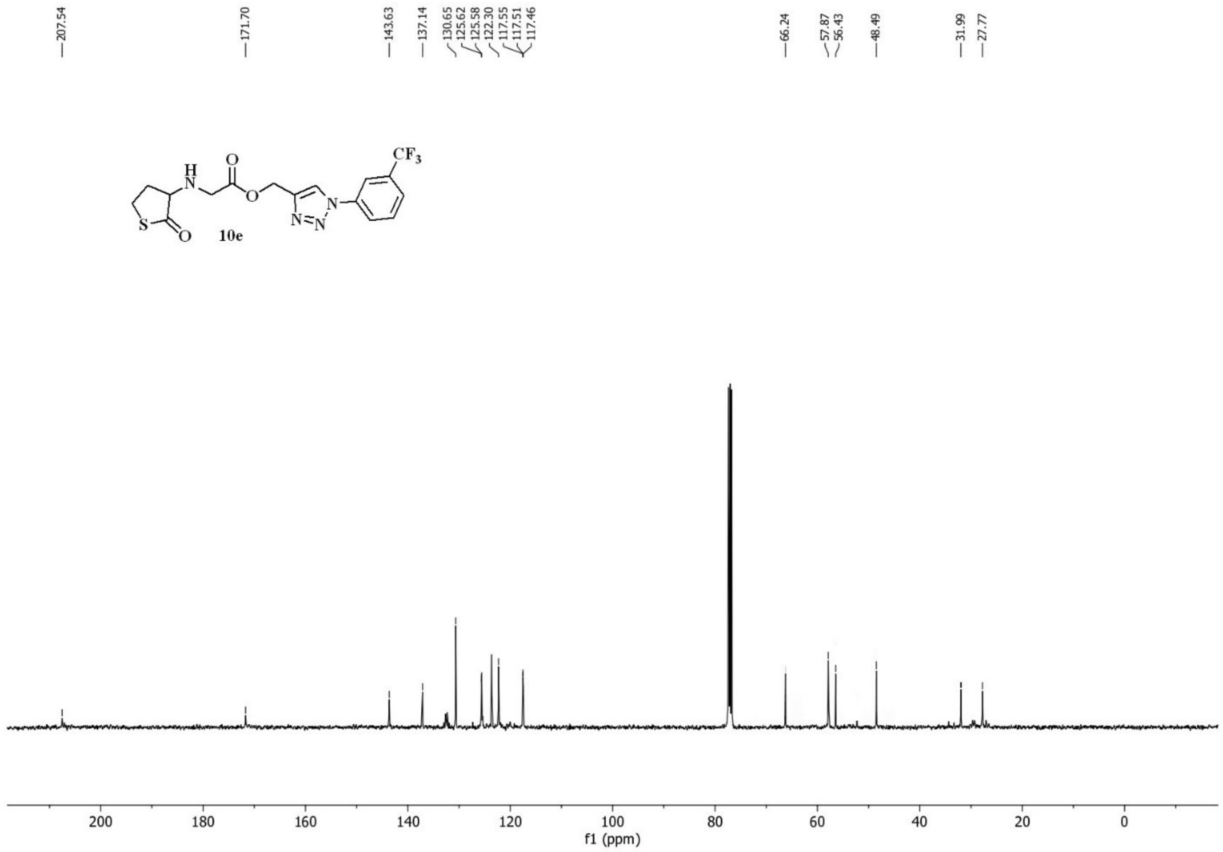

^{13}C-NMR spectrum of 10e (101 MHz, $CDCl_3$)

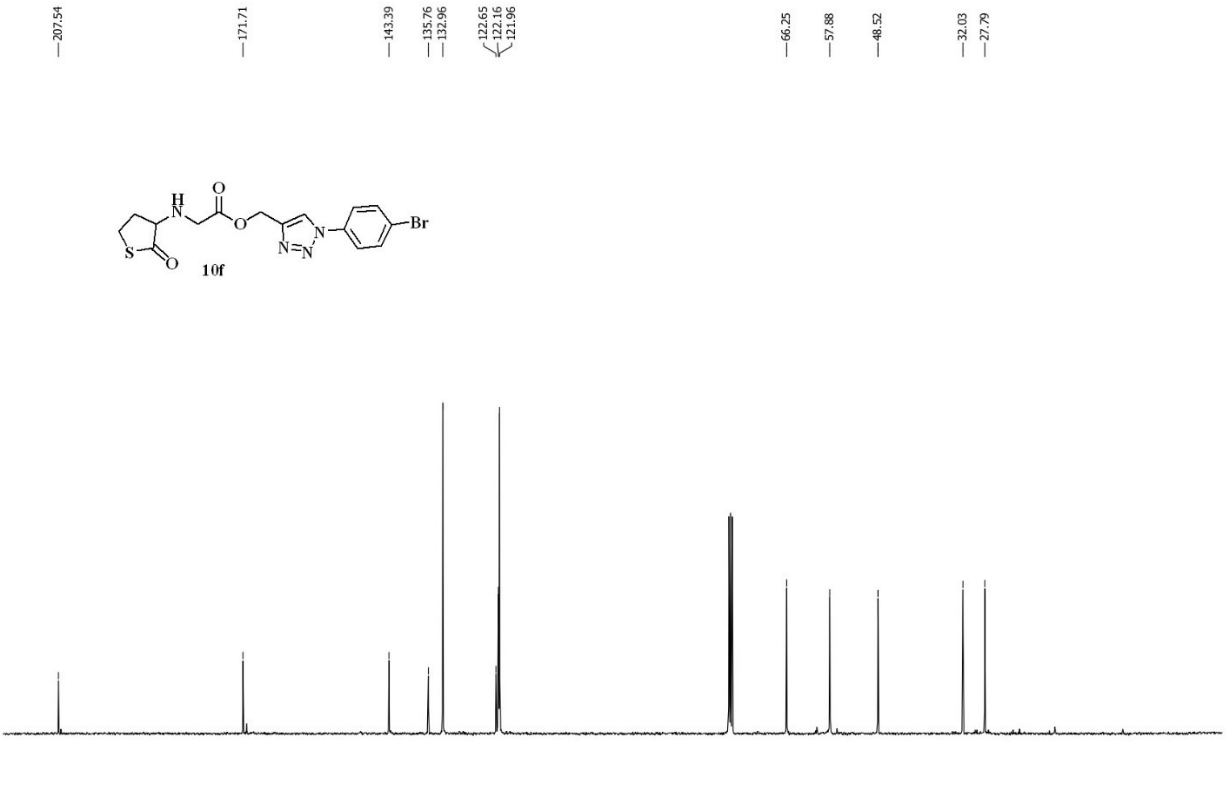

^{13}C-NMR spectrum of 10f (101 MHz, $CDCl_3$)

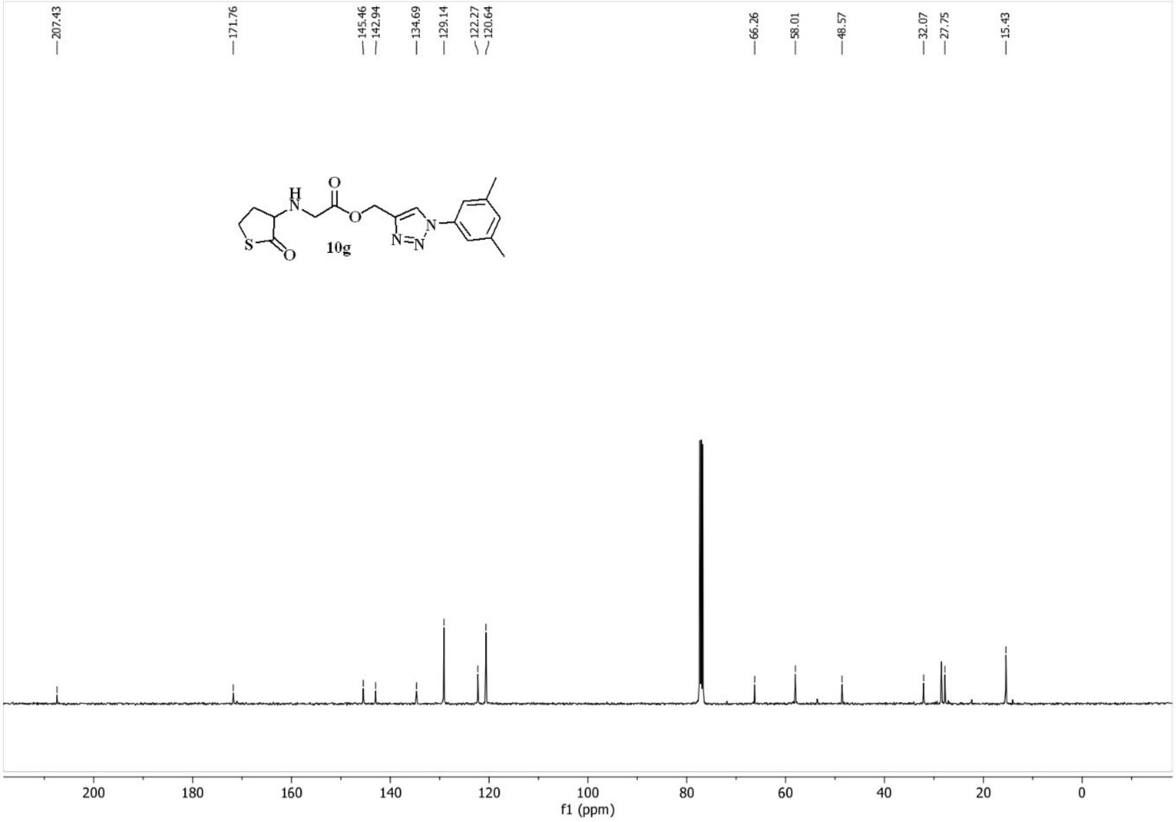

^{13}C-NMR spectrum of 10g (101 MHz, $CDCl_3$)

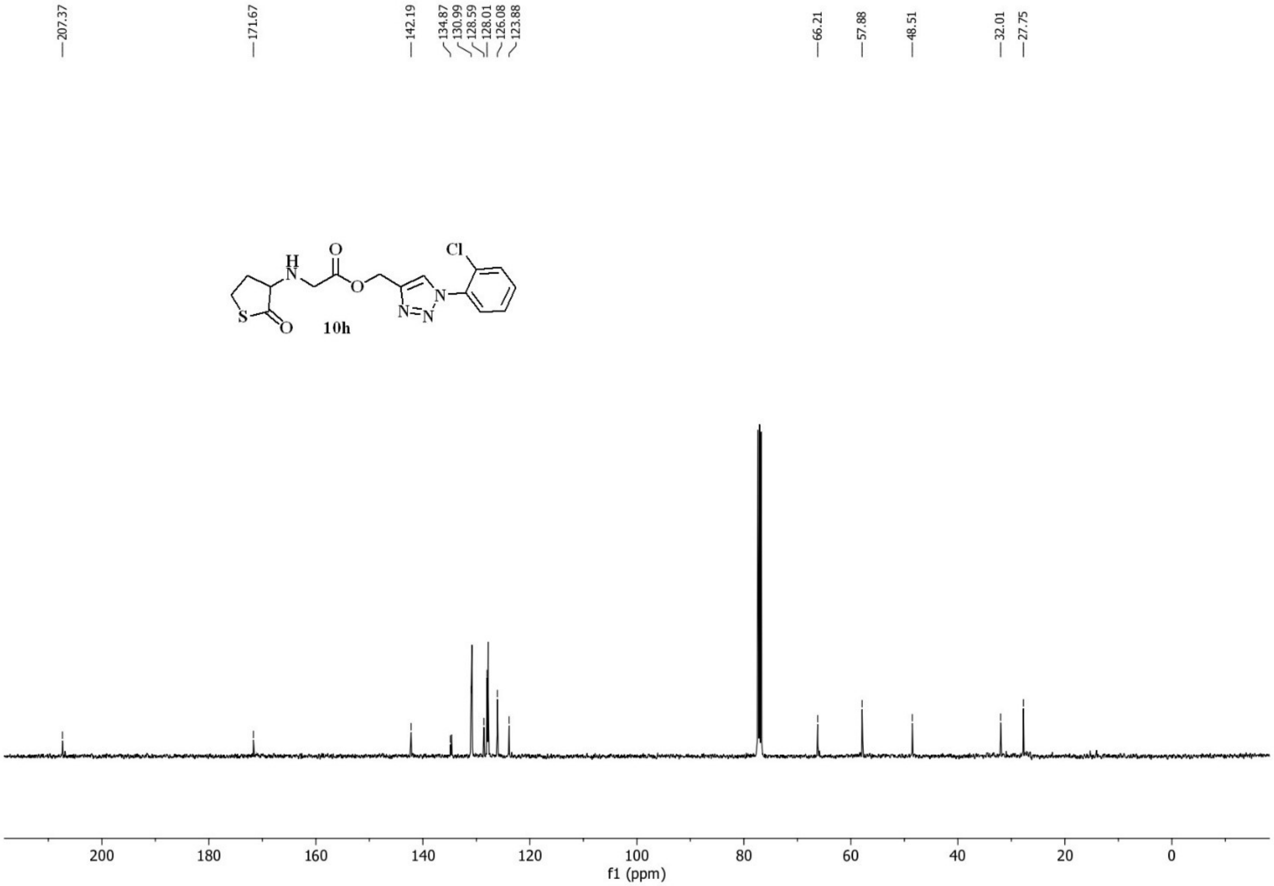

^{13}C-NMR spectrum of 10h (101 MHz, $CDCl_3$)

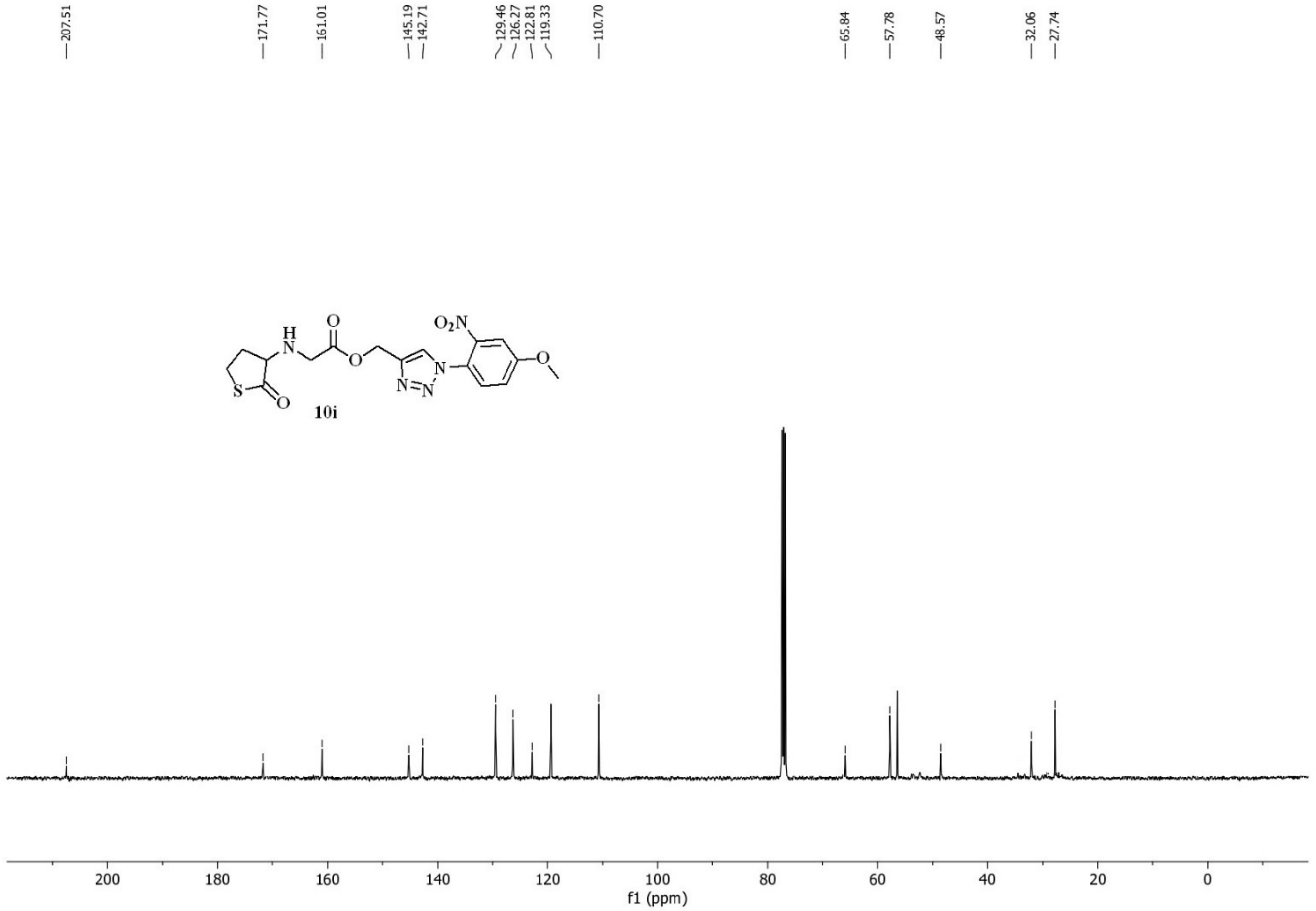

^{13}C-NMR spectrum of 10i (101 MHz, $CDCl_3$)

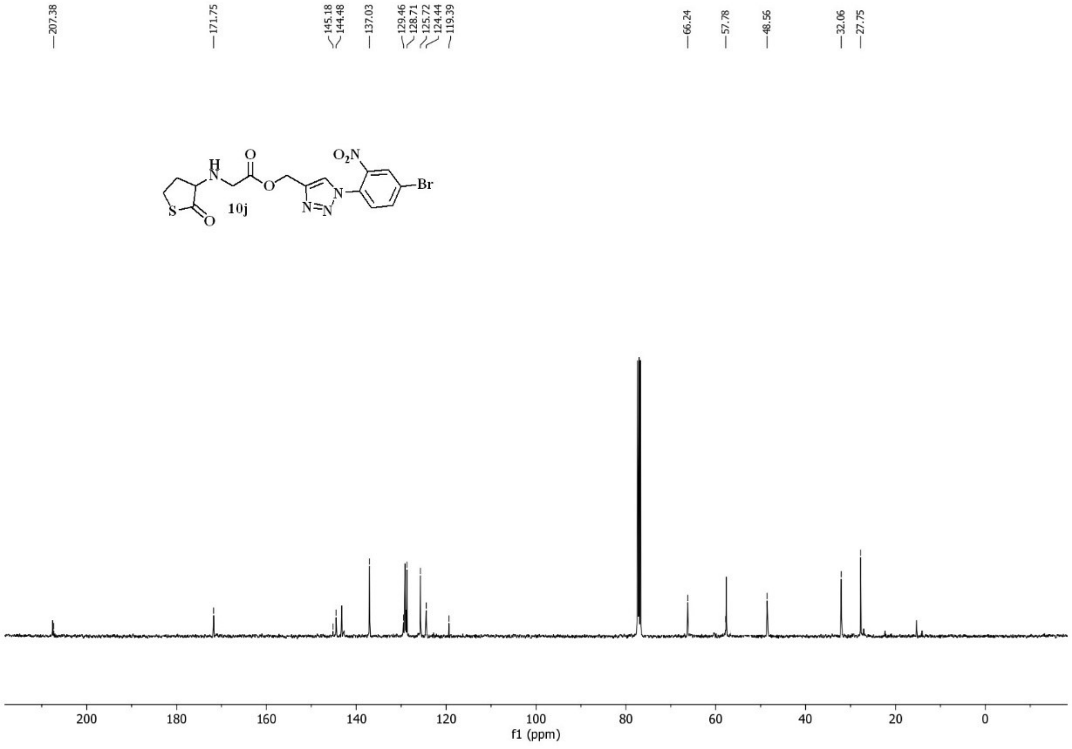

^{13}C-NMR spectrum of 10j (101 MHz, $CDCl_3$)

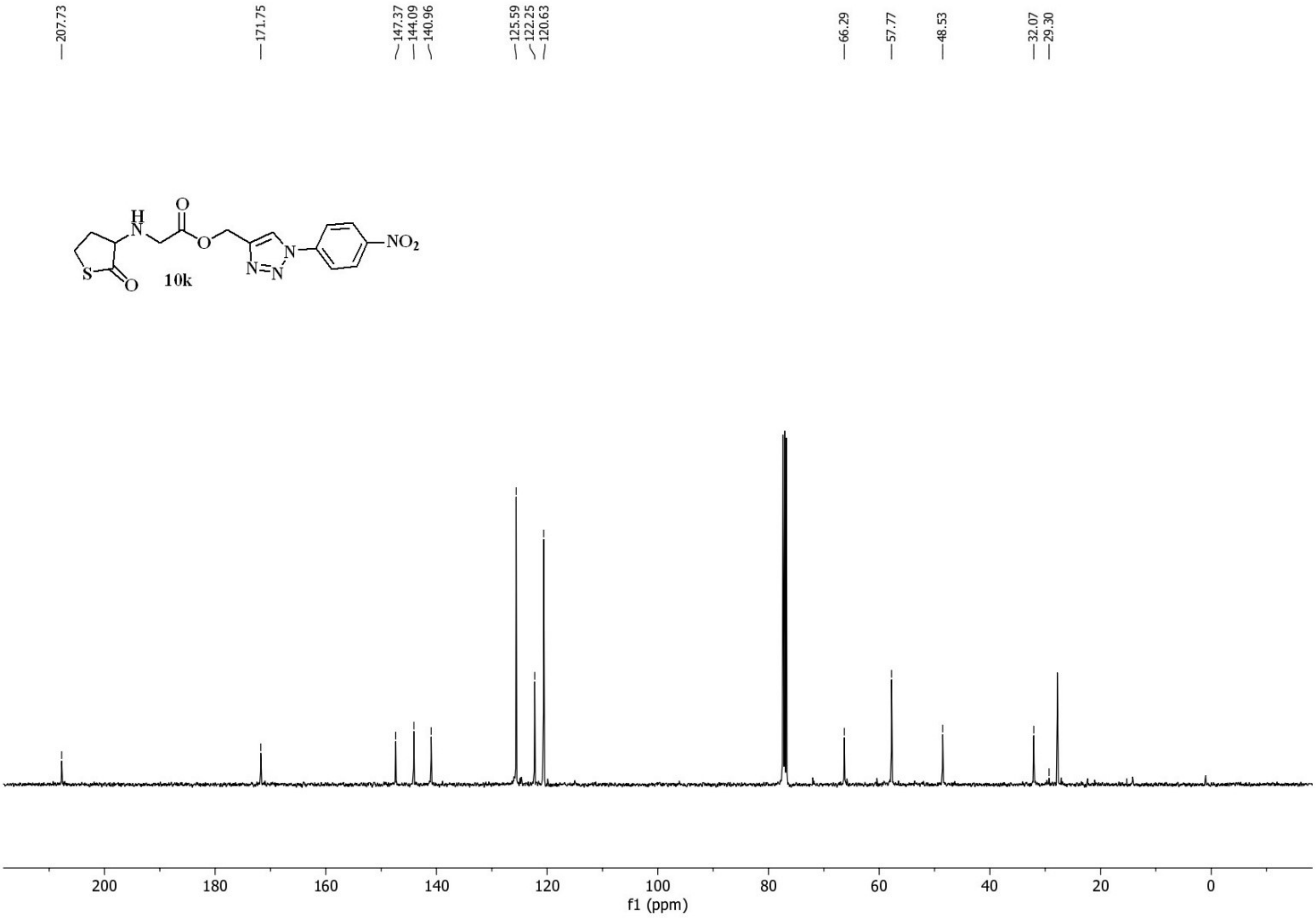

^{13}C-NMR spectrum of 10k (101 MHz, CDCl$_3$)

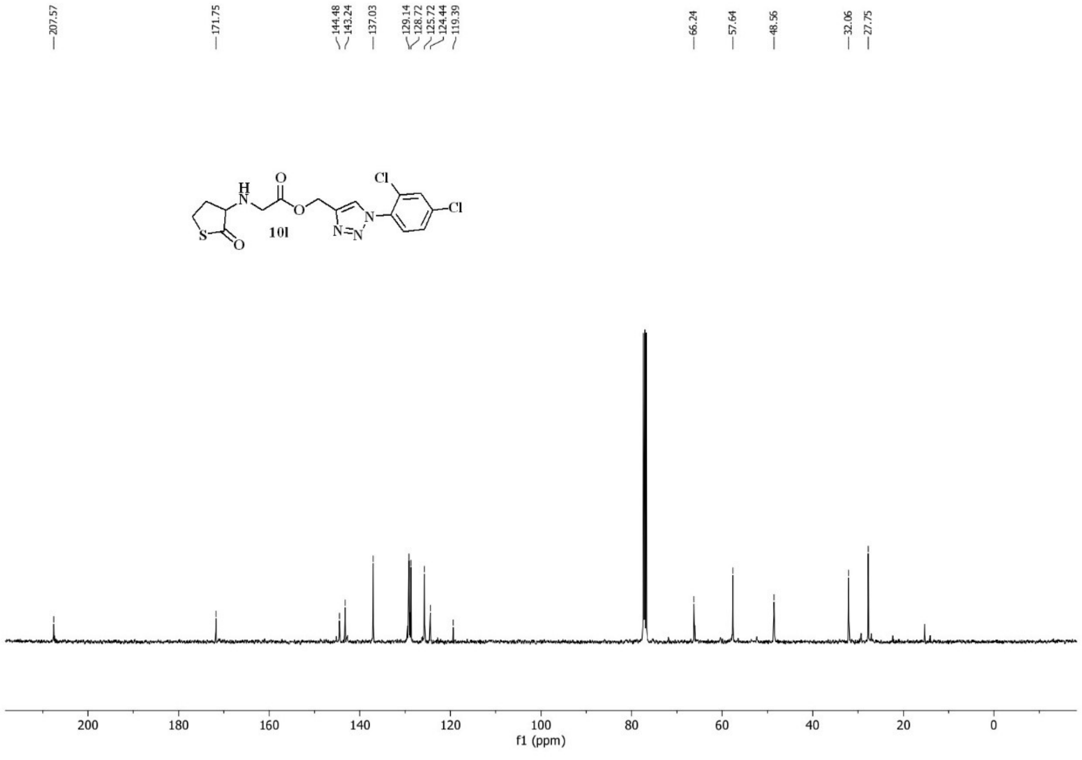

^{13}C-NMR spectrum of 10l (101 MHz, CDCl$_3$)

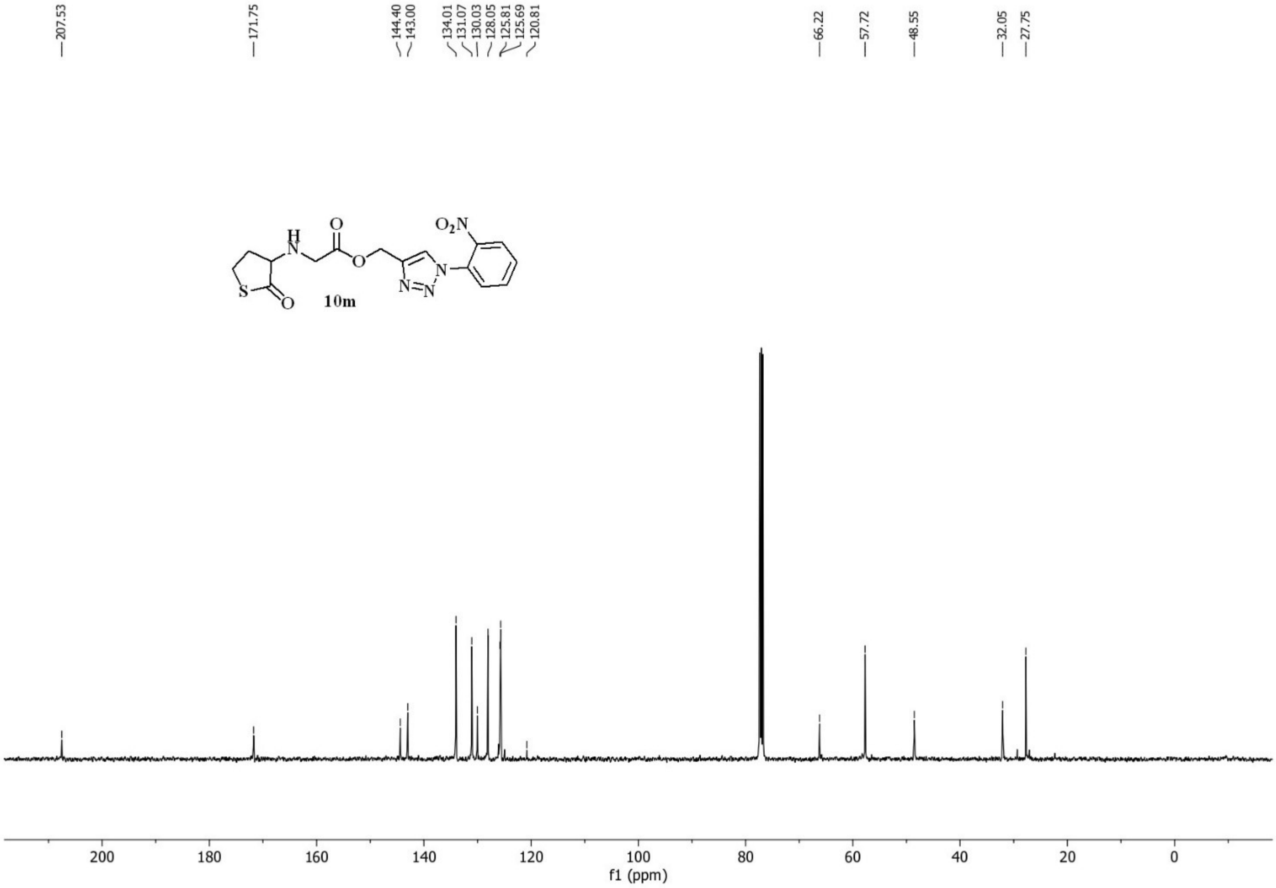

^{13}C-NMR spectrum of 10m (101 MHz, $CDCl_3$)

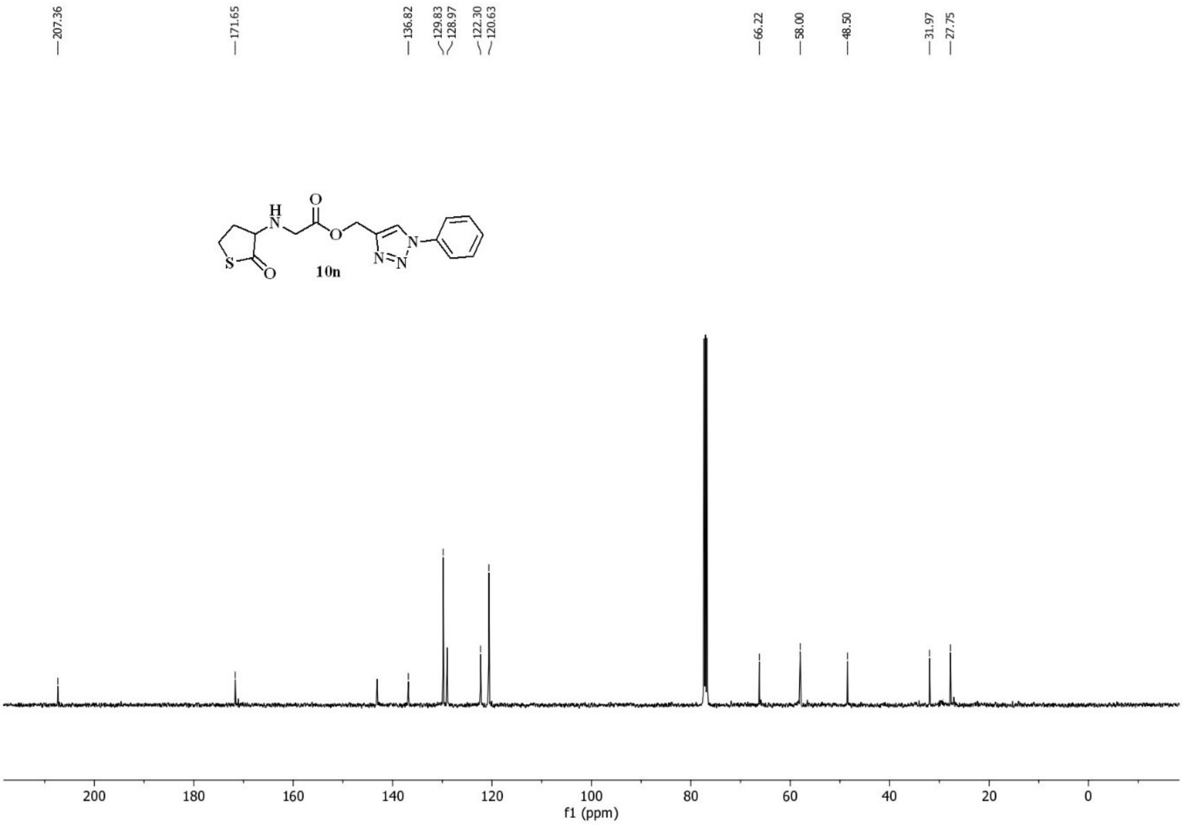

^{13}C-NMR spectrum of 10n (101 MHz, $CDCl_3$)

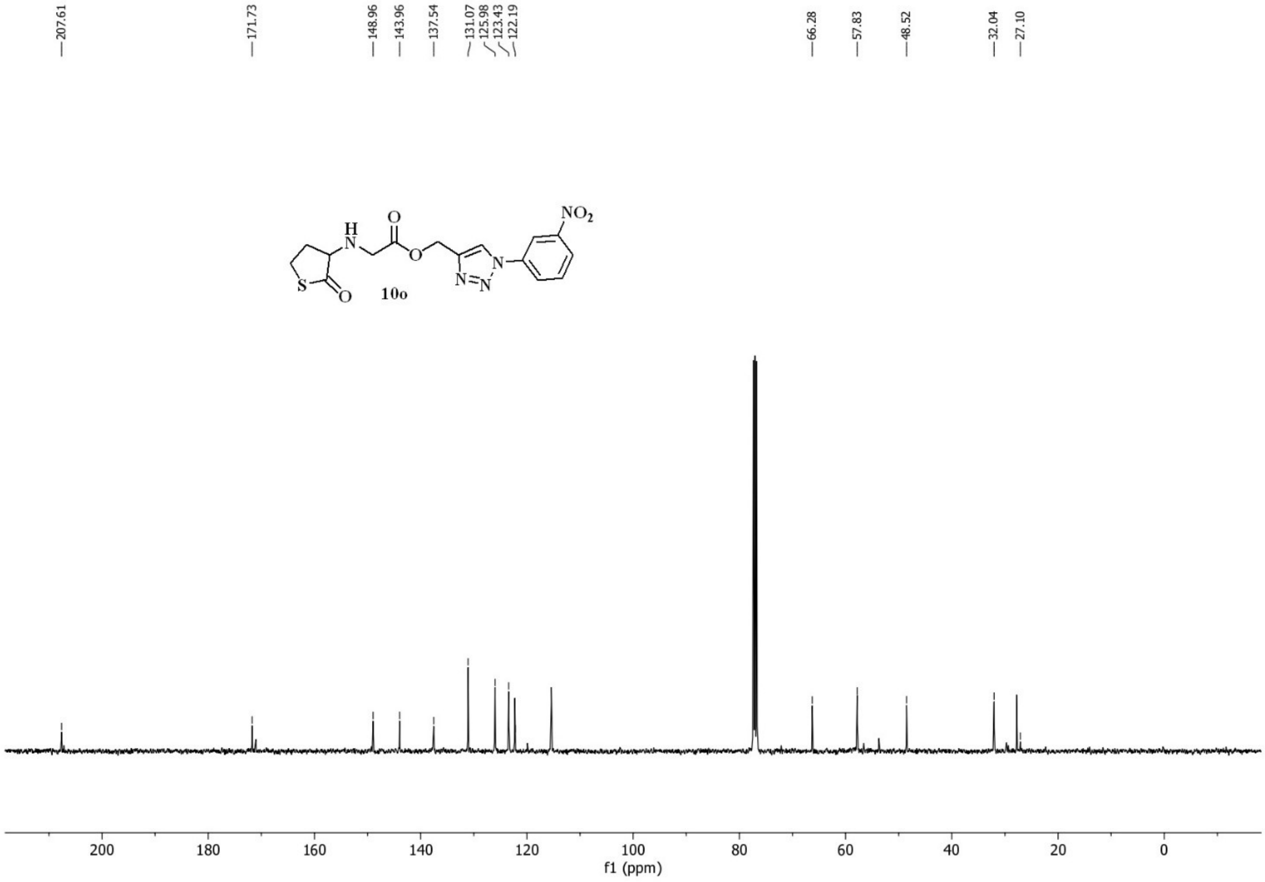

^{13}C-NMR spectrum of 10o (101 MHz, $CDCl_3$)

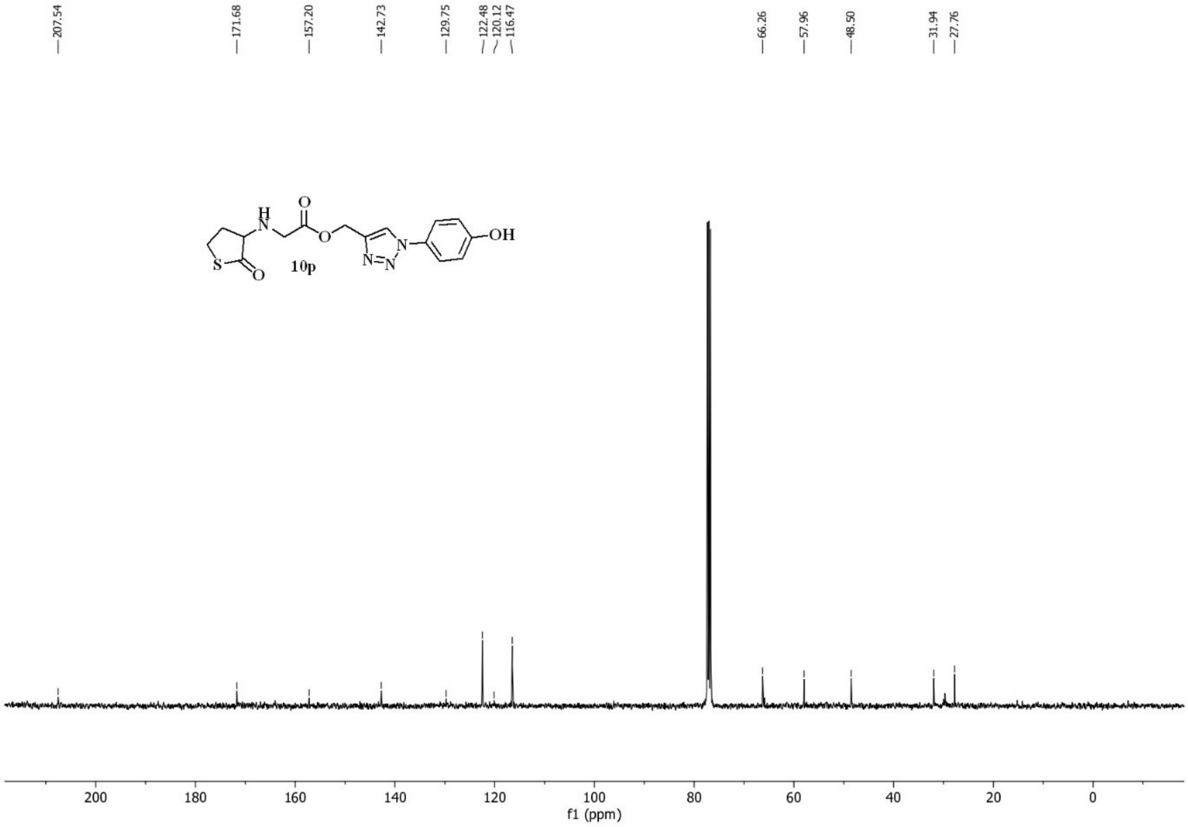

^{13}C-NMR spectrum of 10p (101 MHz, $CDCl_3$)

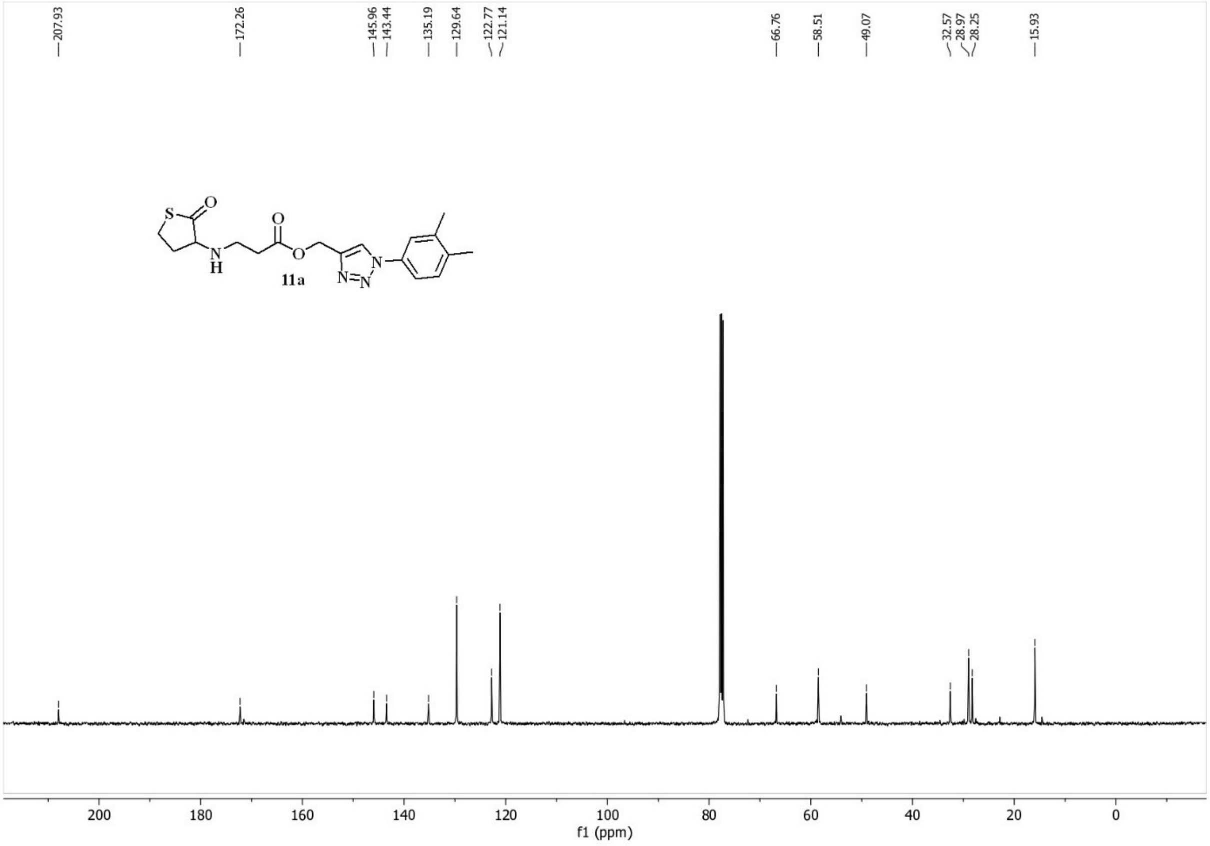

^{13}C-NMR spectrum of 11a (101 MHz, $CDCl_3$)

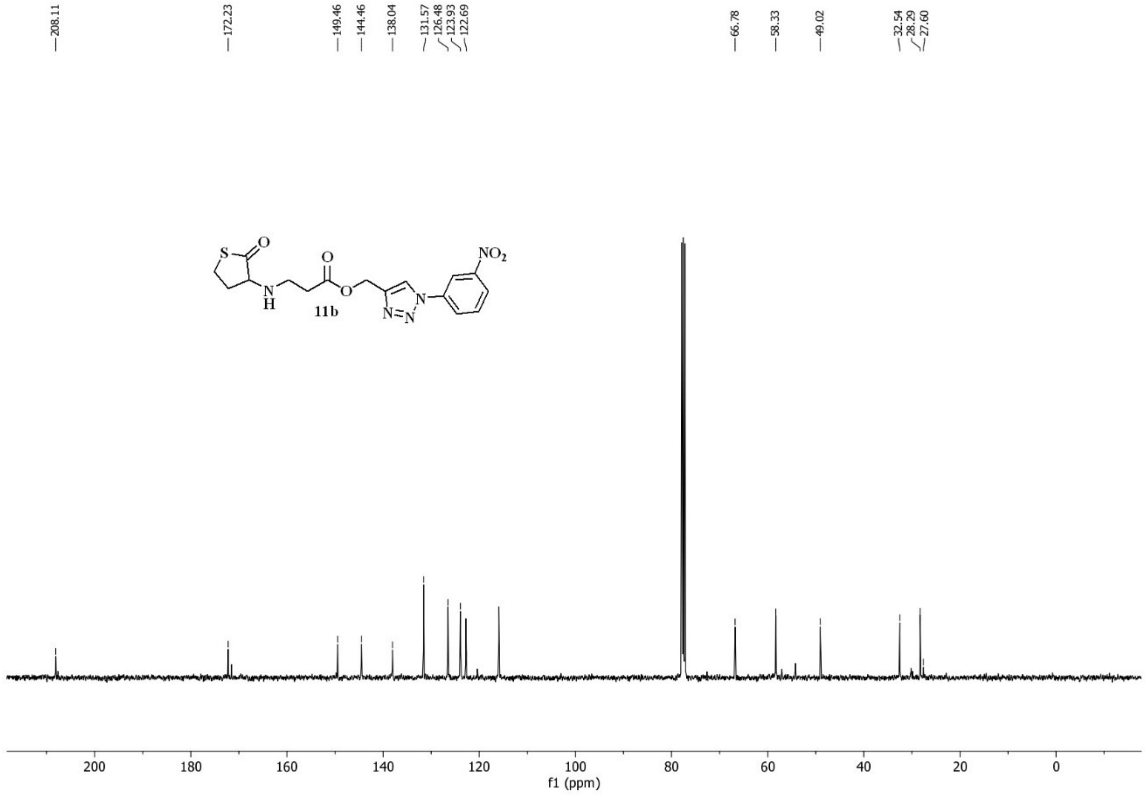

^{13}C-NMR spectrum of 11b (101 MHz, $CDCl_3$)

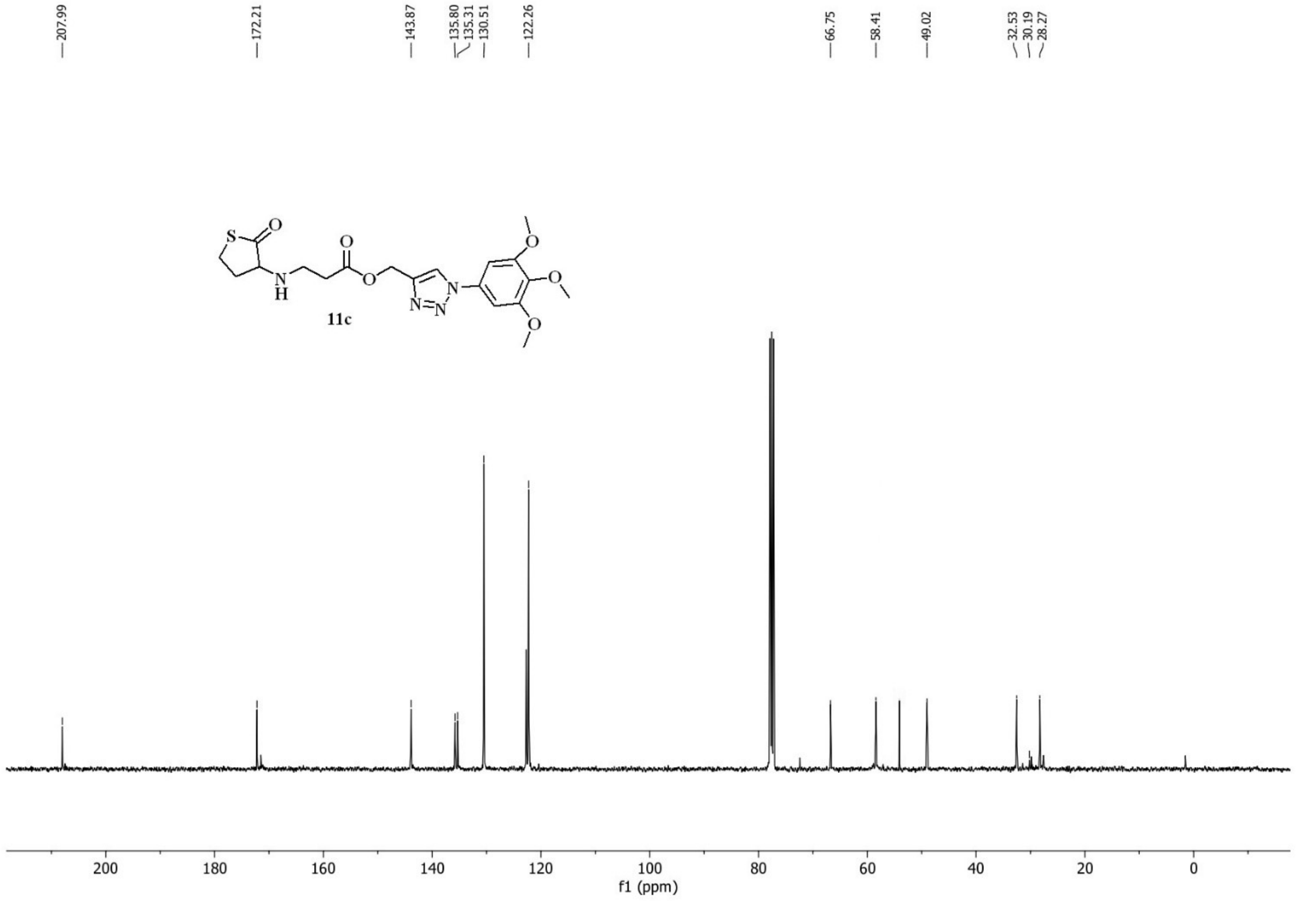

^{13}C-NMR spectrum of 11c (101 MHz, $CDCl_3$)

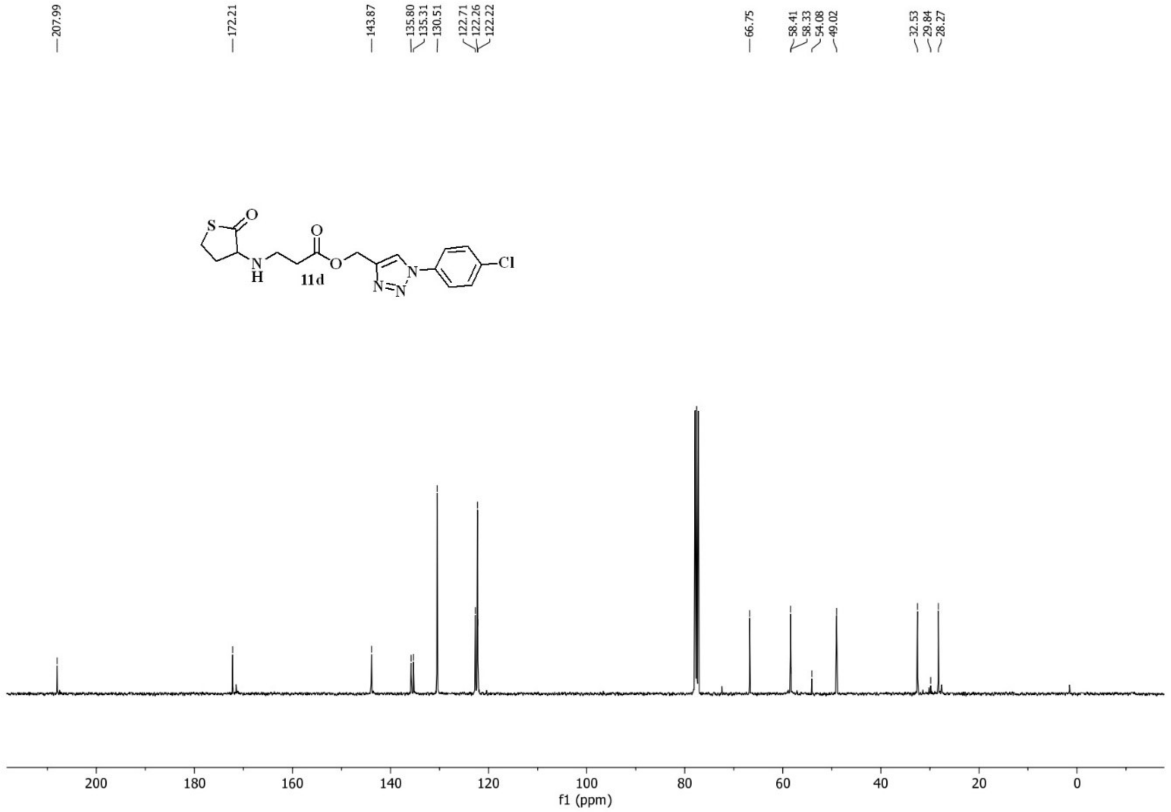

^{13}C-NMR spectrum of 11d (101 MHz, $CDCl_3$)

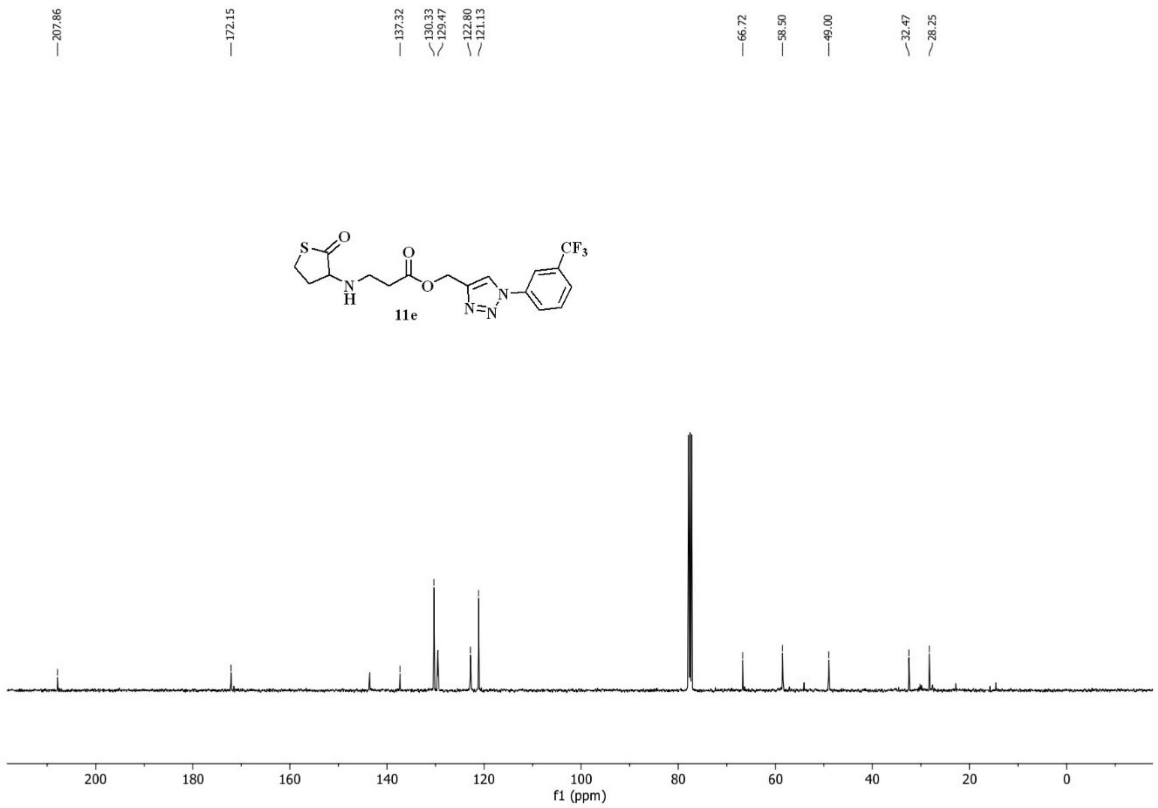

^{13}C-NMR spectrum of 11e (101 MHz, $CDCl_3$)

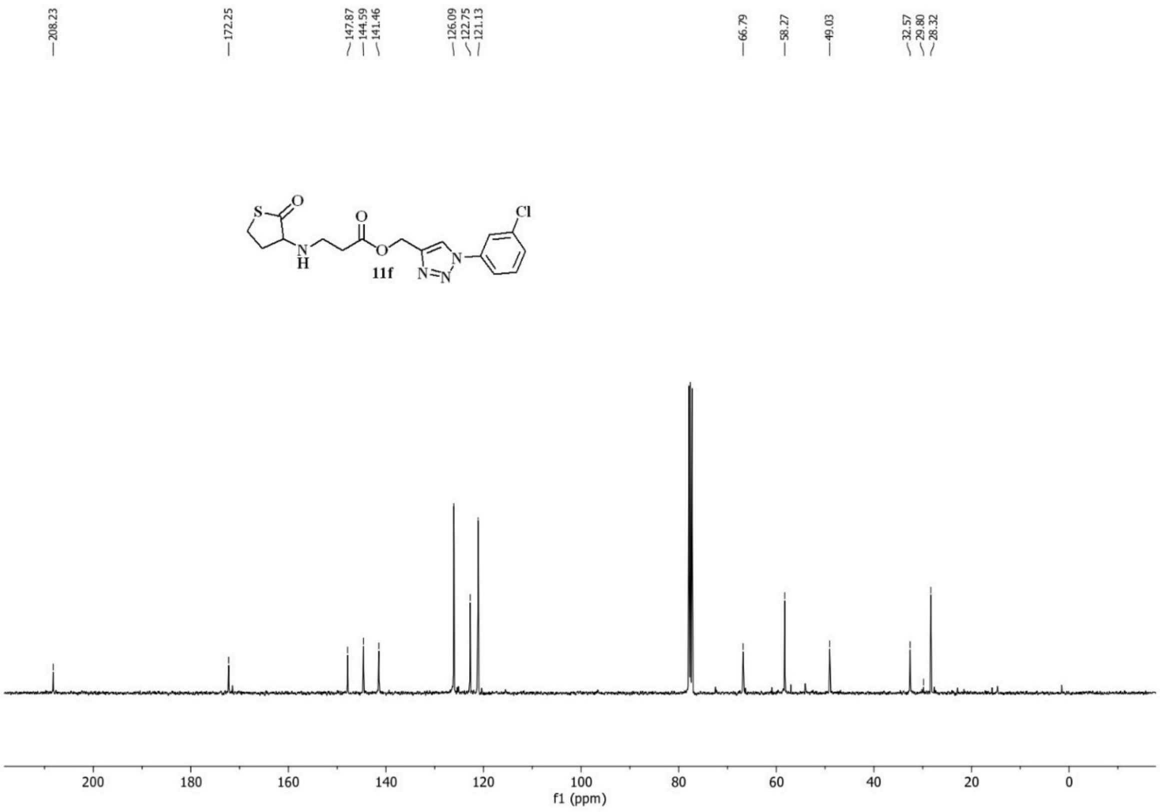

^{13}C-NMR spectrum of 11f (101 MHz, $CDCl_3$)

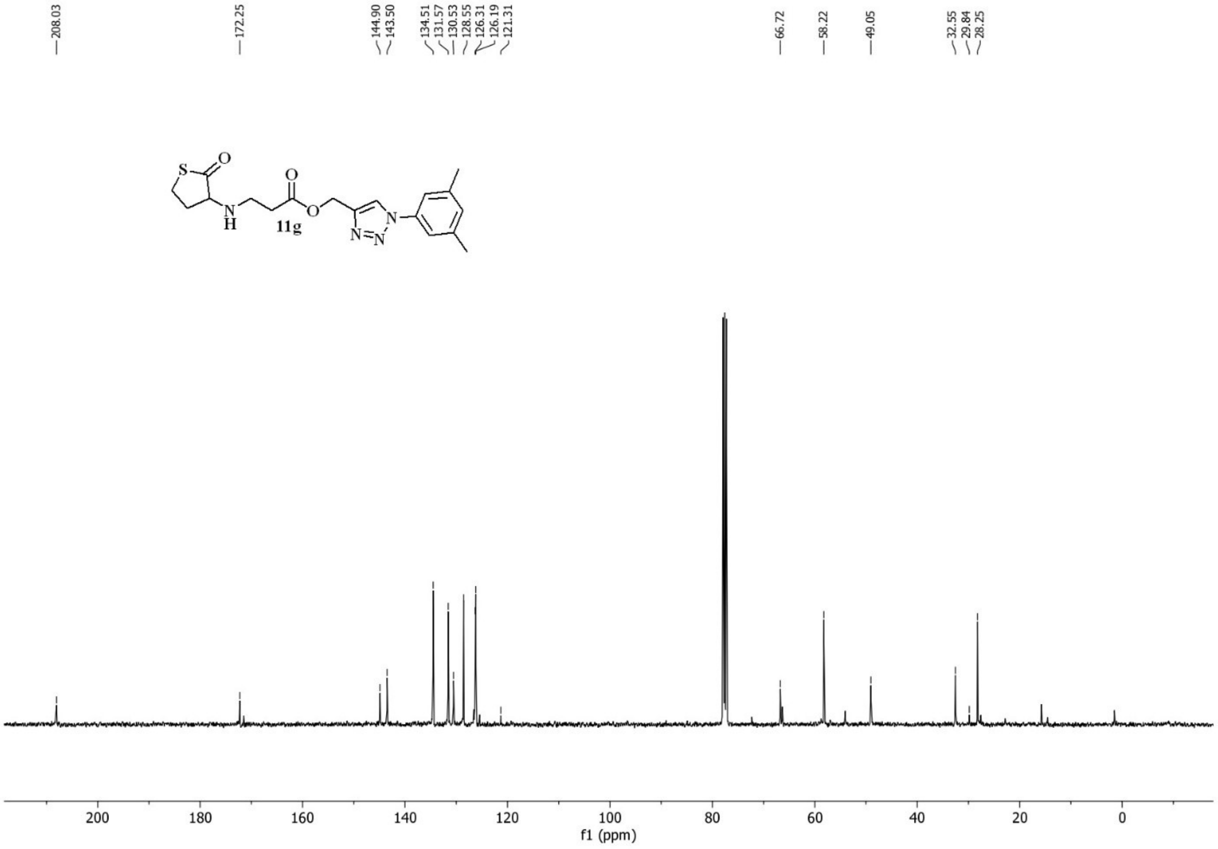

^{13}C-NMR spectrum of 11g (101 MHz, $CDCl_3$)

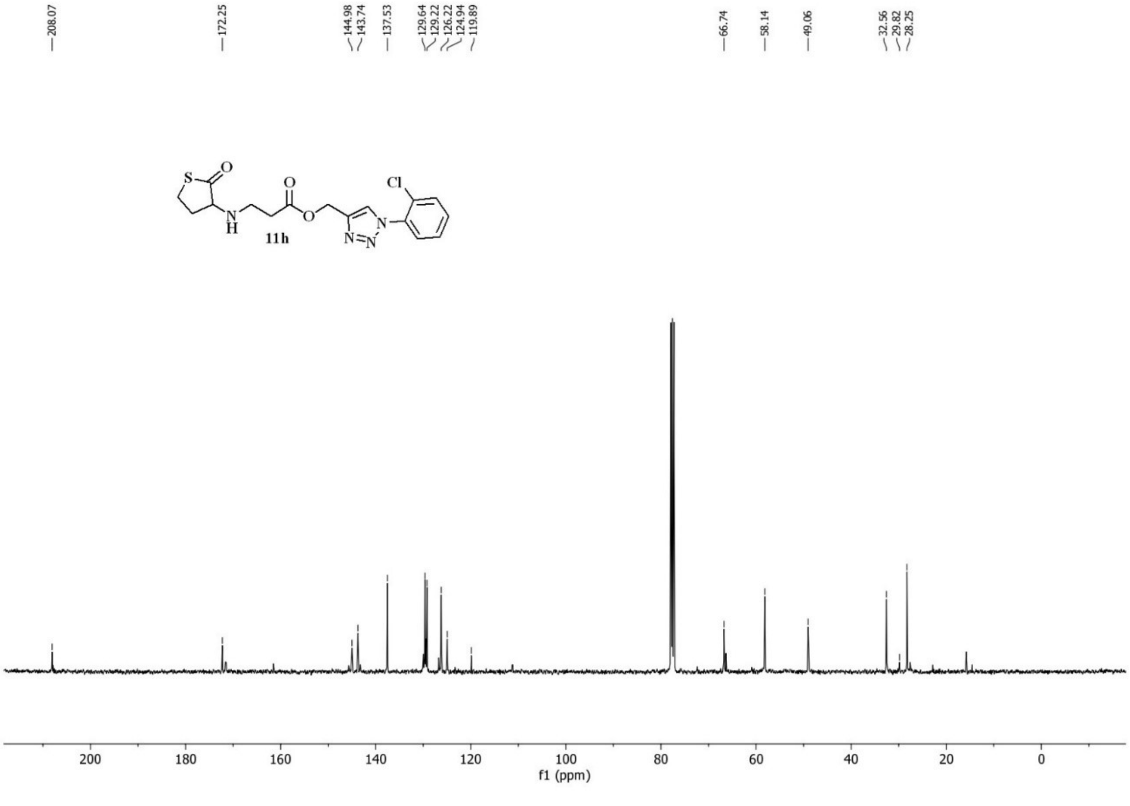

^{13}C-NMR spectrum of 11h (101 MHz, $CDCl_3$)

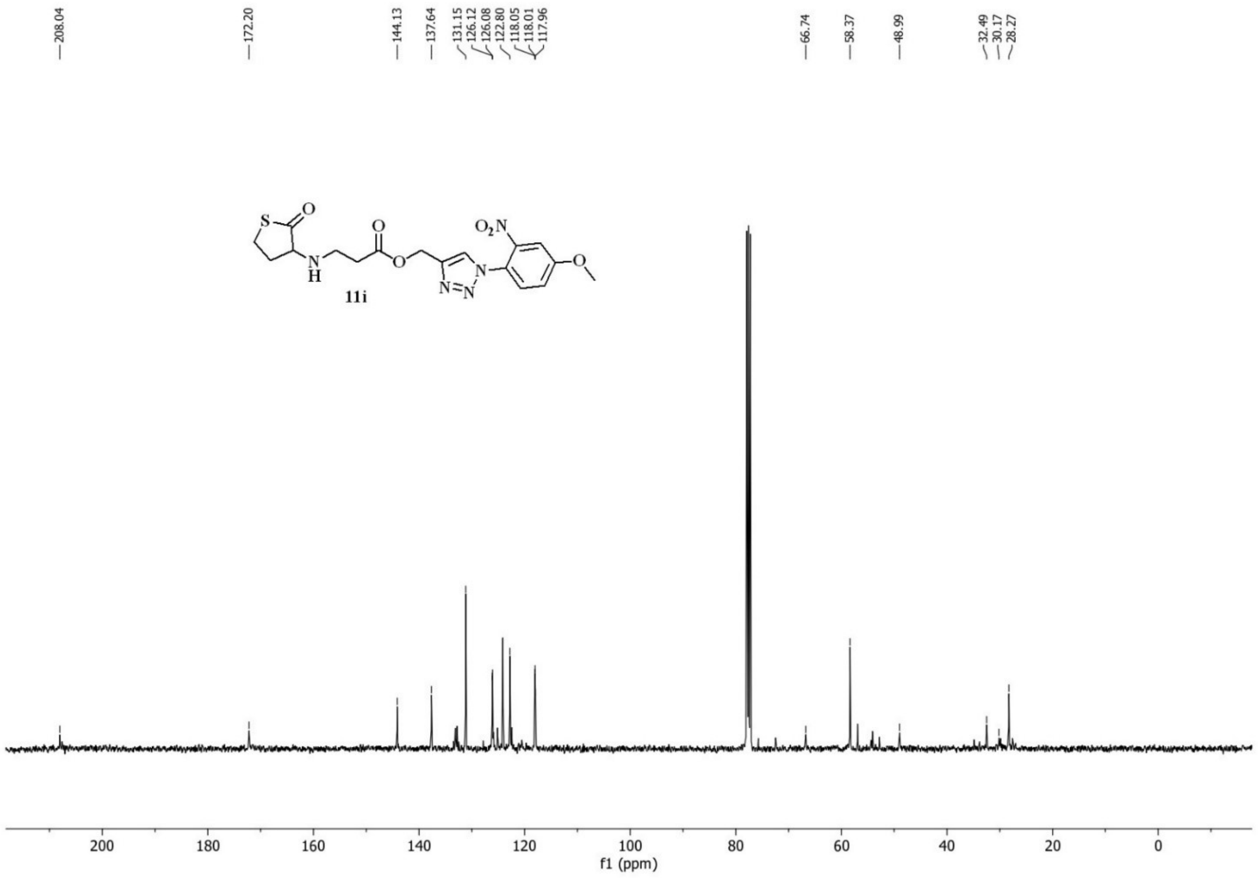

^{13}C-NMR spectrum of 11i (101 MHz, $CDCl_3$)

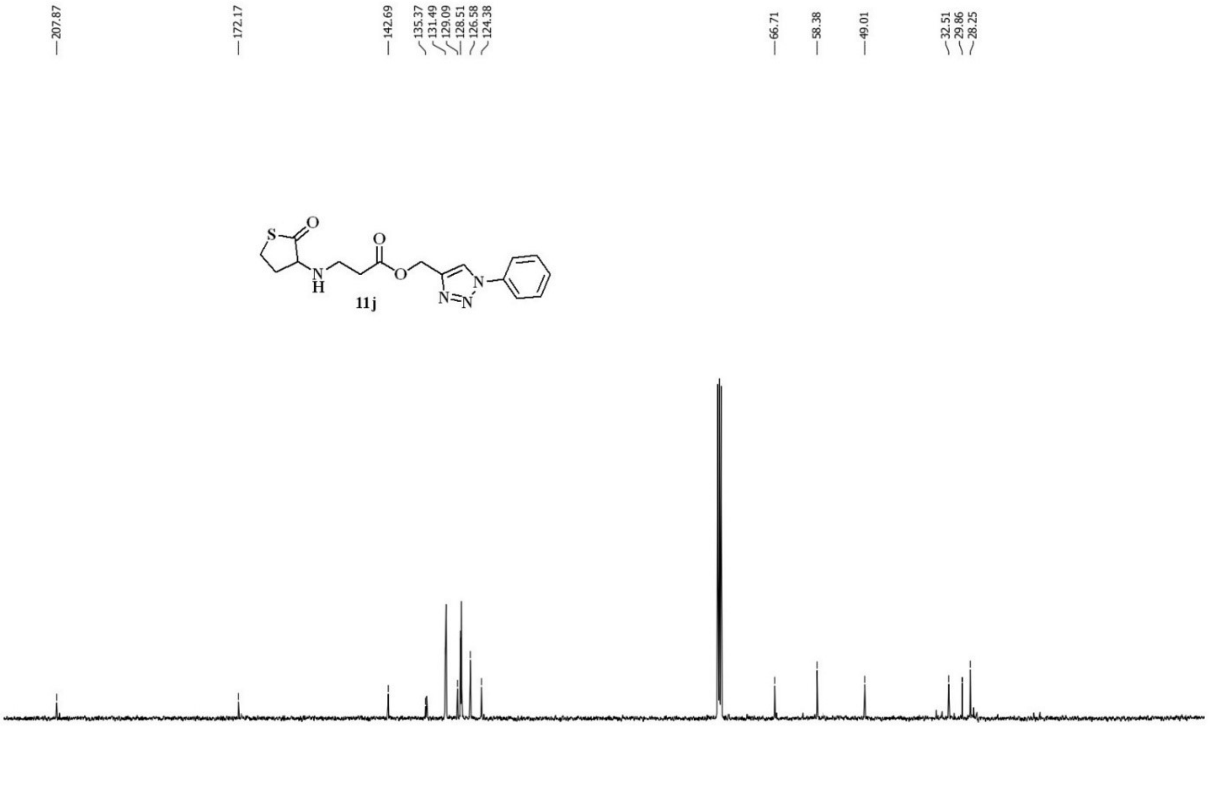

^{13}C-NMR spectrum of 11j (101 MHz, $CDCl_3$)

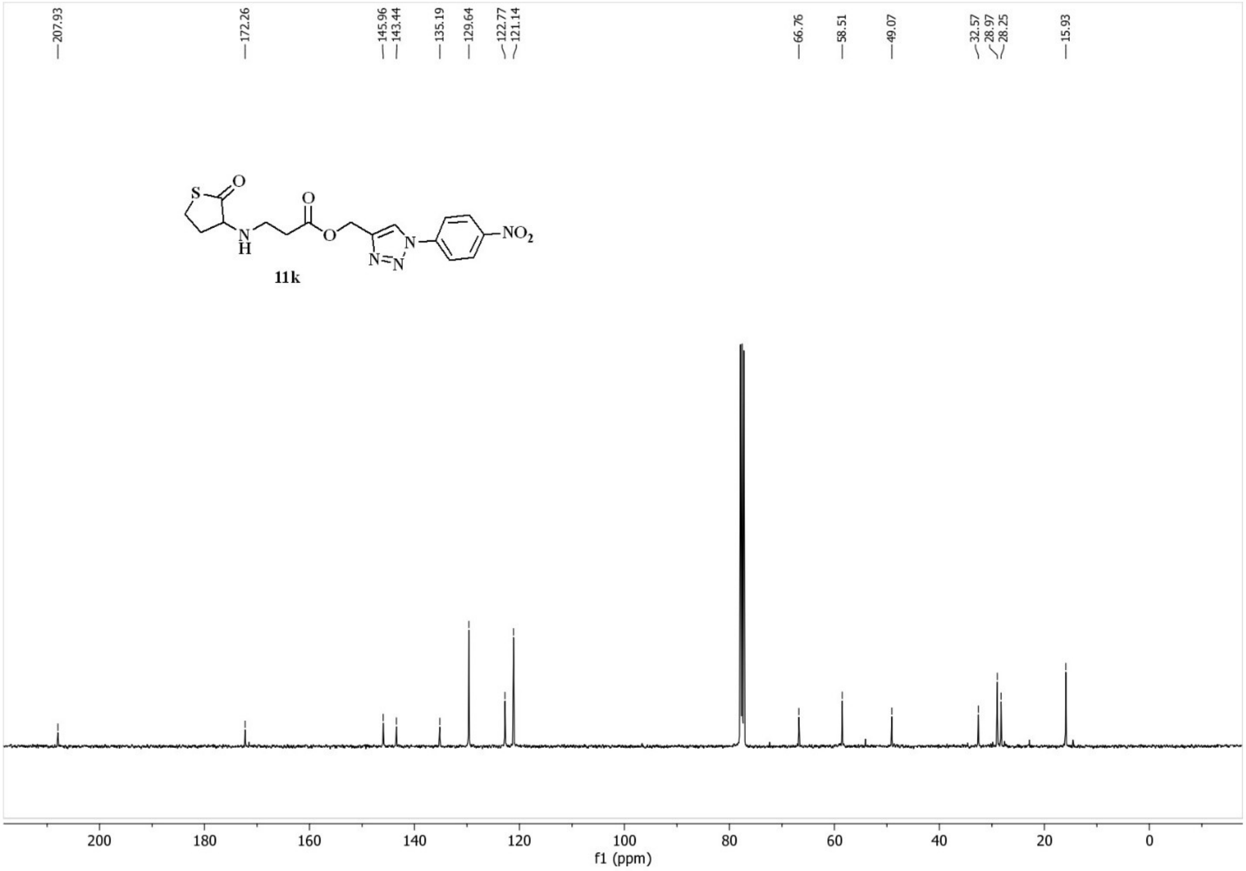

^{13}C-NMR spectrum of 11k (101 MHz, $CDCl_3$)

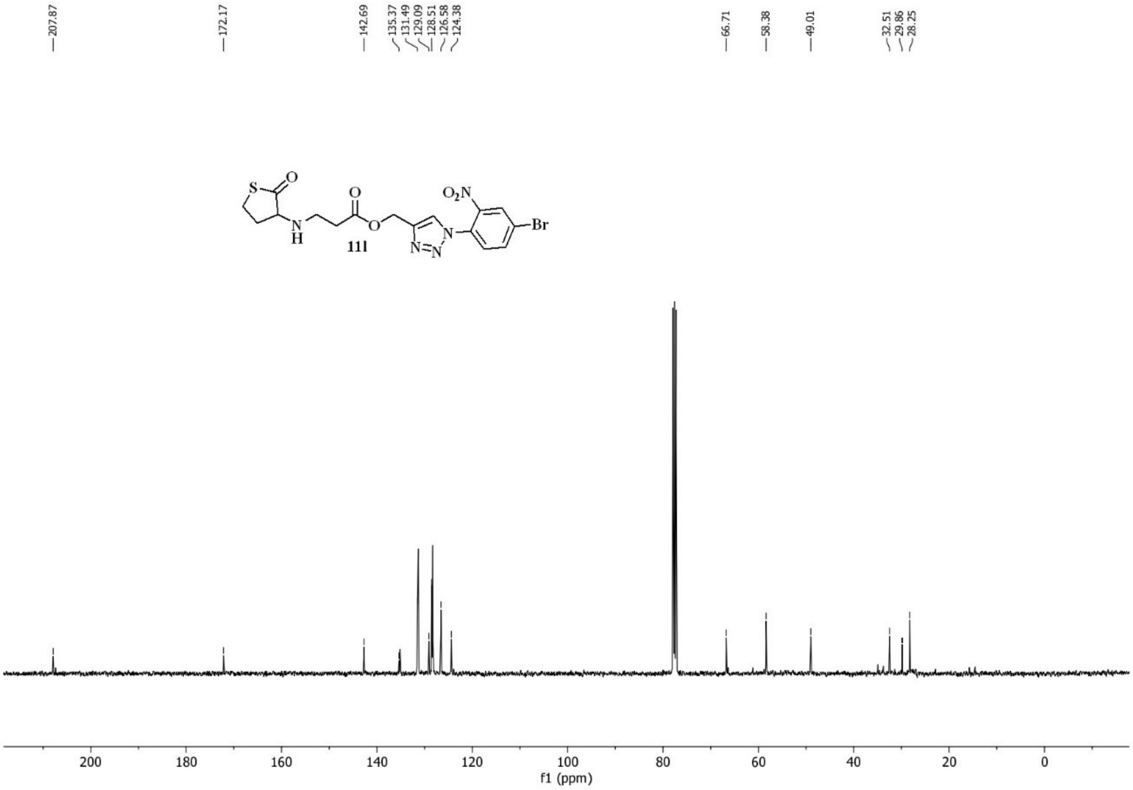

^{13}C-NMR spectrum of 11l (101 MHz, $CDCl_3$)

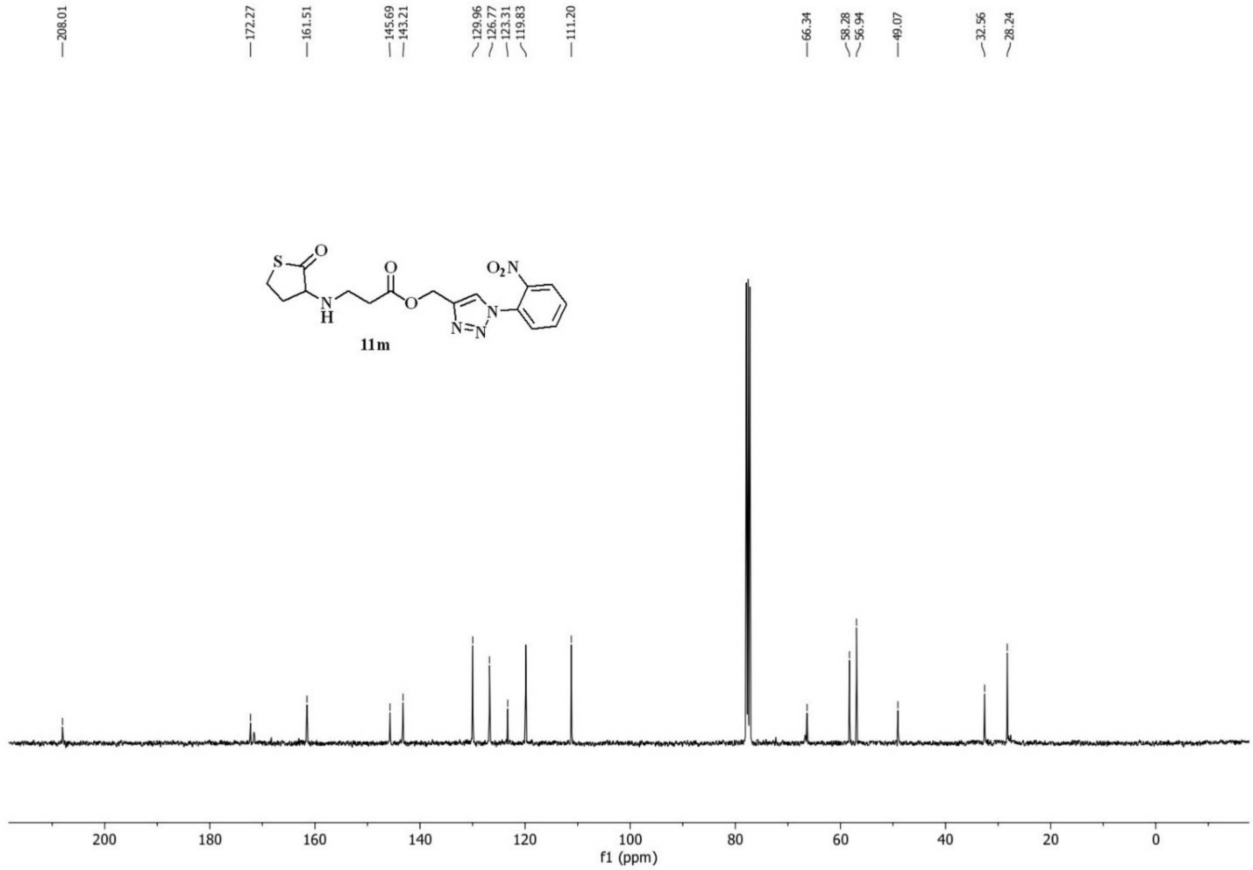

^{13}C-NMR spectrum of 11m (101 MHz, $CDCl_3$)

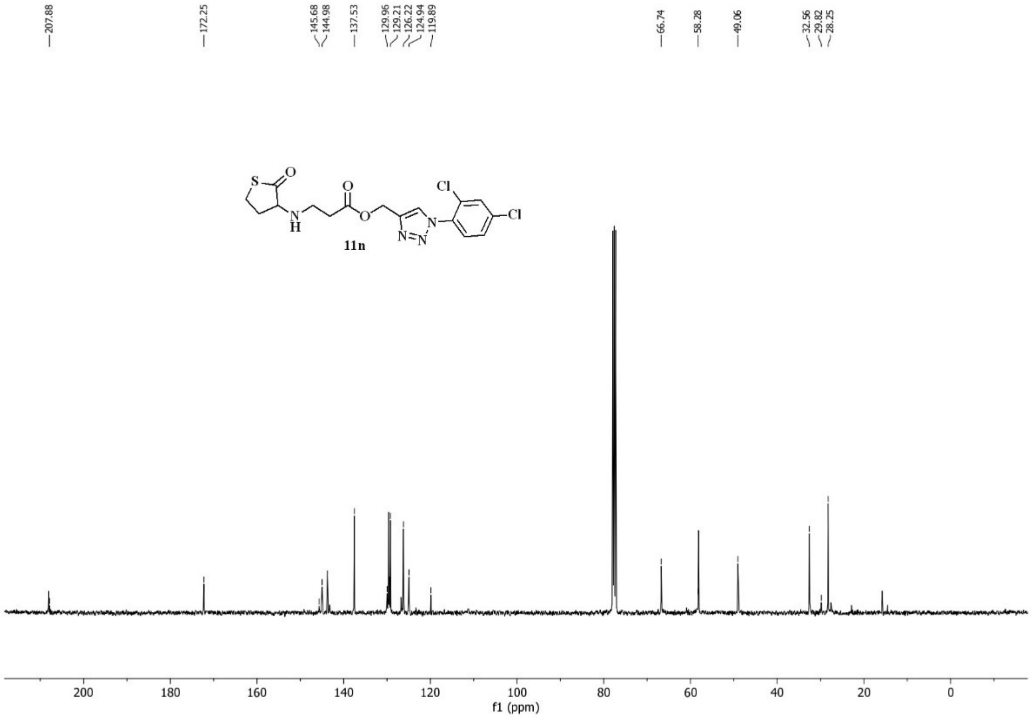

^{13}C-NMR spectrum of 11n (101 MHz, $CDCl_3$)

4. HRMS and ESI spectrum of final compounds (10a-p and 11a-n):

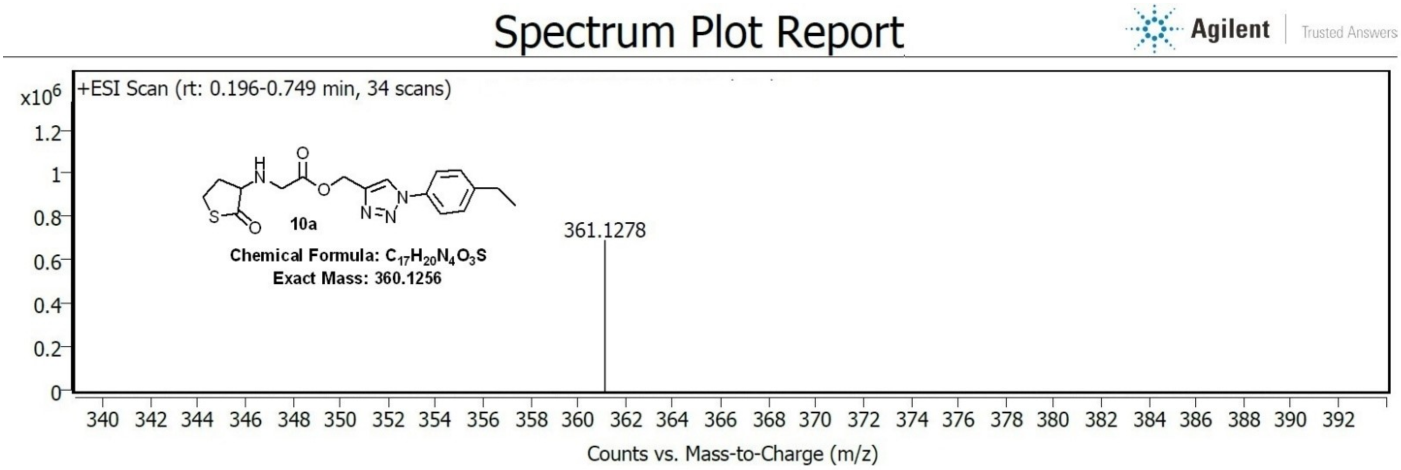

HRMS of 10a

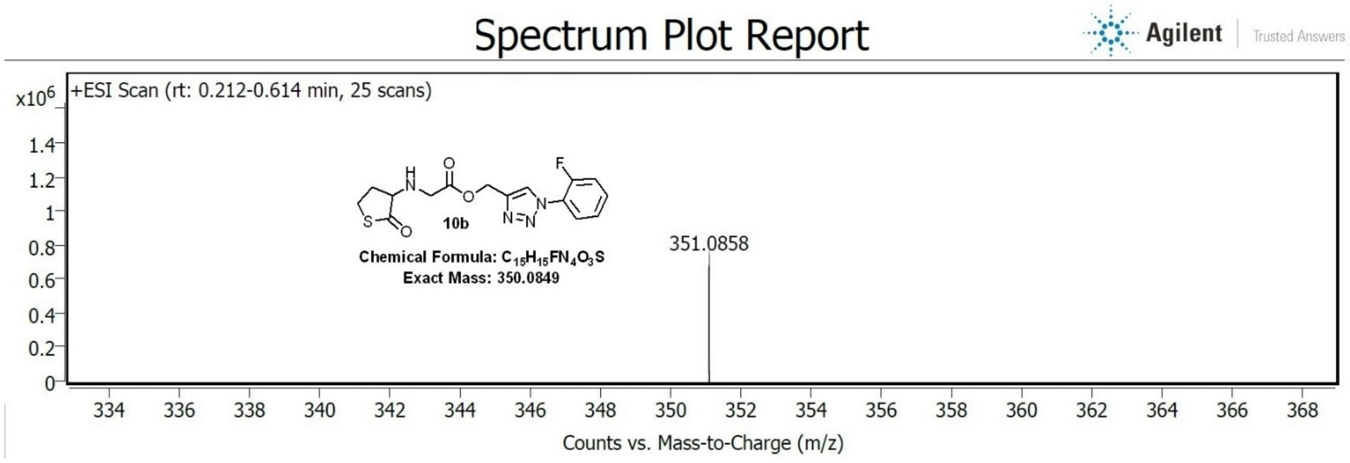

HRMS of 10b

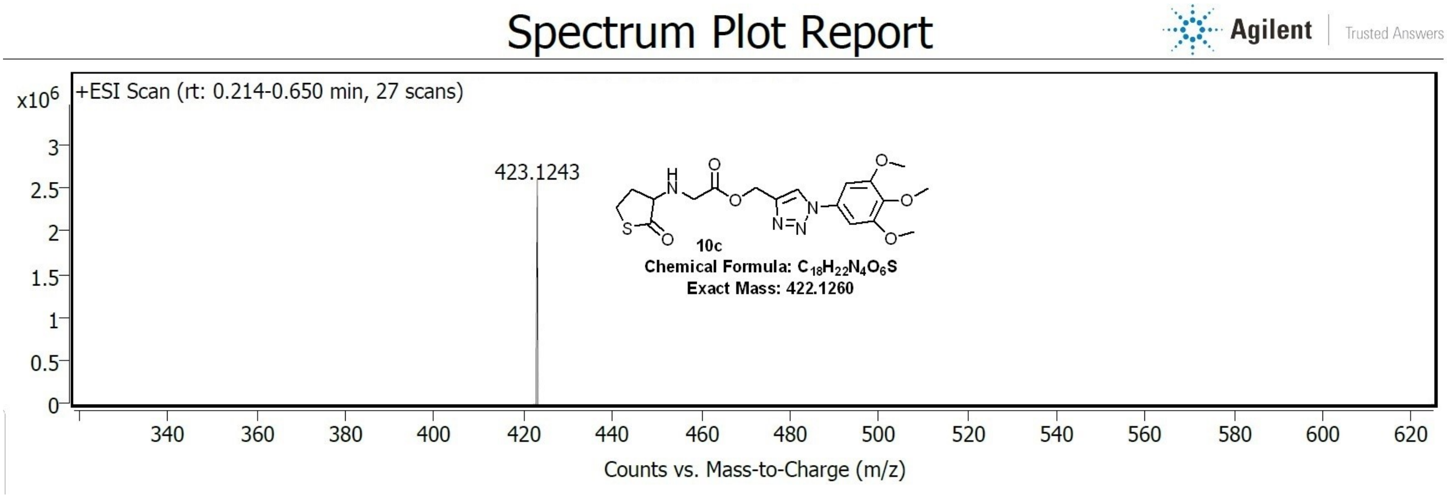

HRMS of 10c

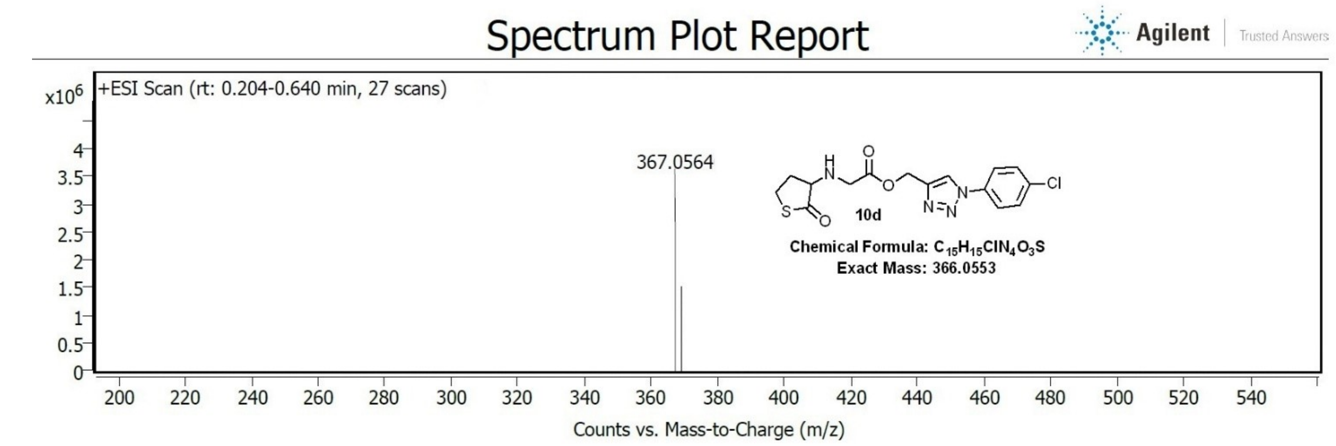

HRMS of 10d

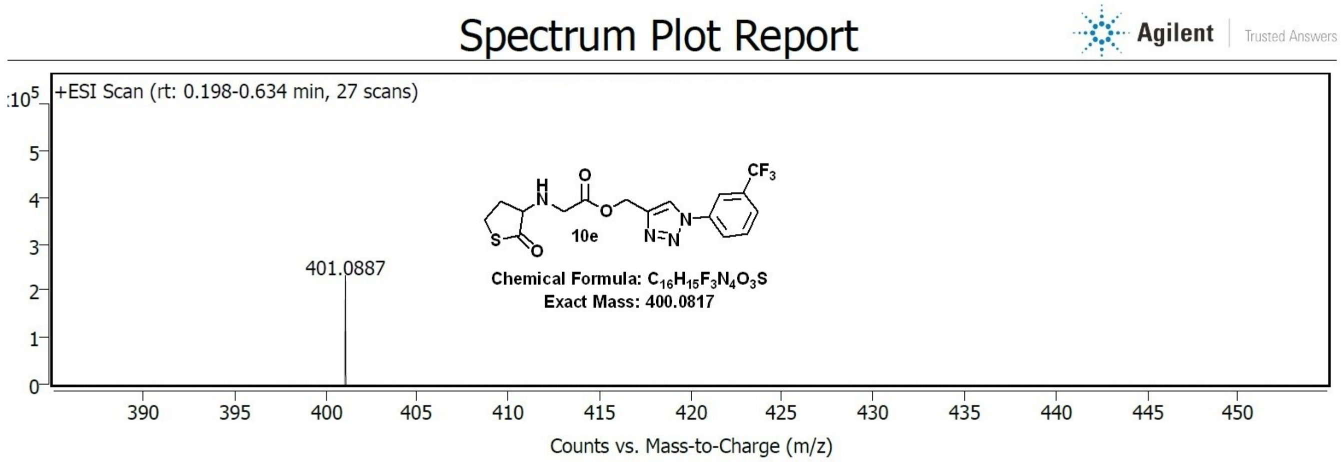

HRMS of 10e

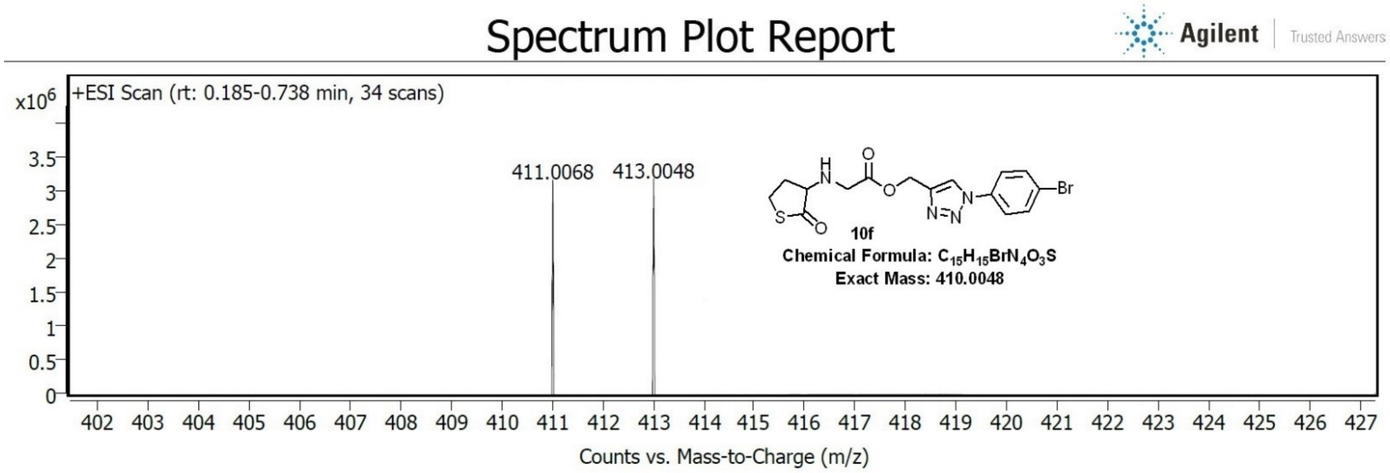

HRMS of 10f

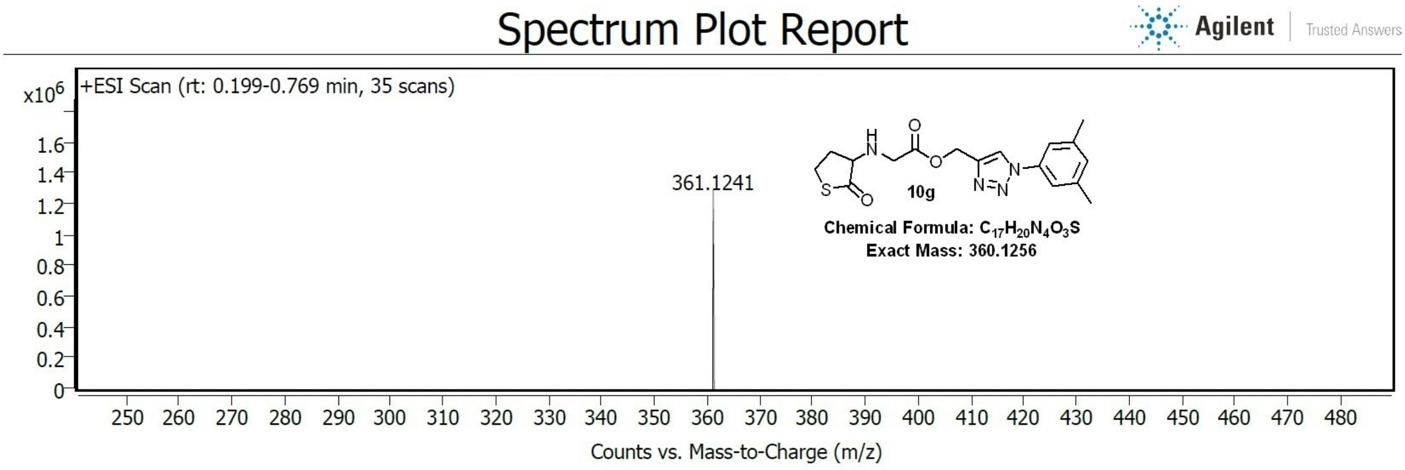

HRMS of 10g

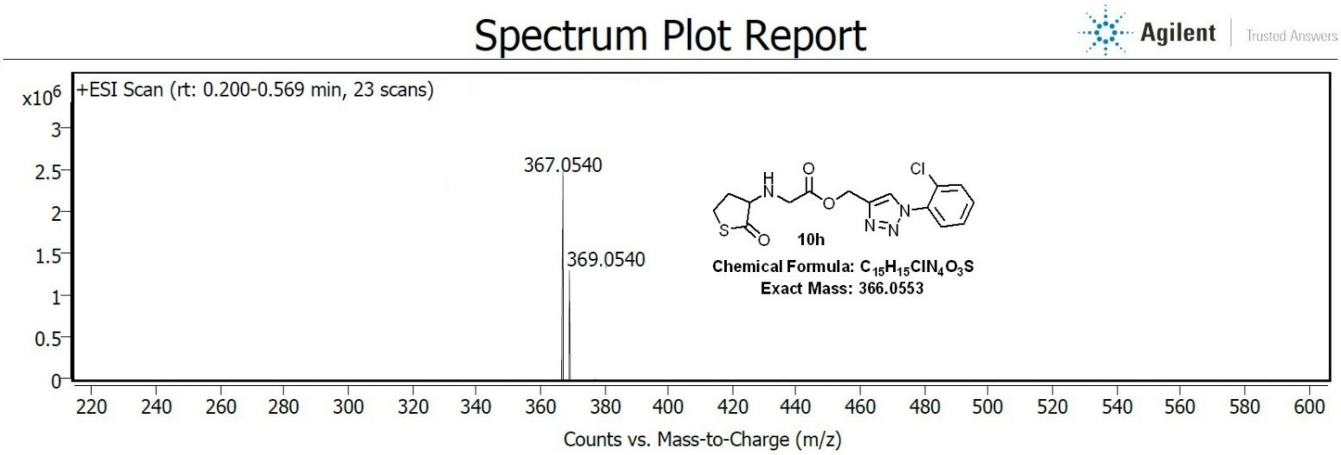

HRMS of 10h

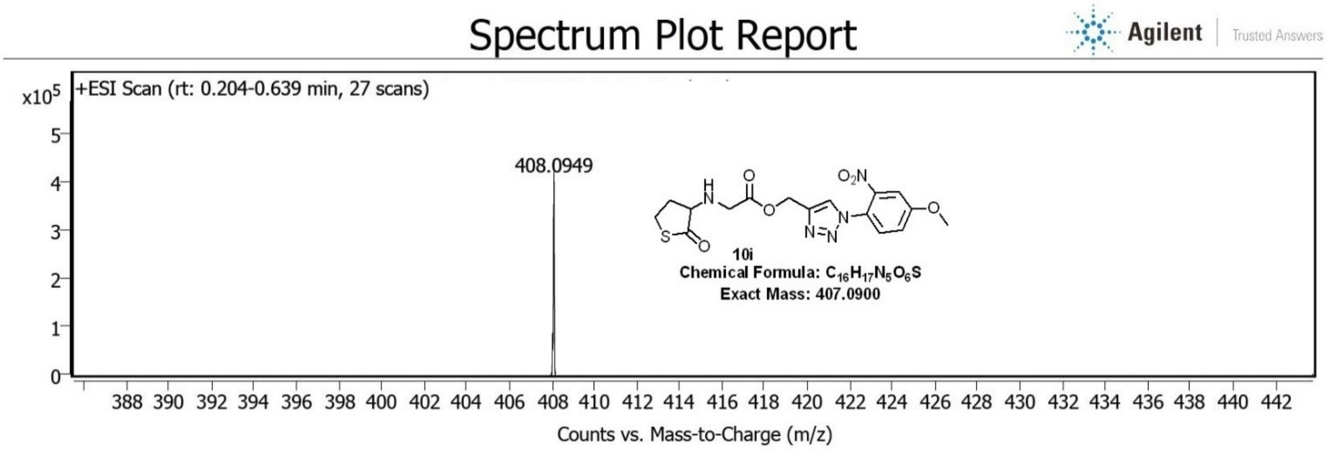

HRMS of 10i

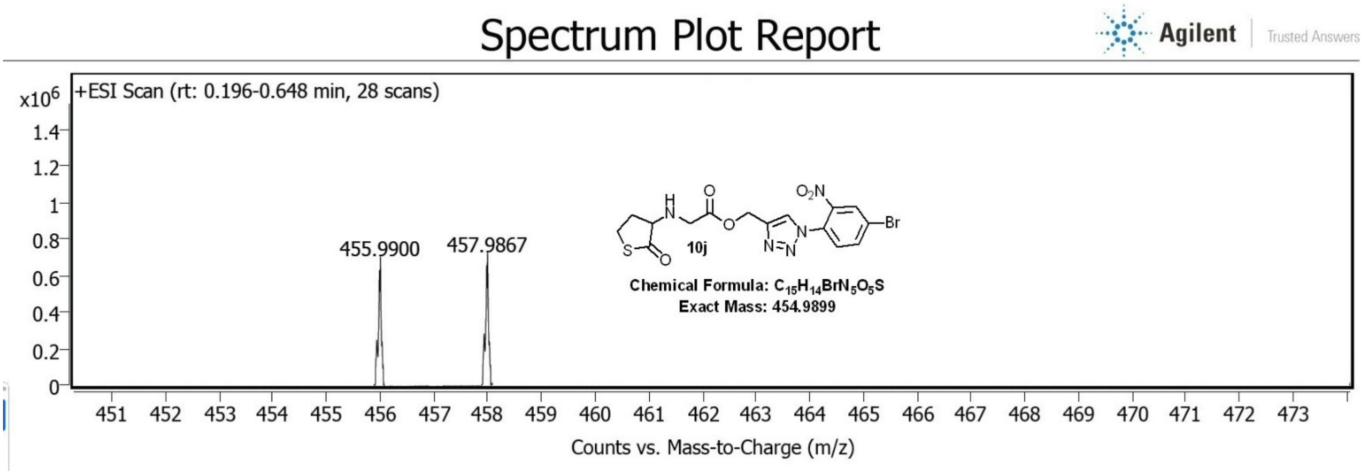

HRMS of 10j

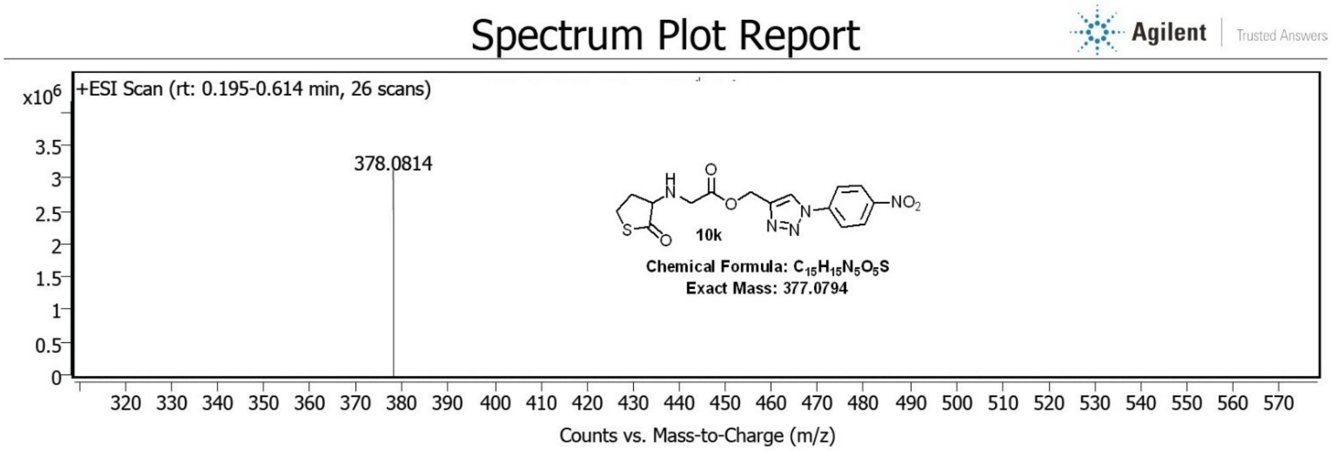

HRMS of 10k

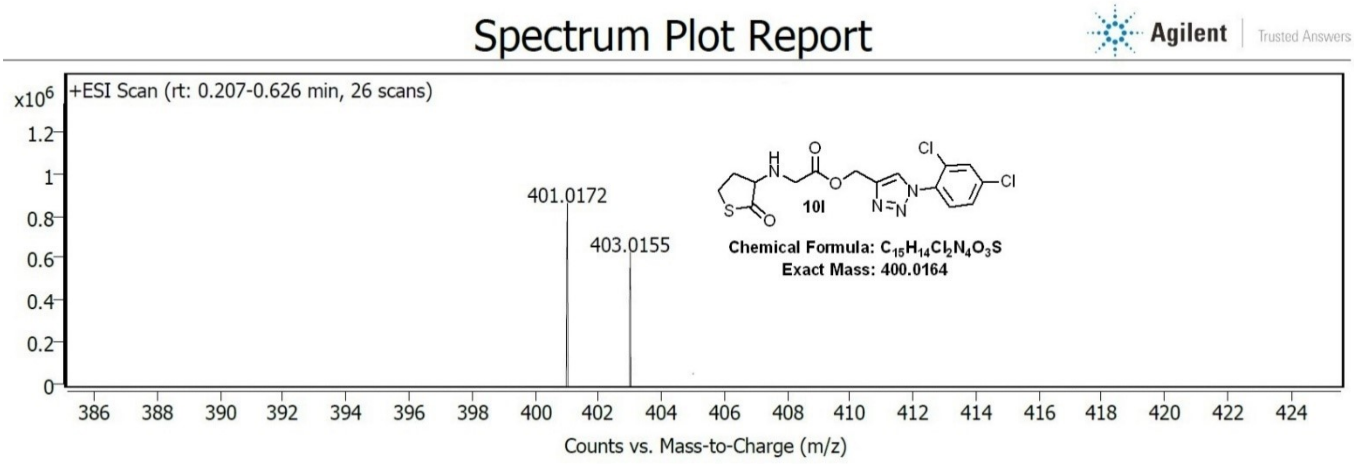

HRMS of 10l

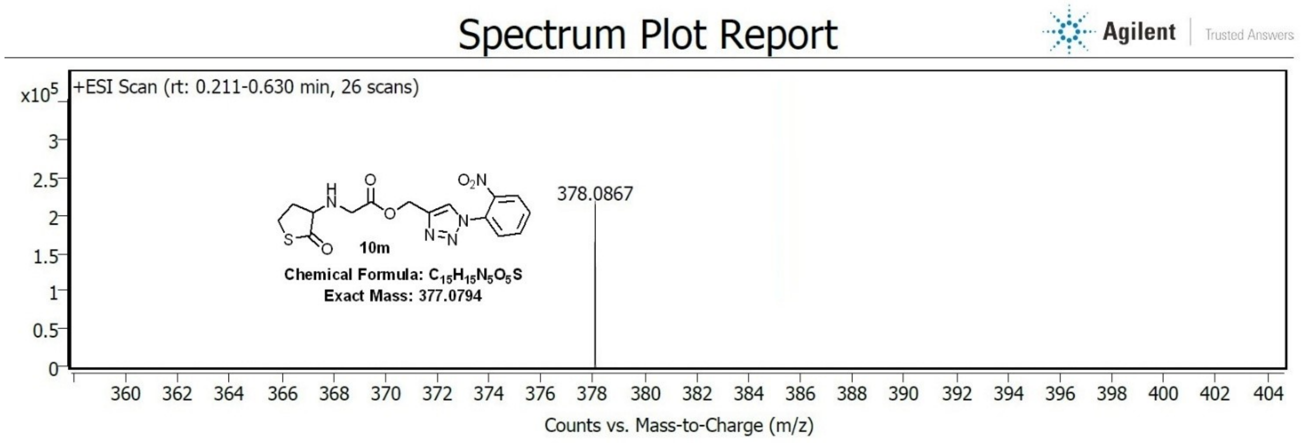

HRMS of 10m

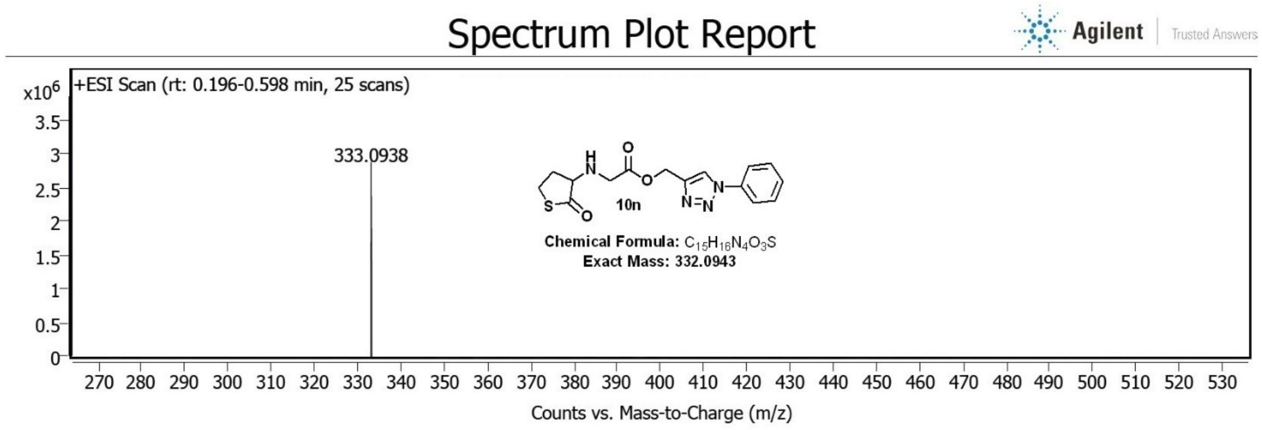

HRMS of 10n

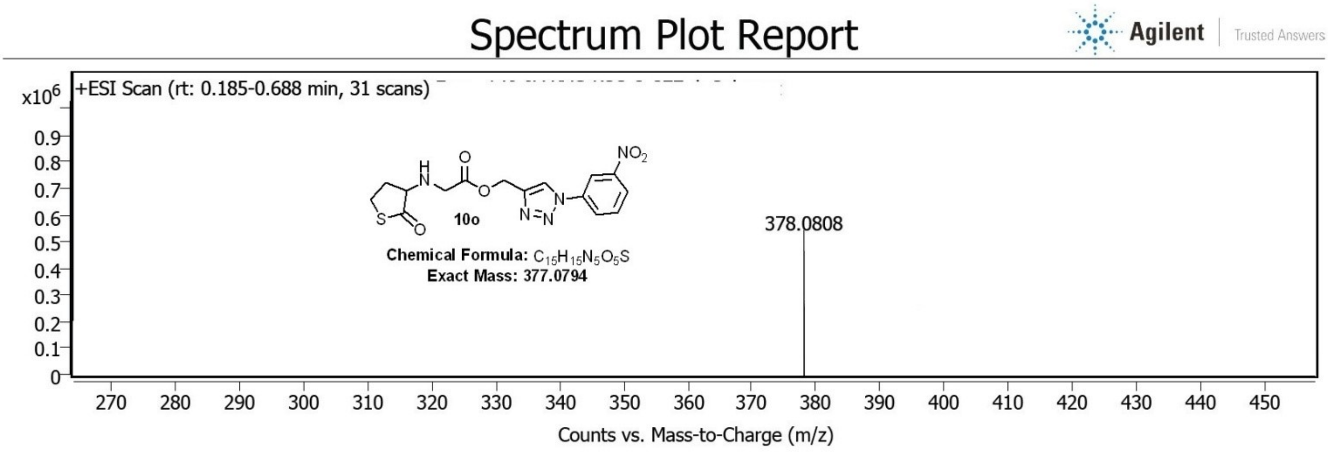

HRMS of 10o

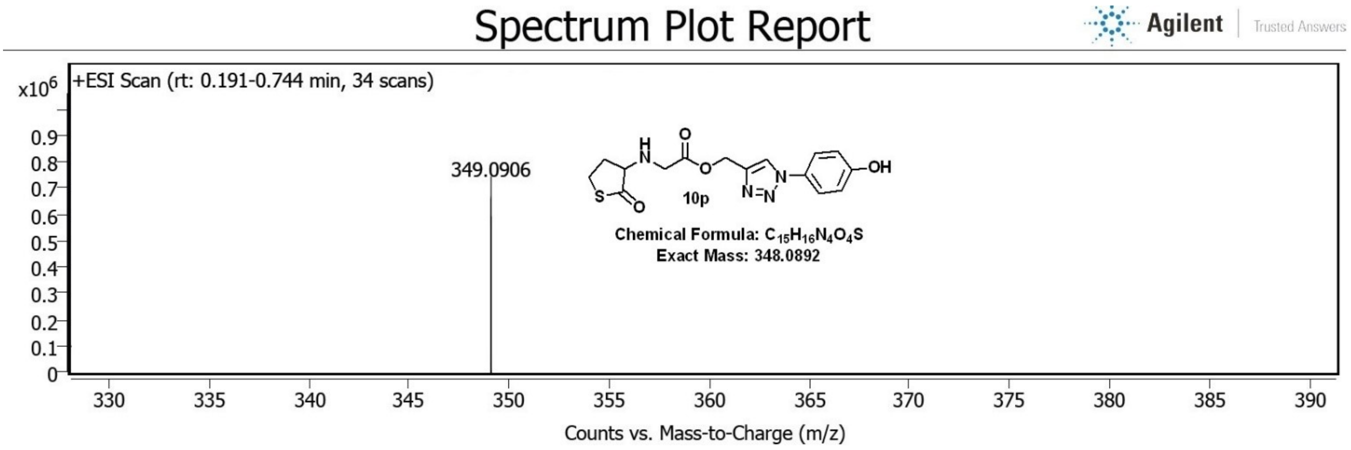

HRMS of 10p

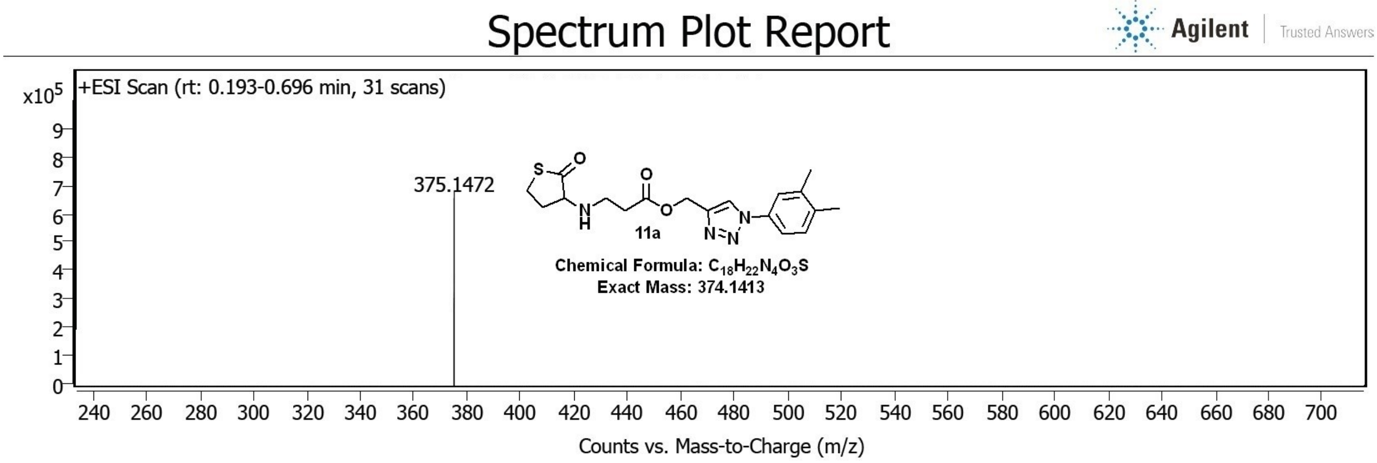

HRMS of 11a

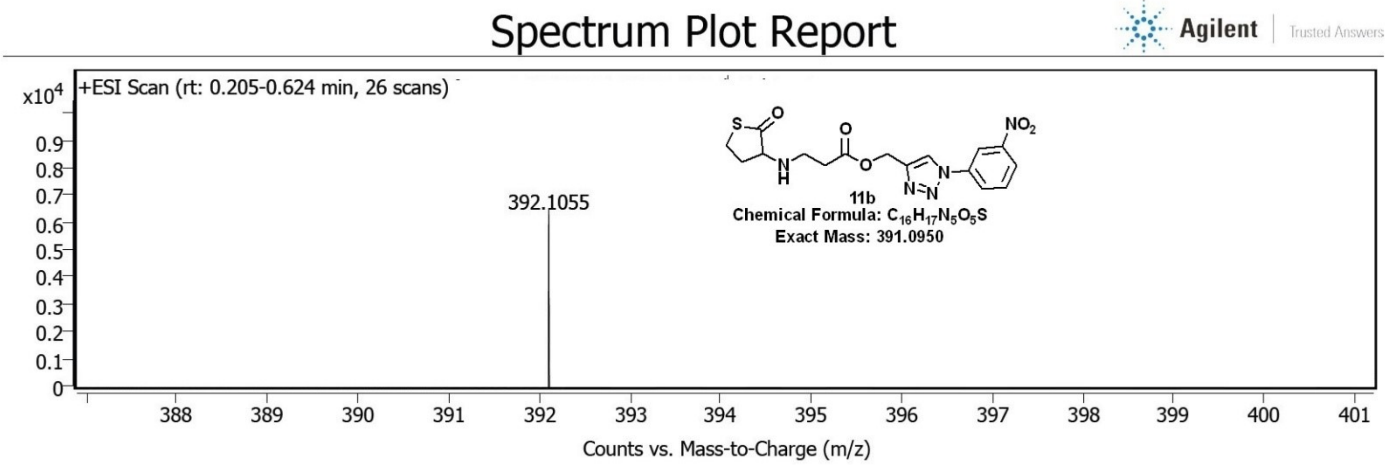

HRMS of 11b

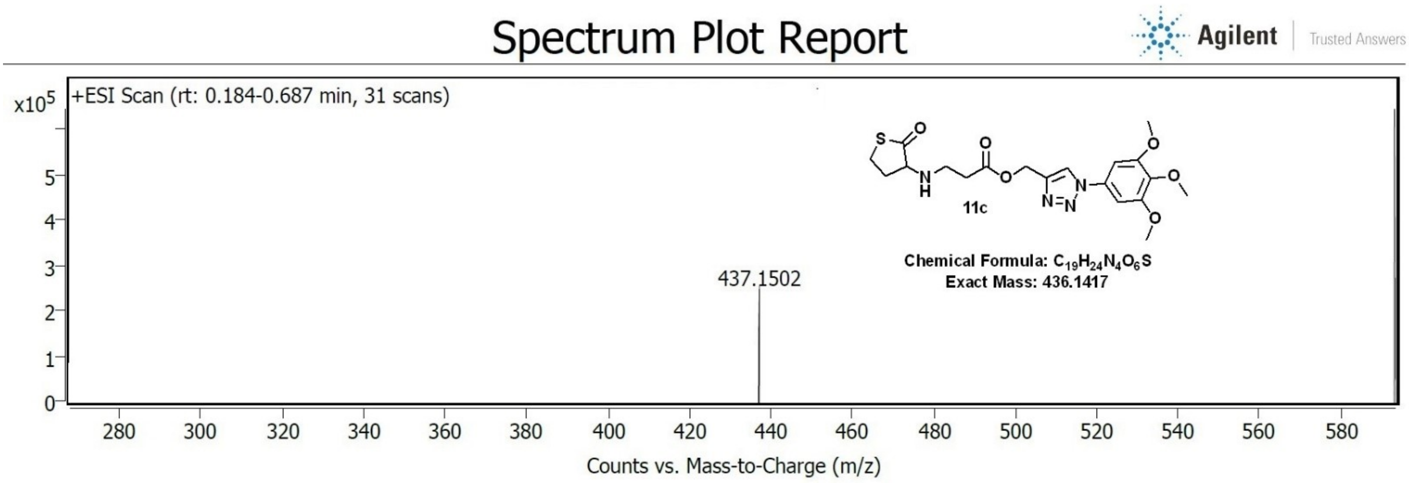

HRMS of 11c

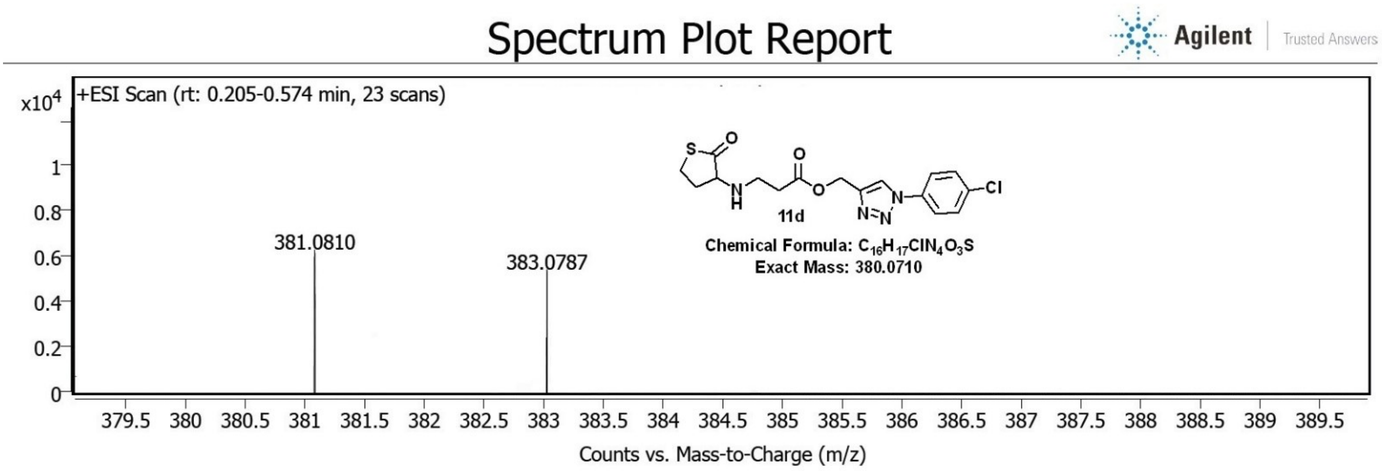

HRMS of 11d

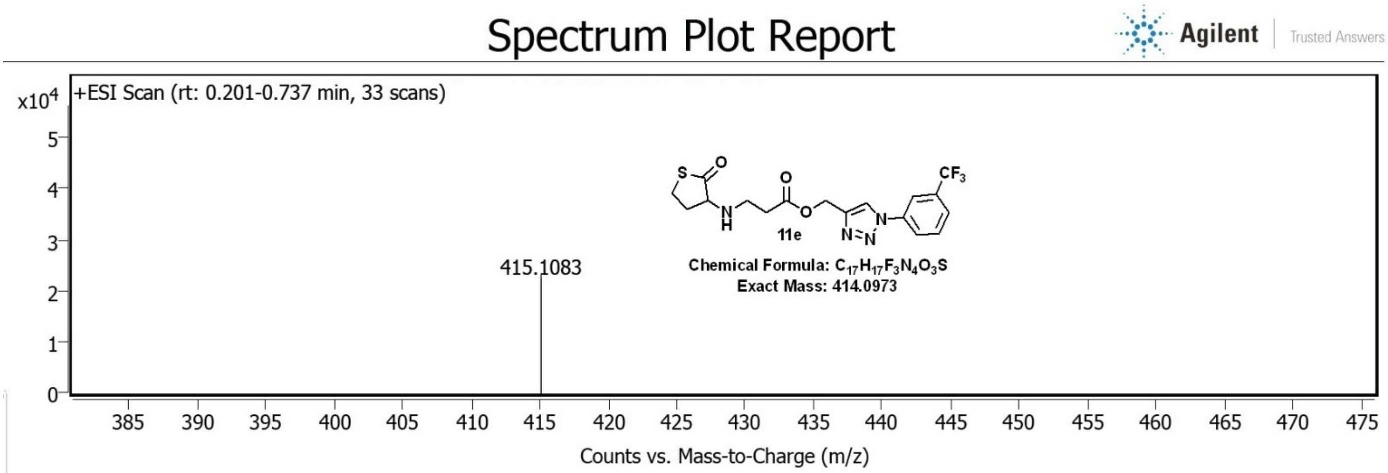

HRMS of 11e

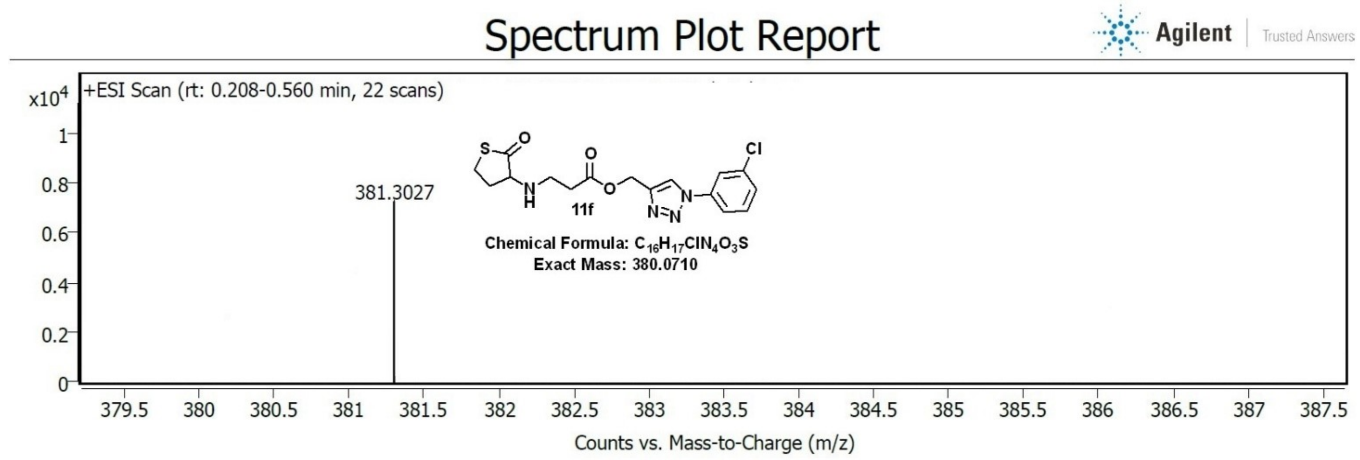

HRMS of 11f

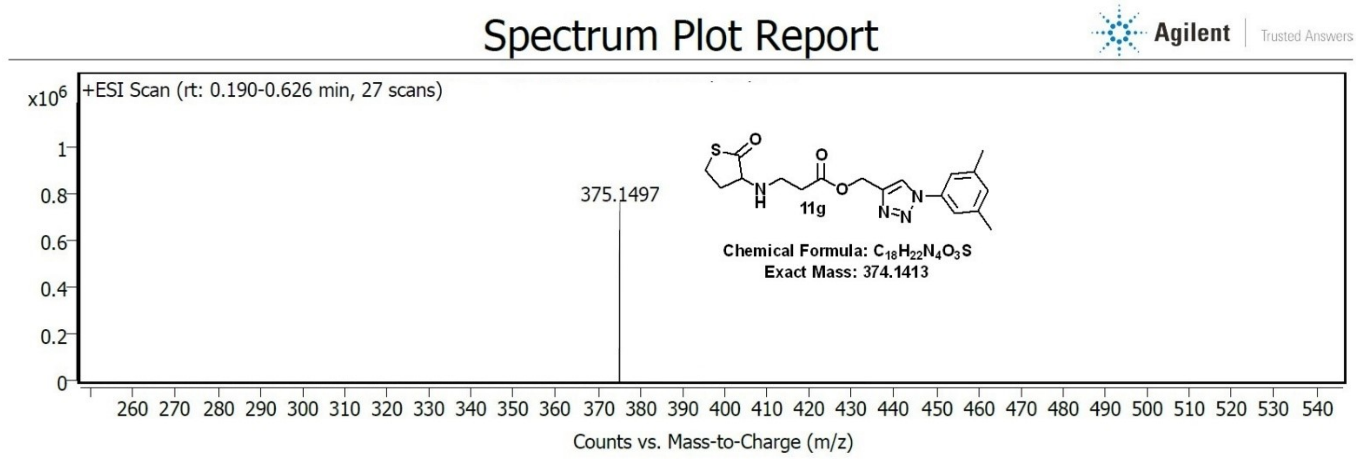

HRMS of 11g

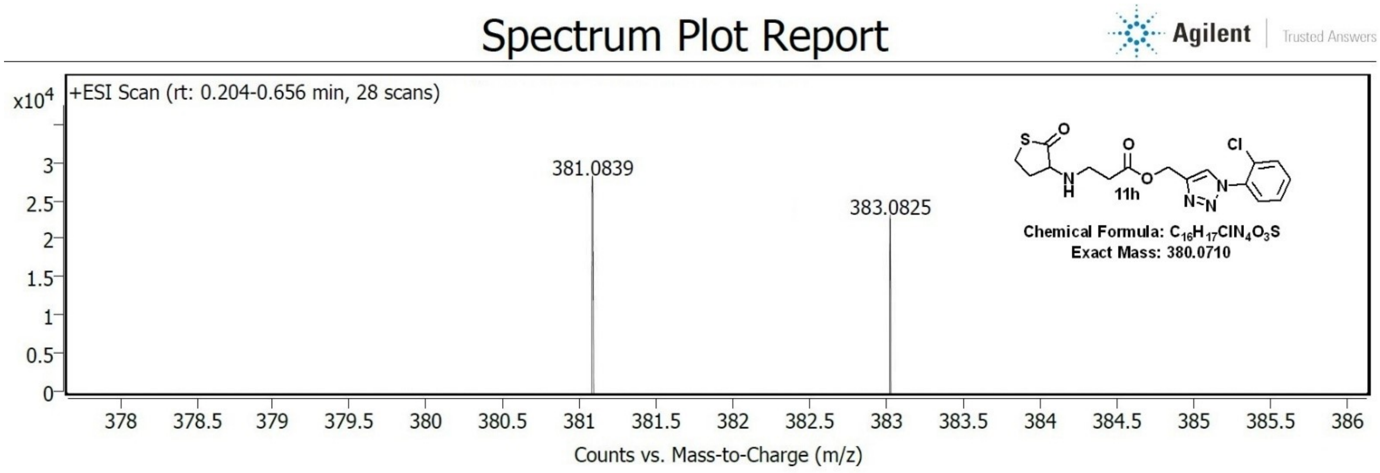

HRMS of 11h

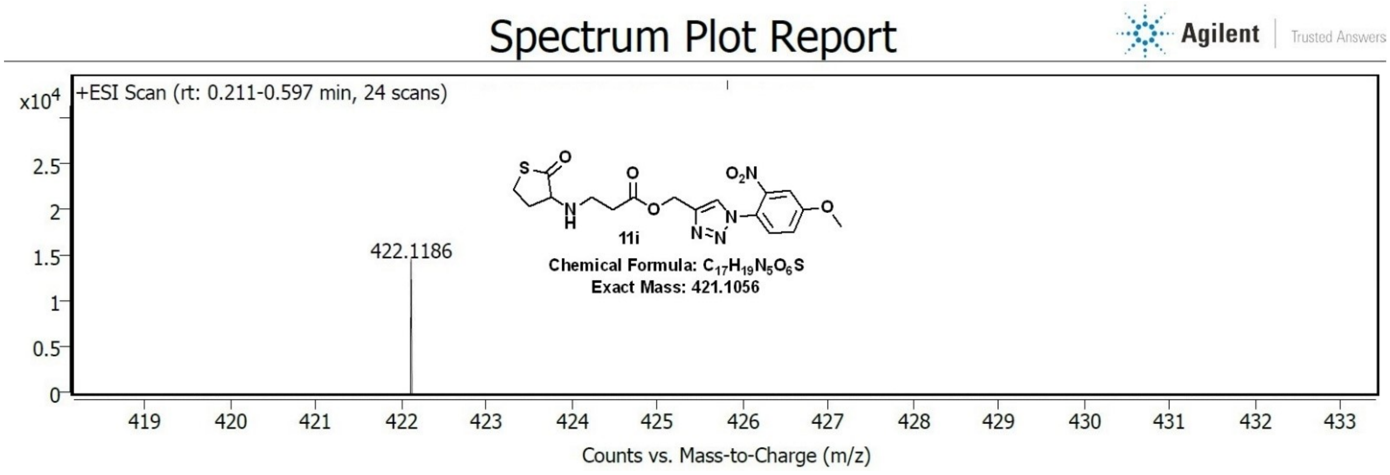

HRMS of 11i

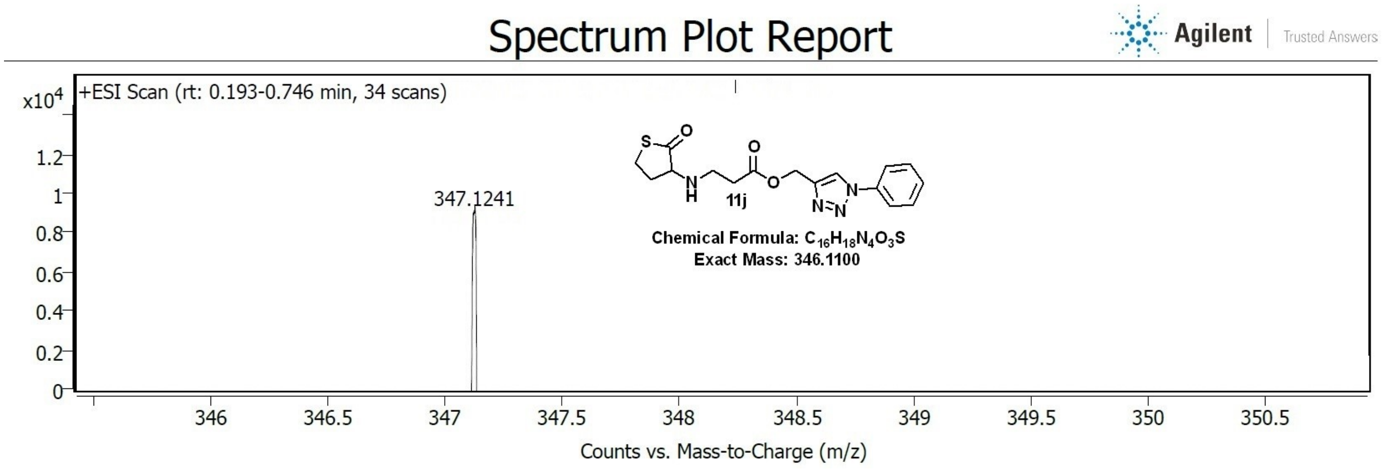

HRMS of 11j

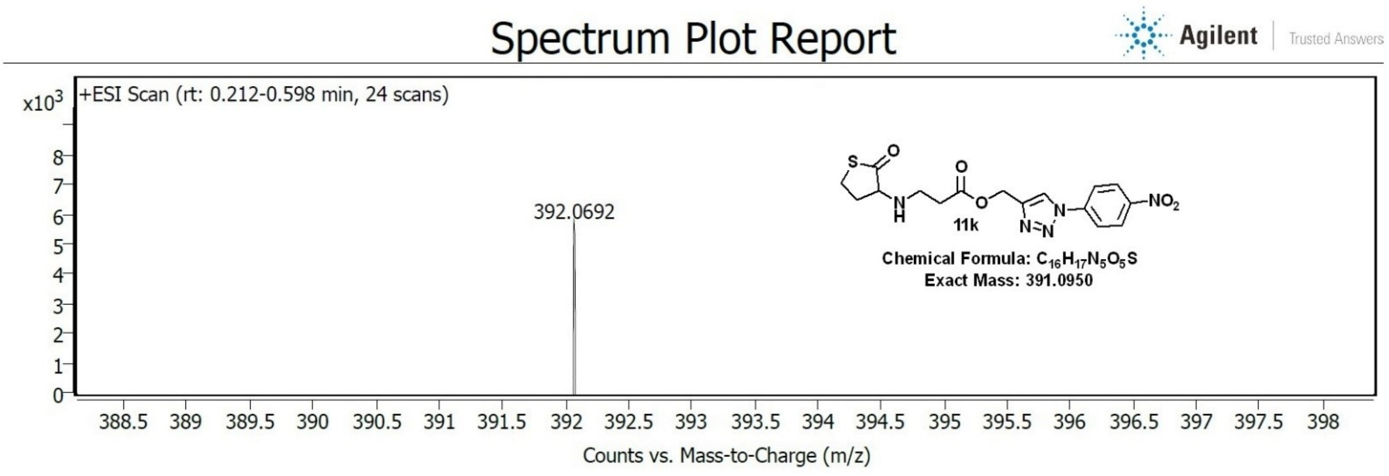

HRMS of 11k

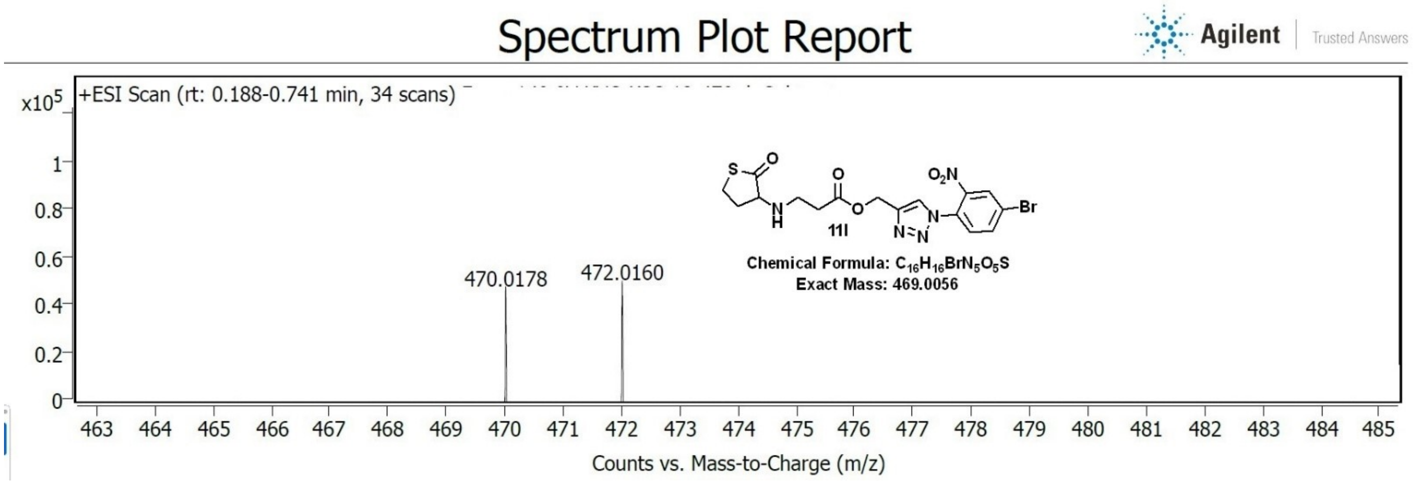

HRMS of11l

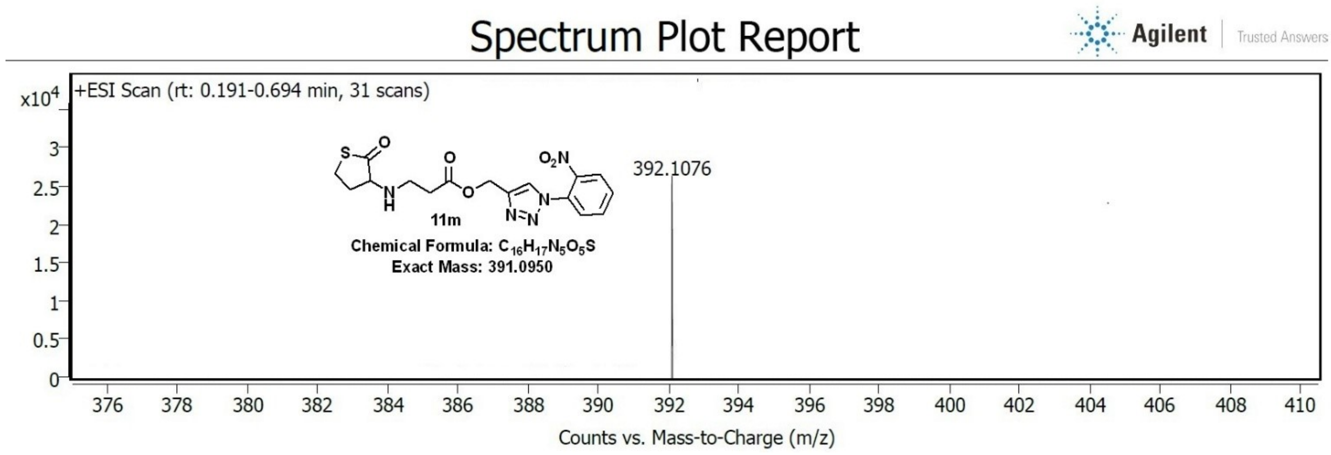

HRMS of 11m

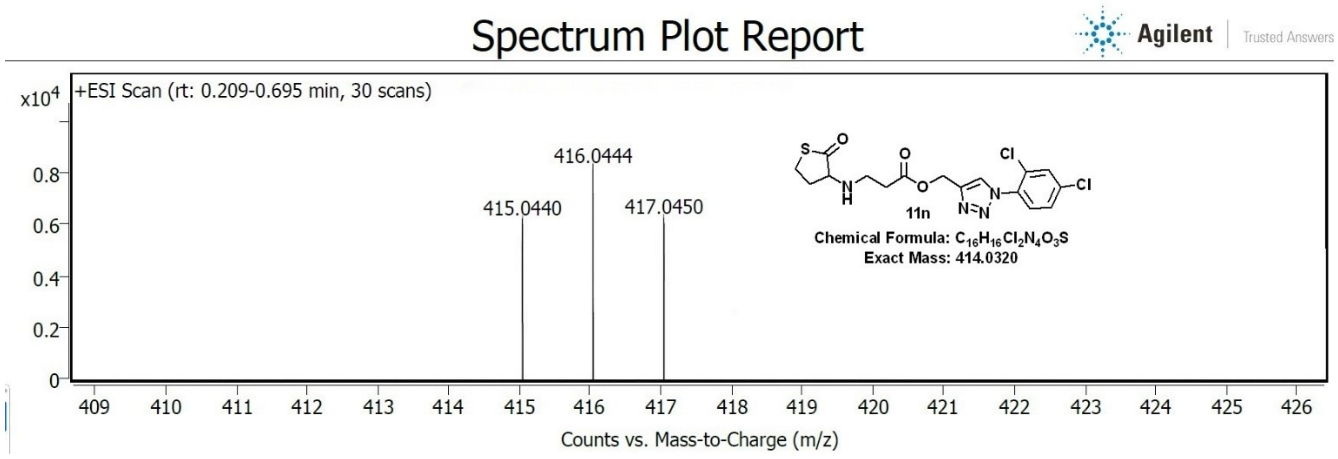

HRMS of 11n

5. **IR spectrum of final compounds (10a-p and 11a-n):**

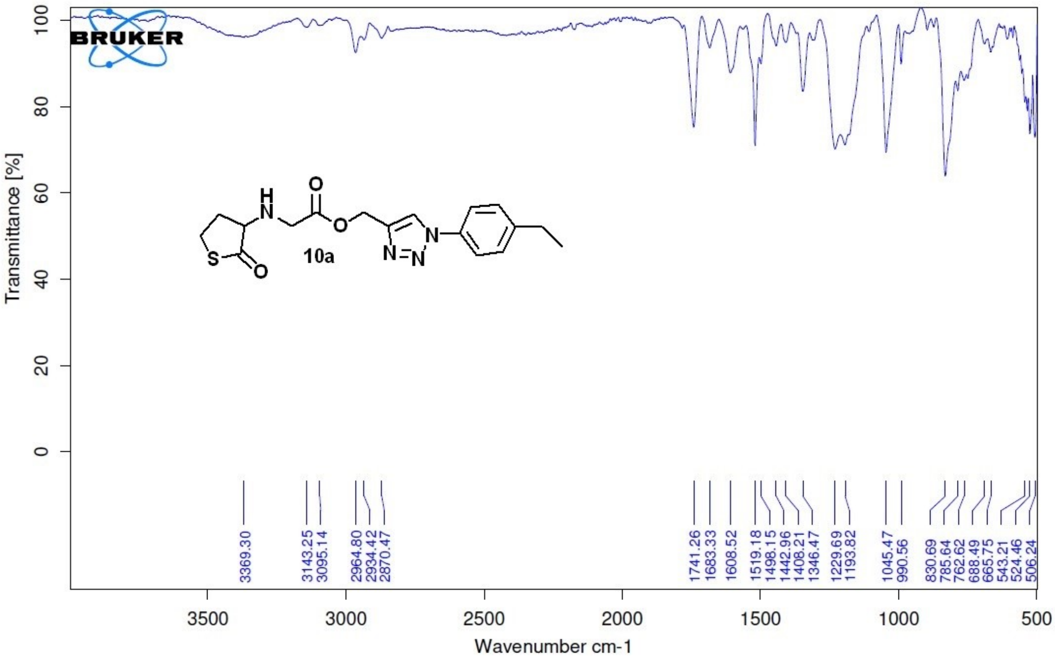

IR spectrum of 10a

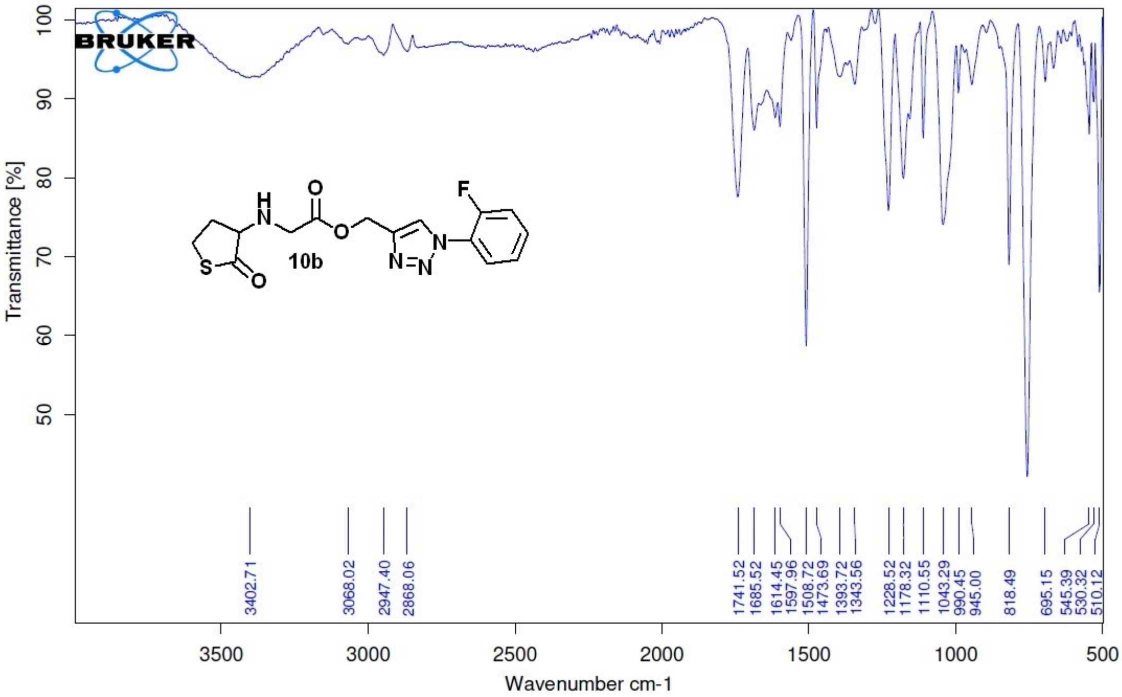

IR spectrum of 10b

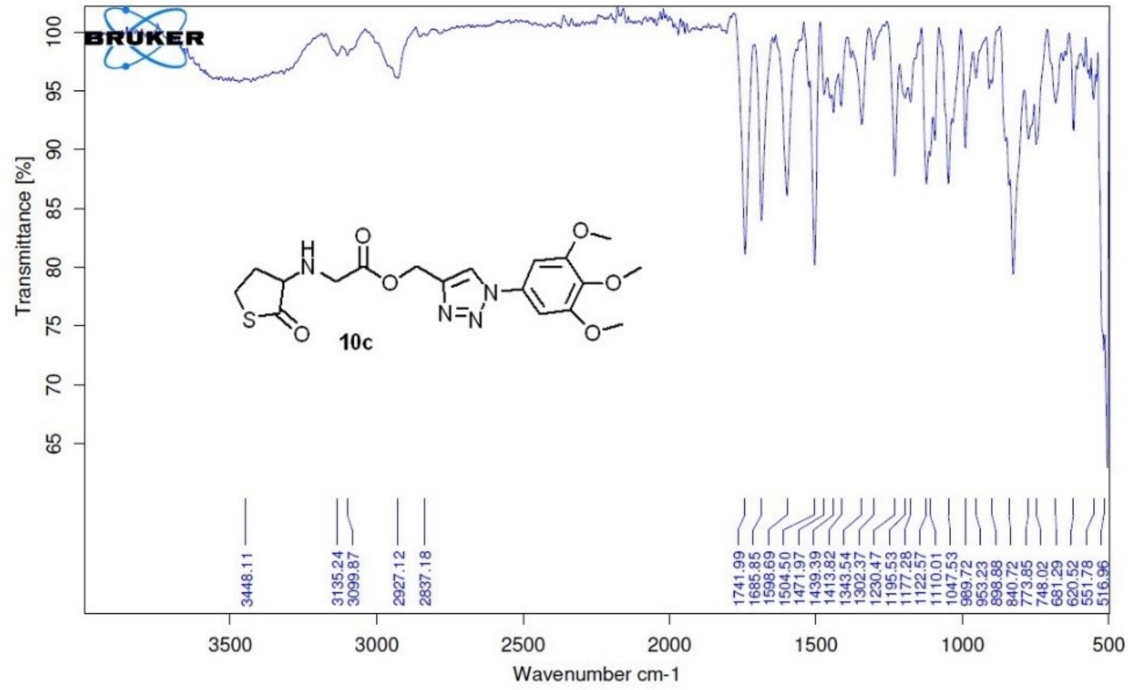

IR spectrum of 10c

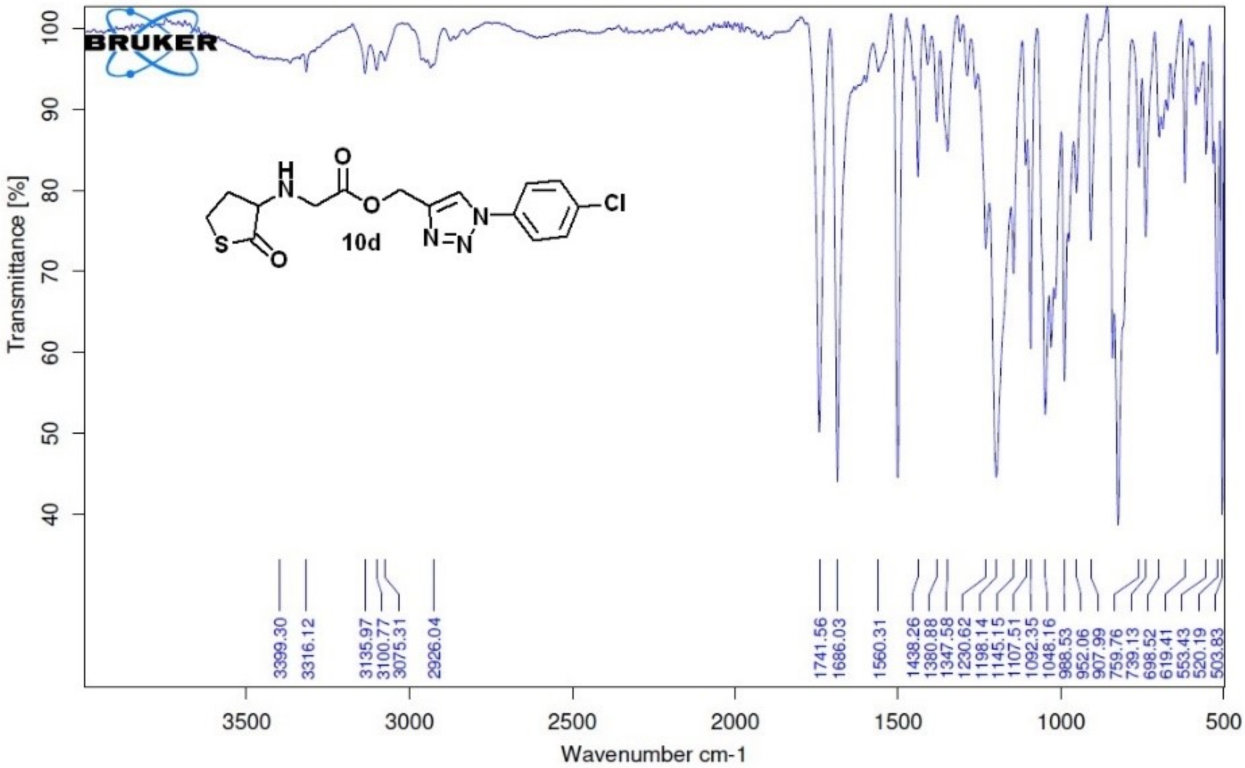

IR spectrum of 10d

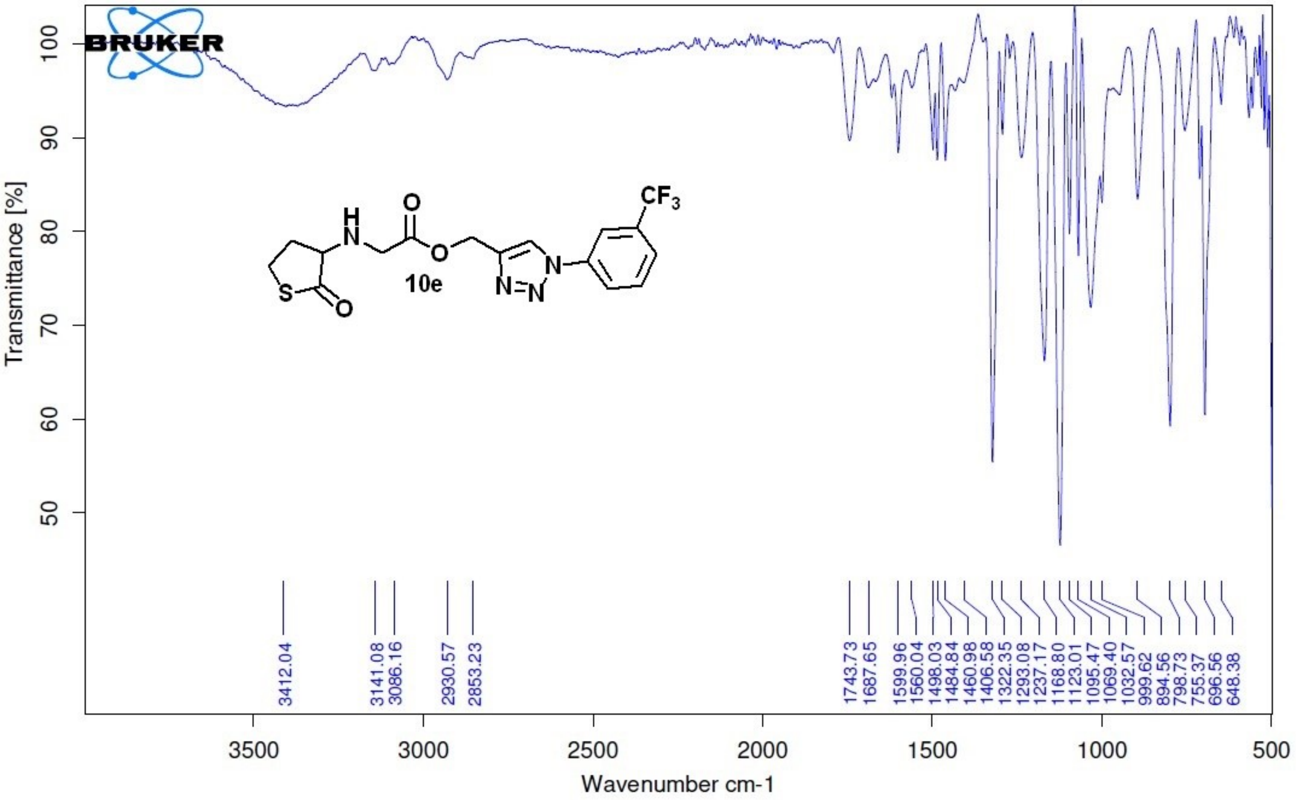

IR spectrum of 10e

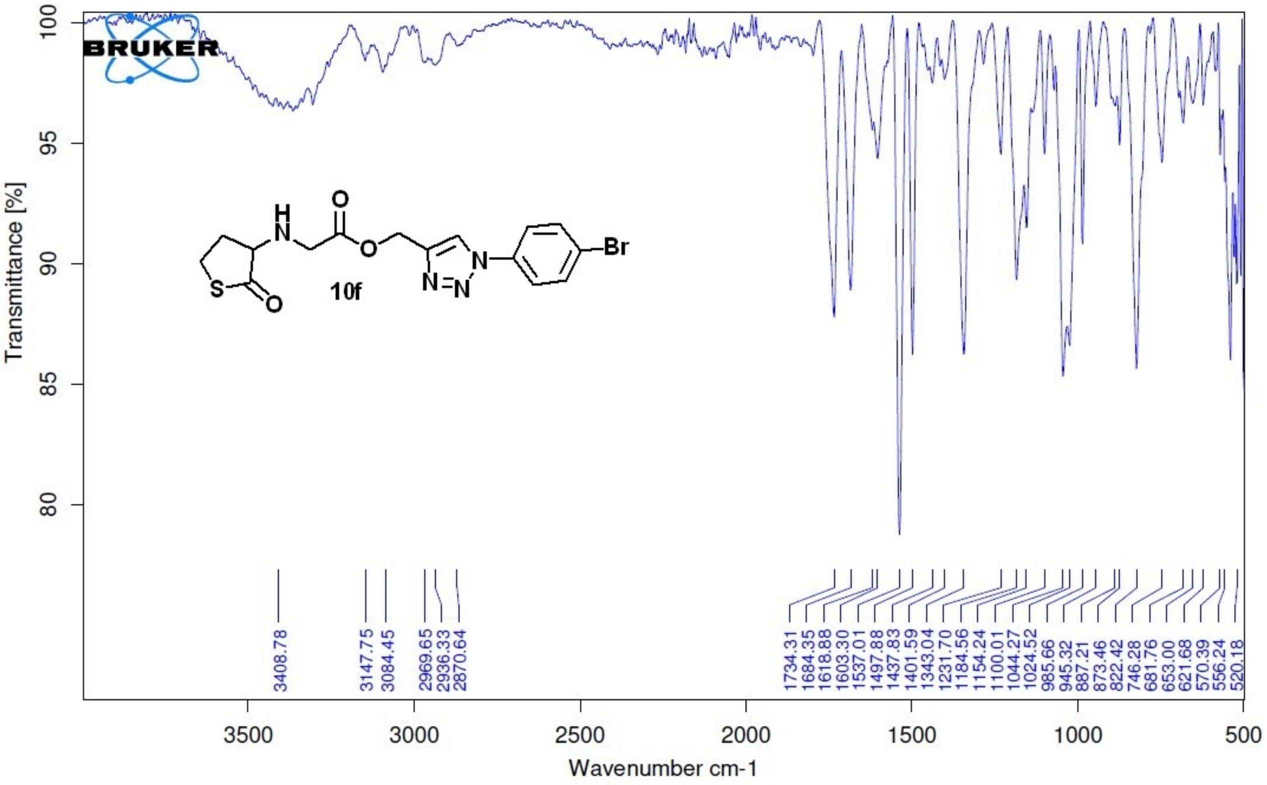

IR spectrum of 10f

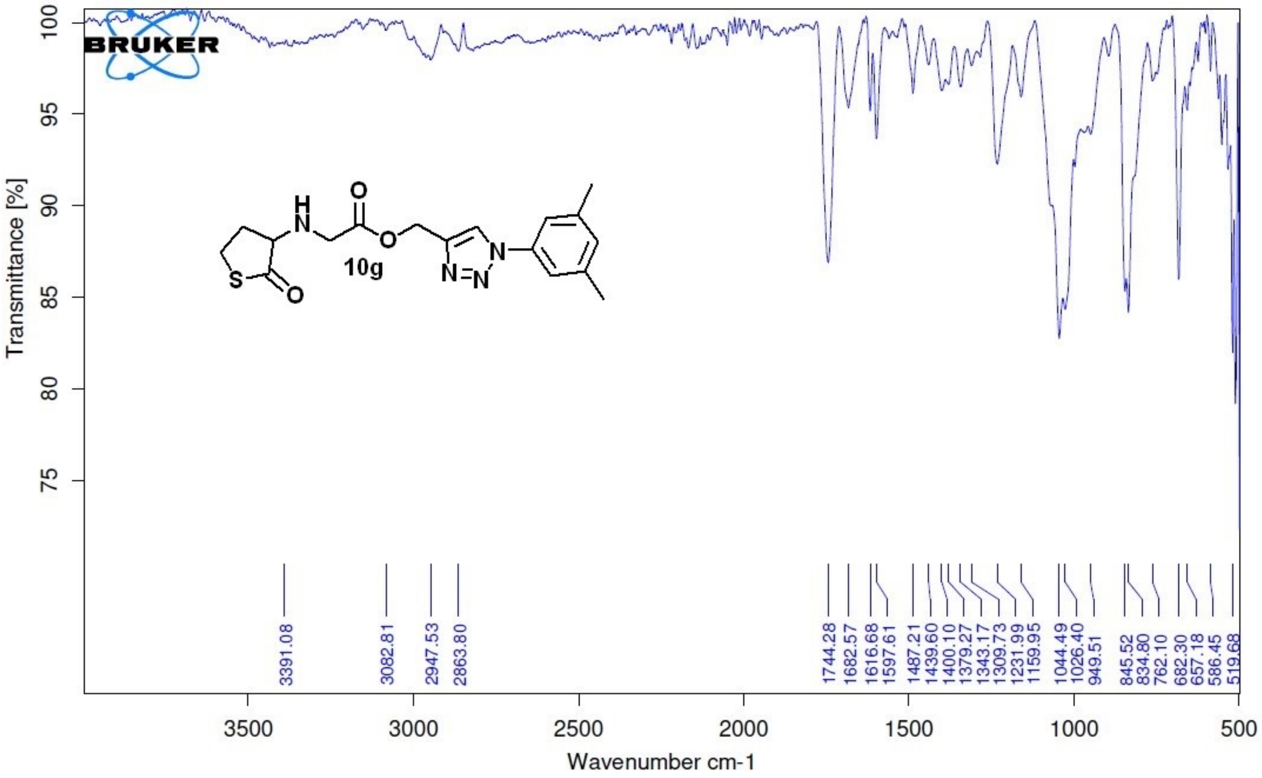

IR spectrum of 10g

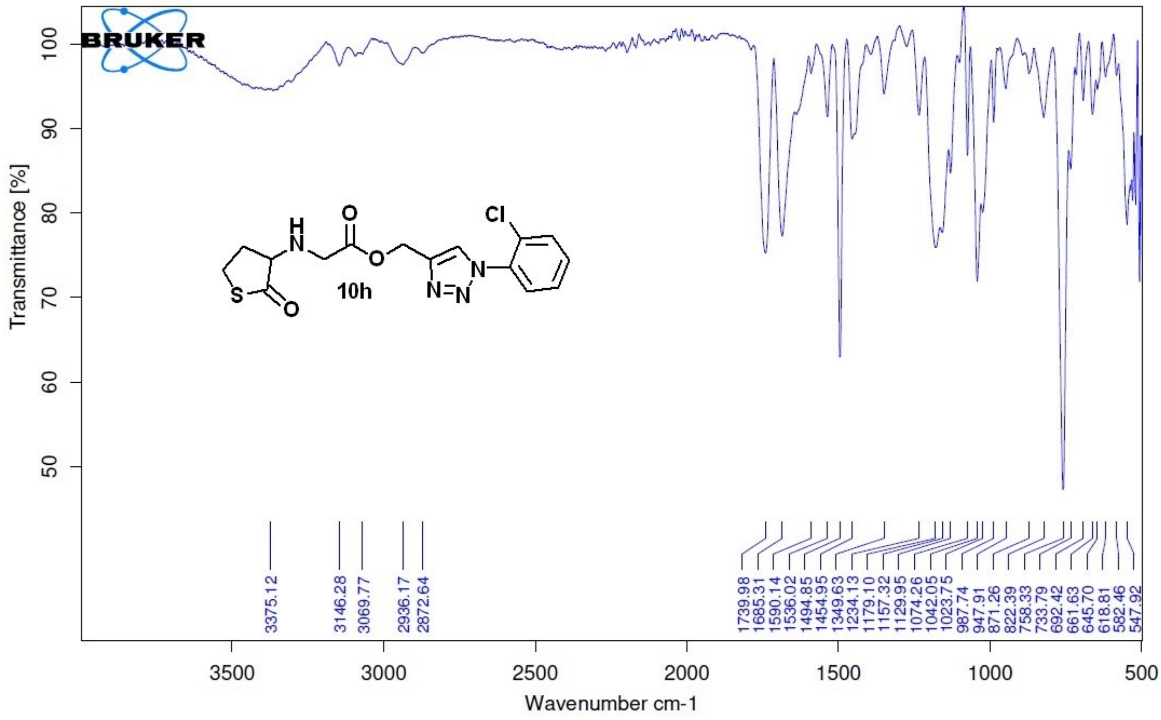

IR spectrum of 10h

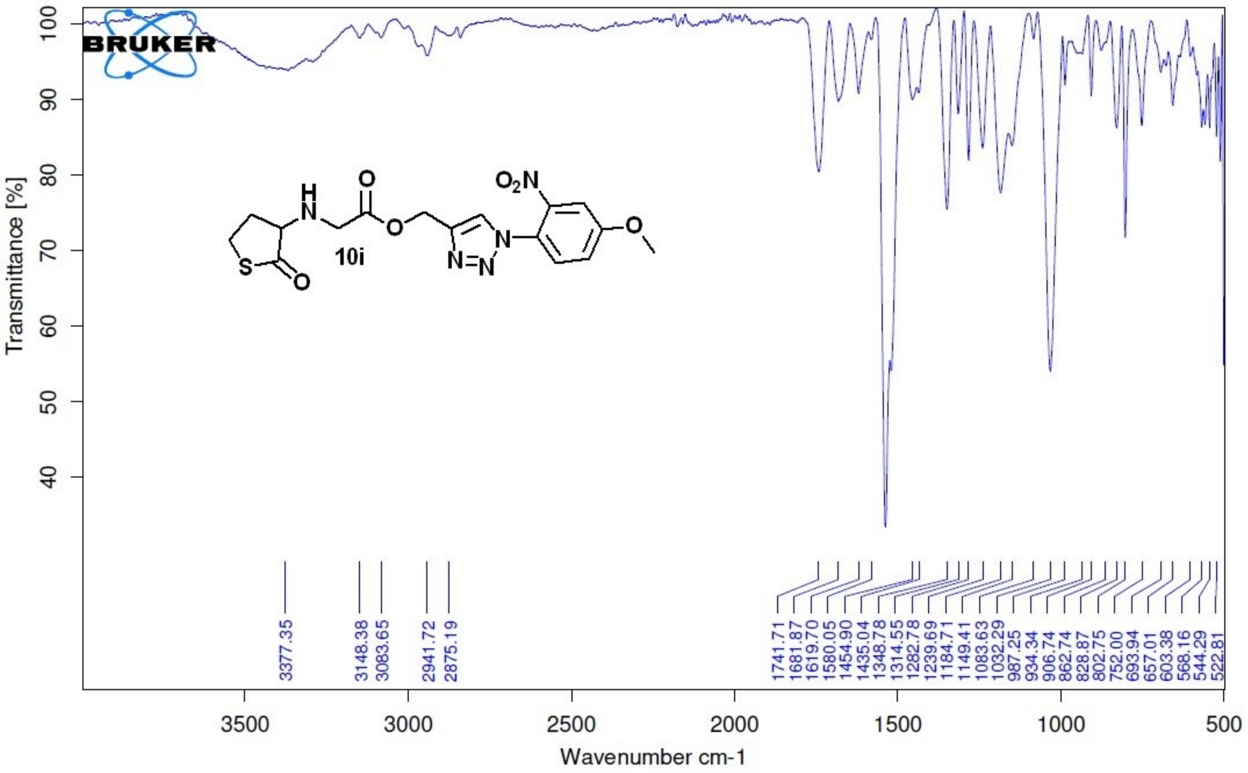

IR spectrum of 10i

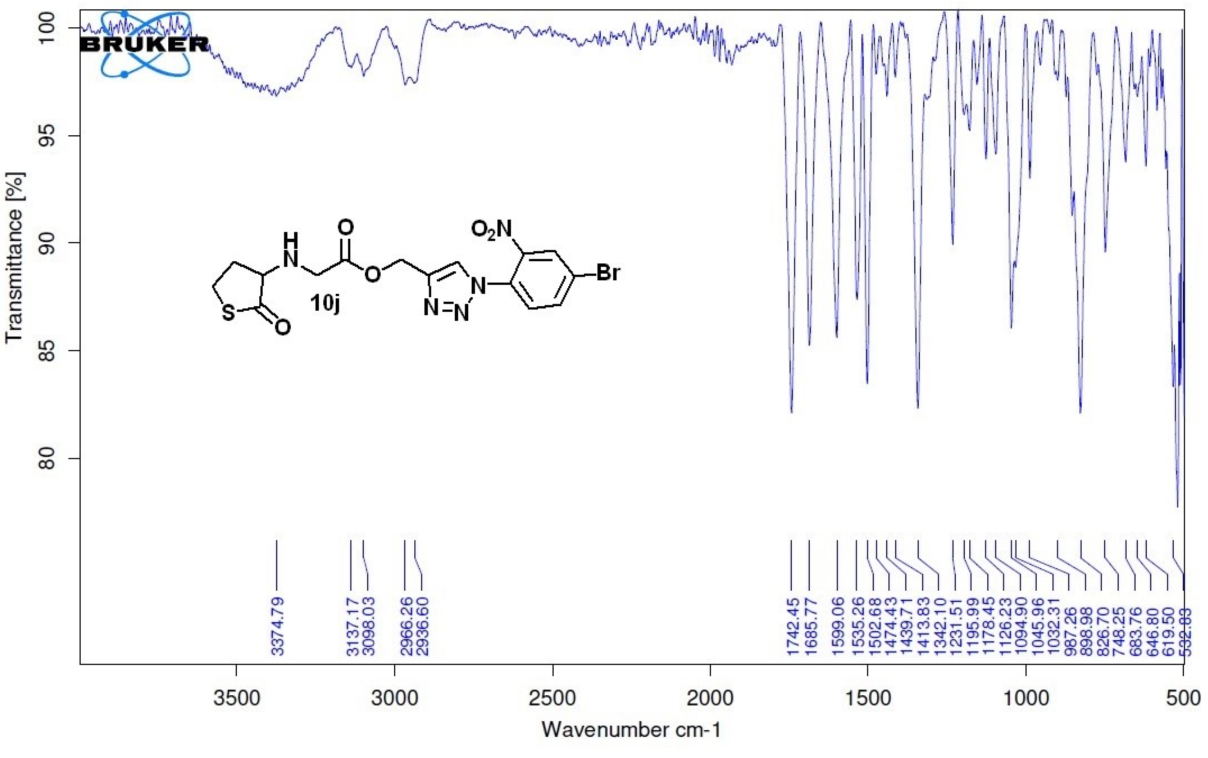

IR spectrum of 10j

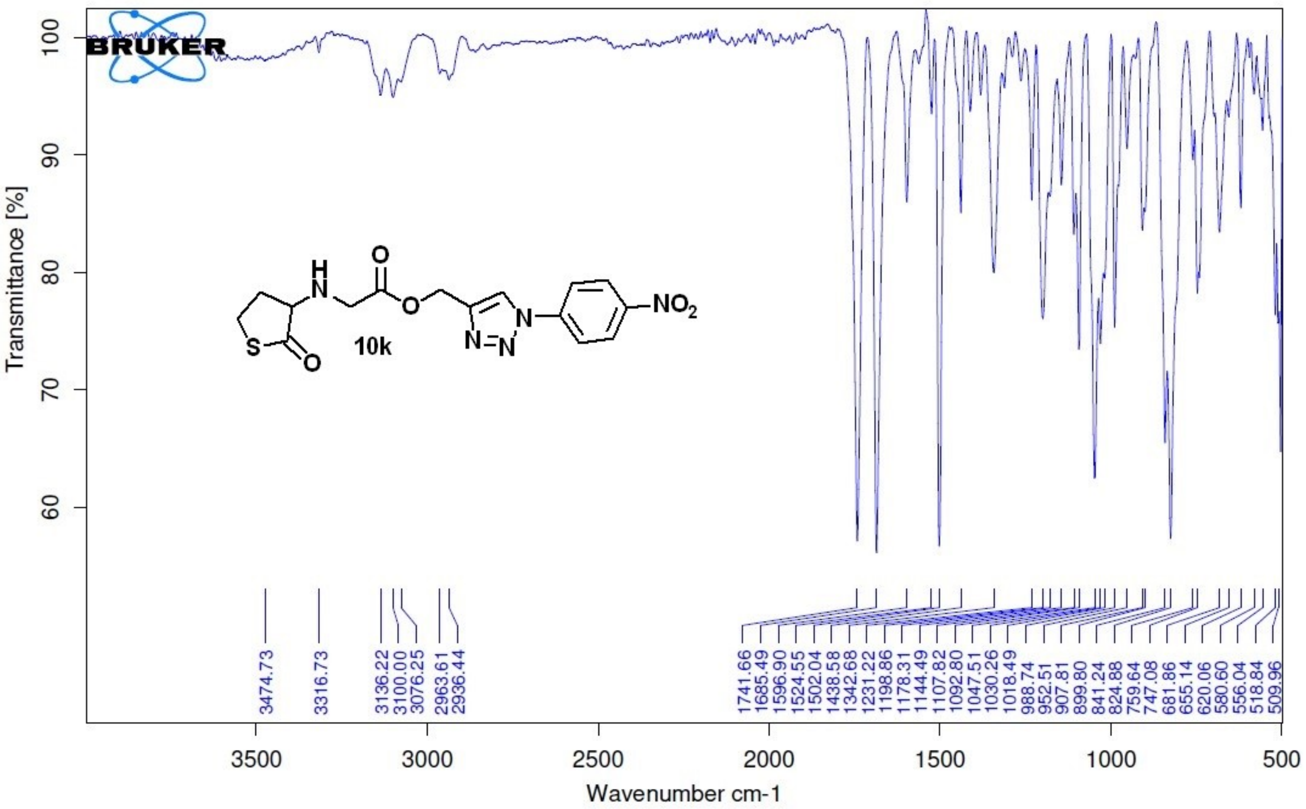

IR spectrum of 10k

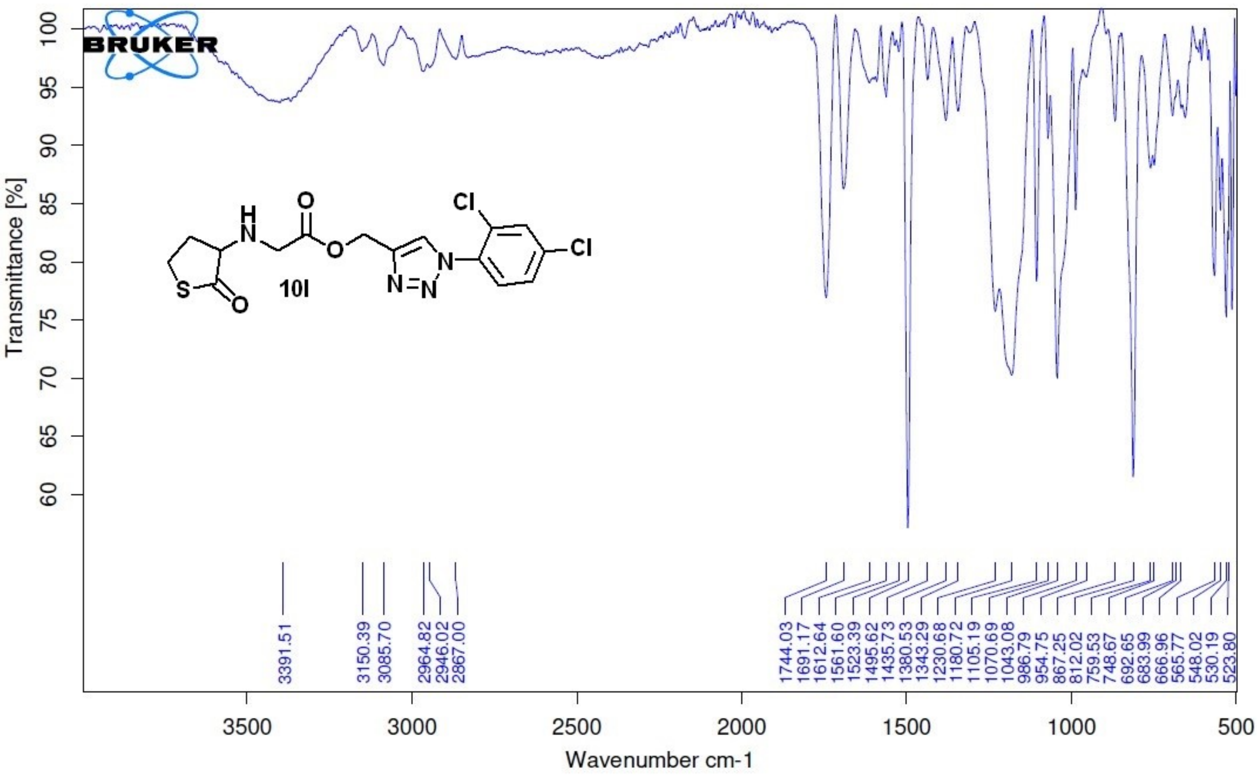

IR spectrum of 10l

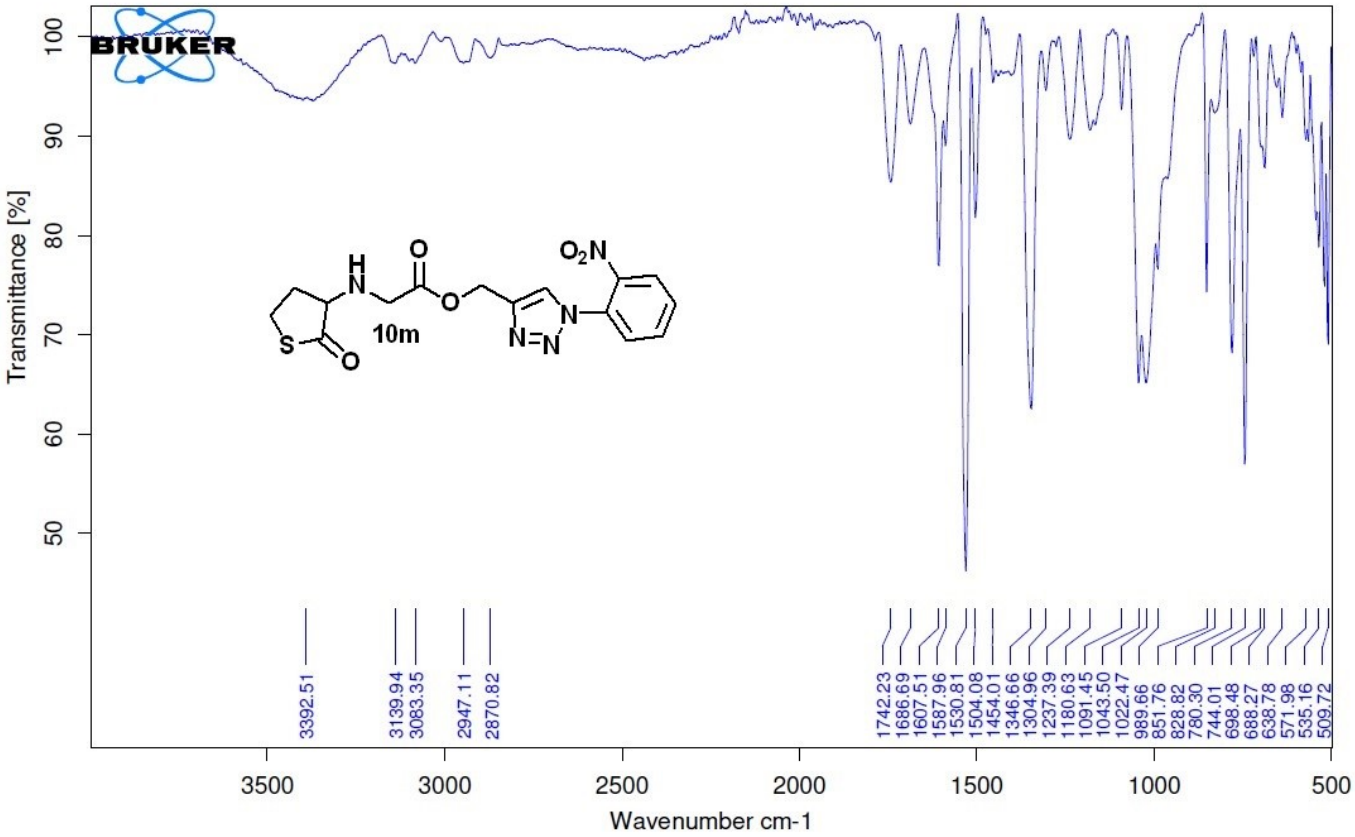

IR spectrum of 10m

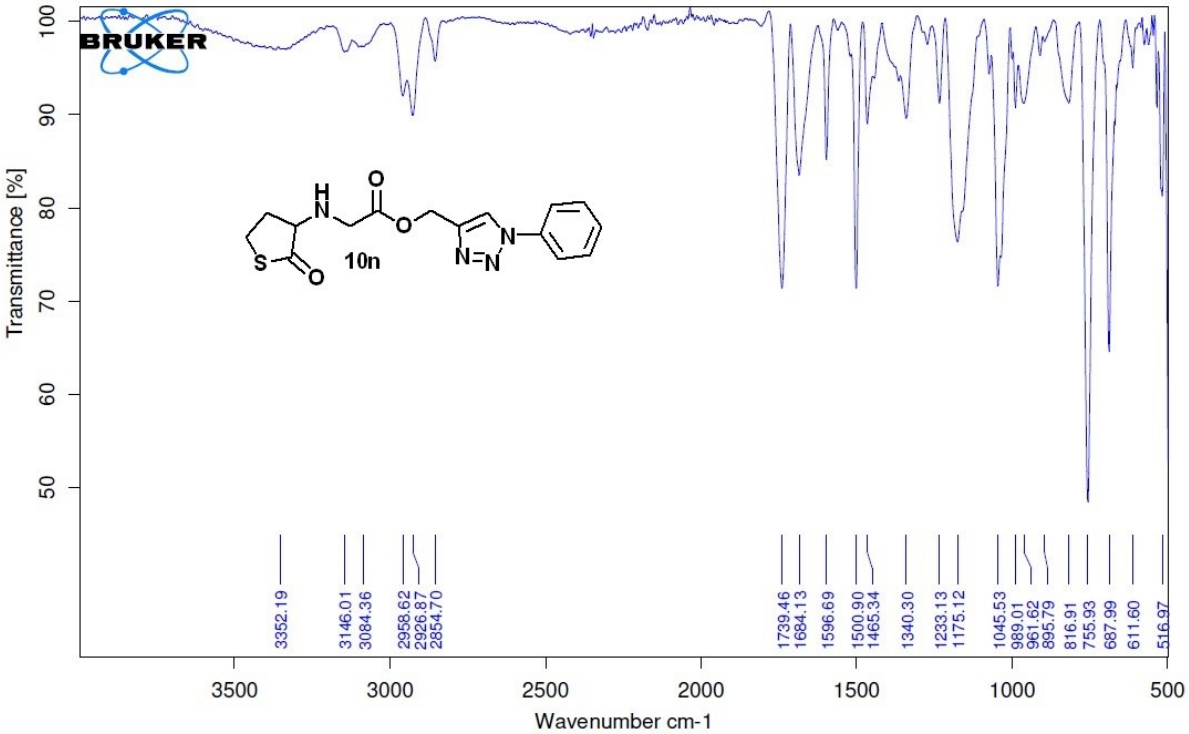

IR spectrum of 10n

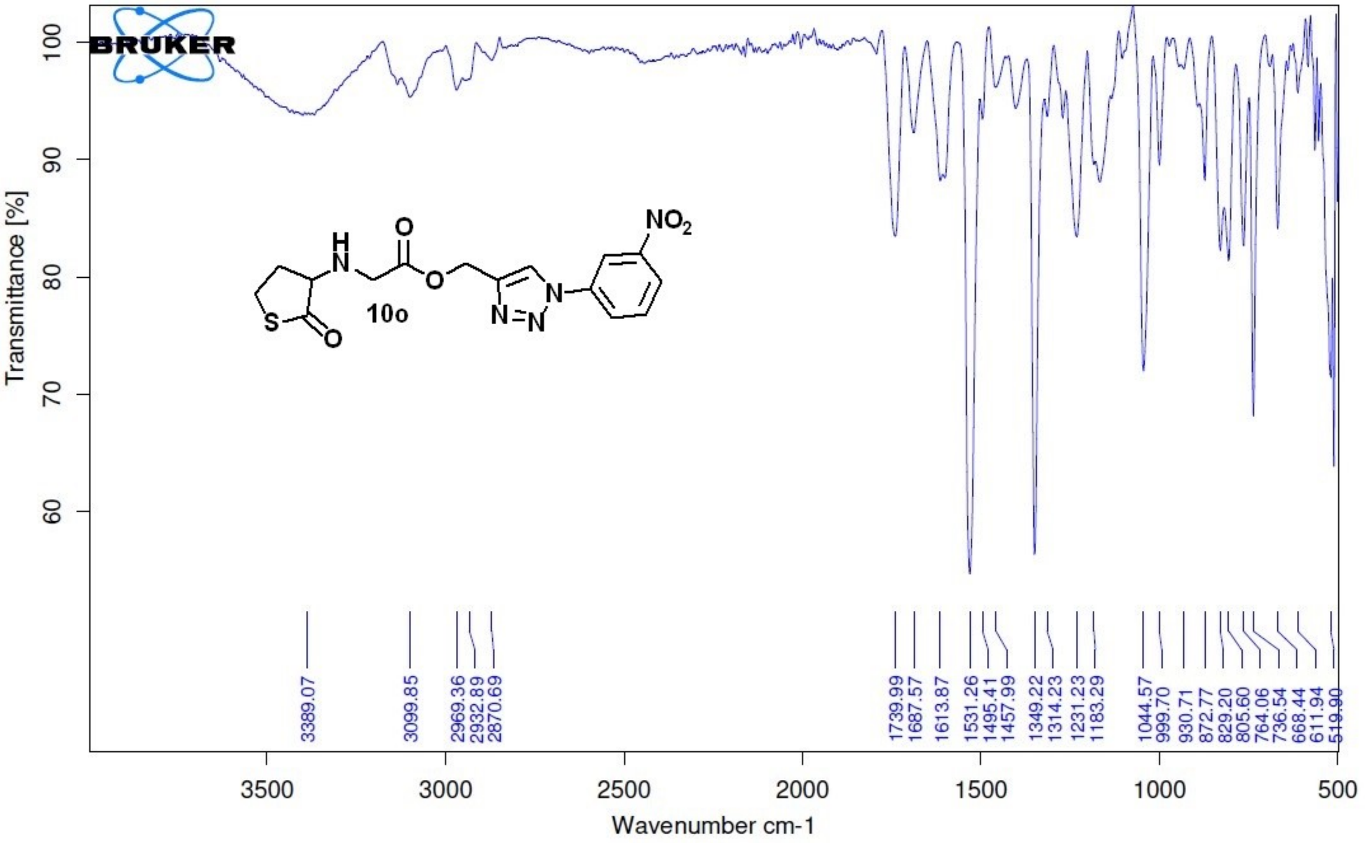

IR spectrum of 10o

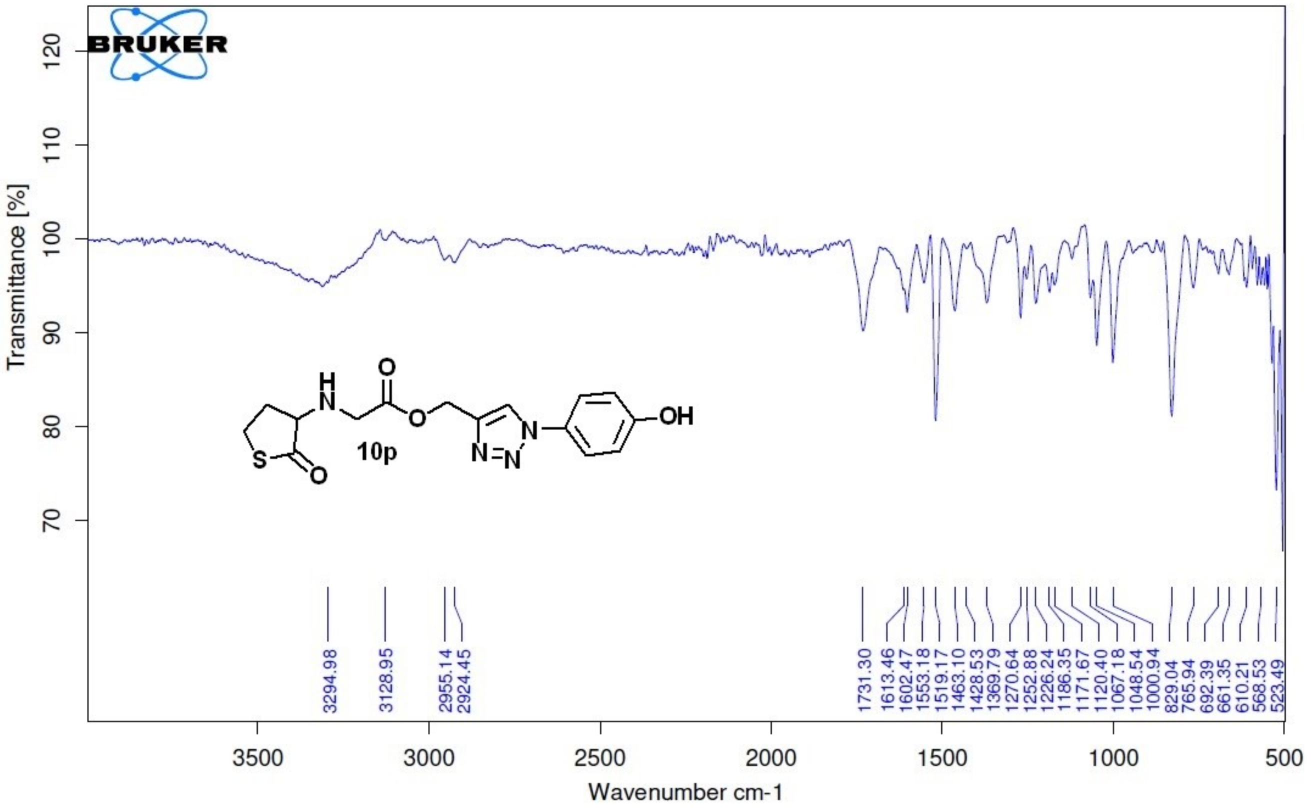

IR spectrum of 10p

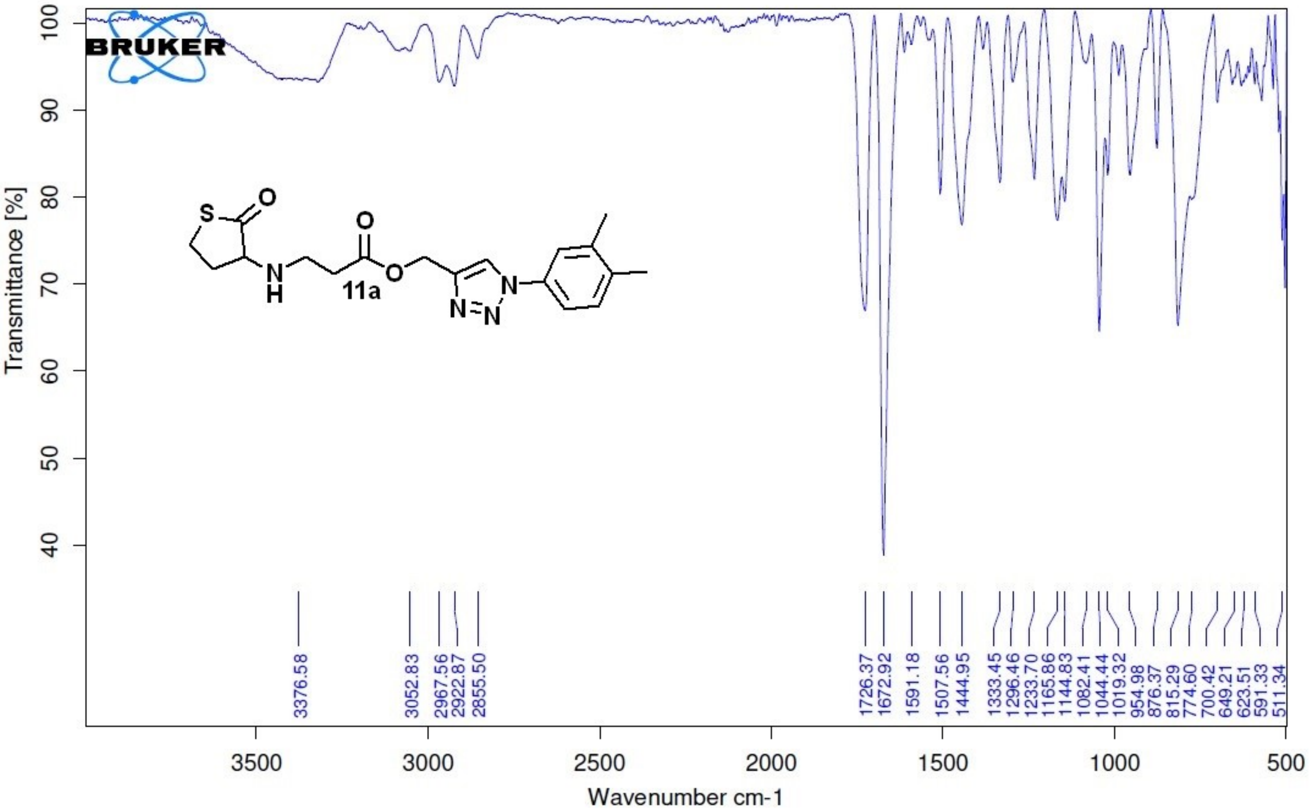

IR spectrum of 11a

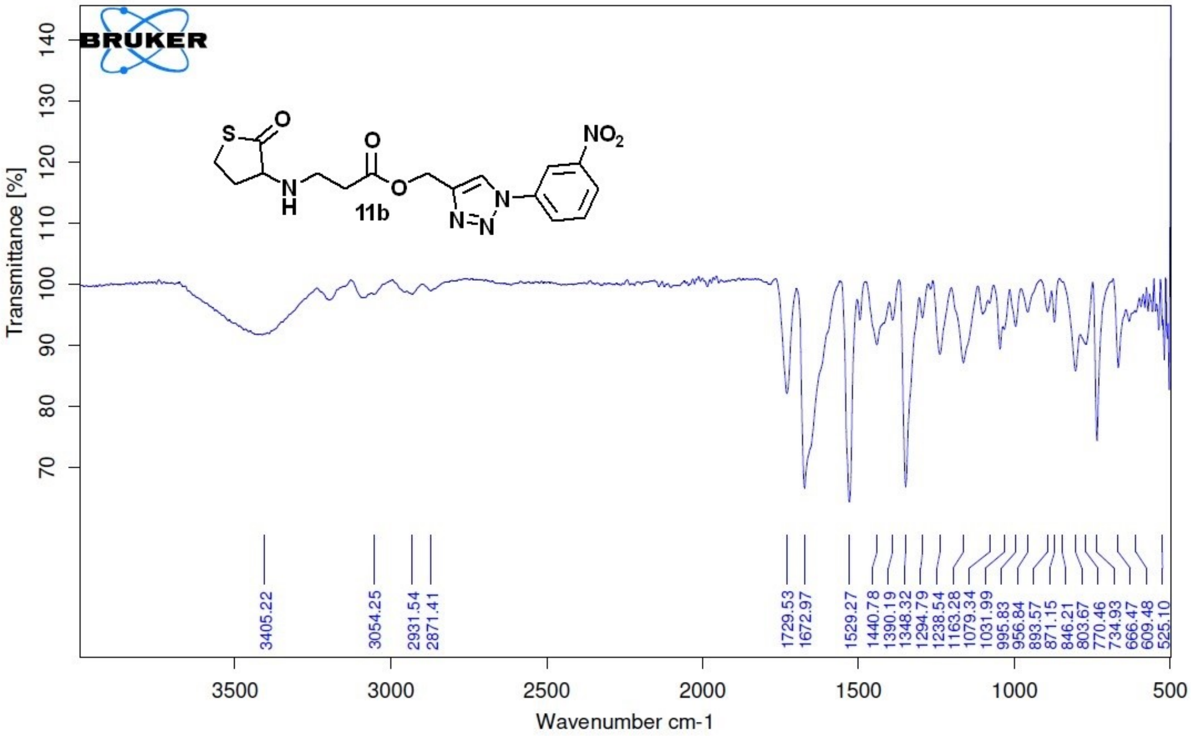

IR spectrum of 11b

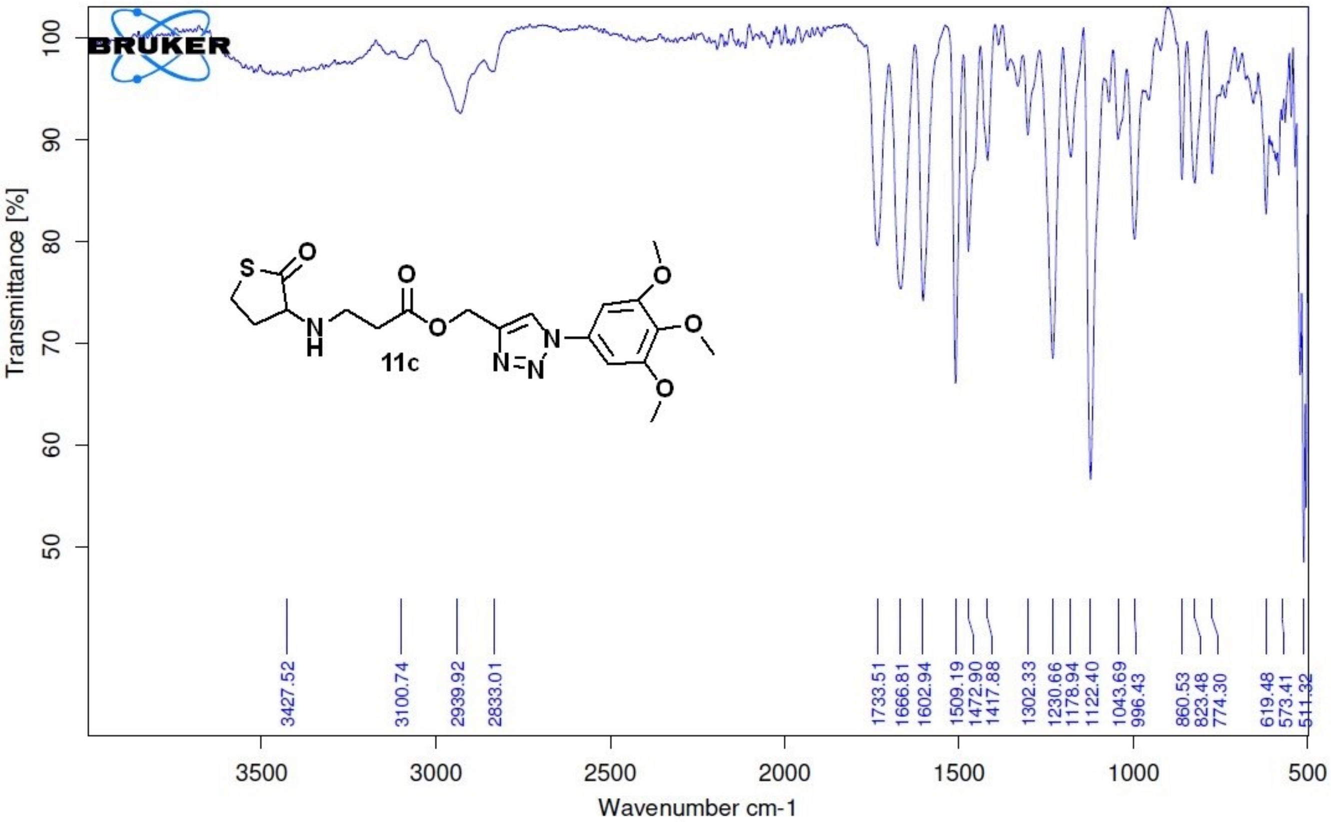

IR spectrum of 11c

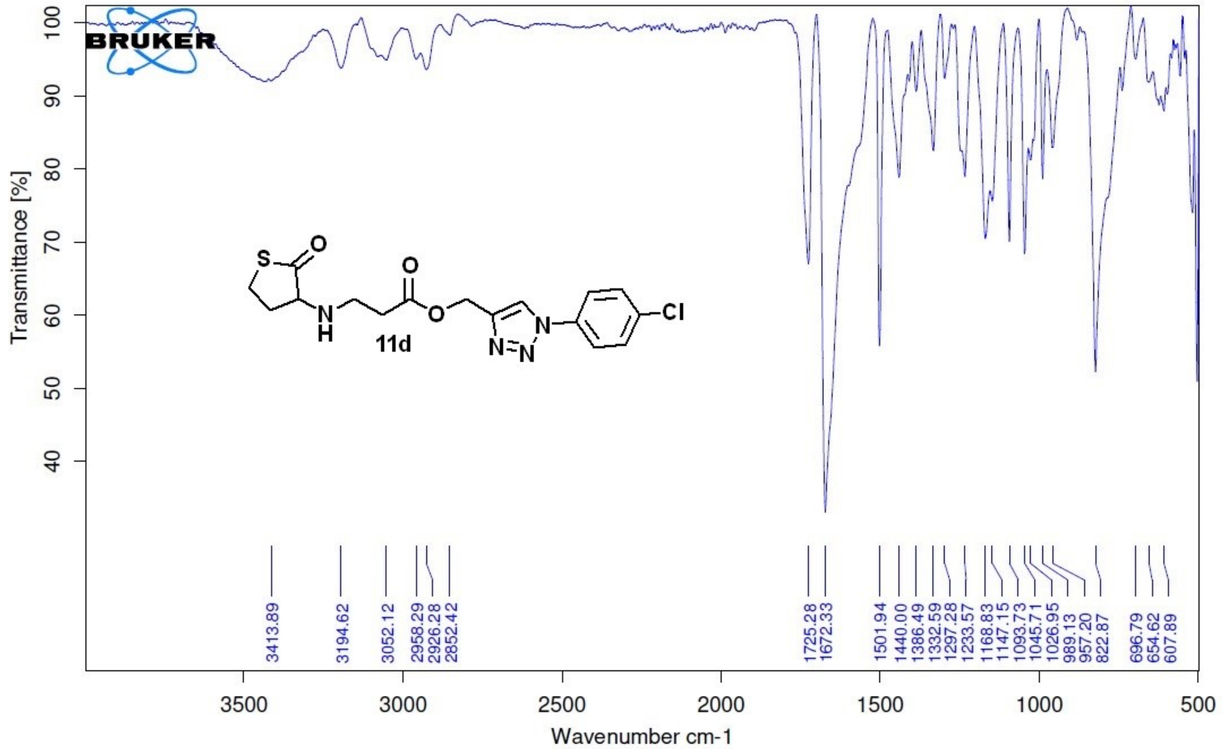

IR spectrum of 11d

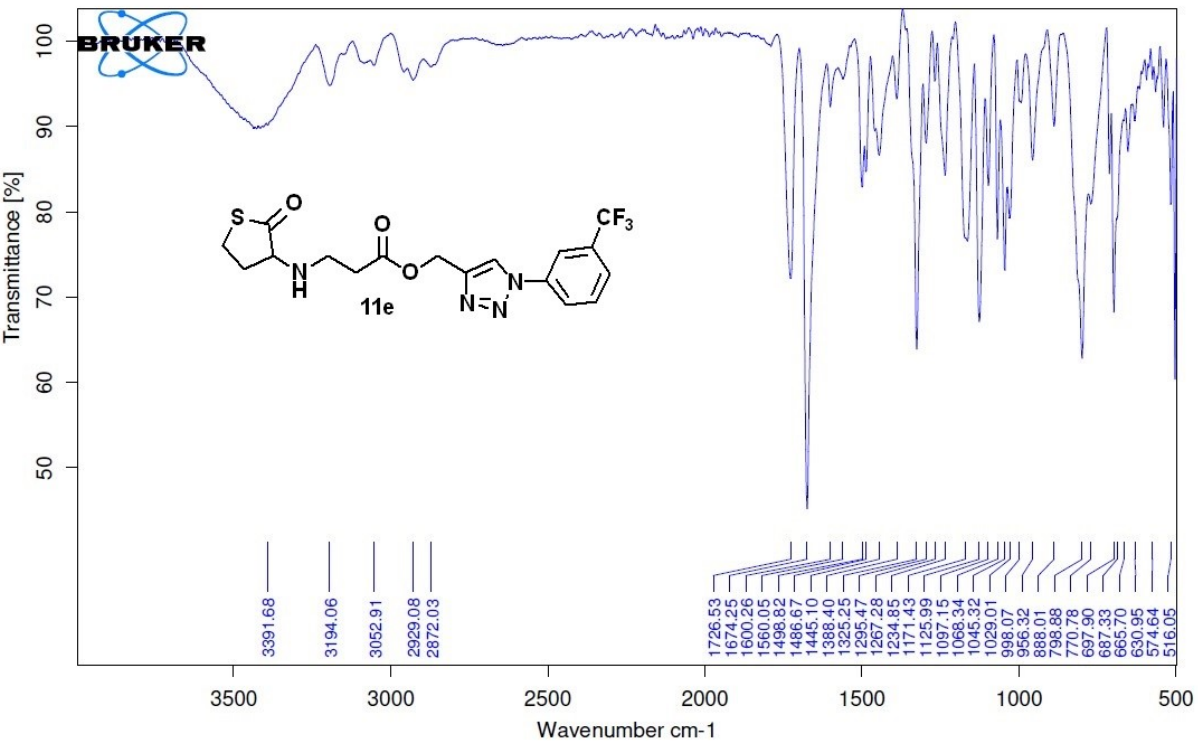

IR spectrum of 11e

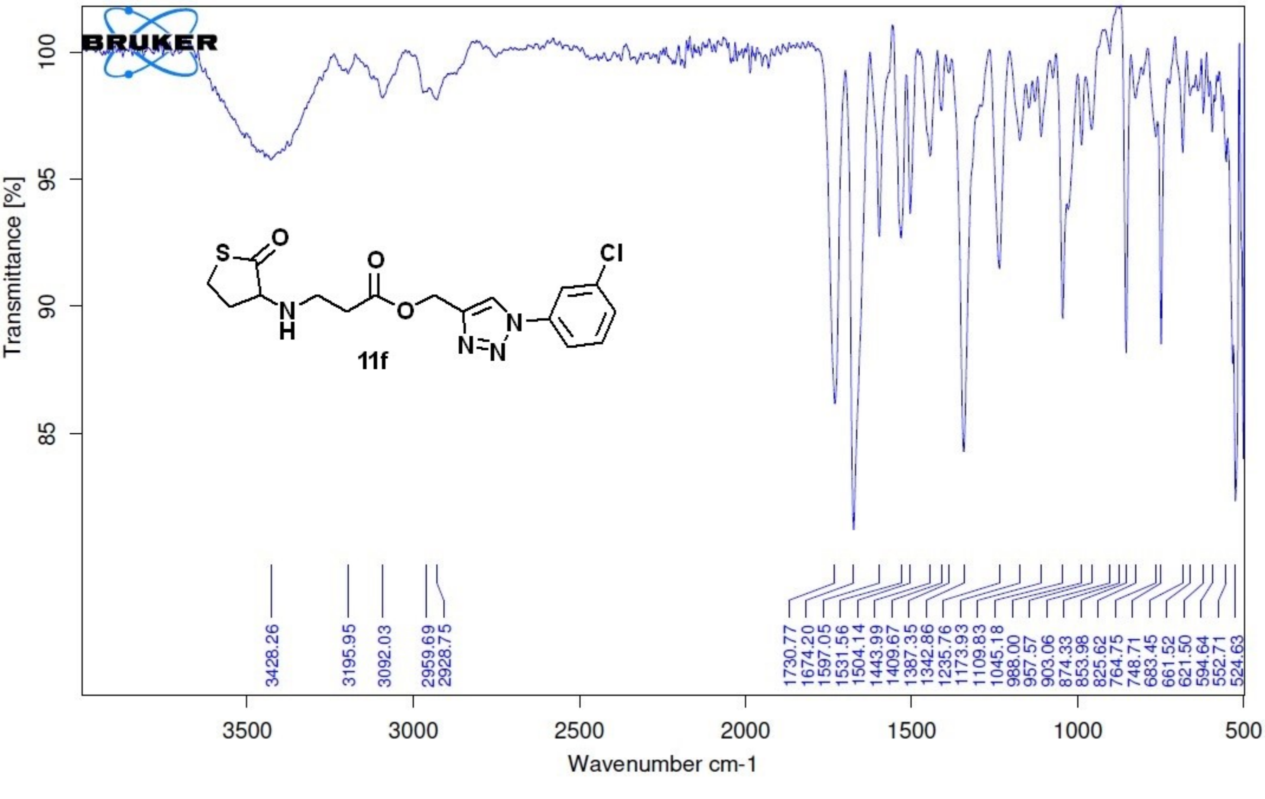

IR spectrum of 11f

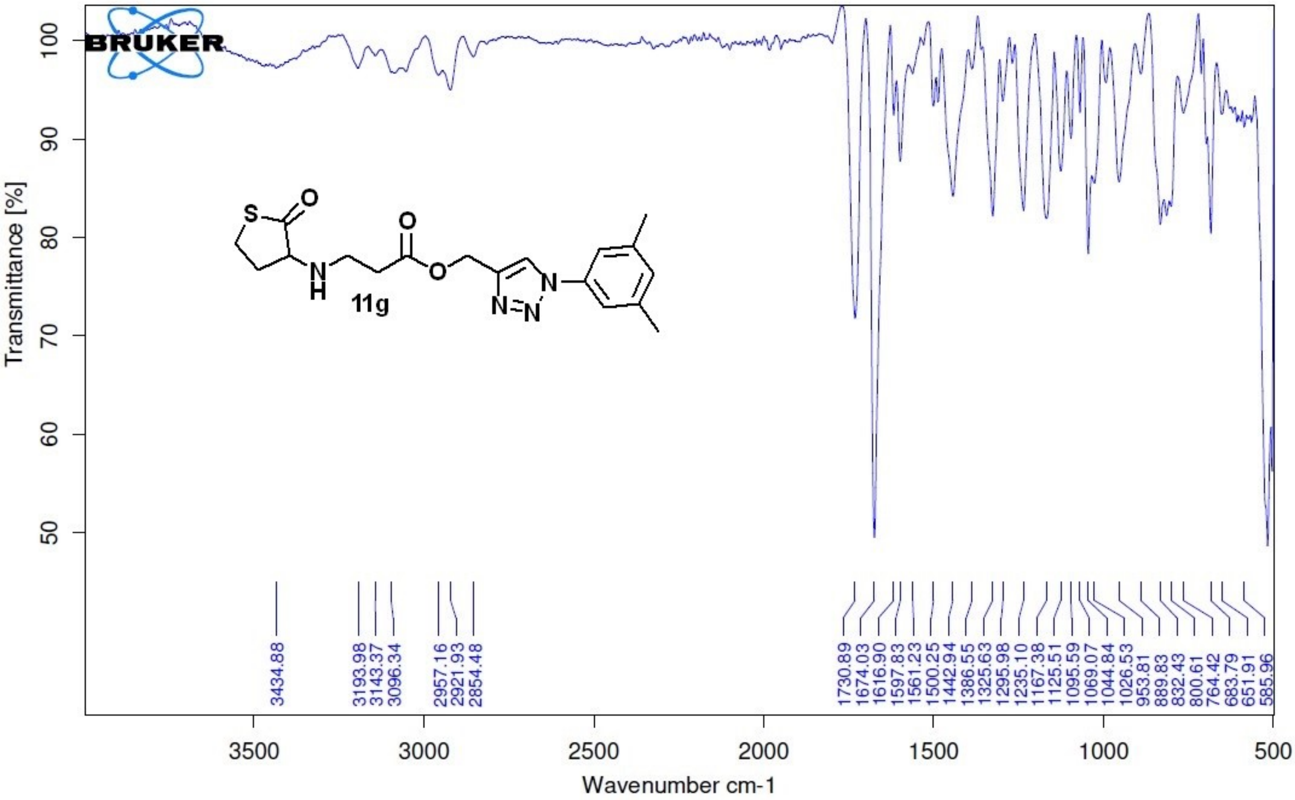

IR spectrum of 11g

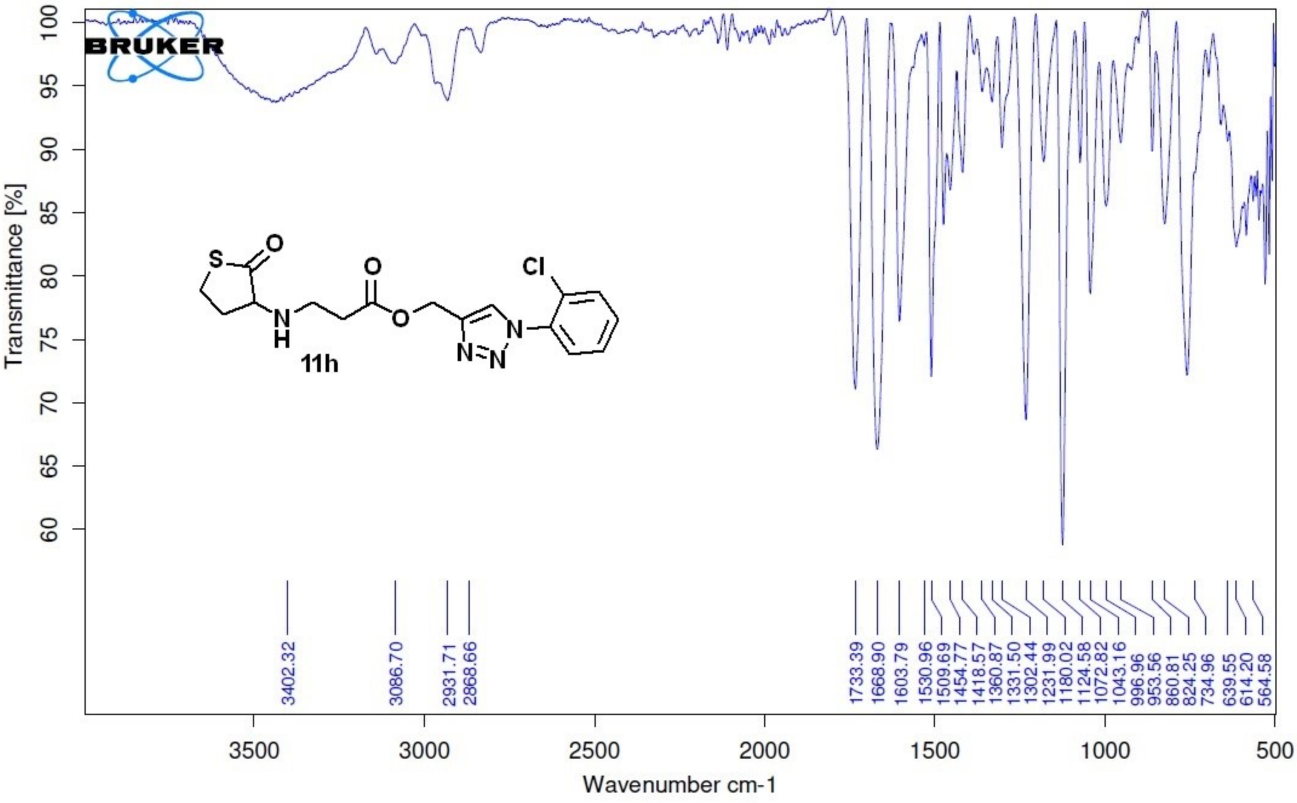

IR spectrum of 11h

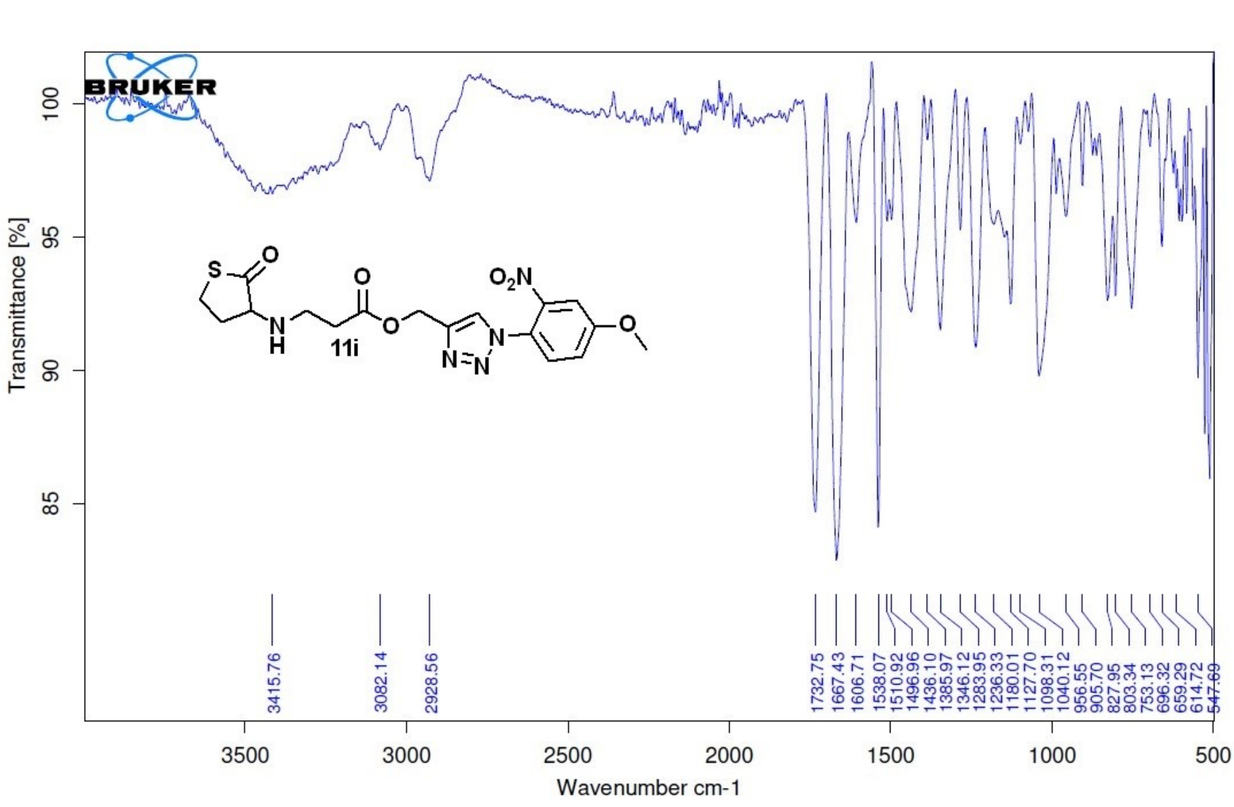

IR spectrum of 11i

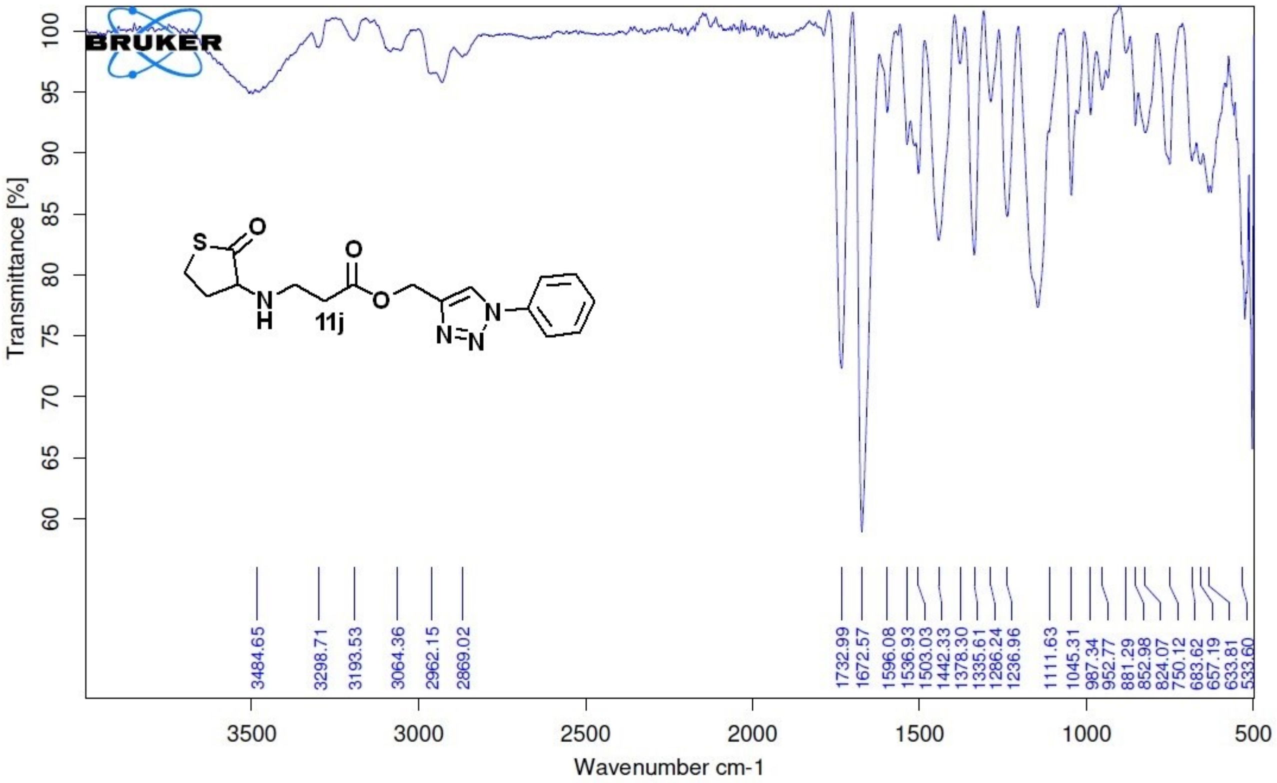

IR spectrum of 11j

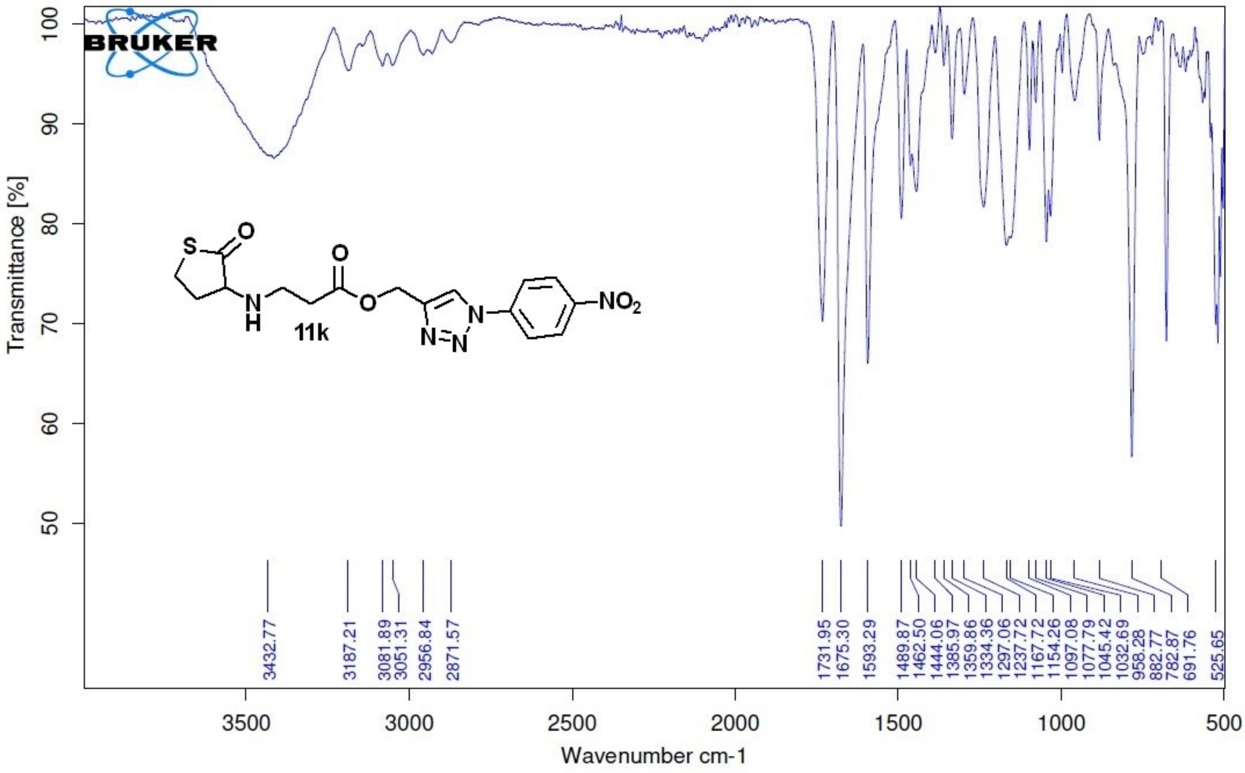

IR spectrum of 11k

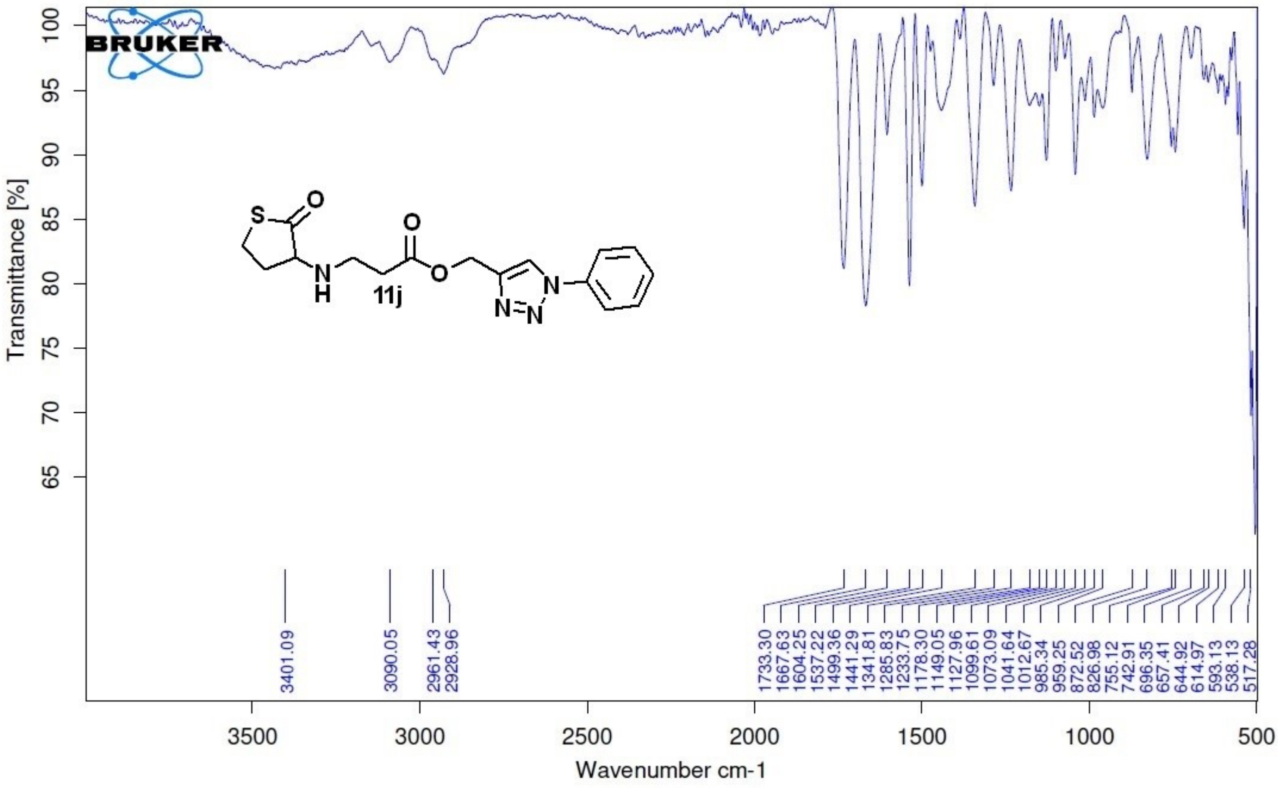

IR spectrum of 11l

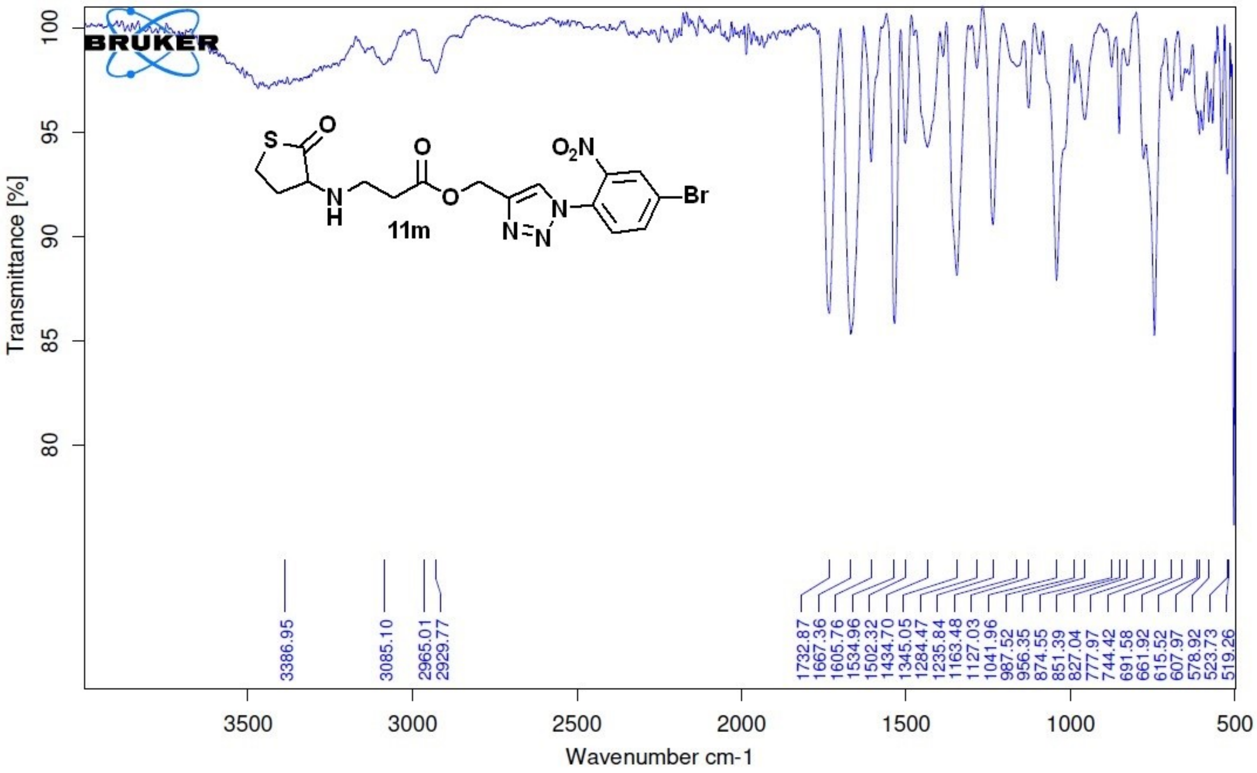

IR spectrum of 11m

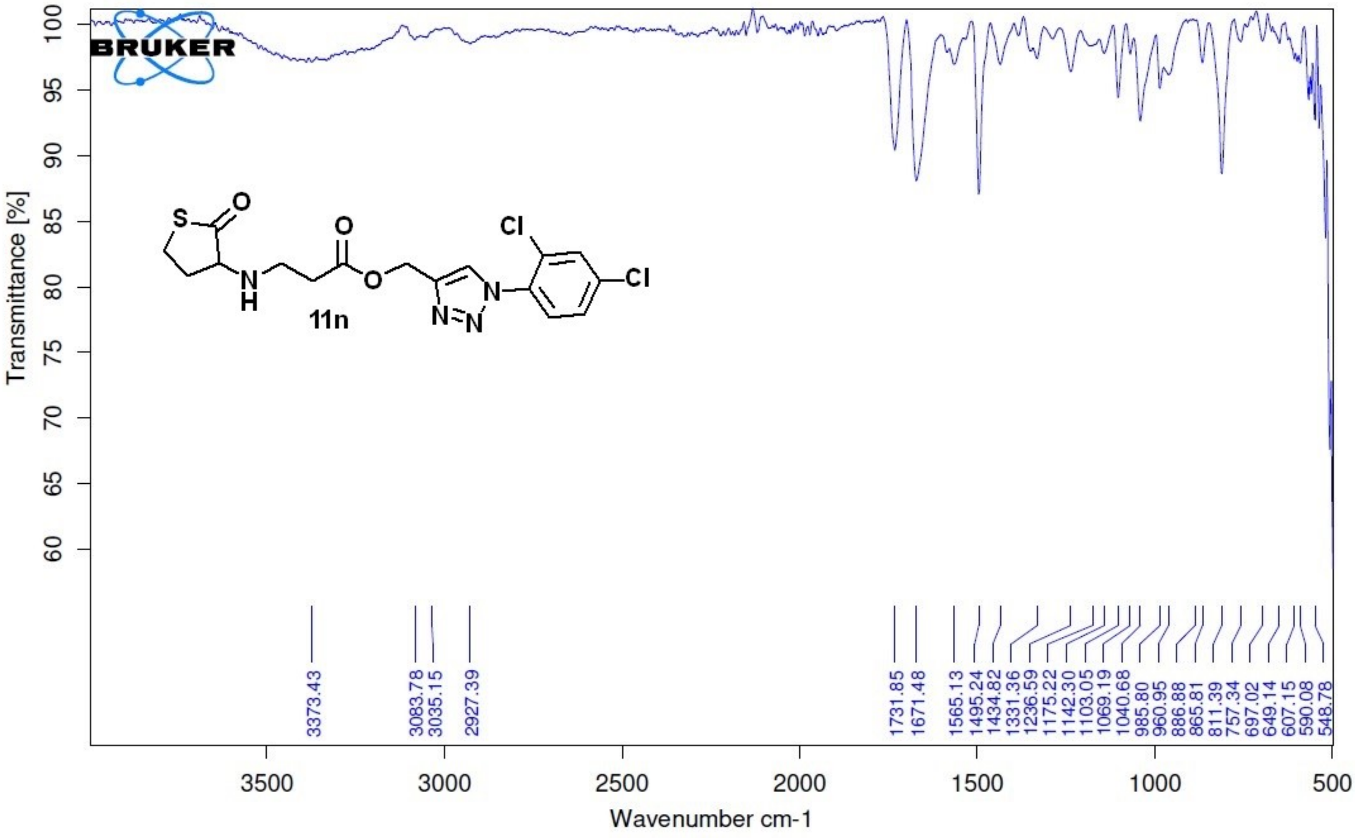

IR spectrum of 11n

9. References:

1. M. Hentzer *et al.*, *Microbiology*, 2002, **148**, 87–102.
2. M. Singh *et al.*, *Scientific Reports*, 2024, **14**, 12170.
3. G. Bennett *et al.*, *Biofilm*, 2023, **6**, 100158.
4. R. Shouman *et al.*, *Microbial Cell Factories*, 2023, **22**, 166.
5. L. Muñoz-Estrada *et al.*, *ChemMedChem*, 2025, **20**, e202400879.
6. J. L. Banks *et al.*, *Journal of Computational Chemistry*, 2005, **26**, 1752–1780.
7. Y. Yang *et al.*, *Journal of Chemical Theory and Computation*, 2021, **17**, 7106–7119.
8. R. A. Friesner, *et al.*, *Journal of Medicinal Chemistry*, 2006, **49**, 6177–6196.
9. K. J. Bowers, *et al.*, *Proceedings of the ACM/IEEE Conference on Supercomputing*, 2006, 11–17.
10. P. A. Kollman, I *et al.*, *Accounts of Chemical Research*, 2000, **33**, 889–897.
11. D. J. Evans *et al.*, *Journal of Chemical Physics*, 1985, **83**, 4069–4074.
12. G. J. Martyna, *et al.*, *Journal of Chemical Physics*, 1994, **101**, 4177–4189.
